# Triplet Metallovinylidenes of Palladium and Platinum Based on a Chelating P/Diazoalkene Ligand

**DOI:** 10.1002/anie.202516032

**Published:** 2025-11-10

**Authors:** Max Amann, Maria Drosou, Tarek Al Said, Alexander Allgaier, Yury Kutin, Patrick W. Antoni, Julian J. Holstein, Müge Kasanmascheff, Joris van Slageren, Alexander Schnegg, Dimitrios A. Pantazis, Max M. Hansmann

**Affiliations:** ^1^ Fakultät für Chemie und Chemische Biologie Technische Universität Dortmund Otto‐Hahn‐Str.6 44227 Dortmund Germany; ^2^ Max‐Planck‐Institut für Kohlenforschung Kaiser‐Wilhelm‐Platz 1 45470 Mülheim an der Ruhr Germany; ^3^ Technische Universität Darmstadt Department of Chemistry Quantum Chemistry, Peter‐Grünberg‐Str. 4 64287 Darmstadt Germany; ^4^ Joint lab EPR4Energy Helmholtz‐Zentrum Berlin für Materialien und Energie Hahn‐Meitner‐Platz 1 14109 Berlin Germany; ^5^ Institut für Physikalische Chemie Universität Stuttgart Pfaffenwaldring 55 70569 Stuttgart Germany; ^6^ Max‐Planck‐Institute for Chemical Energy Conversion Stiftstrasse 34 – 36 45470 Mülheim an der Ruhr Germany

**Keywords:** Diradicals, Metallocarbenes, Reactive intermediates, THz‐EPR spectroscopy, Triplet vinylidenes

## Abstract

Triplet carbenes featuring a metal adjacent to the carbene center (metallocarbenes; R─C─M) are an emerging class of diradicals within the field of reactive intermediates. Here, we report the synthesis of the first spectroscopically characterized triplet metallovinylidenes (R─C→M; M = Pt, Pd). The synthetic access is based on a rigid P/C chelating diazoalkene ligand and its coordination to Pt and Pd. The C/P chelating ligand geometrically constrains the R─C→M angle and inhibits free bending. Irradiation of the free diazoalkene ligand generates a triplet vinylidene, characterized by Q‐band electron paramagnetic resonance (EPR) spectroscopy. Irradiation of the metal coordination complexes (Pt and Pd) affords triplet metallovinylidenes, which were characterized at low temperatures including photochemically triggered *in crystallo* X‐ray diffraction. Combined FD‐FT THz‐EPR spectroscopy and SQUID measurements allowed the determination of the large triplet zero‐field splitting (ZFS) with *D* values of 124.5 cm^−1^ (Pt) and 8.0 cm^−1^ (Pd) in excellent agreement with the electronic structure obtained by high‐level quantum chemical calculations.

## Graphical abstract

A new class of chelating diazoalkenes is presented and their coordination complexes with Pd and Pt are characterized. Irradiation triggers N_2_ loss and gives access to the first triplet metallovinylidenes spectroscopically characterized utilizing FD‐FT THz‐EPR spectroscopy and SQUID measurements. Structural verification is given by in *crystallo* X‐ray diffraction while computational studies support the unique bonding of the new diradical class.

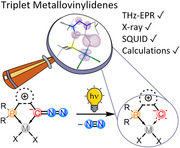

Triplet carbenes (R^1^─C─R^2^) are divalent carbon compounds with two unpaired electrons, which have been studied for decades as fundamental intermediates in organic chemistry.^[^ ^1^ ^]^ In particular diazoalkanes bearing aryl or diaryl substituents have been employed as precursors to photochemically access triplet carbenes of the structure Ar─C─Ar, typically studied in matrix isolation or in organic glasses by IR or EPR spectroscopy.^[^ ^2^ ^]^ Currently there is a growing interest in metallocarbenes, in which one substituent is exchanged by a metal fragment (R─C─M), and which can exist either in a singlet^[^ ^3^, ^4^, ^5^, ^6^ ^]^ or triplet ground state (Figure 1a). Diazoalkanes substituted by late‐transition metals are well‐known,^[^ ^7^, ^8^, ^9^, ^10^, ^11^ ^]^ and the photolysis products suggest the formation of transient metallocarbenes.^[^ ^12^, ^13^, ^14^ ^]^ However, it remains highly challenging to characterize these elusive metal‐flanked diradicals. In 2024, Schneider and Holthausen were able to characterize the first triplet metallocarbenes of the structure ([M]−C−SiMe_3_) based on a PNP‐pincer Pt/Pd entity (**I**; Figure 1).^[^ ^15^ ^]^ *In crystallo* photochemical experiments proved the release of N_2_ with formation of a fairly bent M─C─Si fragment (∠156°) compared to the calculated singlet carbene (133°). SQUID data confirmed a triplet ground state with an isotropic *g*‐factor (*g*  =  2) and ZFS [*D*  =  5.1 cm^−1^ for Pd and 73 cm^−1^ for Pt] in agreement with a metallocarbene with a M─C single bond as opposed to a carbyne (M≡C─R) description. This work was recently extended to a Wolff rearrangement study by switching from a silyl to an ester group.^[^ ^16^, ^17^ ^]^ Recently, the interest in triplet metallocarbenes was extended to the heavy p‐block. Munz and co‐workers performed *in crystallo* photochemical transformations with the Pb‐diazo precursor to access [Pb]−C−SiMe_3_ (**II**; Figure 1a).^[^ ^18^ ^]^ Note that none of these diradicals were studied by EPR spectroscopy due to their high ZFS values (for Pb‐substituted carbene, DFT predicted: *D*  =  112–309 cm^−1^), rendering them undetectable by conventional EPR spectroscopy.^[^ ^18^ ^]^

**Figure 1:**
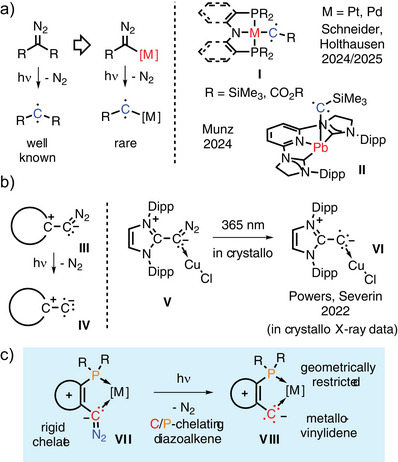
a) Triplet carbenes and triplet metallocarbenes; b) diazoalkenes as precursors to Cu‐vinylidenes; c) triplet metallovinylidenes described in this work.

In 2021, the Severin group and Hansmann group independently described the first room‐temperature‐stable diazoalkenes [R_2_C═C═N_2_ (**III**); Figure 1b],^[^ ^19^, ^20^, ^21^, ^22^, ^23^ ^]^ which upon photoexcitation liberate dinitrogen to form free triplet vinylidenes (**IV**).^[^ ^24^, ^25^ ^]^ In contrast to triplet carbenes, triplet vinylidenes feature a monosubstituted carbon atom. Diazoalkenes can also act as neutral donor ligands to form transition‐metal complexes. Coordination to vanadium or Cp*Ir leads to spontaneous N_2_ liberation and formation of vinylidene complexes,^[^ ^26^, ^27^ ^]^ while Cr,^[^ ^28^ ^]^ Rh,^[^ ^20^ ^]^ Pd,^[^ ^20^ ^]^ Au,^[^ ^20^ ^]^ and Cu^[^ ^29^ ^]^ form the carbon adducts with an intact diazo moiety. Severin and Powers could show that irradiation of the Cu diazoalkene complex **V** generated Cu‐vinylidene **VI** based on a *crystal‐to‐crystal* transformation using synchrotron X‐ray irradiation.^[^ ^29^ ^]^ While DFT studies favored a triplet species, no further experimental data were given. Interestingly, calculations indicated a flat potential energy surface for the bending of the fragment with a minimum at a linear structure while the bent *in‐crystallo* structure was assumed to be not fully relaxed.

In spite of these advances, the classes of triplet metallocarbenes and metallovinylidenes are still extremely rare and poorly characterized. Considering their diradical nature,^[^ ^30^ ^]^ further fundamental understanding is required in order to develop applications for instance as magnetic materials or spintronic devices.^[^ ^31^, ^32^ ^]^ Electronically, the few reported triplet metallocarbenes favor a linear structure in the triplet and a bent structure in the singlet state. In order to remove the flexibility in bending of the C─C─M entity, we targeted a chelating diazoalkene ligand, hence to constrain the metal fragment into a five‐membered ring (Figure 1c). Here, we report the first chelating diazoalkene ligand which sets the stage for the characterization of metallovinylidenes by a crystal‐to‐crystal X‐ray diffraction study as well direct ZFS determination by FD‐FT THz‐EPR spectroscopy and SQUID magnetometry supported by quantum chemical calculations.

## Results and Discussion

### Synthesis of a Chelating Diazoalkene

**Scheme 1:**
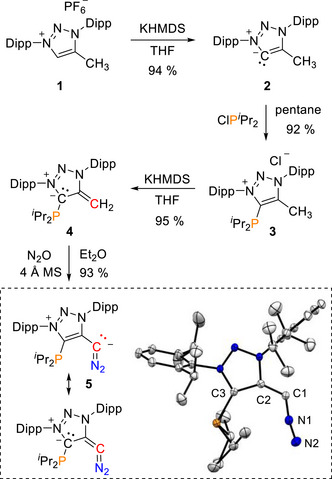
Synthesis of the chelating P/diazoalkene ligand **5**. Dipp = 2,6‐diisopropylphenyl. X‐ray solid state structure of **5**. A molecule of Et_2_O omitted for clarity. Selected bond parameters in [Å] and [°]: C1–C2 1.3951(16), C2–C3 1.4216(16), N1–C1 1.2660(18), N1–N2 1.1551(17), N2–N1–C1 169.08(13), and N1–C1–C2 123.55(12).

The synthesis of the chelating diazoalkene ligand started with the 1,2,3‐triazolium salt **1** obtained by (3+2) cycloaddition of a triazene with propyne (see Supporting Information).^[^ ^33^ ^]^ Deprotonation of **1** by potassium bis(trimethylsilyl)amide (KHMDS) exclusively resulted in the formation of the unknown free mesoionic carbene **2** (Scheme 1), supported by a low field ^13^C NMR carbene resonance at *δ*  =  201.3 ppm.^[^ ^34^ ^]^ Interestingly, a competitive deprotonation at the methyl group to give the mesoionic *N*‐heterocyclic olefin (mNHO) was not observed.^[^ ^35^ ^]^ Upon addition of the free carbene to ClP*^i^* Pr_2_ the cationic phosphine **3** was formed in high yield (92%). Deprotonation of the salt with KHMDS at the methyl group afforded the deeply red colored, phosphorus based chelating P‐mNHO **4** in high yield. **4** shows two strongly high‐field shifted ^1^H NMR [δ(^1^H)  =  2.66/3.10 ppm] and ^13^C NMR signals [δ(^13^C)  =  47.6 ppm] for the exocyclic CH_2_ moiety, in agreement with mNHO formation.^[^ ^35^ ^]^ Note, while there are several NHOs,^[^ ^36^, ^37^, ^38^, ^39^, ^40^ ^]^ chelating mNHOs are very rare.^[^ ^41^ ^]^ Recently, Košmrlj and coworkers targeted the synthesis of a chelating 1,2,3‐triazole derived P‐mNHO but only observed unselective decomposition.^[^ ^42^ ^]^ In our case, P‐mNHO **4** is stable over weeks at room temperature if kept under inert conditions.

Upon exposure of P‐mNHO **4** to 2–3 bar of nitrous oxide (N_2_O), a color change from dark red to light orange occurred to give selectively the stable P‐functionalized diazoalkene **5** in excellent yield (93%). Interestingly, the reaction rate is significantly slower (on the order of two days) compared to the reaction with regular mNHOs.^[^ ^21^ ^]^ This is most likely a result of the reduced nucleophilicity at carbon,^[^ ^43^ ^]^ due to the π‐accepting properties of the PR_2_‐moiety. The diazoalkene **5** shows a characteristic IR band for the C═N_2_ moiety at $\tilde{\nu }$  =  1953 cm^−1^ in a similar range as known diazoalkenes.^[^ ^21^, ^22^, ^23^ ^]^ **5** features a ^13^C NMR shift for the CN_2_ moiety at *δ*  =  34.6 ppm and a ^31^P{^1^H} shift at *δ*  =  −14.1 ppm. In the solid‐state structure the P‐moiety and the diazo fragment are both rotated away from the N‐Dipp groups (Scheme 1; inset). The structural parameters of **5** are similar to non‐chelating diazoalkenes reported previously.^[^ ^21^, ^22^, ^23^ ^]^ P‐diazoalkene **5** is the first chelating diazoalkene and is stable in the solid‐state and in solution under inert atmosphere in the dark for months. Differential scanning calorimetry (DSC) measurements indicate in the solid‐state stability up to 155 °C (Figure S105).

### Photogeneration of (Metallo)Vinylidenes

**Scheme 2:**
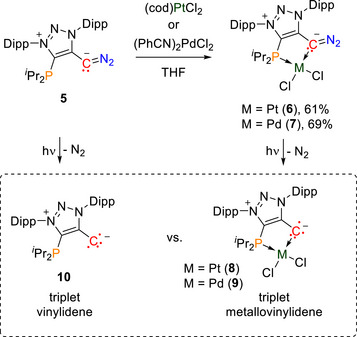
Synthesis of the Pt and Pd complexes **6** and **7** and their irradiation products at low temperature; cod  = 1,5‐cyclooctadiene.

**Figure 2:**
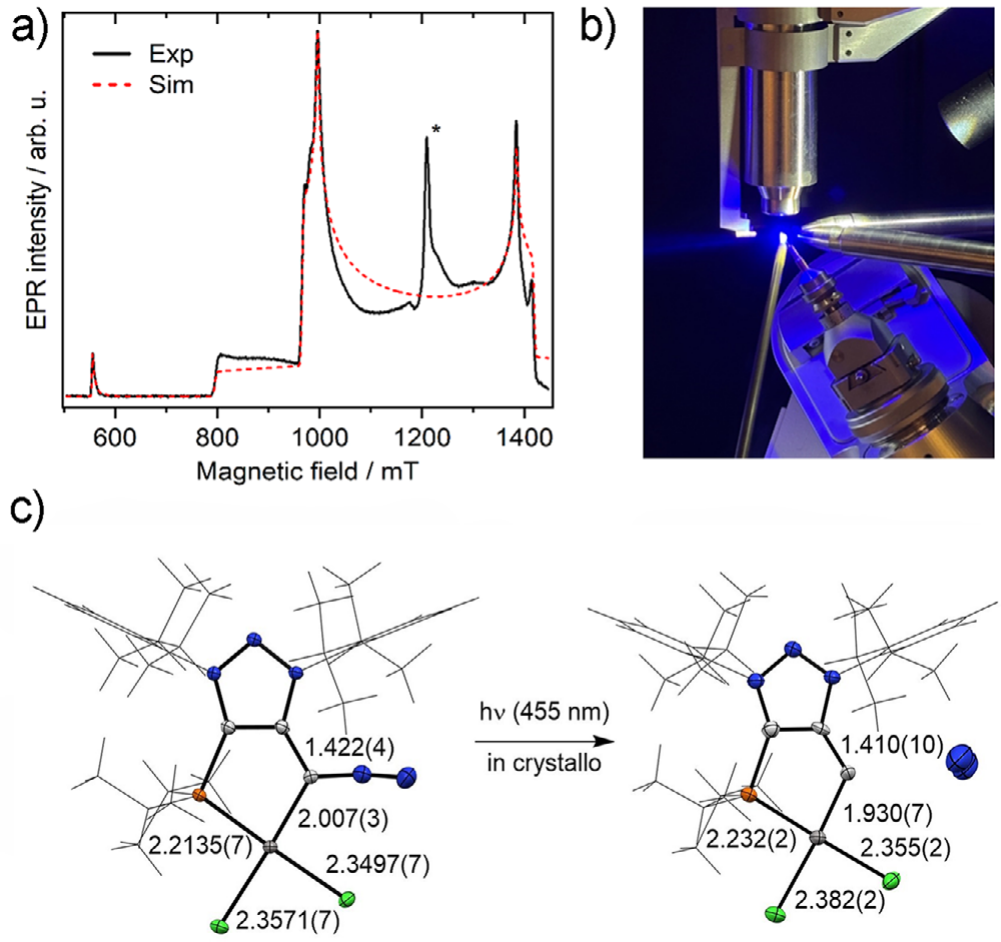
a) Q‐band EPR spectrum (black solid line) of triplet vinylidene **10** recorded at 5 K following the photolysis (at 10 K) of **5** in a frozen toluene solution (12 mM). The spectrum is overlaid with the best fit for an *S*  =  1 species (red dashed line). The narrow signal marked with an asterisk originates from photolysis byproducts (radicals and/or radical pairs); b) Irradiation setup for X‐ray crystallography. After structure determination of **6**, the *in crystallo* photoreaction to **8** is performed, while the crystal is constantly cooled at 100 K and protected in N_2_ cryostream on the goniometer. Structure determination of **8** was subsequently performed on the same crystal; c) Single‐crystal X‐ray structures with selected bond lengths in Å before and after the *in crystallo* reaction. Only one out of two molecules in the asymmetric unit of **8** is shown.

Next, we targeted the formation of the chelated metal diazoalkene complex. Addition of (cod)PtCl_2_ to diazoalkene **5** afforded the chelating P/diazoalkene platinum complex **6** (Scheme 2). Analogously, **5** reacts with [(PhCN)_2_PdCl_2_] to afford the palladium complex **7**. Upon metal coordination, the diazo moiety stays intact and its IR frequency shifts toward higher wavenumbers [$\tilde{\nu }$  =  2033 cm^−1^ (**6**); $\tilde{\nu }$  =  2034 cm^−1^ (**7**)] compared to the free diazoalkene ligand [$\tilde{\nu }$  =  1953 cm^−1^ (**5**)]. The diazo carbon atom of the chelate Pt‐diazoalkene complex **6** appears at *δ* = 25.2 ppm (d, *J_CP_*  =  6.7 Hz). The structural connectivity of both **8** and **9** (for **9**, see Supporting Information) could clearly be established by X‐ray diffraction (see below).^[^ ^44^ ^]^ With the metal complexes and free diazoalkene ligand in hand we targeted the characterization of the triplet vinylidenes. UV irradiation of the free diazoalkene chelate **5** at 10 K in frozen toluene inside an EPR cavity resulted in a characteristic triplet EPR spectrum assigned to **10** (Figure 2a). Spectral fitting of the EPR data yielded the ZFS parameters *D* = 0.388 cm^−1^ and |*E|*/*D* = 0.026 (closely matching the computational values for **10**: *D* = 0.41 cm^−1^ and |*E*|/D = 0.01, see discussion below). These ZFS parameters are very close to those of the previously described triplet vinylidenes based on the 1,2,3‐triazole heterocycle.^[^ ^24^ ^]^ Furthermore, analysis of the orientation‐selective ^14^N ENDOR spectra (see Figure S120) revealed only an insignificant influence of the flanking phosphine moiety on the electronic structure of the triplet vinylidene **10**.^[^ ^24^, ^25^ ^]^ While phosphorus‐related signals were clearly present in the ENDOR data (nuclear spin *I*(^31^P)  =  1/2, 100% natural abundance), its hyperfine tensor could only be partially constrained due to overlapping ^14^N and ^1^H features and a low signal intensity (see Table S5). Temperature‐dependent EPR measurements (Figure S119) demonstrated low stability withthe onset of signal decay around 108 K, comparable to the previously described 1,2,3‐triazole‐based species^[^ ^25^ ^]^ (see also the “reactivity/product studies” section below).

We next switched to the metallovinylidenes. In order to obtain structural information, a single crystal of the Pt‐diazo compound **6** was irradiated for 11 min at 455 nm on the X‐ray diffractometer. The *in crystallo* reaction^[^ ^45^ ^]^ yielded a lower space group symmetry, changing from monoclinic space group *C*2/c containing one Pt‐diazo compound **6** in the asymmetric unit to monoclinic space group *Cc* with two molecules of the triplet metallovinylidene **8** as well as one (out of two) N_2_ gas molecules and one extra void space (Figure 2c). The X‐ray data clearly confirms the formation of the desired metallovinylidene, which shows a Pt─C bond shortening upon N_2_‐loss [2.007(3) Å (**6**) versus 1.930(7) Å (**8**)] and a shortening of the C─C bond [1.442(4) Å (**6**) versus 1.410(10) Å (**8**)].

### Electronic Structure Characterization

Quantum‐chemical calculations were performed with Orca^[^ ^46^ ^]^ to understand the electronic structures of the metallovinylidenes **8** and **9** in relation to the triplet vinylidene **10** and connect them to the experimental findings. Geometry optimizations were performed using the hybrid density functional PBE0^[^ ^47^ ^]^ with the ZORA Hamiltonian^[^ ^48^, ^49^, ^50^, ^51^ ^]^ and the ZORA recontracted versions of the def2 basis sets,^[^ ^52^, ^53^ ^]^ assuming both a triplet (*S*  =  1) and a closed‐shell singlet (*S*  =  0) ground state. Geometry optimization of the free vinylidene **10** is possible only in the triplet state, whereas no minimum of **10** was located on the singlet potential energy surface, as observed in triplet vinylidenes reported previously.^[^ ^25^ ^]^ The adiabatic energy differences between the singlet and triplet states for **8** and **9** were found to be 8.6 and 9.6 kcal mol^−1^, respectively, in favor of the triplet states. In the triplet ground state geometry of **8**, the coordination sphere of Pt is almost planar, consistent with the crystallographic structure. By contrast, the optimized structure of **8** in its excited singlet state is predicted to have a tetrahedrally distorted Pt center. Therefore, **10**, **8**, and **9** are all computed to have *S*  =  1 ground states, in line with experimental observations.

Single‐point calculations were performed on the triplet ground state geometries to determine the singlet–triplet vertical excitation energies. Correlated wavefunction‐based calculations employing the DLPNO‐CCSD(T) approach^[^ ^54^, ^55^, ^56^ ^]^ with a two‐point extrapolation procedure that provides benchmark‐quality spin‐state energetics,^[^ ^57^, ^58^, ^59^ ^]^ locate the vertical gaps to the closed‐shell singlet at 19.4 and 18.0 kcal mol^−1^ for **8** and **9**, respectively. Multireference calculations using the 2^nd^‐order N‐electron valence state perturbation theory (NEVPT2)^[^ ^60^, ^61^ ^]^ on the basis of complete active space self‐consistent field (CASSCF) wavefunctions with an active space of 14 electrons in 11 orbitals (including 7 orbitals of the vinylidene ligand and 4 metal d‐orbitals, see Figure S134A,B), predict energy gaps between the triplet ground states and the excited closed‐shell singlet states‐ of 21.0 and 21.9 kcal mol^−1^ (Tables S8 and S9), respectively, consistent with the coupled cluster results. However, the multireference calculations can additionally identify open–shell singlet excited states, which are located at 11.5 and 12.7 kcal mol^−1^, for **8** and **9**, respectively, above the triplet ground states. The next closed‐shell singlet states lie significantly higher, at 31.4 kcal mol^−1^ for **8** and 28.4 kcal mol^−1^ for **9**. It is noted that for the free vinylidene **10**, NEVPT2 calculations using an active space of 10 electrons in 8 orbitals (Figure S134C) suggest that the first excited state is instead the closed‐shell singlet at 10.5 kcal mol^−1^, with the open‐shell singlet at 14.4 kcal mol^−1^ (Table S10), similar to previously reported vinylidenes.^[^ ^25^ ^]^ These results suggest that coordination of the triplet vinylidene **10** in compounds **8** and **9** destabilizes the closed‐shell singlet excited states while lowering the energy of the open‐shell singlets (Figure S133).

**Figure 3:**
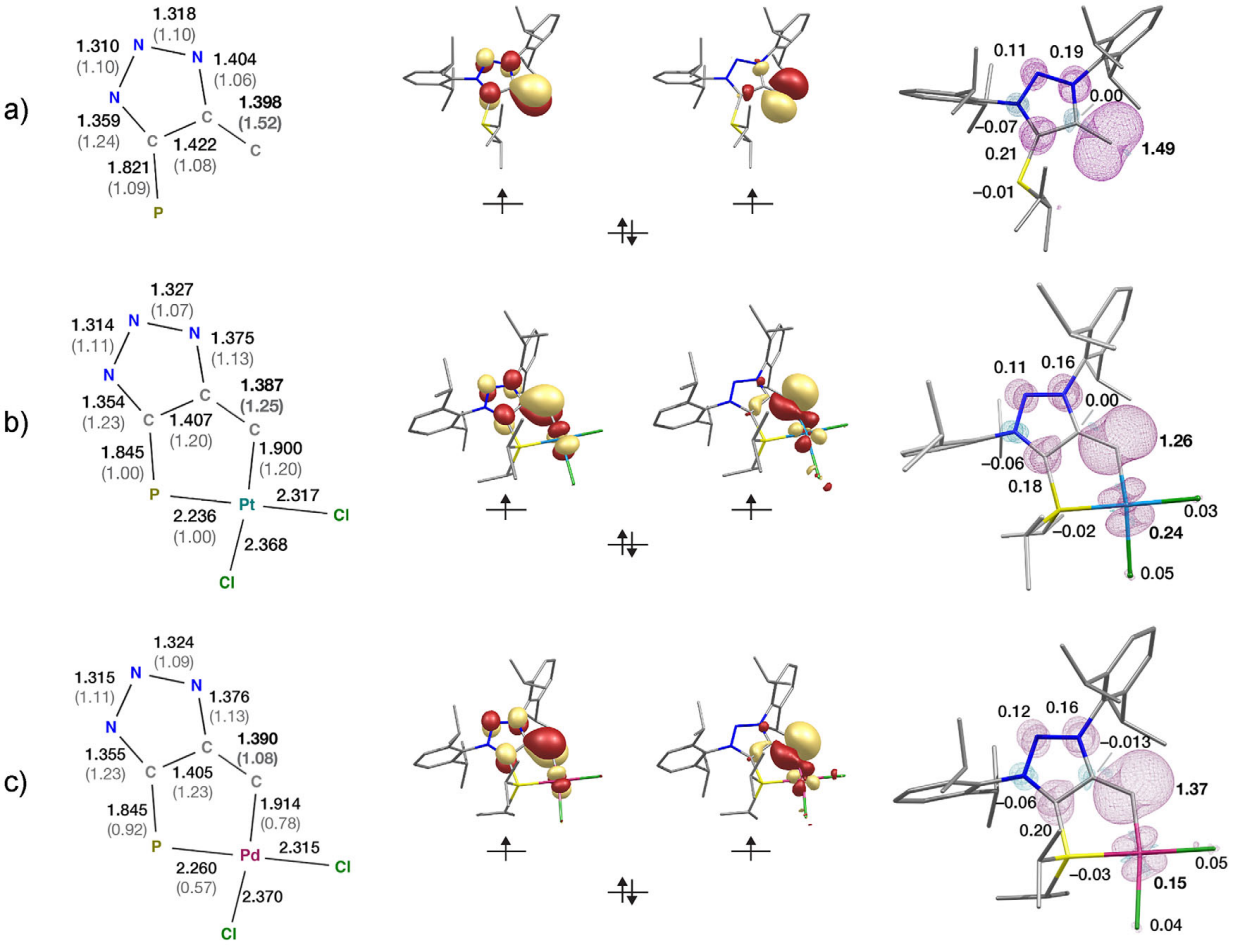
(Left) optimized bond lengths (in Å) and computed Mayer bond orders (in parentheses), (middle) quasi‐restricted singly occupied orbitals describing the valence electronic structure of the triplet states, and (right) spin density distribution and atomic spin populations for selected atoms of a) **10**, b) **8**, and c) **9**. Results are obtained from calculations using the PBE0 functional.

The bond distances, Mayer bond orders, frontier orbitals, and spin density distributions of **10**, **8**, and **9** are shown in Figure 3. It is evident that formation of **8** leaves the bond lengths, Mayer bond orders, and atomic spin populations of the triazole largely unaffected. The most noticeable difference is the decrease of the terminal C─C Mayer bond order from 1.52 in **10** to 1.25 in **8**, due to delocalization of electron density on the Pt center. Regarding changes in the spin density distribution, one of the two unpaired electrons of **10** lies in the in‐plane p‐orbital of the terminal carbon, whereas the other resides in an orbital that is shared between the terminal C and certain members of the triazole. The singly occupied orbitals of **8** are similar to those of **10**, but additionally mix with the Pt d‐orbitals. The decreased spin population on the terminal C from 1.49 in **10** to 1.26 in **8**, and spin population of 0.24 on Pt, as shown in (Figure 3, right), indicates that in **8** some of the unpaired electron density of the *π*‐framework is delocalized on Pt.

Overall, these results show that upon coordination of **10** to Pt, despite electron and spin density delocalization over the metal center, the vinylidene identity is preserved. The Pt─C bond can be understood as a dative σ bond, in which the C atom of the vinylidene donates an electron pair to an empty 5d orbital of the metal center (the corresponding Pt─C σ‐bonding and σ*‐antibonding orbitals are represented by CASSCF orbitals 2 and 11, respectively, in Figure S132). The fact that the Pt─C bond is established through donation from an inner orbital of the vinylidene, while the frontier singly occupied orbitals preserve their vinylidene character, distinguishes clearly these metallovinylidenes from both Fischer‐ and Schrock‐type carbenes. The Pt─C bond has a Mayer bond order of 1.26, indicating a higher than single‐bond character, which arises from weak back‐bonding from Pt to the singly occupied orbitals of the vinylidene (visualized in CASSCF orbitals 3, 4, 7, and 8 in Figure S132). This is confirmed by natural bond orbital (NBO) analysis, which shows that back‐donation from two 5d β orbitals of Pt to two unoccupied β orbitals localized on the vinylidene carbon, is associated with stabilizing interactions of 14.6 and 22.8 kcal mol^−1^, respectively, quantified from second‐order perturbation theory (see Figure S135). This also explains the positive (0.24) spin population on Pt. Overall, the formal oxidation state of the metal center is Pt(II), and the electronic structure of the triplet vinylidene is only weakly perturbed as a result of the coordination.

It is noted that metallovinylidenes **8** and **9** are structurally very similar (Figure S134). Minor differences such as the slight increase of the metal–C distance and the concomitant decrease of the Mayer bond order in **9**, as well as the increase of spin population on the coordinating C and the accompanying decrease on the Pd can be explained by the fact that the 5d orbitals of Pt are more diffuse than the 4d orbitals of Pd and therefore can better interact with the vinylidene orbitals through π‐backbonding. Hence, there is stronger electron and spin density delocalization from the vinylidene to the metal in the case of **8** than in **9**.

The preservation of the vinylidene identity distinguishes metallovinylidenes from the recently reported triplet metallocarbenes of the structure M─C─SiMe_3_ (M = Pt, Pd).^[^ ^15^ ^]^ The primary differences between metallovinylidenes and metallocarbenes are in their spin density distributions, the nature of their singly occupied orbitals, and the mechanisms responsible for triplet state stabilization. As suggested by Schneider, Holthausen and co‐workers^[^ ^15^ ^]^ and reproduced by our own calculations, the two unpaired electrons of the metallocarbenes reside in the p‐orbitals of C, which are perpendicular to the Pt─C─Si axis of the nearly linear Pt─C─Si system (165°). Hence, the resulting unpaired electron density is predominantly localized around C, as reflected in its spin population of 1.67, with only 0.10 on Pt and 0.13 on the SiMe_3_ moiety. By contrast, in the triplet metallovinylidene **8** the spin population on C is only 1.26 because the spin density is delocalized over the π‐system of the five‐membered ring (total spin population of ∼0.4) and on Pt with 0.24. This is consistent with the higher Mayer bond order of the Pt─C bond of 1.26 in **8** versus 0.86 in the metallocarbene (both calculated at the same level of theory with PBE0), suggesting weaker π‐backbonding from Pt to the ligating C in the case of the metallocarbene. The distinct geometries of the complexes are consistent with distinct hybridization at the C, which is also inferred from NBO analysis: in the metallocarbenes, the C is sp hybridized, whereas in the studied metallovinylidenes, with smaller C─C─M (M = Pt, Pd) angles of 122°, it can be considered sp^2^ hybridized. This is consistent with the substantially larger calculated core spin polarization on the terminal C of **8** compared to Pt─C─SiMe_3_ (Table S11), suggesting that the unpaired electrons in **8** reside in orbitals with larger contributions from the s atomic orbitals of C. Finally, we note that in the above mentioned metallocarbenes the triplet state is suggested to be stabilized through a spin‐polarized metal‐push/silyl‐pull mechanism.^[^ ^15^ ^]^ By contrast, in the vinylidenes and metallovinylidenes the dominant factor of triplet state stabilization appears to be the delocalization of one singly occupied C orbital over the π‐system of the ring. Overall, our analysis reveals that this characteristic delocalization of the spin density over the ring π‐system enables the metallovinylidenes to maintain their triplet ground states, despite bending of the C─C─M entity.

#### Mid‐infrared and THz‐EPR Spectroscopy

In a next step, the magnetic properties of the reactive triplet metallovinylidenes **8** and **9** were determined and compared to quantum chemical calculations. UV illumination of the metal diazoalkene complexes **6** and **7** (in frozen DCM and toluene, respectively) produced no discernible EPR signals that could be ascribed to **8** or **9** (Figure S118), despite a clear change in the frozen solution color. This negative result is readily explained by the fact that the ZFS parameter *D* is expected to be much larger for both metallovinylidenes than for **10**, due to delocalization of the spin density onto the metal atoms (see below for more details). Thus, EPR transitions are outside of the spectroscopic window of Q‐band EPR spectroscopy but should be visible in the THz range.

**Figure 4:**
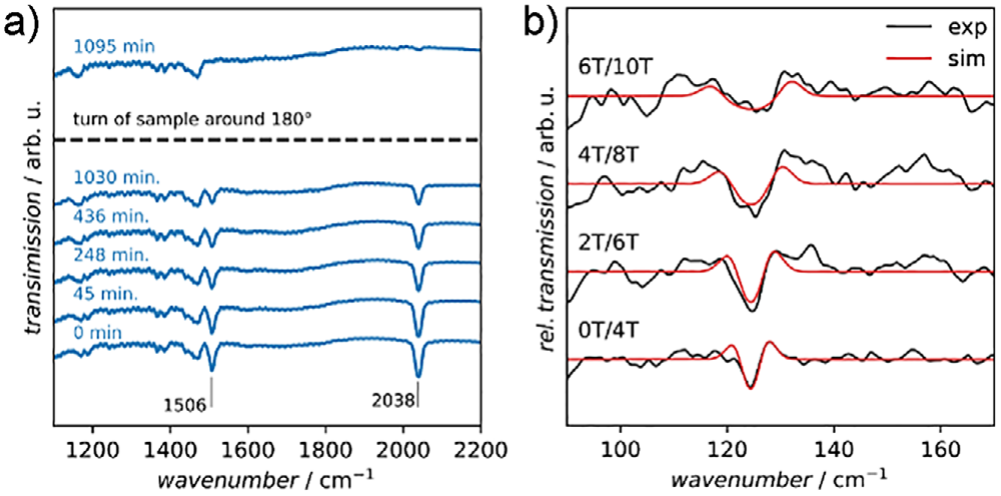
a) Transmission IR spectra of the diazoalkane precursor **6** during irradiation at 395 nm at a temperature of 10 K. b) Magnetic‐field‐division FD‐FT THz‐EPR spectra of the triplet **8** after completed conversion and stopped illumination at a temperature of 5 K.

Prior to performing THz‐EPR measurements on **8**, we first studied the conversion of the diazo precursor **6** to Pt‐vinylidene **8** by infrared (IR) spectroscopy (see Supporting Information for details). This allowed a direct monitoring of the photoreaction via the characteristic diazo stretch vibration band at 2038 cm^−1^ under similar conditions as in the targeted THz‐EPR experiments. As shown in Figure 4a, upon illumination at a temperature of 10 K, the intensity of the vibration band decreased due to N_2_ loss. Quantitative disappearance was achieved by illuminating the sample pellet from both sides, suggesting complete conversion.

Subsequently, FD‐FT THz‐EPR measurements^[^ ^62^ ^]^ were performed following illumination of a freshly prepared precursor sample (see Supporting Information for details). The magnetic‐field‐division spectra (MDS), depicted in Figure 4b, clearly show a magnetic‐field‐dependent signal around 125 cm^−1^. The observed “up‐down‐up” pattern is indicative of a triplet spin state with a large axial ZFS and low rhombicity (*E*/*D* < 0.01). The signals could be simulated^[^ ^62^, ^63^ ^]^ with a triplet spin Hamiltonian (see Supporting Information for details) using the optimal parameters *g* = 2.0(2), *D *= 124.5(5) cm^−1^, *E*/*D* = 0 and a Gaussian linewidth of 4.0 cm^−1^. Attempts to measure the ZFS for the triplet metallovinylidene based on Pd (**9**) by FD‐FT THz‐EPR at lower transition energies down to 3 cm^−1^ were not successful.

### SQUID Measurements

**Figure 5:**
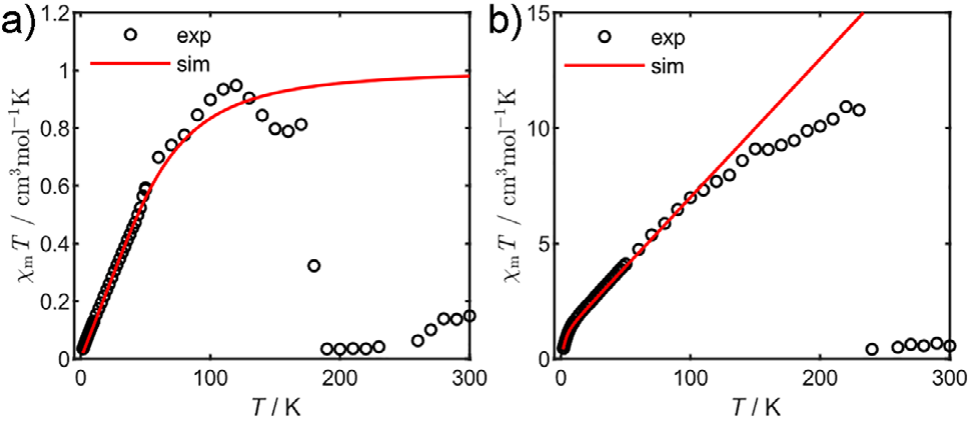
χ_m_ *T* over *T* plot from 1.8–300 K of **8** a) and **9** b) formed in the SQUID magnetometer by illumination with 390 nm at 10 K.

SQUID measurements were performed to gain insight into the magnetic properties as well as the stability of **8** and **9**. For reference, the diamagnetic precursors **6** and **7** were measured prior to illumination with a 395 nm LED that was coupled via a fiber to the sample in the magnetometer. The samples were kept at 10 K during illumination and the photoreaction was followed continuously (see Supporting Information). After illumination, magnetisation measurements at low temperatures and a susceptibility measurement between 1.8–300 K were performed.^[^ ^64^ ^]^ The subtraction of the magnetic moments of the diamagnetic precursors allowed extracting the contribution of the species formed by illumination. The resulting χ_m_ *T* over *T* plots for **8** (Figure 5a) and **9** (Figure 5b) show that a paramagnetic species was formed in both experiments. The data for **8** shows a linear increase for χ_m_ *T* from essentially zero to around 1 cm^3^mol^−1^K^−1^ at 100 K before it drops to 0 cm^3^mol^−1^K^−1^ at 180 K. For **9**, the increase in χ_m_ *T* is much stronger with an additional curvature at low temperature. For this compound, χ_m_ *T* reaches 11 cm^3^mol^−1^K^−1^ at 230 K before dropping to 0 cm^3^mol^−1^K^−1^. In both samples, the original susceptibility values could not be recovered by cooling the sample again (see Supporting Information).

The data for **8** was modelled using the spin Hamiltonian assuming a *S *= 1 species with *g *= 2 resulting in a ZFS of *D *= 120(5) cm^−1^ and *E/D *= 0.0(1).^[^ ^65^ ^]^ The drop at 180 K is not considered in the simulation and is attributed to thermal decomposition of **8**. The data of **9** was modelled similarly. In this case the strong increase in χ_m_ *T* above 10 K is attributed to a strong temperature‐independent paramagnetism (TIP) of 0.060(1) cm^3^mol^−1^ and a much smaller ZFS of *D* = 8.0(5) cm^−1^ and *E/D *= 0.1(1). This becomes more evident by considering the magnetisation at low temperatures (see Supporting Information).

**Figure 6:**
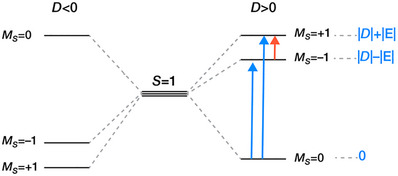
Representation of the zero‐field splitting for a *S*  =  1 system for *D* < 0 (left) and *D* > 0 (right). The transitions between the *M_S_*  =  0 and *M_S_*  =  ±1 states are marked in blue, while the transitions between the *M_S_*  =  +1 and *M_S_*  =  −1 states in red. Relative energies of states for the *D* > 0 case are shown in blue.

The much lower ZFS in the Pd compound is expected due to the weaker spin‐orbit coupling (SOC) with respect to the heavier Pt atom. The ZFS of triplet metallovinylidene **8**, derived from multireference perturbation theory calculations with CASSCF/NEVPT2 with an active space of 14 electrons in 11 orbitals averaging over 22 triplet and 22 singlet states, is *D* = 124.1 cm^−1^ with |*E*|/*D* = 0.04. It arises mainly from the coupling of the triplet ground state with singlet and triplet excited states through SOC, whereas the contribution of spin–spin coupling (SSC) is negligible (less than 1 cm^−1^). The excited states that contribute predominantly to the ZFS involve excitations localized in the vinylidene ligand and excitations from metal‐centered orbitals to vinylidene orbitals (Tables S12 and S13). The ZFS of **9**, calculated by averaging over 35 triplet and 35 singlet states, is *D* = 12.1 cm^−1^ with |*E*|/*D* = 0.27. The calculated ZFS parameter of both samples agree very well with the experimental data. ZFS of a system with *S* = 1 describes the splitting of the spin multiplet into three sublevels with *M_s_* = +1, −1, and 0, in the absence of magnetic field and it is described by the parameters *D* and *E*. The sign of *D* determines the type of magnetic anisotropy associated with the multiplet (Figure 6). If *D* > 0, then the *M_s_* = 0 state will have the lowest energy, whereas if *D* < 0, the *M_s_* = 0 state will be higher than the *M_s_* = +1 and *M_s_* = −1 states. The splitting between the *M_s_* = 0 and *M_s_* = ± 1 states is |*D*|± |*E*| and the splitting between the *M_s_* = +1 and *M_s_* = −1 states is 2|*E*|. EPR signals could arise either from transitions between the *M_s_* = 0 state and the *M_s_* = ± 1 states (blue arrows in Figure 6) or transitions between the *M_s_* = +1 and *M_s_* = −1 states (red arrow).

For **8** and **9**, the energy of transitions from the *M_s_*  =  0 state to the *M_s_*  =  ± 1 exceeds those that can be observed in typical EPR experiments. Therefore, the absence of Q‐band EPR signals for **8** and **9** is attributed to a very large axial ZFS arising from SOC effects of Pt and Pd, respectively. However, at higher frequencies in the THz frequency range the direct EPR detection should be possible,^[^ ^66^ ^]^ which indeed was confirmed for the Pt‐complex **8**.

### Reactivity/Product Studies

**Scheme 3:**
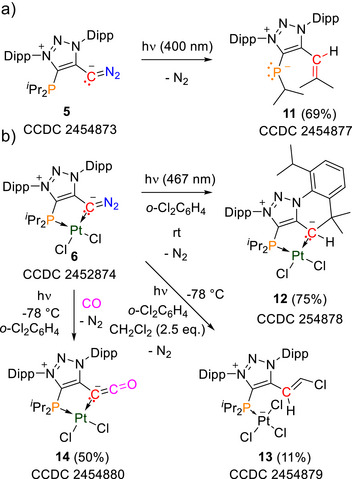
Reactivity of the free vinylidene **10** and the Pt‐metallovinylidene **8** derived from the diazo precursors **5** and **6**.

Finally, we studied the reactivity of the triplet vinylidene as well as the Pt‐metallovinylidene (Scheme 3). Irradiation of the free chelating diazoalkene **5** in toluene at room temperature afforded cleanly the unknown mesoionic phosphinidene **11** [δ(^31^P)  =  −12.5 ppm] in which one isopropyl group was activated and migrated to the vinylidene carbon.^[^ ^67^ ^]^ The structure could be clearly verified by X‐ray diffraction.^[^ ^44^ ^]^ In contrast, irradiation of the Pt complex **6** in *ortho*‐dichlorobenzene (*o*‐DCB) at room temperature afforded the insertion product **12** in which C─H insertion into the Dipp moiety occurred. C─H insertion into the C(sp^3^)−H could be observed previously for triplet vinylidenes,^[^ ^25^ ^]^ which was also observed for metallocarbenes.^[^ ^15^, ^18^ ^]^ Besides the insertion into the Dipp C─H bond, we could also detect another compound [δ(^31^P in CD_2_Cl_2_)  =  45.0 ppm] which we believe to arise from C─H insertion of the ─P(^i^Pr)_2_ fragment. However, the species proved challenging to crystallize in order to ambiguously verify its structure by X‐ray diffraction.

While **6** is stable in CH_2_Cl_2_, interestingly, irradiation (467 nm) in the presence of CH_2_Cl_2_ (2.5 eq.) leads to a minor side product **13** (11% yield) besides the C─H insertion products, which was structurally verified by X‐ray diffraction. In **13** CH_2_Cl_2_ was activated to form a chloroalkene while the second chloride migrated to platinum to form a platinate anion.

**Figure 7:**
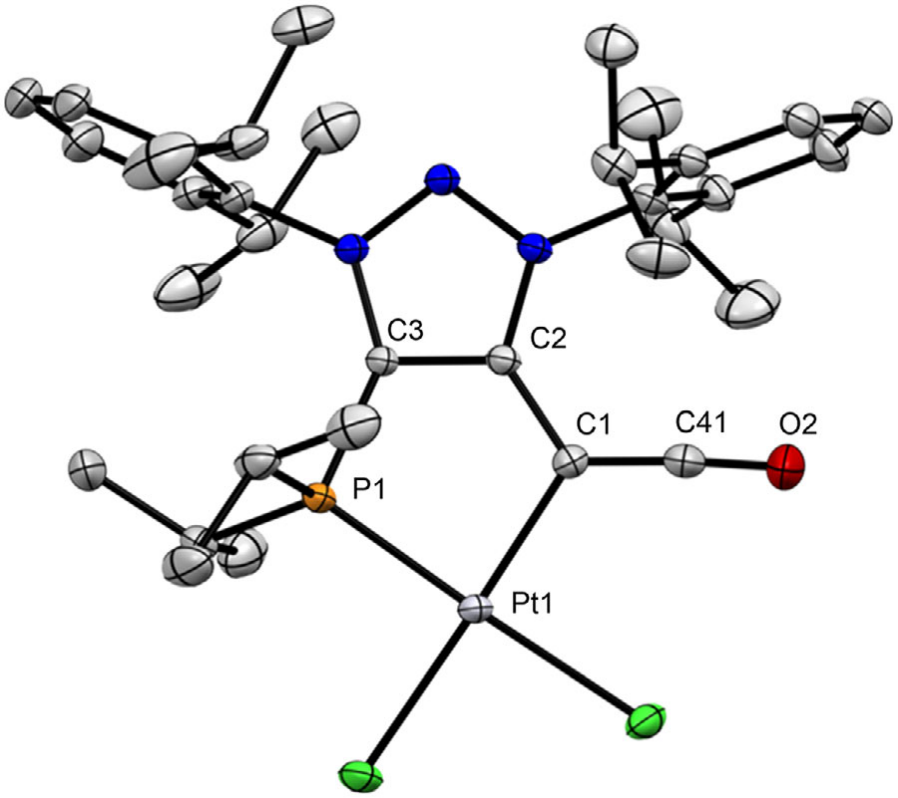
X‐ray solid‐state structure of Pt‐ketenyl complex **14**. Selected bond parameters in [Å] and [°]:C1–C2 1.433(5), C1–Pt1 2.044(2), C1–C41 1.313(5), C41–O2 1.167(5) P1–Pt1 2.207(1), C2–C3 1.389(4), C3–P1 1.826(2), C2–C1–Pt1 114.7(2), C1–Pt1–P1 87.09(8), C2–C1–C41 123.2(2), and Pt1–C1–C41 122.1(2). C1–C41–O2 176.1(4).

Finally, we investigated the reactivity of the Pt‐metallovinylidene in the presence of carbon monoxide. No reaction occurred between **6** and CO gas at room temperature. Note, free diazoalkenes are known to thermally react with CO to form vinylidene ketenes.^[^ ^21^, ^68^ ^]^ However, irradiation (467 nm) of **6** in a frozen *o*‐DCB solution at −78 °C in the presence of CO afforded the ketenyl complex **14**, in a mixture of the C─H insertion products. The connectivity could be structurally verified by X‐ray diffraction (Figure 7). Interestingly, in this case we observed a temperature effect: irradiation at room temperature in the presence of CO only afforded the C─H insertion products. A reason could be that low temperatures do not allow conformational freedom to rotate/align the fragments required for C─H insertion opening up intermolecular reactivity of the triplet metallovinylidene.

## Conclusions

In summary, we present the synthesis and characterization of the first chelating diazoalkene ligand and its metal coordination complexes. Photoexcitation of the free diazoalkene ligand and the metal complexes allowed the systematic investigation of the free triplet vinylidene and triplet metallovinylidenes by Q‐band EPR/ENDOR and THz‐EPR spectroscopy, respectively. The results were supported by *in crystallo* experiments using X‐ray diffraction as well as reactivity studies. While the free vinylidene in its triplet ground state shows no interaction with the PR_2_ moiety in close proximity to the vinylidene center, the ligand allows to geometrically constrain the triplet metallovinylidenes into a rigid five‐membered ring. The determined large axial ZFS of *D*  =  124.5(5) cm^−1^ (Pt) by THz‐EPR spectroscopy agrees within the error limits with the value determined by SQUID magnetometry [*D * =  120(5) cm^−1^ for Pt and *D*  =  8.0(5) cm^−1^ for Pd] in perfect agreement with quantum chemical calculations. The presented findings open up an exciting field of new diradical compounds: triplet metallovinylidenes. In contrast to the very recently described first triplet metallocarbenes, triplet metallovinylidenes feature spin density, which is, similar to the free vinylidenes, delocalized over the π‐system shortening the C─C bond. In theory this concept could be transferred to other elements, while the new chelating diazoalkene ligand class should allow exploration of the coordination chemistry of diazoalkenes across the elements of the periodic table.

## Conflict of Interests

The authors declare no conflict of interest.

## Supporting information



Supporting Information

Supporting Information

## Data Availability

The data that support the findings of this study are available in the Supporting Information material of this article.
