# Supplementary material for: Triplet Metallovinylidenes of Palladium and Platinum Based on a Chelating P/Diazoalkene Ligand
Source: Angew Chem Int Ed Engl. 2025 Nov 10;65(1):e16032. doi: 10.1002/anie.202516032 (PMC12759197; doi:10.1002/anie.202516032)
Supplement: Supplementary file 1 — Supporting Information [file ANIE-65-e16032-s001.pdf]

Supplementary Materials for

Triplet Metallovinylidenes of Palladium and Platinum based on a Chelating P/Diazoalkene Ligand

M. Amann, M. Drosou, T. Al Said, A. Allgaier, Y. Kutin, P. W. Antoni, J. J. Holstein, M. Kasanmascheff, J. van Slageren, A. Schnegg, D. Pantazis, M. M. Hansmann

Table of Contents

1. Materials and Methods 2
2. Characterization data 3
3. NMR spectra 14
4. IR Spectra 55
5. UV-VIS 61
5.1 UV-VIS spectra 61
5.2 VT UV-VIS 62
6. SDT measurement 65
7. TGA measurement 66
8. X-ray characterization data 67
9. Q-Band (34 GHz) and X-band (9.5 GHz) EPR 91
9.1 EPR sample preparation 91
9.2 EPR experimental details 91
10. THz-EPR and IR spectroscopy 95
11. SQUID Magnetometry 99
13. Literature 117

1. Materials and Methods

All solvents were purified by distillation over the drying agents indicated, or stored over molecular sieves and degassed with argon. Reactions were carried out either under N_2 or Ar atmosphere. Solids were handled and NMR samples were prepared in a nitrogen filled glovebox. High resolution MS (EI): Finnigan MAT 8200 (70 eV), ESIMS: Finnigan MAT 95, accurate mass determinations: Bruker APEX III FT-MS (7 T magnet) and LTQ-Orbitrap-XL (Thermo Scientific) equipped with a heated electrospray ionization source (HESI). NMR: NMR spectra were measured on the spectrometers Bruker AV 500 Avance NEO, Bruker AV 600 Avance NEO, Bruker AV 400 Avance III HD NanoBay, and AV 600 Avance III HD and chemical shifts (δ) are referenced to their solvent signals [C_6D_6, 7.16 (^{1}H-NMR) 128.06 (^{13}C-NMR); CD_3CN, 1.94 (^{1}H-NMR) 118.26 (^{13}C-NMR), $CDCl_3$ 7.26 (^{1}H-NMR) 77.16 (^{13}C-NMR), CD_2Cl_2, 5.32 (^{1}H-NMR) 54.00(^{13}C-NMR)],coupling constants (J) in Hz. All spectra were recorded in 5 mm NMR tubes at the temperatures indicated. The solvent signals were used as references and the chemical shifts converted to the TMS scale. UV-Vis spectra were recorded on an Agilent Cary60. All commercially available compounds (Acros, ABCR, Merck, Fisher Scientific) were used as received unless otherwise stated. IR-ATR measurements (diamond) were performed in reflection mode on a Bruker Alpha II inside a glovebox, wavenumbers in cm^{-1}. Melting points were measured with a Büchi M-560 apparatus. TGA measurements were recorded on a TA Instruments – Discovery SDT 650 apparatus and SDT measurements on a TA Instruments – DSC 25 apparatus.

For irradiation experiments either a *Kessil PR160L* (at given wavelength) lamp or a *THORLABS DC2200 1-Channel LED Driver* coupled with a *THORLABS M455F3 fiber-coupled LED* (at given wavelength) and a *THORLABS M59L02 SMA-SMA Fiber Patch Cable* was used.

Dipp-Triazene was prepared as described in the literature.[1] For diazotization N_2O was directly used from the gas bottle (quality 5.0 obtained from Messer).

2. Characterization data

Synthesis of 1

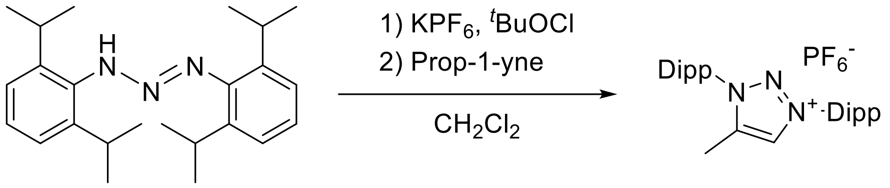

1 was synthesized after a modified literature procedure.[2] To a mixture of Dipp-triazene (1.5 g, 4.1 mmol, 1.0 eq.) and KPF_6 (1.04 g, 5.1 mmol, 1.2 eq.) in CH_2Cl_2 (30 mL) *tert*-butyl-hypochlorite (480 µL, 4.2 mmol, 1.0 eq.) was added in the dark at -78 °C. The reaction mixture was stirred in the dark at -78 °C for 1 hour and then at this temperature a solution of 5% prop-1-yne (10 mL, 10 mmol, 2.4 eq.) in THF was added. The reaction was warmed up to room temperature overnight, resulting in a dark red reaction mixture. The reaction was filtered and the remaining solid washed with CH_2Cl_2 (30 mL). The collected filtrates were evaporated to complete dryness, dissolved in the minimum volume of CH_2Cl_2 (8 mL) and the crude product precipitated by addition of Et_2O (250 mL) under vigorous stirring. The solid was collected by filtration and washed with Et_2O (50 mL), this procedure was repeated once more. After filtration, the product was dried under reduced pressure to give the desired product **1** as a colorless solid (882 mg, 1.60 mmol, 39%). The spectral data is in agreement with previous reports.[2]

m.p.: 217 °C

^{1}H NMR (400 MHz, $CDCl_3$, 298 K): δ [ppm] = 8.87-8.84 (m, 1H, triaz.-C*H*), 7.71 (t, J = 7.9 Hz, 1H, Ar-*H*), 7.65 (t, J = 7.8 Hz, 1H, Ar-*H*), 7.45 (d, J = 7.9 Hz, 2H, Ar-*H*), 7.39 (d, J = 7.9 Hz, 2H, Ar-*H*), 2.50 (s, 3H, C*H*$_3$), 2.30-2.22 (m, 2H, C*H*$(CH_3)_2$), 2.18-2.11 (m, 2H, C*H*$(CH_3)_2$), 1.30 (d, J = 7.0 Hz, 12H, $CH(CH_3)_2$), 1.15 (dd, J_1 = 6.9 Hz, J_2 = 2.7 Hz, 12H, $CH(CH_3)_2$).

^{13}C {^{1}H} NMR (127 MHz, $CDCl_3$; 298 K): δ [ppm] = 145.5 (2C, Ar-C_Q), 145.3 (2C, Ar-C_Q), 144.2 (1C, triaz.C_Q-CH_3), 133.6 (2C, Ar-H), 133.3 (2C, Ar-H), 133.0 (1C, triaz.*C*-H), 130.6 (1C, Ar-C_Q), 128.0 (1C, Ar-C_Q), 125.4 (2C, Ar-H), 124.9 (2C, Ar-H) 29.4 (2C, *C*H$(CH_3)_2$), 29.2 (2C, *C*H$(CH_3)_2$), 25.1 (2C, $CH(CH_3)_2$), 24.8 (2C, $CH(CH_3)_2$), 23.6 (2C, $CH(CH_3)_2$), 23.1 (2C, $CH(CH_3)_2$), 9.6 (1C, CH_3).

IR (ATR) [cm^{-1}]: ṽ = 3170, 2965, 2932, 2872, 1388, 1367, 836, 775, 764, 751, 687, 557.

HR-MS-ESI(+) calc. [M$^+$]: 404.3060; found: 404.3061.

Synthesis of 2

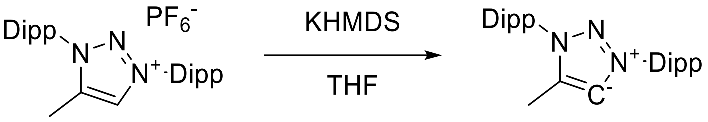

To solid triazolium salt **1** (400 mg, 0.73 mmol, 1.0 eq.) and solid KHMDS (160 mg, 0.8 mmol, 1.1 eq.) cooled to -78 °C was added THF (30 mL). The reaction turns immediately salmon colored. The reaction was stirred for 15 min at -78 °C, warmed up to room temperature for 30 min and stirred for an additional 1 h. The color changed to red while stirring at room temperature. The solvent was removed under reduced pressure. Pentane (20 mL) was added and subsequently evaporated. The remaining solid was extracted with pentane (2x20 mL). The solvent of the red colored solution was removed under reduced pressure, to afford the product **2** as an off-white solid (277 mg, 0.68 mmol, 94%).

m.p. 154 °C

^{1}H NMR (400 MHz, C_6D_6, 298 K): δ [ppm] = 7.32 (m, 1H, Ar-*H*), 7.21-7.17 (m, 3H, Ar-*H*), 7.03 (d, *J* = 7.8 Hz, 2H, Ar-*H*), 3.03 (hept, *J* = 6.9 Hz, 2H, C*H*(CH_3)$_2$), 2.48 (hept, *J* = 6.9 Hz, 2H, C*H*(CH_3)$_2$), 2.28 (s, 3H, C*H*$_3$), 1.31 (d, *J* = 6.8 Hz, 6H, CH(C*H*$_3$)$_2$), 1.21 (d, *J* = 6.9 Hz, 6H, CH(C*H*$_3$)$_2$), 1.09 (d, *J* = 6.9 Hz, 6H, CH(C*H*$_3$)$_2$), 1.06 (d, *J* = 6.9 Hz, 6H, CH(C*H*$_3$)$_2$).

^{13}C {^{1}H} NMR (100 MHz, C_6D_6; 298 K): δ [ppm] = 201.3 (1C, $C_{carbene}$), 145.7 (1C, Ar-C_Q), 145.7 (1C, triaz-C_Q), 145.5 (1C, Ar-C_Q), 139.7 (2C, Ar-C_Q), 132.4 (2C, Ar-C_Q), 131.0 (1C, Ar-H), 129.6 (1C, Ar-H), 124.3 (2C, Ar-H), 123.7 (2C, Ar-H), 28.9 (2C, CH(*C*H$_3$)$_2$), 28.9 (2C, CH(*C*H$_3$)$_2$), 25.2 (2C, CH(*C*H$_3$)$_2$), 24.6 (2C, *C*H(CH_3)$_2$), 24.2 (2C, *C*H(CH_3)$_2$), 23.0 (2C, CH(*C*H$_3$)$_2$), 12.6 (1C, *C*H$_3$).

IR (ATR) [cm^{-1}]: ṽ = 2961, 2929, 2868, 1679, 1456, 1385, 1302, 1135, 804, 781, 764, 753.

HR-MS-ESI(+) calc. [M+H$^+$]: 404.3060; found: 404.3062.

Synthesis of 3

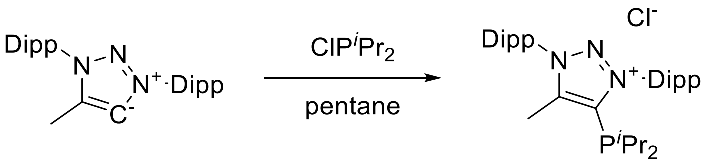

Carbene **2** (140 mg, 0.35 mmol, 1.0 eq.) in pentane (40 mL) at room temperature was added dropwise over 2 hours to ClP^{i}Pr$_2$ (250 µL, 1.56 mmol, 4.5 eq.) in pentane (40 mL) at -40 °C, leading to the precipitation of a colorless solid. During the end of the addition, the color of the reaction

changes to off-white-green. The reaction was warmed to room temperature and stirred for additional 3 hours, which results in a colorless suspension. The solid was filtered and washed with pentane (3x20 mL) and the remaining solvent was removed under reduced pressure to afford **3** as a colorless solid (178 mg, 0.32 mmol, 92%).

Note: High rates of carbene addition result in deprotonation of the product **3** to afford **1** and **4** which is visible by a color change of the solution to yellow/orange.

m.p. 252 °C

^{1}H NMR (500 MHz, CD_3CN, 298 K): δ [ppm] = 7.79 (t, J = 7.9 Hz, 1H, Ar-*H*), 7.75 (t, J = 7.9 Hz, 1H, Ar-*H*), 7.58 (d, J = 7.9 Hz, 2H, Ar-*H*), 7.53 (d, J = 7.9 Hz, 2H, Ar-*H*), 2.54 (dhept.,J_1 = 4.7 Hz, J_2 =7.1 Hz, 2H, P((C*H*(CH$_3$)$_2$)$_2$), 2.52 (s, 3H, C*H*$_3$), 2.14 (hept, J = 6.9 Hz, C*H*(CH$_3$)$_2$), 2.09 (hept, J = 6.9 Hz, C*H*(CH$_3$)$_2$), 1.35 (d, J = 6.7 Hz, 6H, CH(C*H*$_3$)$_2$), 1.27 (d, J = 6.7 Hz, 6H, CH(C*H*$_3$)$_2$), 1.20 (dd, J_1 = 15.2 Hz, J_2 = 6.4 Hz, 6H, P(C*H*(CH$_3$)$_2$) ,1.17 (d, J = 6.6 Hz, 6H, CH(C*H*$_3$)$_2$), 1.09 (dd J_1 = 15.0 Hz, J_2 = 7.2 Hz, 6H, P(C*H*(CH$_3$)$_2$), 1.09 (d, J = 6.8 Hz, 6H, CH(C*H*$_3$)$_2$).

^{13}C {^{1}H} NMR (127 MHz, CD_3CN; 298 K): δ [ppm] = 148.0 (d, J = 9.9 Hz, 1C, triaz-*C*$_Q$-CH$_3$), 146.5 (2C, Ar-*C*$_Q$),146.4 (d, J = 1.6 Hz, 2C, Ar-*C*$_Q$), 144.6 (d, J = 50,7 Hz, 1C, triaz-*C*$_Q$-P), 134.7 (1C, Ar-H), 134.5 (1C, Ar-H),131.0 (1C, Ar-*C*$_Q$), 128.7 (1C, Ar-*C*$_Q$), 126.7 (2C, Ar-H), 126.0 (2C, Ar-H), 30.6 (d, J = 1.6 Hz, 2C, (*C*H(CH$_3$)$_2$), 30.2 (2C, (*C*H(CH$_3$)$_2$), 26.7 (2C, CH(*C*H$_3$)$_2$), 25.1 (2C, CH(*C*H$_3$)$_2$), 24.1 (d, J = 11.7 Hz, 2C, P(*C*H(CH$_3$)$_2$)), 23.5 (2C, *C*H(CH$_3$)$_2$), 22.3 (d, J = 1.7 Hz, 2C, CH(*C*H$_3$)$_2$, 21.3 (d, J = 13.8 Hz, 2C, P(CH(*C*H$_3$)$_2$), 20.8 (d, J = 13.8 Hz, 2C, P(CH(*C*H$_3$)$_2$), 12.0 (1C, *C*H$_3$).

^{31}P {^{1}H} NMR (200 MHz, CD_3CN; 298 K): δ [ppm] = -7.8 (s).

^{31}P NMR (162 MHz, CD_3CN; 298 K): δ [ppm] = -7.5-(-7.8) (m).

IR (ATR) [cm^{-1}]: ṽ =2964, 2927, 2868, 1386, 1364, 1329, 1183, 1042, 800.

HR-MS-ESI(+) calc. [M$^+$-Cl$^-$]: 520.3815; found: 520.3808.

Synthesis of 4

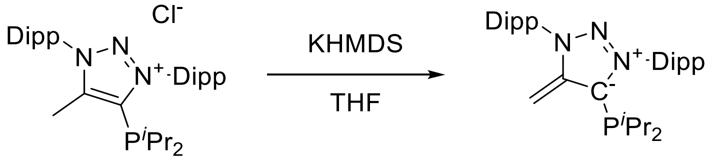

To solid triazolium salt **3** (100 mg, 180 μmol, 1.0 eq.) and solid KHMDS (40 mg, 200 μmol, 1.0 eq.) cooled to -78 °C was added THF (20 mL). The reaction turns dark red immediately. The reaction was stirred for 15 min at -78 °C, warmed up to room temperature for 30 min and stirred for 1 h. The

solvent was removed under reduced pressure. Et_2O (20 mL) was added and subsequently removed under reduced pressure. The remaining solid was extracted twice with Et_2O (20 mL each). The solvent was removed under reduced pressure to afford **4** as red solid (89 mg, 171 μmol, 95 %).

m.p. 170 °C

^{1}H NMR (500 MHz, C_6D_6, 298 K): δ [ppm] = 7.28 (dd, J_1 = 8.5 Hz, J_2 = 6.8 Hz, 1H, Ar-*H*), 7.25-7.18 (m, 3H, Ar-*H*), 7.08 (d, J = 7.7 Hz, 2H, Ar-*H*), 3.42 (hept, J = 6.9 Hz, 2H, C*H*(CH_3)$_2$), 3.19 (hept, J = 6.8 Hz, 2H, C*H*(CH_3)$_2$), 3.10 (d, J = 1.6 Hz, 1H, C*H*$_2$), 2.66 (d, J = 1.6 Hz, 1H, C*H*$_2$), 2.62 (dhept., J_1 = 6.9 Hz, J_2 = 7.1 Hz, 2H, P(C*H*(CH_3)$_2$)), 1.47 (d, J = 6.9 Hz, 6H, CH(C*H*$_3$)$_2$), 1.36 (dd, J_1 = 12.2 Hz, J_2 = 7.1 Hz, 6H, CH(C*H*$_3$)$_2$), 1.35 (d, J = 6.8 Hz, 6H, CH(C*H*$_3$)$_2$), 1.25 (d, J = 7.0 Hz, 6H, CH(C*H*$_3$)$_2$),1.24 (d, J = 6.8 Hz, 6H, CH(C*H*$_3$)$_2$), 1.08 (dd, J_1 = 17.2 Hz, J_2 = 7.0 Hz, 6H, P(CH(C*H*$_3$)$_2$)).

^{13}C {^{1}H} NMR (127 MHz, C_6D_6; 298 K): δ [ppm] = 151.0 (d, J = 7.7 Hz, 1C, triaz.-*C*$_Q$-P), 148.6 (2C, Ar-*C*$_Q$), 146.2 (d, J = 1.8 Hz, 2C, Ar-*C*$_Q$), 133.7 (1C, Ar-*C*$_Q$), 132.7 (1C, Ar-*C*$_Q$), 131.0 (1C, Ar-H), 130.4 (1C, Ar-H), 124.8 (2C, Ar-H), 124.0, (2C, Ar-H), 116.8 (d, J = 19.9 Hz, 1C, triaz.-*C*$_Q$-CH$_2$), 47.6 (1C, *C*H$_2$), 29.1 (2C, *C*H(CH_3)$_2$), 29.0 (2C, *C*H(CH_3)$_2$), 27.3 (2C, CH(*C*H$_3$)$_2$), 24.4 (2C, CH(*C*H$_3$)$_2$), 24.3 (d, J = 7.7 Hz, 2C, P(*C*H(CH_3)$_2$)), 23.9 (2C, CH(*C*H$_3$)$_2$), 23.1 (2C, CH(*C*H$_3$)$_2$), 22.4 (d, J = 26.7 Hz, 2C, P(CH(*C*H$_3$)$_2$), 20.8 (d, J = 8.6 Hz, 2C, P(CH(*C*H$_3$)$_2$)).

^{31}P {^{1}H} NMR (200 MHz, C_6D_6; 298 K): δ [ppm] = -16.2 (s).

^{31}P NMR (243 MHz, C_6D_6; 298 K): δ [ppm] = -16.2 (dddt, J_1 = 29.3 Hz, J_2 = 18.0 Hz, J_3 = 11.8 Hz, J_4 = 5.8 Hz).

IR (ATR) [cm^{-1}]: ṽ =2962, 2928, 2866, 1579, 1465, 1351, 1156, 1106, 882, 810.

HR-MS-ESI(+) calc. [M+H$^+$]: 520.3815; found: 520.3812.

Synthesis of 5

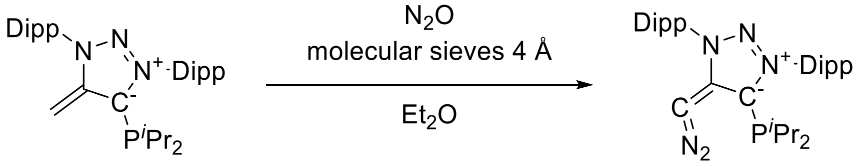

Under an atmosphere of nitrogen, P-mNHO **4** (150 mg, 0.29 mmol, 1.0 eq.) was dissolved in Et_2O (15 mL) and 4 Å molecular sieves (ca. 750 mg) were added. The solution was cooled to -78 °C and the inert gas was exchanged for 2 atm. N_2O, by evacuating the pressure Schlenk flask until gas evolution from the solution at -78 °C ceased and repressuring it with N_2O (2 bar). The flask was

closed and warmed up to room temperature (**Caution**: the actual pressure in the flask at room temperature is higher approximately 3-5 bar; use pressure glassware and an explosion shield!). Stirring the solution for 36 h in the dark led to a change of color from red to orange. The solvent was evaporated and THF (20 mL) was added. The solution was separated from the molecular sieves by filtration. Drying under reduced pressure afforded the desired diazoalkene **5** as an orange solid (146 mg, 0.27 mmol, 93%).

m.p. 132 °C (decomposition)

^{1}H NMR (600 MHz, C_6D_6, 298 K): δ [ppm] = 7.31 (t, *J* =7.8 Hz, 1H, Ar-*H*), 7.18 (d, *J* =7.9 Hz, 1H, Ar-*H*), 7.14 (d, *J* = 7.8 Hz, 2H, Ar-*H*), 7.01 (d, *J* =7.8 Hz, 2H, Ar-*H*), 3.14 (dhept., J_1 = 7.0 Hz, J_2 = 6.9 Hz, 2H, P(C*H*(CH_3)$_2$)$_2$), 2.97 (hept, *J* = 7:0 Hz, 2H, C*H*(CH_3)$_2$), 2.74 (hept, *J* = 6.8 Hz, 2H, C*H*(CH_3)$_2$), 1.50 (d, *J* = 6.8 Hz, 6H, CH(C*H*$_3$)$_2$), 1.24 (dd, J_1 = 13.1 Hz, J_2 = 7.0 Hz, 6H, P(CH(C*H*$_3$)$_2$), 1.23 (d, *J* = 6.8 Hz, 6H, CH(C*H*1.14 (d, *J* = 6.9 Hz, 6H, CH(C*H*1.13 (dd, J_1 = 17.1 Hz, J_2 = 7.0 Hz, 6H, P(CH(C*H*$_3$)$_2$), 1.09 (d, *J* = 6.8 Hz, 6H, CH(C*H*

^{13}C {^{1}H} NMR (150 MHz, C_6D_6; 298 K): δ [ppm] = 152.7 (d, *J* = 8.4 Hz, 1C, triaz-C_Q-P), 147.4 (2C, Ar-C_Q), 145.8 (d, *J* = 1.2 Hz, 2C, Ar-C_Q), 132.8 (1C, Ar-C_Q), 132.1 (1C, Ar-C_Q), 130.9 (1C, Ar-H), 130.8 (1C, Ar-H), 130.9 (d, *J* = 33.2 Hz, 1C, triaz-C_Q=CN_2), 124.3 (2C, Ar-H), 124.2 (2C, Ar-H), 34.6 (1C, *C*N_2), 29.4 (2C, *C*H(CH_3)$_2$), 29.4 (2C, *C*H(CH_3)$_2$), 26.7 (2C, CH(*C*H_3)$_2$), 25.2 (d, *J* = 8.1 Hz, 2C, P(*C*H(CH_3)$_2$)), 24.2 (2C, CH(*C*H_3)$_2$), 23.7 (2C, CH(*C*H_3)$_2$), 22.6 (2C, CH(*C*H_3)$_2$), 21.8 (d, *J* = 24.6 Hz, 2C, P(CH(*C*H_3)$_2$), 21.1 (d, *J* = 10.0 Hz, 2C, P(CH(*C*H_3)$_2$)).

^{31}P {^{1}H} NMR (243 MHz, C_6D_6; 298 K): δ [ppm] = -14.1 (s).

^{31}P NMR (243 MHz, C_6D_6; 298 K): δ [ppm] = -14.1 (dt, J_1 = 24.4 Hz, J_2 = 8.0 Hz).

IR (ATR) [cm^{-1}]: ṽ = 2961, 2929, 2867, 1953 (ṽ(CN_2)), 1528, 1444, 1124, 804, 755.

HR-MS-ESI(+) calc. [M+H2O+H^{+}]: 564.3826, found: 564.3833

Synthesis of 6

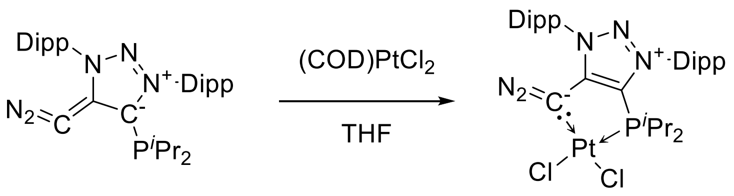

(COD)$PtCl_2$ (69 mg, 185.0 μmol, 1.01 eq.) in THF (20 mL) was added dropwise within 15 minutes in the dark to a solution of diazoalkene **5** (100 mg, 183.2 μmol, 1.0 eq.) in THF (15 mL). The reaction was stirred for 1 h at room temperature, leading to a color change from orange to red and a red solid

precipitate. The solution was filtered off, the precipitate washed twice with THF (5 mL), dissolved in CH_2Cl_2 (3 mL) and dried under reduced pressure. The product **6** was obtained as a red powder (90.3 mg, 111.2 μmol, 61 %).

m.p. 157 °C (decomposition)

^{1}H NMR (500 MHz, CD_2Cl_2, 298 K): δ [ppm] = 7.69 (t, J = 7.9 Hz, 1H, Ar-H), 7.69 (t, J = 7.9 Hz, 1H, Ar-H), 7.45 (d, J = 7.9 Hz, 2H, Ar-H), 7.41 (d, J = 7.9 Hz, 2H, Ar-H), 2.59 (hept. J = 7.6 Hz, 2H, CH(CH_3)$_2$), 2.58 (dhept. J_1 = 6.8 Hz, J_2 = 7.0 Hz, 2H, P(CH(CH_3)$_2$)), 2.59 (hept. J = 6.7 Hz, 2H, CH(CH_3)$_2$), 1.44 (d, J = 6.5 Hz, 6H, CH(CH_3)$_2$), 1.41 (dd, J_1 = 20.1 Hz, J_2 = 8.1 Hz, 6H, P(CH(CH_3)$_2$)), 1.34 (d, J = 6.8 Hz, 6H, CH(CH_3)$_2$), 1.25 (d, J = 6.8 Hz, 6H, CH(CH_3)$_2$), 1.15 (d, J = 6.2 Hz, 6H, CH(CH_3)$_2$), 1.41 (dd, J_1 = 17.7 Hz, J_2 = 7.1 Hz, 6H, P(CH(CH_3)$_2$)).

^{13}C {^{1}H} NMR (127 MHz, CD_2Cl_2; 298 K): δ [ppm] = 166.8 (d, J = 37.6 Hz, 1C, triaz.-C_Q-P), 147.2 (2C, Ar-C_Q), 146.0 (2C, Ar-C_Q), 134.1 (1C, Ar-H), 133.9 (1C, Ar-H), 132.1 (2C, Ar-C_Q), 132.0 (d, J =21.8 Hz, 1C, triaz.-C_Q), 128.2 (2C, Ar-C_Q), 125.4 (2C, Ar-H), 125.0 (2C, Ar-H), 30.0 (2C, CH(CH_3)$_2$), 29.7 (2C, CH(CH_3)$_2$), 27.6 (2C, CH(CH$_3$)$_2$), 25.2 (d, J = 33.7 Hz, 2C, P(CH(CH_3)$_2$)), 25.2 (d, J = 6.7 Hz, 1C, CN$_2$Pt), 24.4 (2C, CH(CH$_3$)$_2$), 23.6 (2C, CH(CH$_3$)$_2$), 21.3 (2C, CH(CH$_3$)$_2$), 18.0 (d, J = 1.2 Hz, 2C, P(CH(CH$_3$)$_2$)), 17.8 (d, J = 2.0 Hz, 2C, P(CH(CH$_3$)$_2$)).

^{31}P {^{1}H} NMR (203 MHz, CD_2Cl_2; 298 K): δ [ppm] = 38.7 (s, $J_{Pt\text{-}P}$ = 2017.3 Hz).

^{195}Pt NMR (129 MHz, CD_2Cl_2; 298 K): δ [ppm] = -3902 (d, J = 4027.4 Hz, 1Pt).

IR (ATR) [cm^{-1}]: ṽ =2965, 2033 ((ṽ(PtC=N$_2$), 1499, 1464, 1366, 1185, 1167, 882, 805, 755, 670, 517.

HR-MS-ESI(+) calc. [M-Cl+MeCN+H$^+$]: 817.3327 found: 817.3247; calc. [M-Cl+MeCN]: 816.3246 found: 816.3246

Synthesis of 7

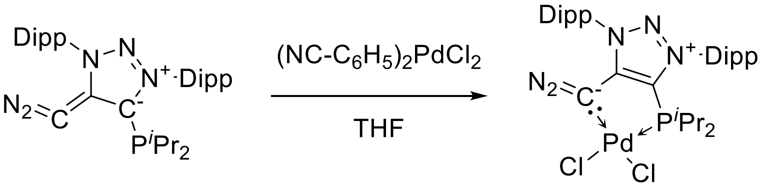

(NC-Ph)$_2$PdCl$_2$ (54.5 mg, 142 μmol, 1.01 eq.) in THF (8 mL) was added dropwise within 5 minutes in the dark to a solution of diazoalkene **5** (76.7 mg, 141 μmol, 1.0 eq.) in THF (15 mL). The reaction was stirred for 1 h at room temperature, leading to a color change from orange to yellow-brown and a yellow-brown solid precipitate. The solution was filtered off, the precipitate washed twice with THF

(5 mL), dissolved in CH_2Cl_2 (3 mL) and dried under reduced pressure. The product **7** was obtained as yellow-brown powder (70.5 mg, 97.5 μmol, 69 %).

m.p. 162 °C (decomposition)

^{1}H NMR (500 MHz, CD_2Cl_2, 298 K): δ [ppm] = 7.69 (t, J = 7.9 Hz, 1H, Ar-H), 7.69 (t, J = 7.9 Hz, 1H, Ar-H), 7.69 (d, J = 7.9 Hz, 2H, Ar-H), 7.69 (t, J = 7.8 Hz, 2H, Ar-H), 2.59 (hept, J = 6.9 Hz, 2H, CH(CH$_3$)$_2$), 2.55 (dhept, J_1 = 6.8 Hz, J_2 = 4.2 Hz, 2H, P(CH(CH$_3$)$_2$)), 2.49 (hept, J = 6.8 Hz, 2H, CH(CH$_3$)$_2$), 1.46 (dd, J_1 = 6.9 Hz, J_2 = 18.0 Hz, 6H, P(CH(CH_3)$_2$)), 1.44 (d, J = 6.8 Hz, 6H, CH(CH_3)$_2$), 1.36 (d, J = 6.9 Hz, 6H, CH(CH_3)$_2$), 1.26 (d, J = 6.9 Hz, 6H, CH(CH_3)$_2$), 1.15 (d, J = 6.7 Hz, 6H, CH(CH_3)$_2$), 1.46 (dd, J_1 = 7.1 Hz, J_2 = 18.0 Hz, 6H, P(CH(CH_3)$_2$)).

^{13}C {^{1}H} NMR (127 MHz, CD_2Cl_2; 298 K): δ [ppm] = 166.0 (d, J = 46.9 Hz, 1C, triaz.-C_Q-P), 134.1 (1C, Ar-H), 133.9 (1C, Ar-H), 132.5 (2C, Ar-C_Q), 132.0 (2C, Ar-C_Q), 130.5 (d, J =21.8 Hz, 1C, triaz.-C_Q), 129.5 (2C, Ar-C_Q), 128.1 (2C, Ar-C_Q), 125.4 (2C, Ar-H), 125.0 (2C, Ar-H), 34.9 (1C, CN$_2$Pd), 30.0 (2C, CH(CH$_3$)$_2$), 29.7 (2C, CH(CH$_3$)$_2$), 27.7 (2C, CH(CH$_3$)$_2$), 25.8 (d, J = 25.7 Hz, 2C, P(CH(CH$_3$)$_2$)), 24.4 (2C, CH(CH$_3$)$_2$), 23.7 (2C, CH(CH$_3$)$_2$), 21.3 (2C, CH(CH$_3$)$_2$), 18.2 (d, J = 1.0 Hz, 2C, P(CH(CH$_3$)$_2$)), 18.1 (d, J = 2.9 Hz, 2C, P(CH(CH$_3$)$_2$)).

^{31}P {^{1}H} NMR (203 MHz, C_6D_6; 298 K): δ [ppm] = 61:8 (s).

IR (ATR) [cm^{-1}]: ṽ = 2966, 2931, 2872, 2034 ((ṽ(PdC=N$_2$),1504, 1465, 1166, 820, 804, 755.

HR-MS-ESI(+) calc. [M-Cl+MeCN]: 727.2636 found: 727.2621.

Synthesis of 11

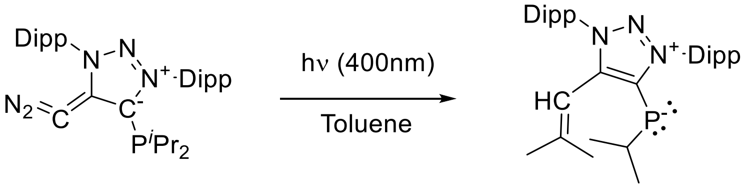

A solution of diazoalkene **5** (50 mg, 92 μmol, 1.0 eq.) in toluene (20 mL) was irradiated with a Kessil LED (400 nm) at a distance of ca. 20 cm for 60 min. The solvent was evaporated and pentane (8 mL) was added to crystalize **11** (33 mg, 64 μmol, 69%) at -40 °C as green crystals suitable for X-ray diffraction.

m.p. 145 °C

^{1}H NMR (500 MHz, C_6D_6, 298 K): δ [ppm] = 7.29 (t, J = 7.8 Hz, 1H, Ar-H), 7.21 (d, J = 7.8 Hz, 2H, Ar-H), 7.14 (t, J = 7.8 Hz, 1H, Ar-H), 6.99 (d, J = 7.8 Hz, 2H, Ar-H), 5.72 (t, J =1.4 Hz, 1H, triaz-C_Q-

C*H*=C_Q) 3.15 (hept, *J* = 6.8 Hz, 2H, C*H*(CH_3)$_2$), 2.80 (hept, *J* = 6.8 Hz, 2H, C*H*(CH_3)$_2$), 2.05 (dhept., J_1 = 6.8 Hz, J_2 = 6.9 Hz, 1H, P(C*H*(CH_3)$_2$), 1.91 (d, *J* = 1.4 Hz, 3H, C_Q(C*H*$_3$)), 1.61 (d, *J* = 6.8 Hz, 6H, CH(C*H*$_3$)$_2$), 1.52 (d, *J* = 1.4 Hz, 3H, C_Q(C*H*$_3$)), 1.39 (d, *J* = 6.8 Hz, 6H, P(CH(C*H*$_3$)$_2$)), 1.22 (d, *J* = 6.8 Hz, 6H, CH(C*H*$_3$)$_2$), 1.13 (d, *J* = 6.9 Hz, 6H, CH(C*H*$_3$)$_2$), 1.12 (d, *J* = 6.9 Hz, 6H, CH(C*H*$_3$)$_2$).

^{13}C {^{1}H} NMR (127 MHz, C_6D_6; 298 K): δ [ppm] = 168.4 (d, *J* = 89.7 Hz, 1C, triaz.-*C*$_Q$-P), 147.1 (d, *J* = 2.8 Hz, 1C, Ar-*C*$_Q$), 146.0 (1C, Ar-*C*$_Q$), 143.9 (1C, *C*$_Q$-(CH_3)$_2$), 134.1 (d, *J* = 12.9 Hz, 1C, triaz.-*C*$_Q$-CH), 132.3 (2C, Ar-C_Q), 131.6 (1C, Ar-H), 131.5 (1C,Ar-*C*$_Q$), 131.1 (1C, Ar-H), 124.8 (2C, Ar-H), 124.4 (2C, Ar-H), 110.1 (1C, triaz-C_Q-*C*H=C_Q), 29.6 (d, *J* = 1.4 Hz, 2C, C*H*(CH_3)$_2$), 29.0 (2C, C*H*(CH_3)$_2$), 25.3 (d, *J* = 22.4 Hz, 2C P(CH(*C*H$_3$)$_2$)), 25.2 (1C, C_Q-*C*H$_3$), 25.0 (2C, CH(*C*H$_3$)$_2$), 25.0 (2C, CH(*C*H$_3$)$_2$), 23.7 (d, *J* = 3.7 Hz, 2C, CH(*C*H$_3$)$_2$), 23.1 (2C, CH(*C*H$_3$)$_2$), 20.9 (d, *J* = 3.4 Hz, 1C, C_Q-*C*H$_3$), 19.6 (d, *J* = 32.3 Hz, 1C, P(*C*H(CH_3)$_2$)).

^{31}P {^{1}H} NMR (202 MHz, C_6D_6; 298 K): δ [ppm] = -12.5 (s).

^{31}P NMR (202 MHz, C_6D_6; 298 K): δ [ppm] = -12.5 (s).

IR (ATR) [cm^{-1}]: ṽ = 2962, 2930, 2869, 1466, 1364, 1340, 1180, 1144, 1111, 984, 822, 803.

HR-MS-ESI(+) calc. [M+H$^+$]: 318.3656, found: 318.3648

Synthesis of 12

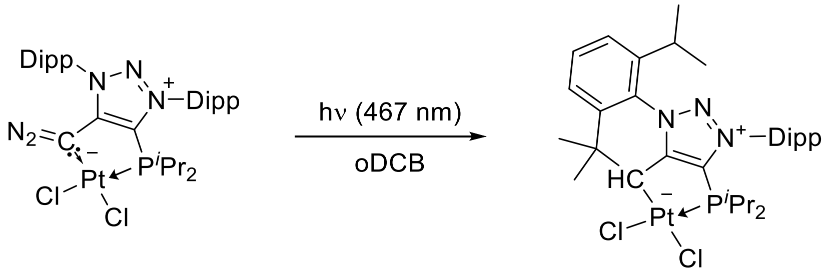

A solution of Pt-P-diazoalkene **6** (10 mg, 12.3 μmol, 1.0 eq.) in *ortho*-dichloro-benzene (0.7 mL) was irradiated for 1 hour at 467 nm at room temperature. A color change from red to yellow was observed. The crude ^{31}P-NMR indicates the formation of two species with δ(^{31}P) = 44.1 ppm in *o*-DCB and δ(^{31}P) = 41.5 ppm in *o*-DCB with an integrated ratio of 21:79. After recrystallisation from CH_2Cl_2 /pentane layer diffusion the mixture could be purified to an integrated ratio of 16:84 [δ(^{31}P) = 45.0 ppm in CD_2Cl_2 : δ(^{31}P) = 42.4 ppm in CD_2Cl_2]. The product **12** was obtained as yellow crystals (7.2 mg, 9.2 μmol, 75 %). The main product in this transformation was identified via X-ray diffraction.

m.p. 152°C (decomposition)

^{1}H NMR (600 MHz, CD_2Cl_2, 298 K): 7.70 (t, J = 7.9 Hz, 1H, Ar-H), 7.55-7.51 (m, 2H, Ar-H), 7.47-7.44 (m, 3H, Ar-H), 3.62 (hept, J = 6.8 Hz, 1H, CH(CH_3)$_2$), 3.40 (d, J = 1.5 Hz, 1H, CH), 2.74 (dhept, J_1 = 14.3 Hz, J_2 = 7.1 Hz, 1H, P(CH(CH_3)$_2$)), 2.55 (dhept. J_1 = 10 Hz, J_2 = 7.0 Hz, 1H, P(CH(CH_3)$_2$)), 2.36 (hept, J = 6.8 Hz, 1H, CH(CH_3)$_2$), 2.29 (hept, J = 6.8 Hz, 1H, CH(CH_3)$_2$), 1.97 (s, 3H, CH_3), 1.47 (dd, J_1 = 18.8 Hz, J_2 = 7.1 Hz, 3H, P(CH(CH_3)$_2$)), 1.41 (dd, J_1 = 18.2 Hz, J_2 = 7.2 Hz, 3H, P(CH(CH_3)$_2$)), 1.39 (d, J = 6.7 Hz, 3H, CH(CH_3)$_2$), 1.35 (d, J = 6.7 Hz, 3H, CH(CH_3)$_2$), 1.26 (d, J = 6.7 Hz, 3H, CH(CH_3)$_2$), 1.24 (d, J = 6.9 Hz, 3H, CH(CH_3)$_2$), 1.16 (d, J = 6.7 Hz, 3H, CH(CH_3)$_2$), 1.09 (dd, J_1 = 18.4 Hz, J_2 = 7.0 Hz, 3H, P(CH(CH_3)$_2$)), 1.05(d, J = 6.8 Hz, 3H, CH(CH_3)$_2$), 0.98 (dd, J_1 = 16.8 Hz, J_2 = 7.1 Hz, 3H, P(CH(CH_3)$_2$)), 0.97 (s, 3H, CH_3).

^{13}C {^{1}H} NMR (150 MHz, CD_2Cl_2; 298 K): 173.7 (d, J = 43.0 Hz, 1C, CH$C_{Triaz.}$), 146.6 (1C, C_Q(CH(CH_3)$_2$), 146.0 (1C, C_Q(CH(CH_3)$_2$), 144.3 (1C, C_Q(C(CH_3)$_2$)CH)), 144.2 (d, J = 11.9 Hz, 1C, $C_{Triaz.}$P), 141.4 (1C, C_Q(CH(CH_3)$_2$), 134.1 (1C, Ar-H), 132.5 (1C, Ar-H), 132.4 (1C, C_Q), 128.3 (1C, C_Q), 126.4 (1C, Ar-H), 125.3 (1C, Ar-H), 125.2 (1C, Ar-H), 124.4 (1C, Ar-H), 42.3 (1C, (CH_3)$_2$$C$CHPt), 30.0 (1C, CH(CH_3), 29.8 (1C, CH(CH_3), 29.6 (1C, CH$_3$), 29.3 (1C, CH$_3$), 28.9 (1C, CH(CH_3), 27.0 (1C, CH(CH$_3$)$_2$), 26.8 (1C, CH(CH$_3$)$_2$), 25.5 (d, J = 37.1 Hz, 1C, P(CH(CH_3)$_2$), 24.7 (1C, CH(CH$_3$)$_2$), 24.5 (d, J = 37.1 Hz, 1C, P(CH(CH_3)$_2$), 23.1 (1C, CH(CH$_3$)$_2$), 21.1 (1C, CH(CH$_3$)$_2$), 21.1 (1C, CH(CH$_3$)$_2$), 19.7 (s, 1C, P(CH(CH$_3$)$_2$), 18.6 (d, J = 1.9 Hz, 1C, P(CH(CH$_3$)$_2$), 18.5 (d, J = 2.2 Hz, 1C, P(CH(CH$_3$)$_2$), 18.2 (d, J = 2.8 Hz, 1C, P(CH(CH$_3$)$_2$), 11.6 (d, J = 2.5 Hz, 1C, CCHPt).

^{31}P {^{1}H} NMR (243 MHz, CD_2Cl_2; 298 K): δ [ppm] = 42.4 (s, $J_{P\text{-}Pt}$ = 2280.1 Hz, 1P).

^{195}Pt NMR (129 MHz, CD_2Cl_2; 298 K): δ [ppm] = -3824 (d, J = 4557.9 Hz, 1Pt).

IR (ATR) [cm^{-1}]: ṽ = 2965, 2929, 2869, 1549, 1463, 1364, 1261, 1100, 1059, 1037, 802, 755, 545.

HR-MS-ESI(+) calc.[M-Cl$^-$+H$^+$] 748.3000, found: 748.2953.

Synthesis of 13

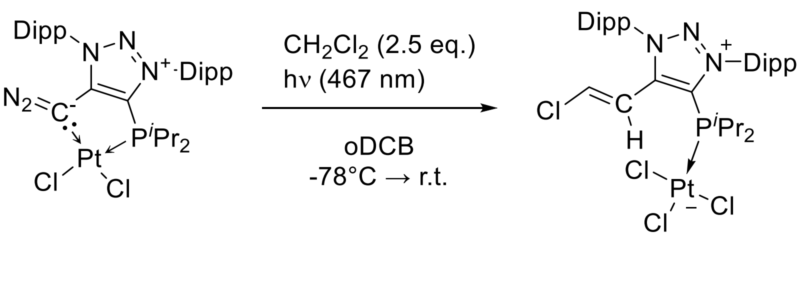

To a solution of Pt-P-diazoalkene **6** (10 mg, 12.3 μmol, 1.0 eq.) in *ortho*-dichloro-benzene (0.7 mL) CH_2Cl_2 (2 μL, 30.8 μmol, 2.5 eq.) was added and irradiated for 15 min at 467 nm at -78 °C. The sample was allowed to warm up and mixed in the dark and subsequently irradiated again at -78°C.

This procedure was repeated until the sample was irradiated for 1 hour in total. A color change from red to yellow was observed. The crude ^{31}P-NMR indicates the formation of four species with $\delta(^{31}P)$ = 44.1 ppm (indicating formation of **15**) in *o*-DCB, $\delta(^{31}P)$ = 41.5 ppm (indicating formation of **12**) in *o*-DCB, $\delta(^{31}P)$ = 28.9 ppm in *o*-DCB and trace amounts of $\delta(^{31}P)$ = 24.5 ppm in *o*-DCB. After crystallization from CH_2Cl_2 (1 mL), C_6D_6 (1 drop) and pentane (4 drops) the product **13** was obtained as yellow crystals (1.2 mg, 1.4 µmol, 11%) suitable for X-ray diffraction. Noteworthy, irradiation in CH_2Cl_2 (as solvent) only results in the formation of compound **12** and **15**.

m.p. 156°C (decomposition)

^{1}H NMR (600 MHz, CD_2Cl_2, 243 K): δ [ppm] = 8.48 (dd, J_1 = 14.3 Hz, J_2 = 1.0 Hz, 1H, Cl–C*H*=C), 7.76 (t, *J* = 7.9 Hz, 1H, Ar-*H*), 7.72 (t, *J* = 7.8 Hz, 1H, Ar-*H*), 7.49 (ddd, J_1 = 11.9 Hz, J_2 = 7.9 Hz, J_3 = 1.3 Hz, 2H, Ar-*H*), 7.49 (ddd, J_1 = 14.0 Hz, J_2 = 7.9 Hz, J_3 = 1.3 Hz, 2H, Ar-*H*), 5.83 (d, *J* = 14.3 Hz, 1H, ClHC=C*H*–$C_{Triaz.}$), 3.06 (dhept. J_1 = 7.0 Hz, J_2 = 6.9 Hz, 1H, P(C*H*(CH_3)$_2$)), 2.88 (hept, *J* = 6.7 Hz, 1H, C*H*(CH_3)$_2$), 2.38 (hept, *J* = 6.6 Hz, 2H, C*H*(CH_3)$_2$), 2.17 (dhept, J_1 = 13.3 Hz, J_2 = 6.8 Hz, 1H, P(C*H*(CH_3)$_2$)), 1.56 (dd, J_1 = 19.1 Hz, J_2 = 7.0 Hz, 3H, P(CH(C*H*$_3$)$_2$)), 1.37(d, *J* = 6.6 Hz, 3H, CH(C*H*$_3$)$_2$), 1.34 (d, *J* = 6.5 Hz, 3H, CH(C*H*$_3$)$_2$), 1.27-1.16 (m, 6H, CH(C*H*$_3$)$_2$, 6H P(CH(C*H*$_3$)$_2$)), 1.14 (d, *J* = 6.8 Hz, 6H, CH(C*H*$_3$)$_2$), 1.10 (d, *J* = 6.8 Hz, 3H, CH(C*H*$_3$)$_2$), 0.99 (dd, J_1 = 16.4 Hz, J_2 = 7.0 Hz, 3H, P(CH(C*H*$_3$)$_2$)), 0.97 (d, *J* = 6.8 Hz, 3H, CH(C*H*$_3$)$_2$).

^{13}C {^{1}H} NMR (150 MHz, CD_2Cl_2; 243 K): δ [ppm] =147.5 (1C, *C*$_Q$(C(CH_3)$_2$), 145.3 (1C, *C*$_Q$(C(CH_3)$_2$), 144.8 (1C, *C*$_Q$(C(CH_3)$_2$), 144.8 (d, *J* = 12.0 Hz, 1C, *C*$_{Triaz.}$P), 144.5 (1C, *C*$_Q$(C(CH_3)$_2$), 134.9 (d, *J* = 9.2 Hz, 1C, *C*$_{Triaz.}$–CH=CHCl), 134.2 (1C, Ar-H), 134.2 (1C, Ar-H), 131.7 (1C, $C_{Triaz.}$–*C*H=CHCl), 131.4 (1C, *C*$_Q$), 128.6 (1C, *C*$_Q$), 126.9 (1C, Ar-H), 125.9 (1C, Ar-H), 125.8 (1C, Ar-H), 124.6 (1C, Ar-H), 118.7 (d, 1C, Cl–*C*H=C), 29.9 (1C, *C*H(CH_3)$_2$), 29.3 (1C, *C*H(CH_3)$_2$), 29.2 (1C, *C*H(CH_3)$_2$), 28.9 (1C, *C*H(CH_3)$_2$), 27.4 (1C, CH(*C*H$_3$)$_2$), 26.8 (1C, CH(*C*H$_3$)$_2$), 26.4 (d, *J* = 30.5 Hz, 1C, P(*C*H(CH_3)$_2$)), 24.9 (1C, CH(*C*H$_3$)$_2$), 24.1 (1C, CH(*C*H$_3$)$_2$), 23.6 (1C, CH(*C*H$_3$)$_2$), 23.5 (d, *J* = 5.7 Hz, 1C, P(CH(*C*H$_3$)$_2$)),23.4 (1C, CH(*C*H$_3$)$_2$), 22.8 (d, *J* = 34.4 Hz, 1C, P(*C*H(CH_3)$_2$)), 21.6 (1C, CH(*C*H$_3$)$_2$), 21.2 (1C, CH(*C*H$_3$)$_2$), 20.6 (1C, P(CH(*C*H$_3$)$_2$)), 18.2 (d, *J* = 5.4 Hz, 1C, P(CH(*C*H$_3$)$_2$)), 16.5 (1C, P(CH(*C*H$_3$)$_2$)).

^{31}P {^{1}H} NMR (243 MHz, CD_2Cl_2; 243 K): δ [ppm] = 31.1 (s, 1P, P).

IR (ATR) [cm^{-1}]: ṽ = 2966, 2930, 2872, 1678, 1622, 1465, 1389, 1260, 1197, 1018, 797, 615.

HR-MS-ESI(+) calc.[M-Cl^{-}]: 832.2534, found: 832.2472.

Synthesis of 14

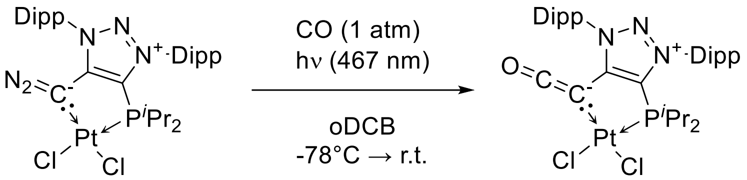

In a J-Young NMR tube the Pt-complex **6** (5.0 mg, 6.2 μmol, 1.0 eq.) was dissolved in 1,2-dichlorobenzene (0.7 mL) and the nitrogen atmosphere was exchanged with 1 atm. of dried carbon monoxide. The reaction was monitored by unlocked ^{31}P-NMR experiments up to 4 days which showed no reaction at room temperature. After the sample was cooled to -78 °C and irradiated for 15 min at 467 nm (Kessil LED; 20 cm distance), the sample was warmed up to r.t. in the dark. The cooling/irradiation steps were repeated 3 times until full conversion was reached. The crude product was precipitated by addition of pentane (10 mL), filtered and dried in vacuo. The product was recrystallized from a mixture of *o*-DCB (0.1 mL), CH_2Cl_2 (0.7 mL) and pentane (1.7 mL) by layer diffusion to obtain yellow crystals. The product was obtained by separating of the yellow crystals of **14** (2.5 mg, 3.1 μmol, 50 %) from the colorless amorphous side products.

m.p. 143 °C (decomposition)

^{1}H NMR (600 MHz, CD_2Cl_2, 298 K): 7.71 (t, J =7.9 Hz, 1H, Ar-H), 7.69 (t, J =7.9 Hz, 1H, Ar-H), 7.46 (d, J =7.9 Hz, 2H, Ar-H), 7.45 (t, J =7.9 Hz, 2H, Ar-H), 2.59 (dhept, J_1 = 9.2 Hz, J_2 = 7.1 Hz, 2H, P(CH(CH$_3$)$_2$), 2.43 (dtt, J_1 = 13.6 Hz, J_2 = 6.8 Hz, J_3 = 3.1 Hz, 4H, CH(CH$_3$)$_2$), 1.43 (d, J = 6.7 Hz, 6H, CH(CH_3)$_2$), 1.41 (dd, J_1 = 17.7 Hz, J_2 = 7.0 Hz, 6H, P(CH(CH_3)$_2$), 1.35 (d, J = 6.8 Hz, 6H, CH(CH_3)$_2$), 1.23 (d, J = 6.8 Hz, 6H, CH(CH_3)$_2$), 1.14 (d, J = 6.7 Hz, 6H, CH(CH_3)$_2$), 0.97 (dd, J_1 = 17.7 Hz, J_2 = 7.0 Hz, 6H, P(CH(CH_3)$_2$).

^{13}C {^{1}H} NMR (151 MHz, CD_2Cl_2; 298 K): 176.6 (O=C_Q=C), 169.3 (d, J = 37.1 Hz, 1C, $C_{Triaz.}$C=C=O), 146.8 (2C, C_QCH(CH$_3$)$_2$), 146.0 (2C, C_QCH(CH$_3$)$_2$), 137.1 (d, J = 22.2 Hz, 1C, $C_{Triaz.}$P), 134.2 (1C, Ar-H), 134.1 (1C, Ar-H), 132.9 (1C, N$C_{Q\text{-}Ar}$), 132.2 (1C, N$C_{Q\text{-}Ar}$), 127.8 (1C, O=C=C_Q), 125.7 (2C, Ar-H), 125.4 (2C, Ar-H),30.0 (2C, CH(CH$_3$)$_2$), 29.7 (2C, CH(CH$_3$)$_2$), 27.6 (2C, CH(CH$_3$)$_2$), 25.2 (d, J = 33.6 Hz, 2C, PCH(CH$_3$)$_2$),24.6 (2C, CH(CH$_3$)$_2$), 23.8 (2C, CH(CH$_3$)$_2$), 21.3 (2C, CH(CH$_3$)$_2$), 18.0 (d, J = 1.7 Hz, 2C, PCH(CH$_3$)$_2$), 17.8 (d, J = 2.0 Hz, 2C, PCH(CH$_3$)$_2$).

^{31}P {^{1}H} NMR (243 MHz, CD_2Cl_2; 298 K): δ [ppm] = 38.3 (s, 1P, P).

^{195}Pt NMR (129 MHz, CD_2Cl_2; 298 K): δ [ppm] = -3834 (d, J = 4044.8 Hz, 1Pt).

IR (ATR) [cm^{-1}]: ṽ = 2965, 2930, 2872, 2078 (br), 1535, 1465, 1259, 1088, 954, 796.

HR-MS-ESI(+) calc. [M+MeCN+H$^+$-Cl$^-$] 817.3210, found: 817.3150.

3. NMR spectra

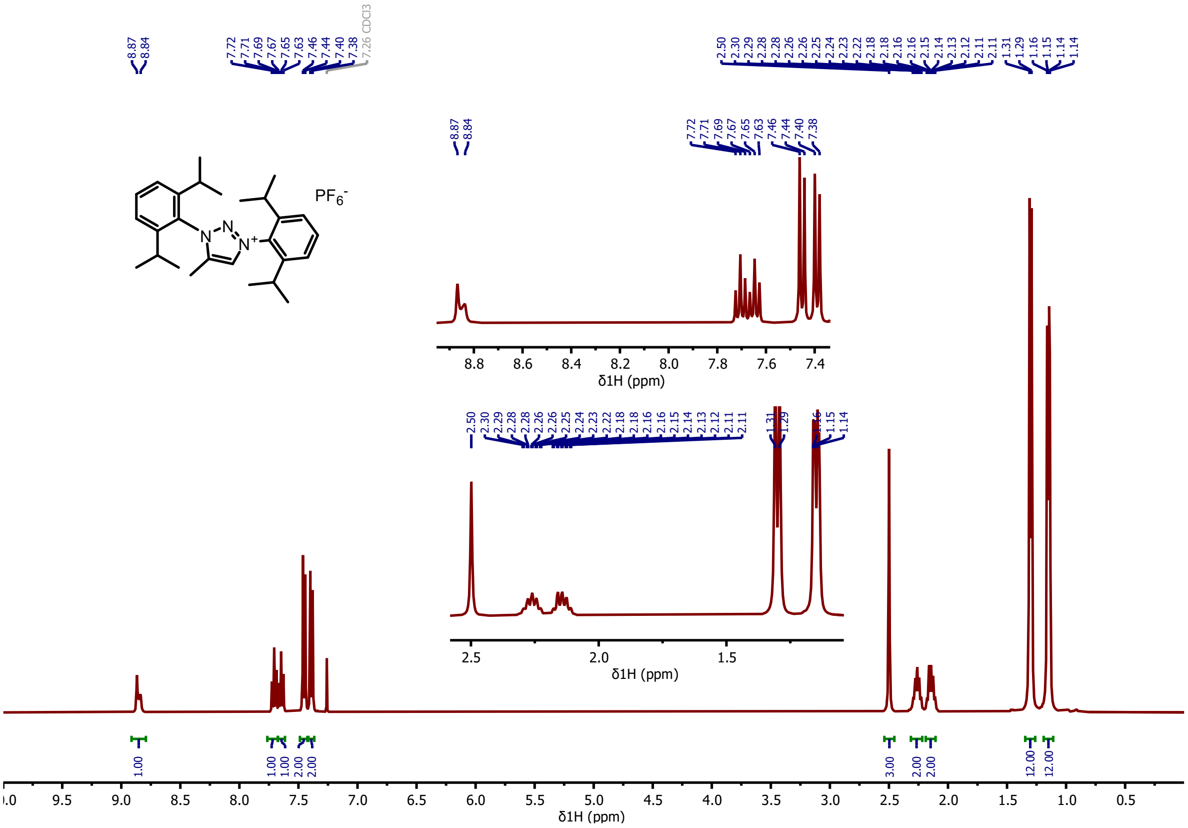

Fig S1: ^{1}H NMR (400 MHz, $CDCl_3$, 298K) of **1**.

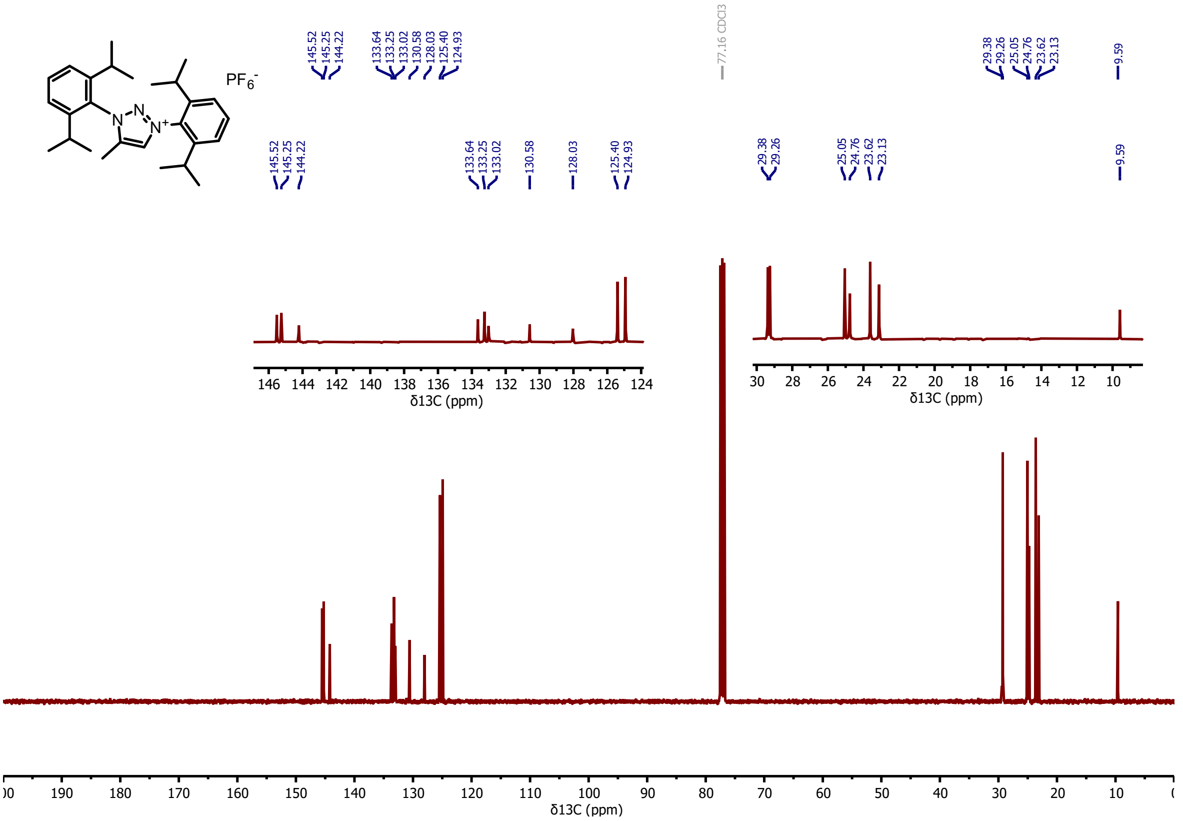

Fig S2: ^{13}C {^{1}H} NMR (100 MHz, $CDCl_3$, 298K) of **1**.

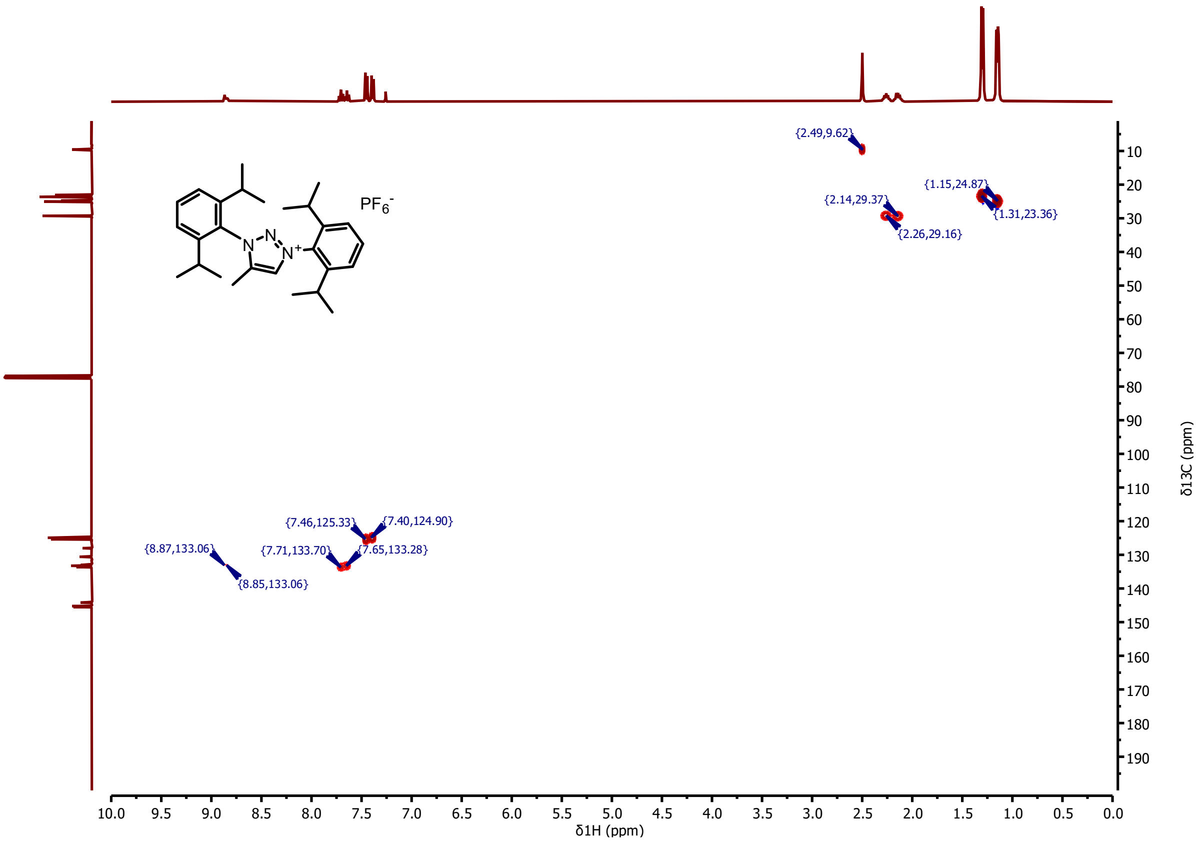

Fig S3: **^{1}H/^{13}C HSQC** (400/100 MHz, $CDCl_3$, 298K) of **1.**

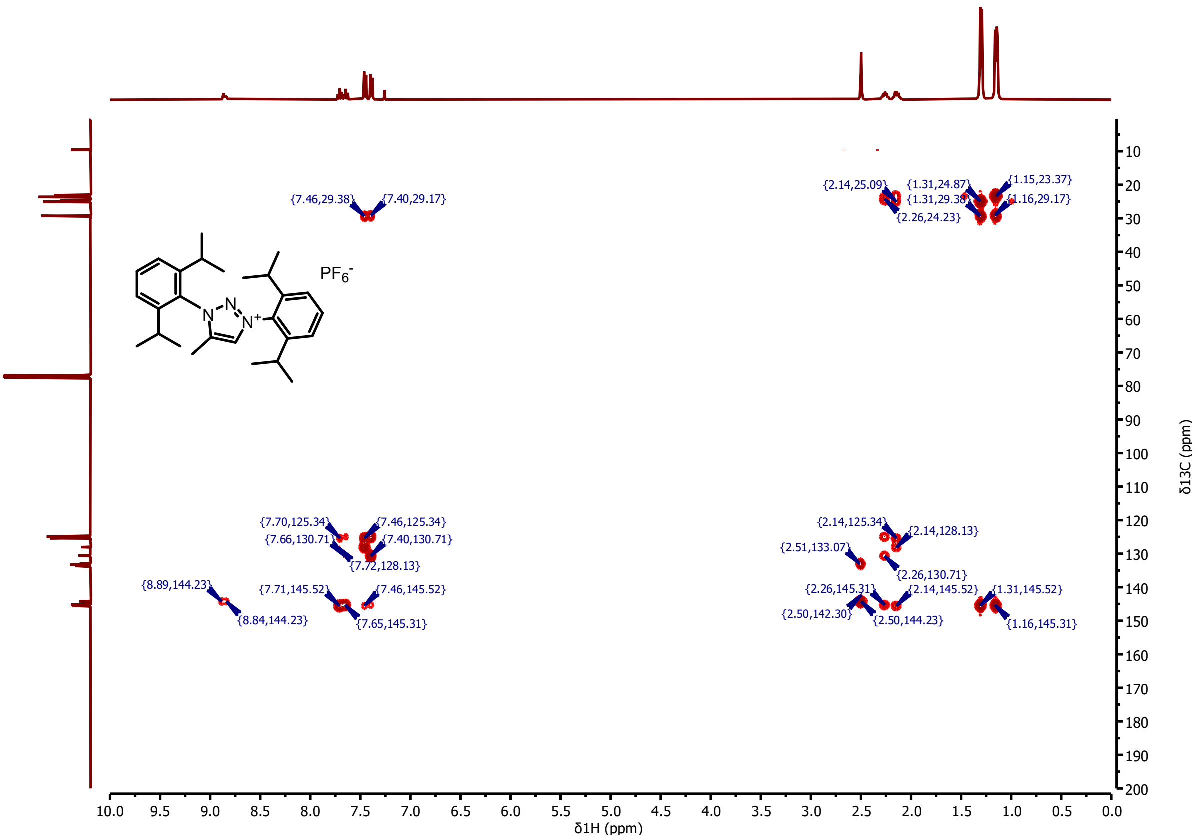

Fig S4: **^{1}H/^{13}C HMBC** (400/100 MHz, $CDCl_3$, 298K) of **1.**

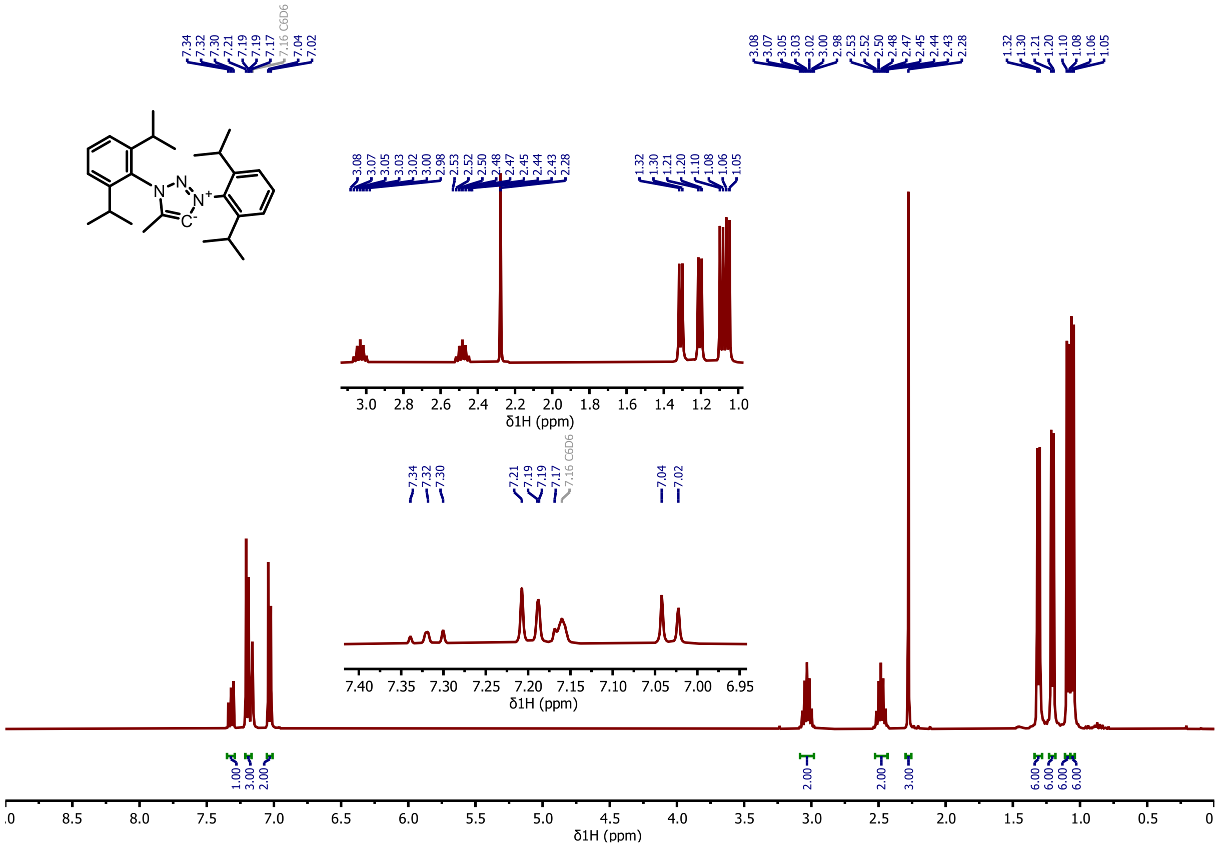

Fig S5: ^{1}**H NMR** (400 MHz, C_6D_6, 298K) of **2.**

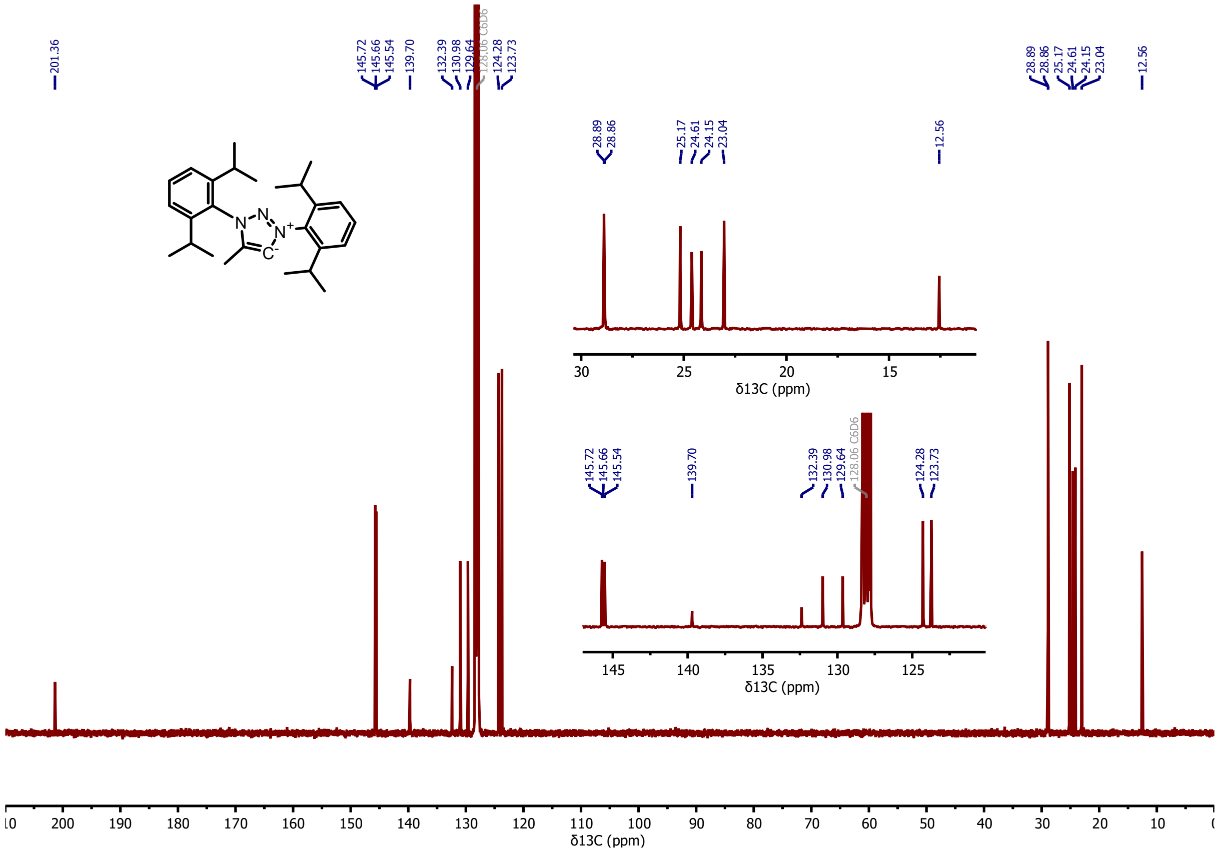

Fig S6: ^{13}**C NMR** (400 MHz, C_6D_6, 298K) of **2.**

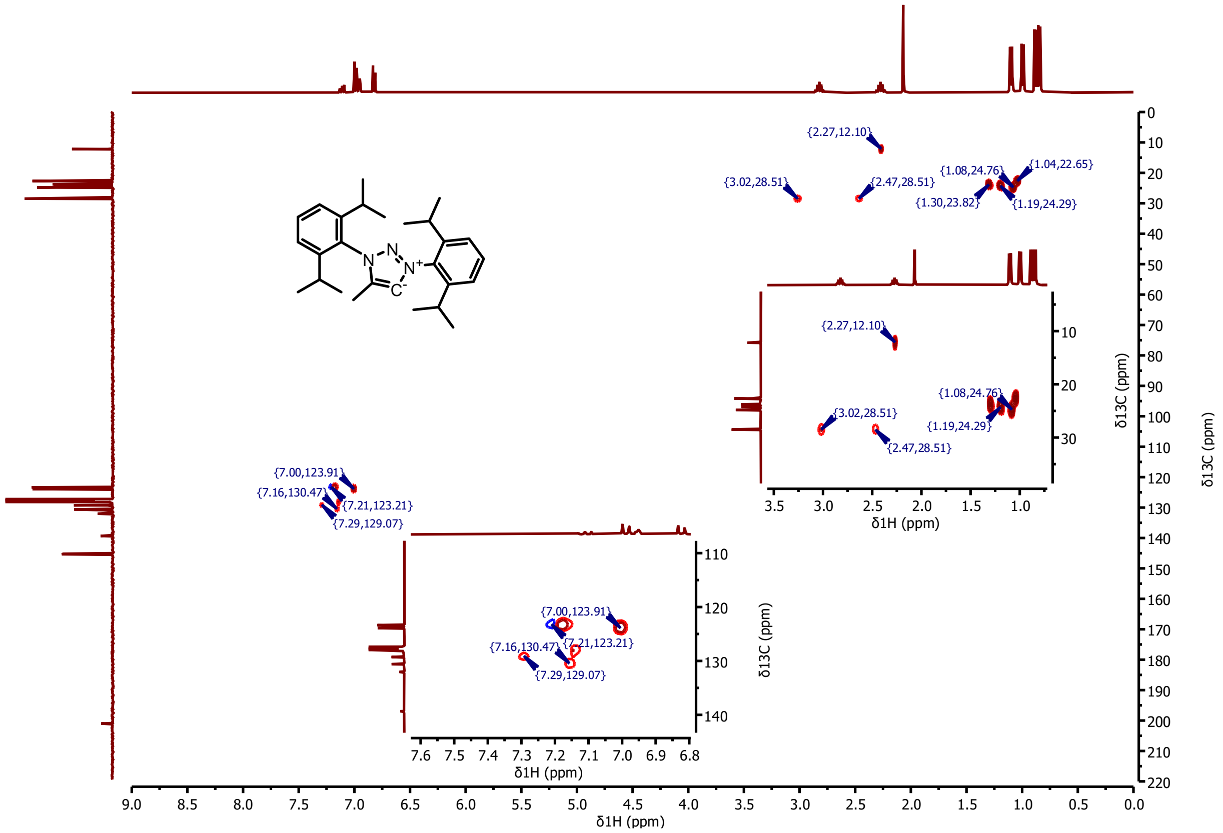

Fig S7: ^{1}**H/**^{13}**C HSQC** (500/127 MHz, C_6D_6, 298K) of **2.**

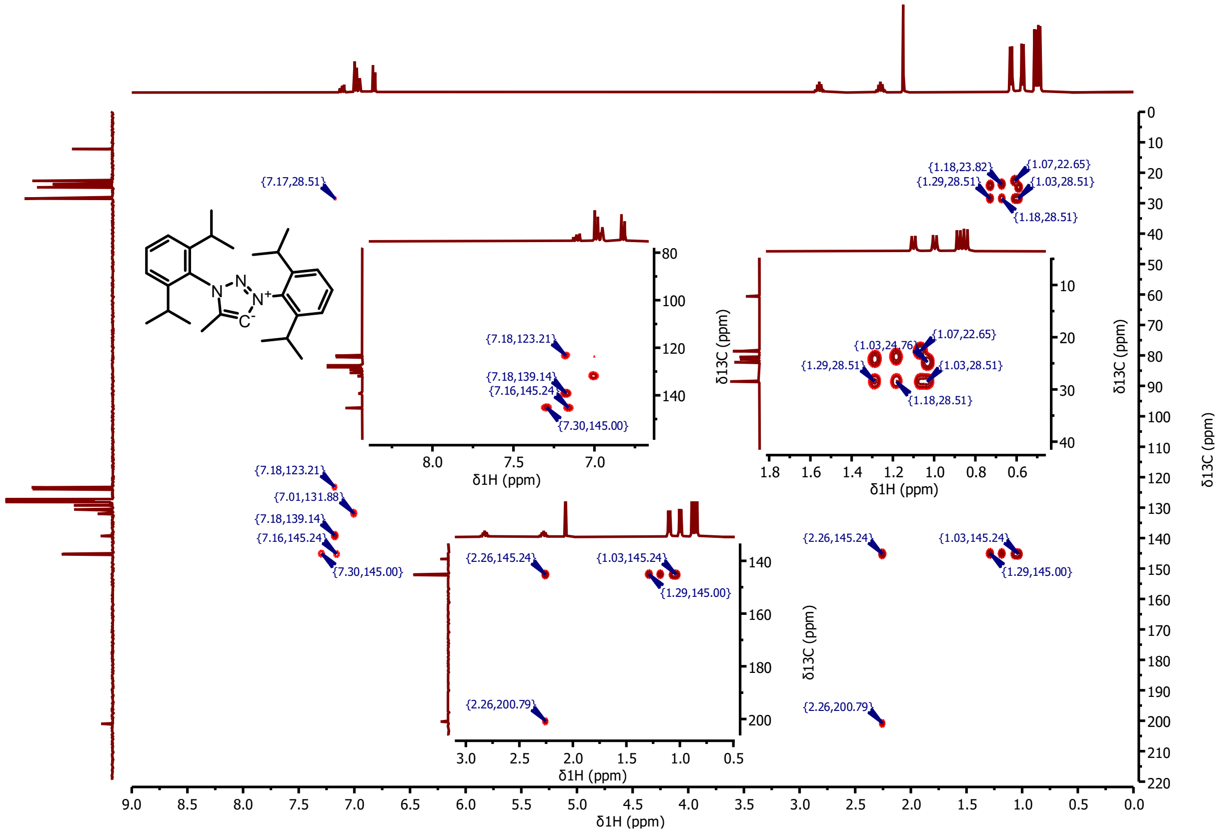

Fig S8: ^{1}**H/**^{13}**C HMBC** (500/127 MHz, C_6D_6, 298K) of **2.**

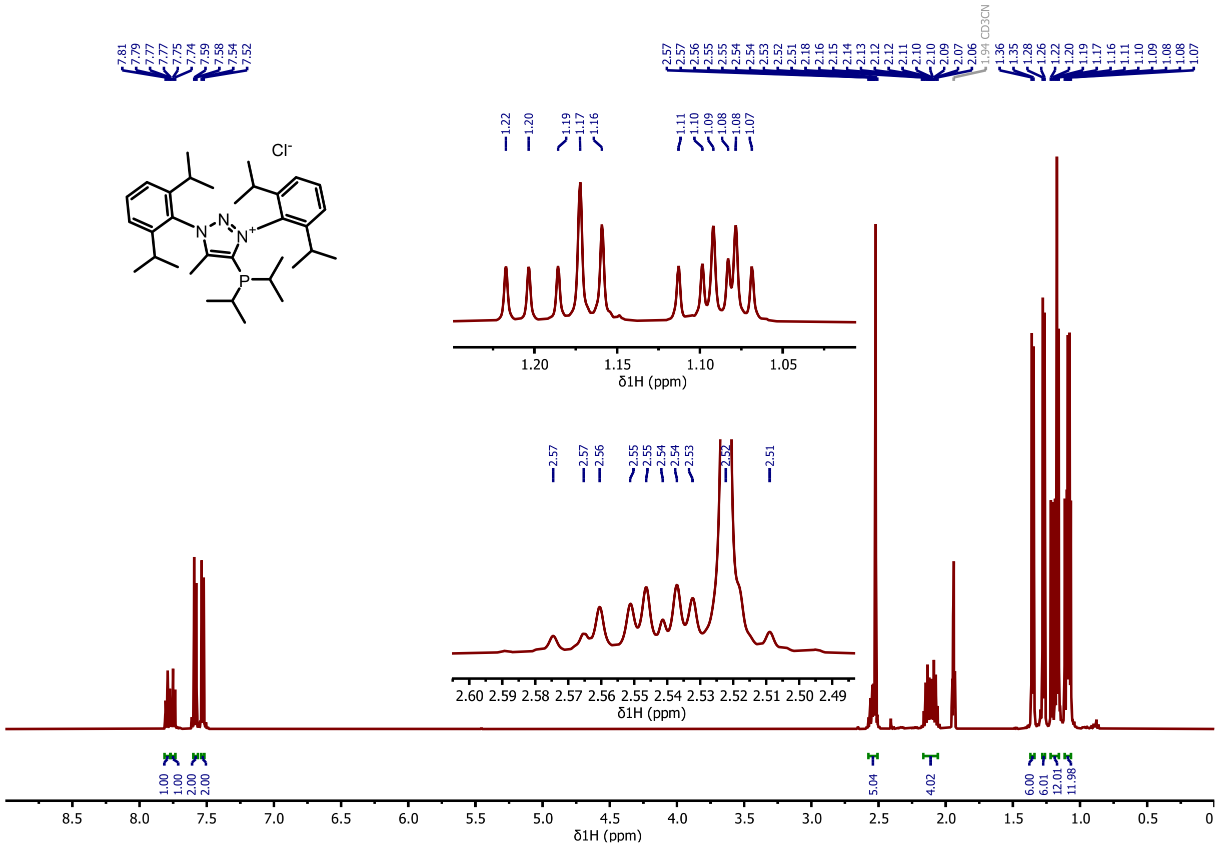

**Fig S9: **

Fig S9: ^{1}**H NMR** (500 MHz, CD_3CN, 298K) of **3.**

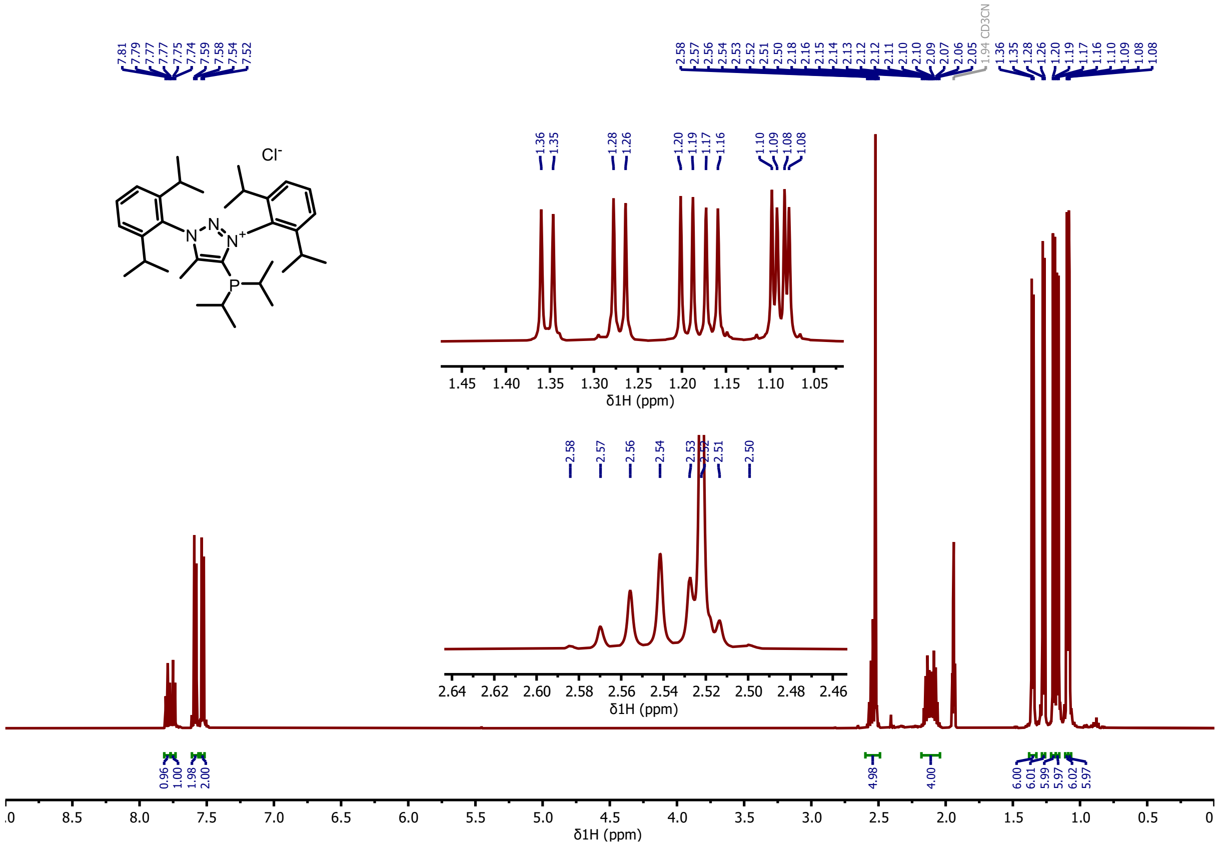

Fig S10: ^{1}**H NMR {31P}** (500 MHz, CD_3CN, 298K) of **3.**

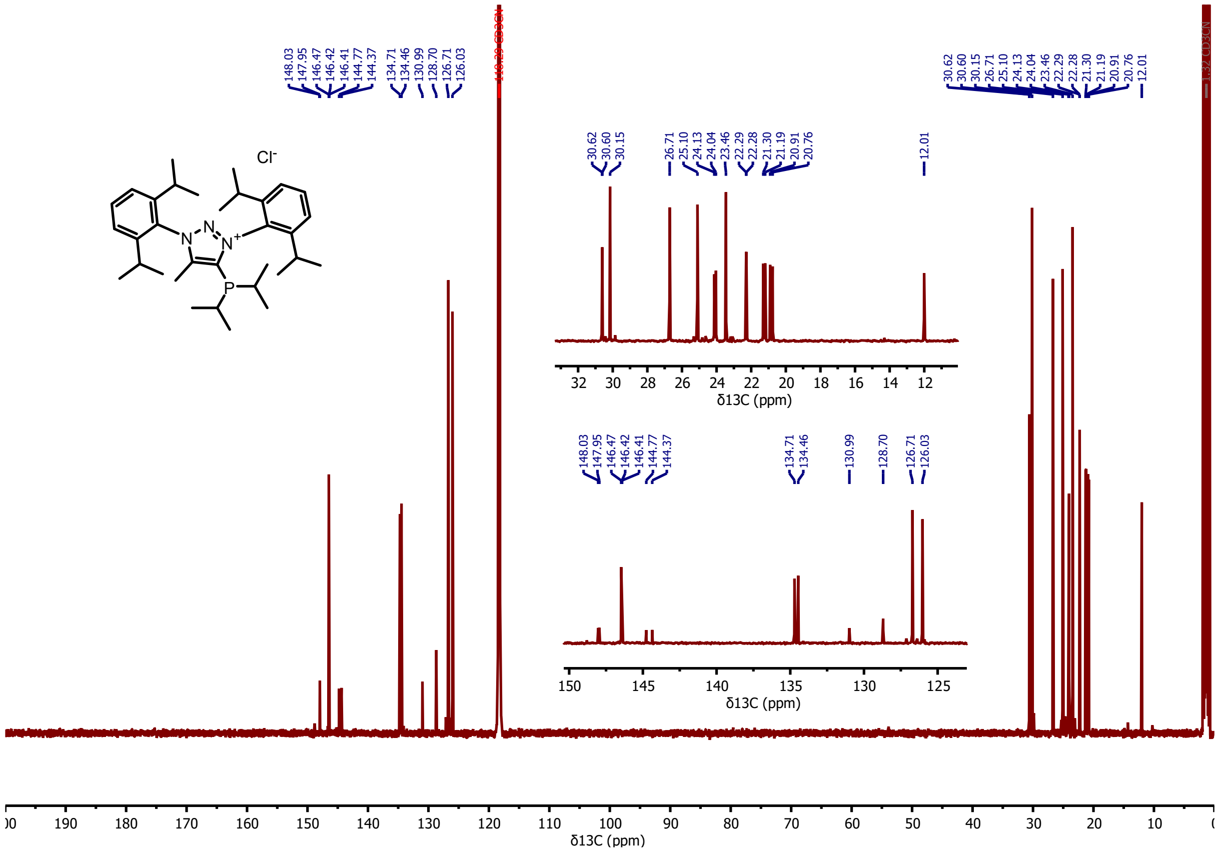

Fig S11: ^{13}C NMR (127 MHz, CD_3CN, 298K) of **3.**

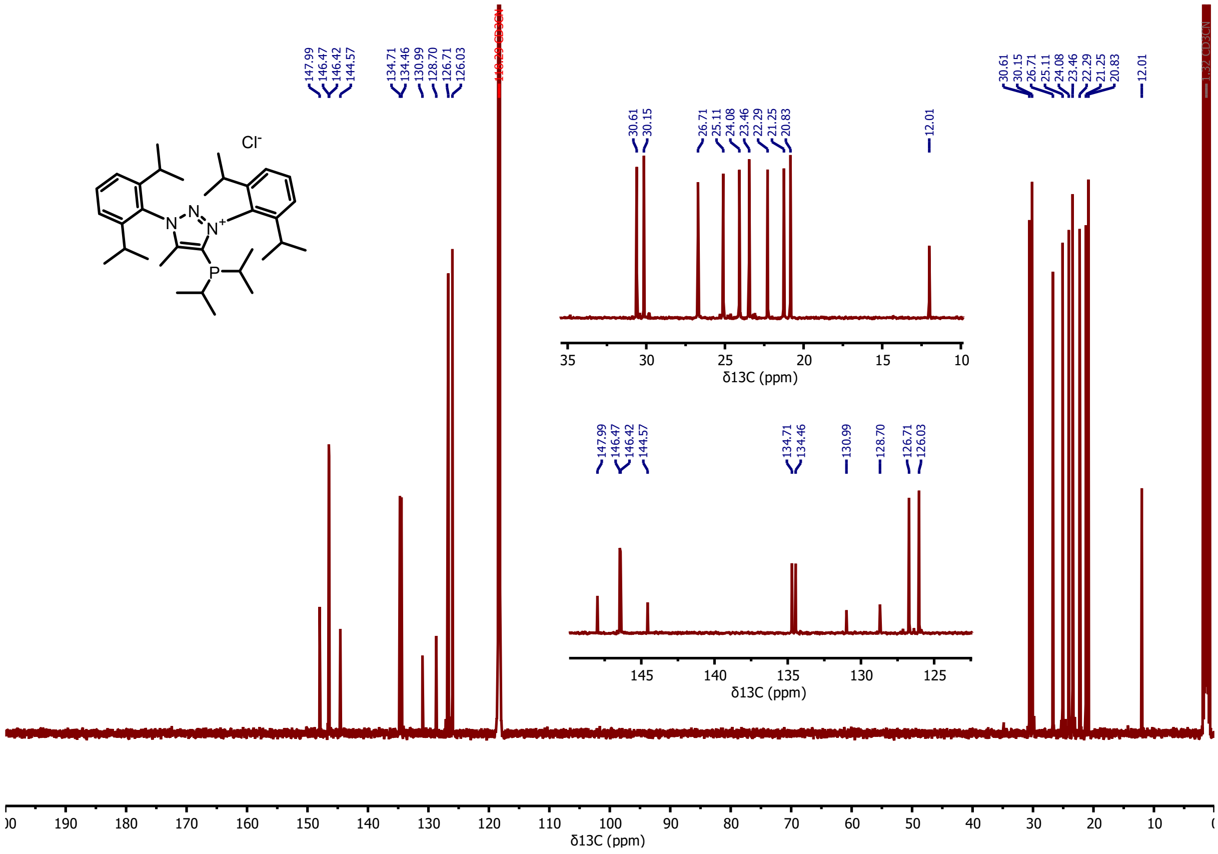

Fig S12: ^{13}C NMR {31P} (127 MHz, CD_3CN, 298K) of **3.**

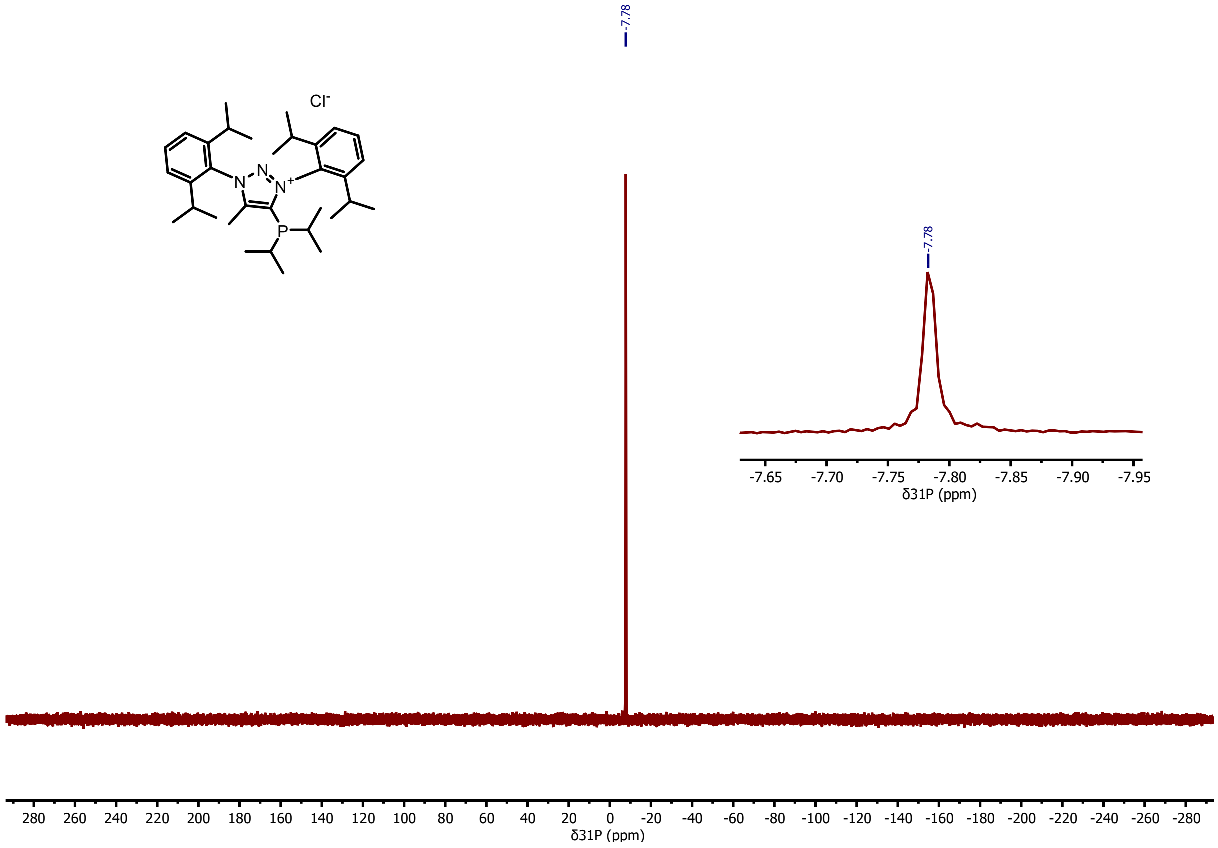

Fig S13: ^{31}P NMR {1H} (200 MHz, CD_3CN, 298K) of **3.**

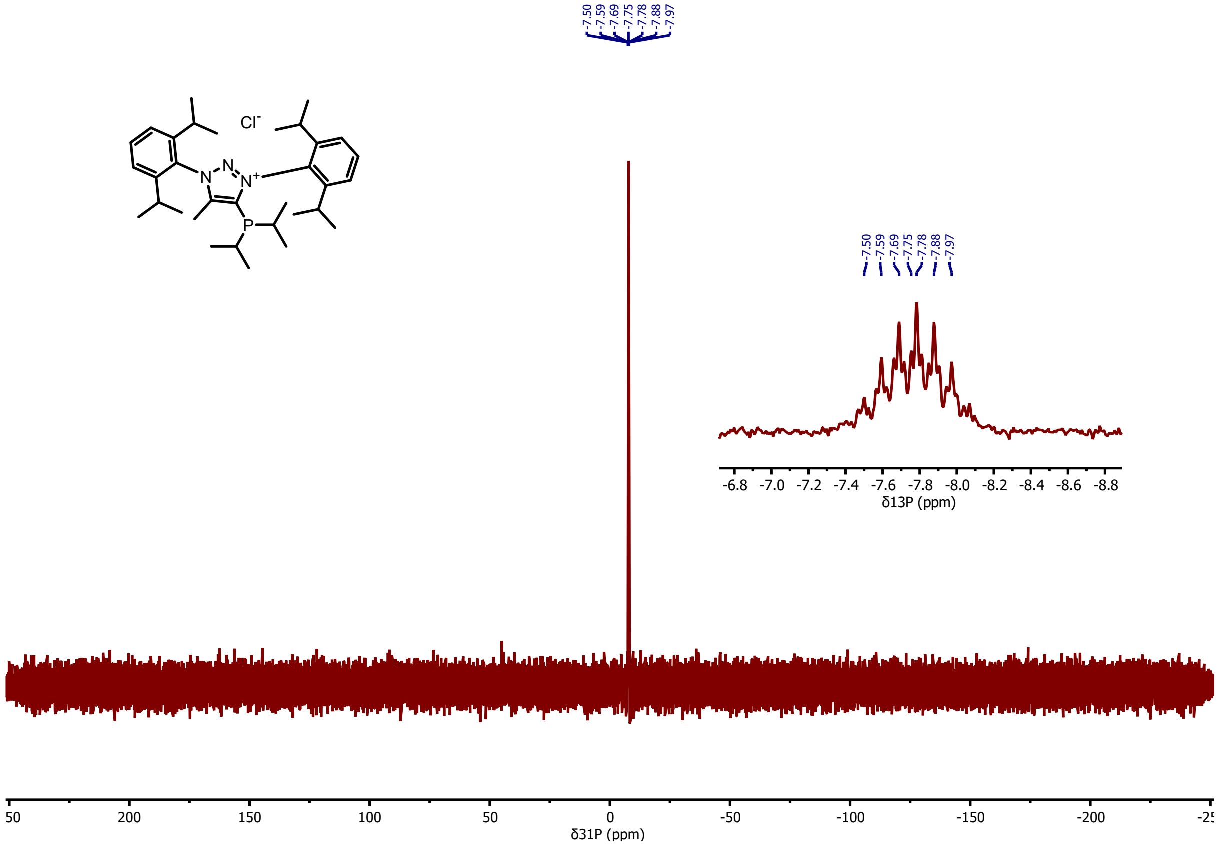

Fig S14: ^{31}P NMR (162 MHz, CD_3CN, 298K) of **3.**

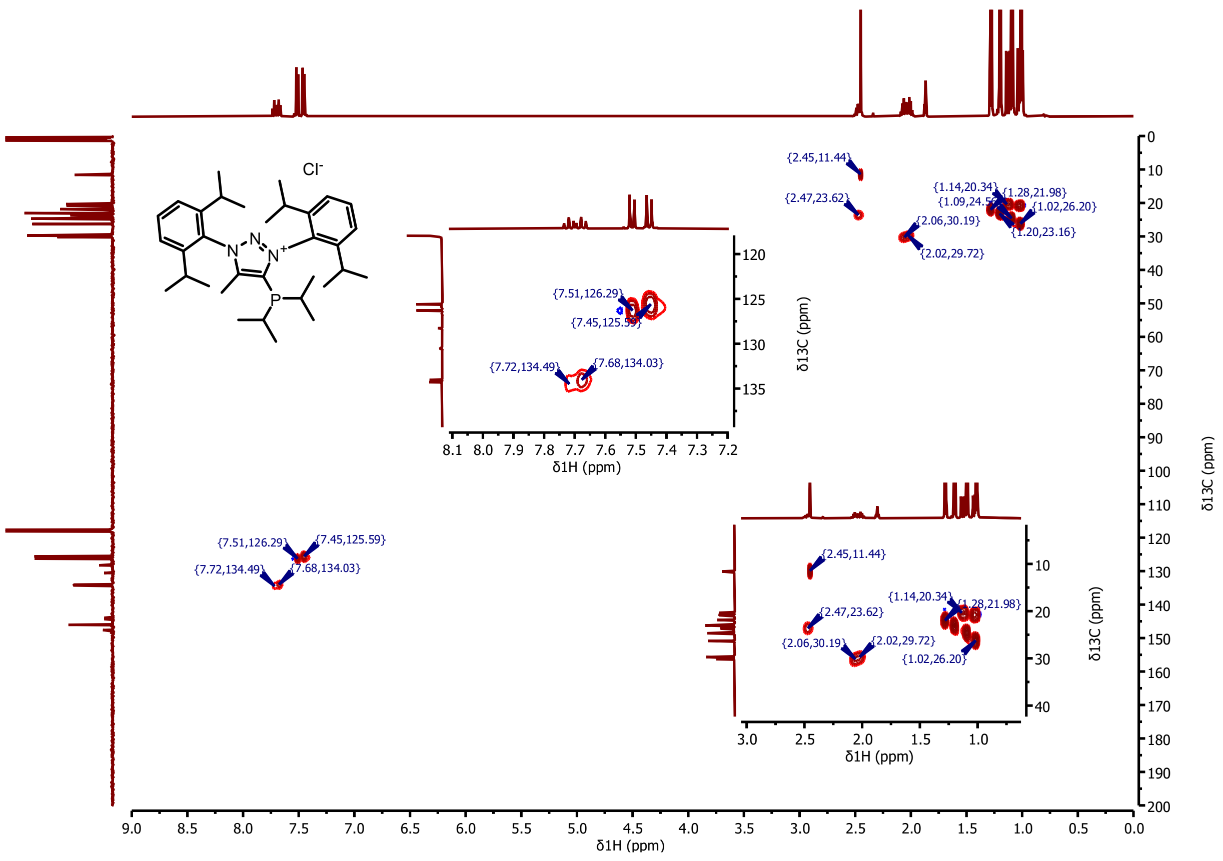

Fig S15: $^{1}H/^{13}C$ HSQC (500/127 MHz, $CDCl_3$, 298K) of **3.**

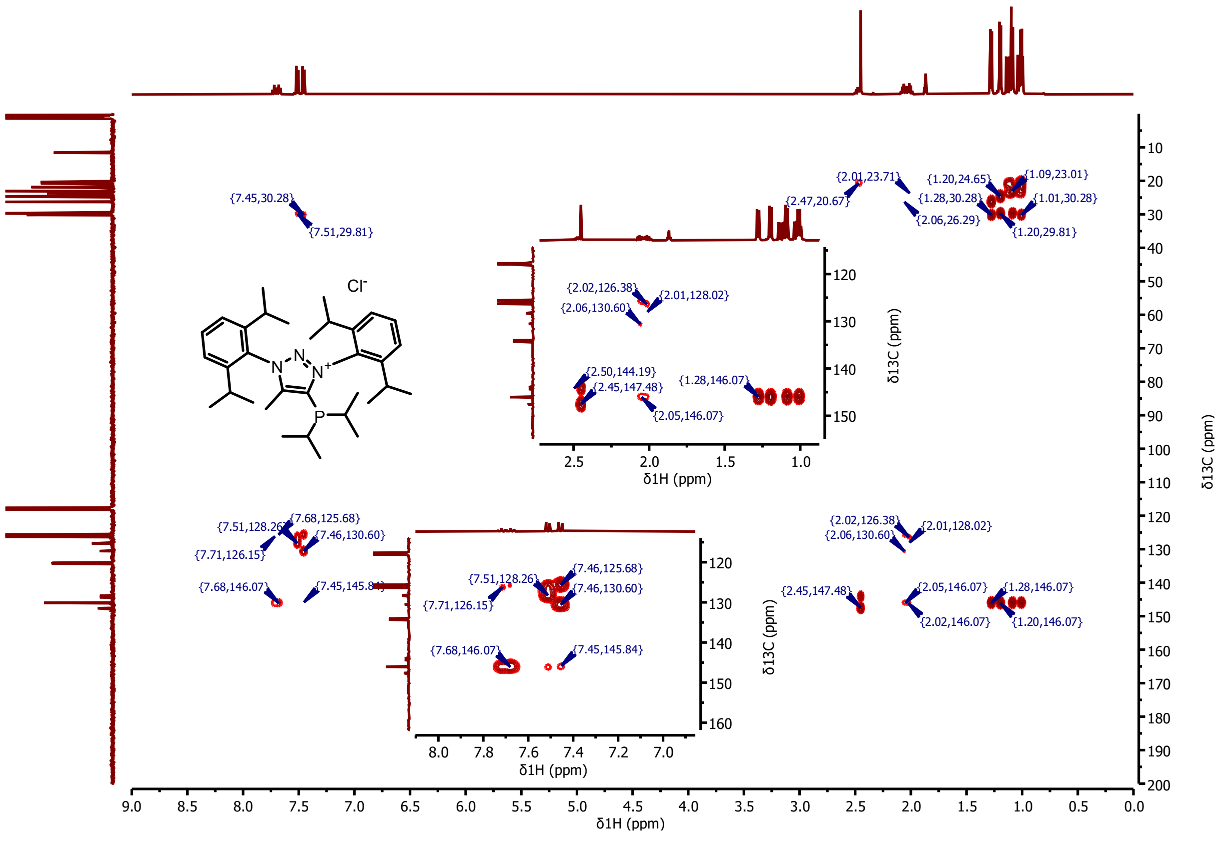

Fig S16: $^{1}H/^{13}C$ HMBC (500/127 MHz, $CDCl_3$, 298K) of **3.**

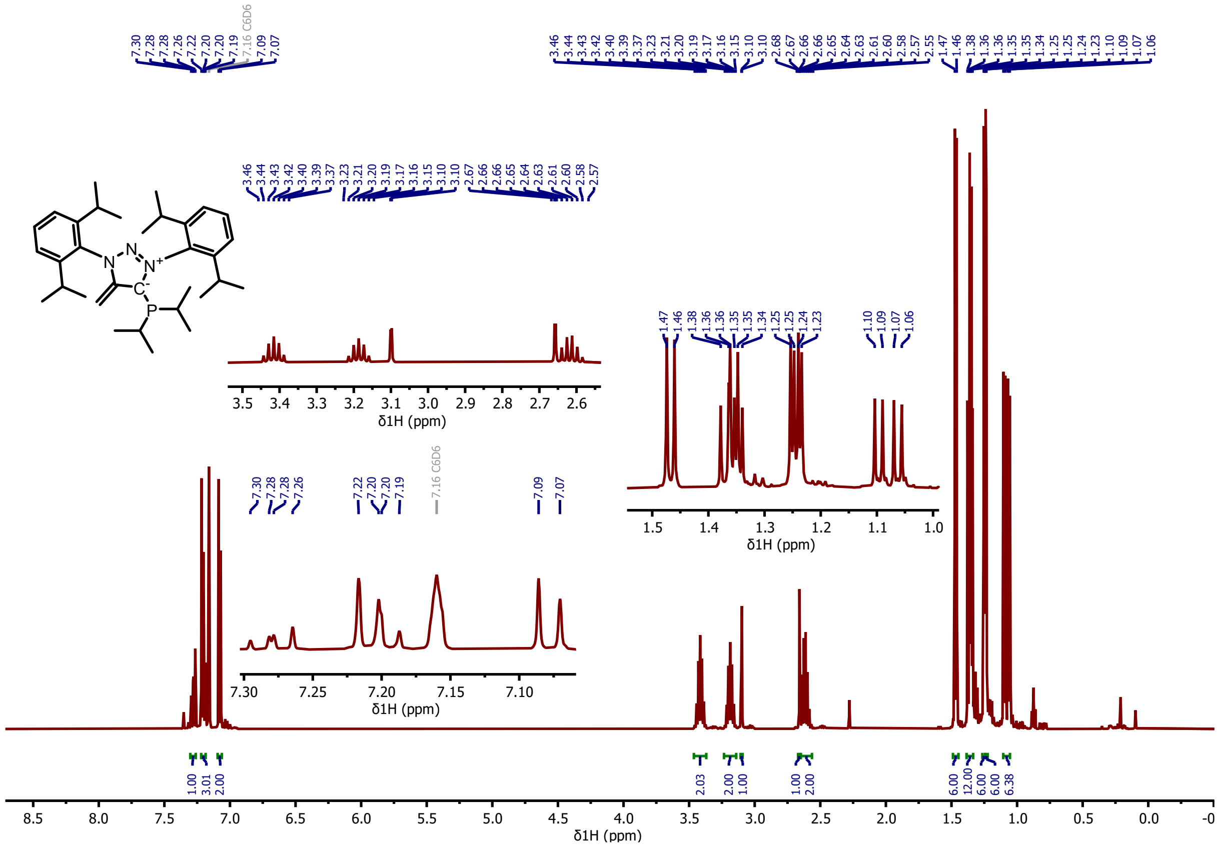

Fig S17: ^{1}**H NMR** (500 MHz, C_6D_6, 298K) of **4.**

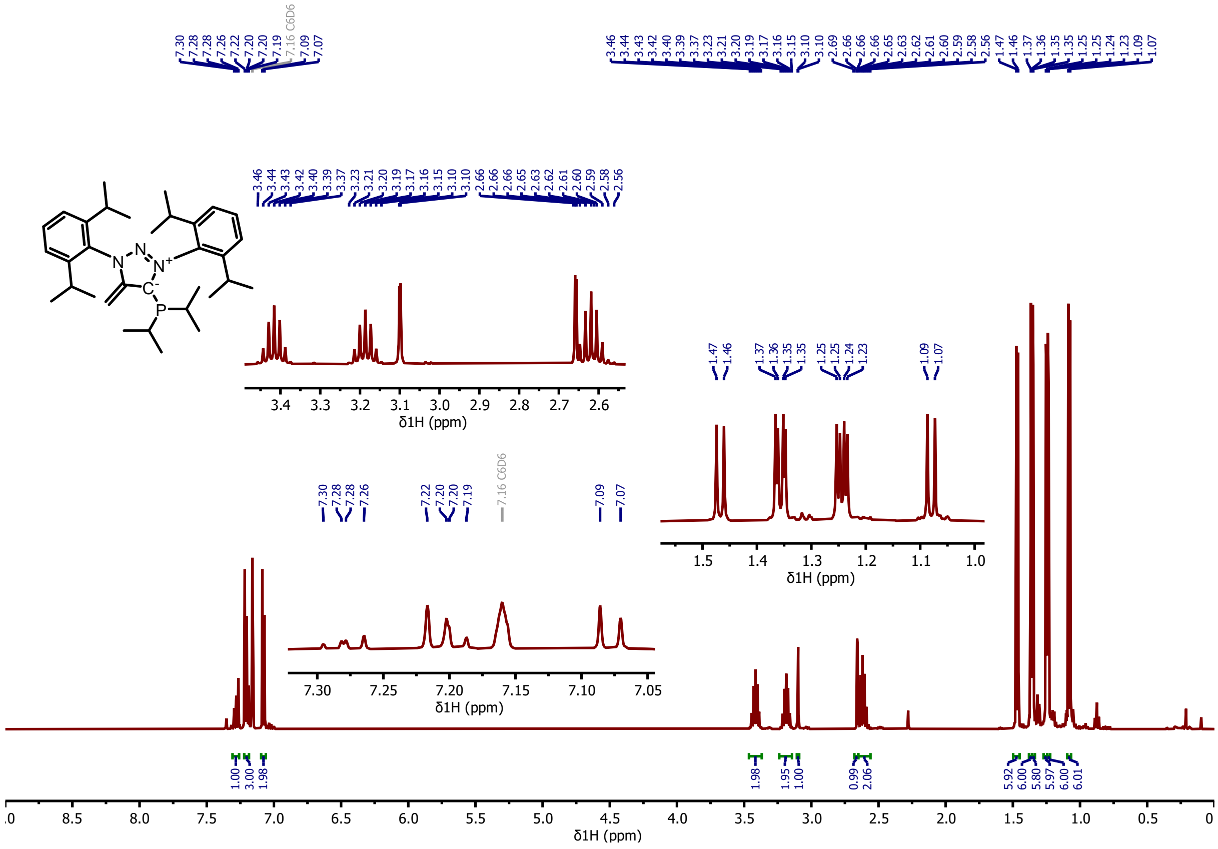

Fig S18: ^{1}**H NMR {^{31}P}** (500 MHz, C_6D_6, 298K) of **4.**

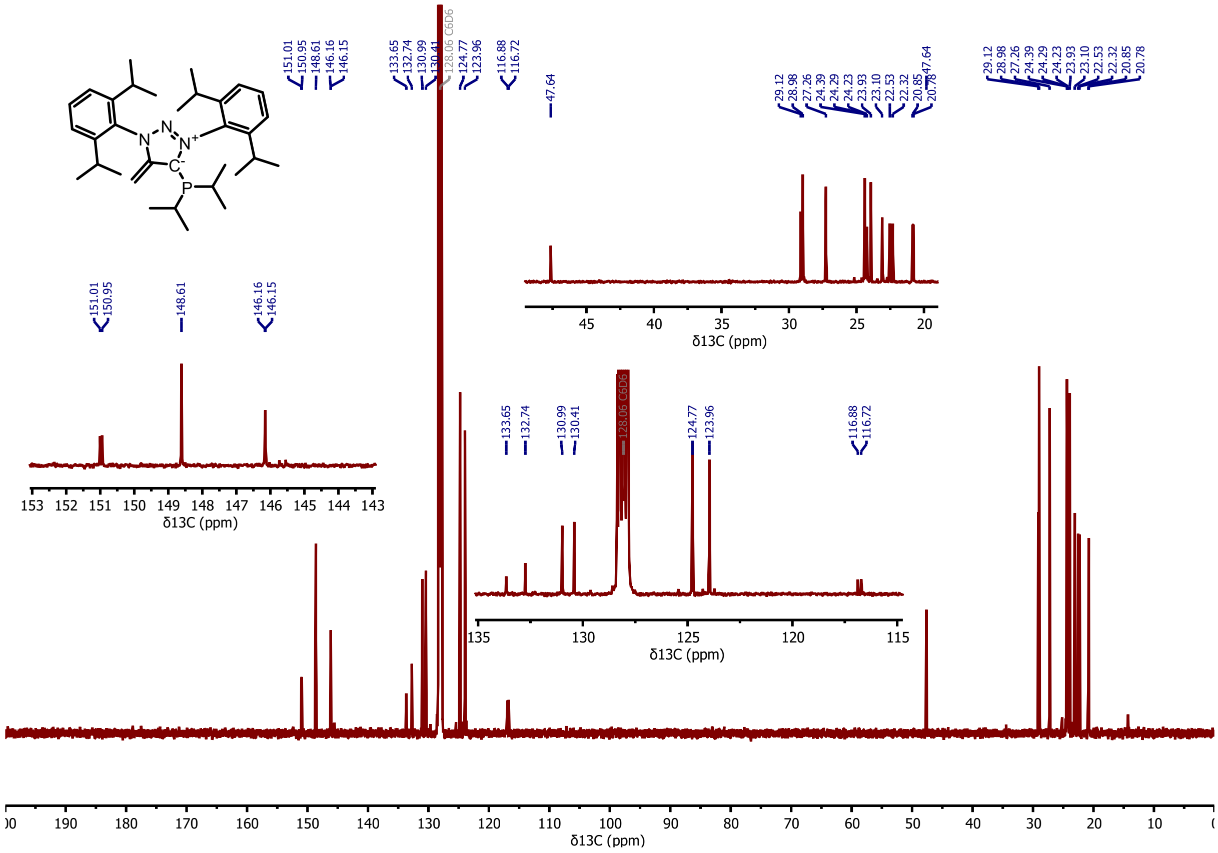

Fig S19: ^{13}C NMR (127 MHz, C_6D_6, 298K) of **4.**

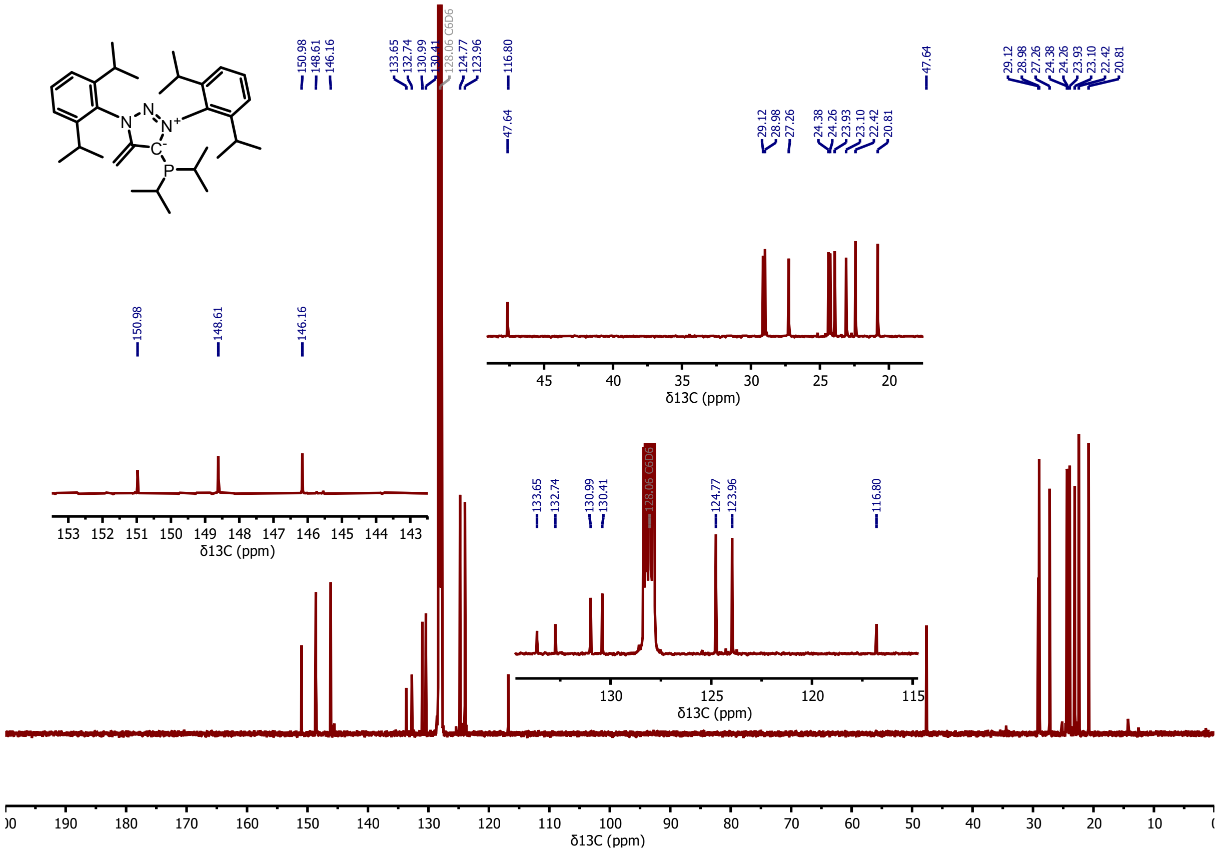

Fig S20: ^{13}C NMR {^{31}P} (127 MHz, C_6D_6, 298K) of **4.**

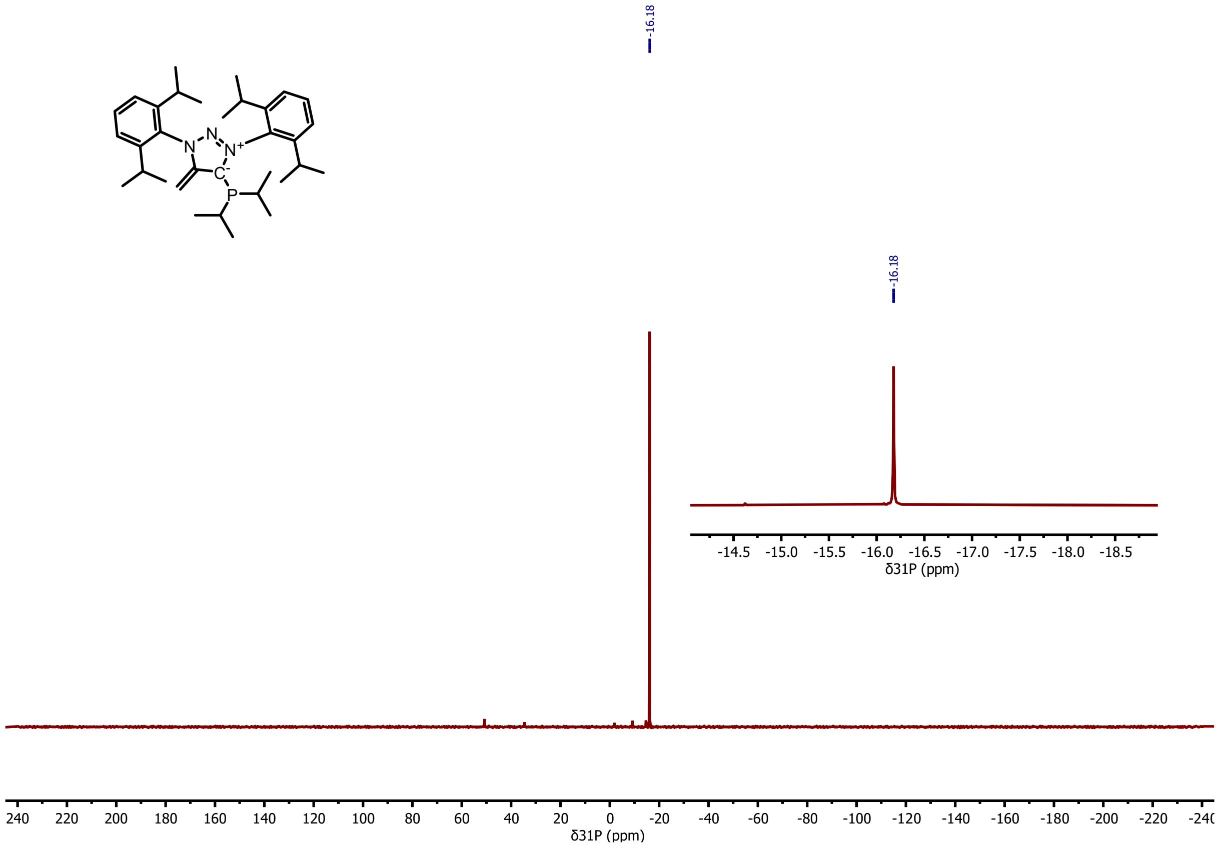

Fig S21: ^{31}**P NMR** (243 MHz, C_6D_6; 298 K) of **4.**

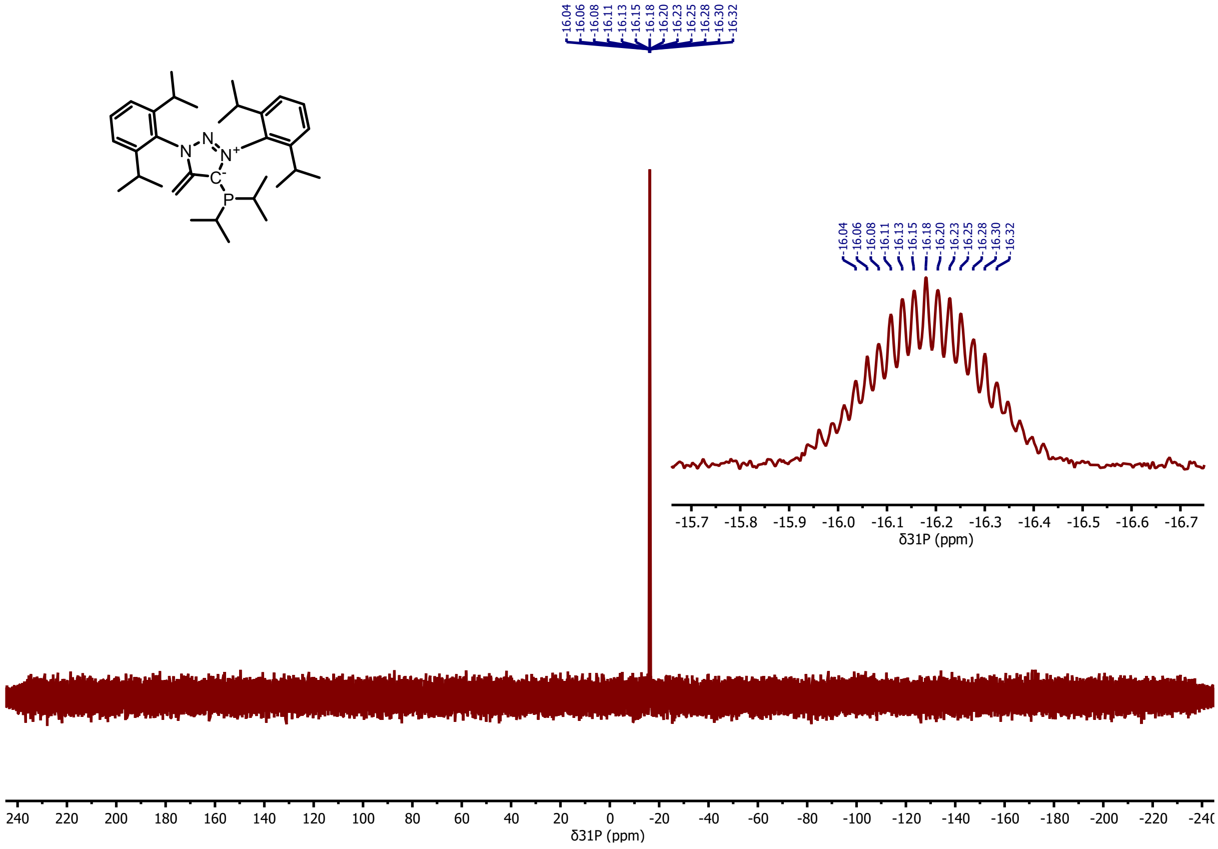

Fig S22: ^{31}**P NMR** (200 MHz, C_6D_6; 298 K) of **4.**

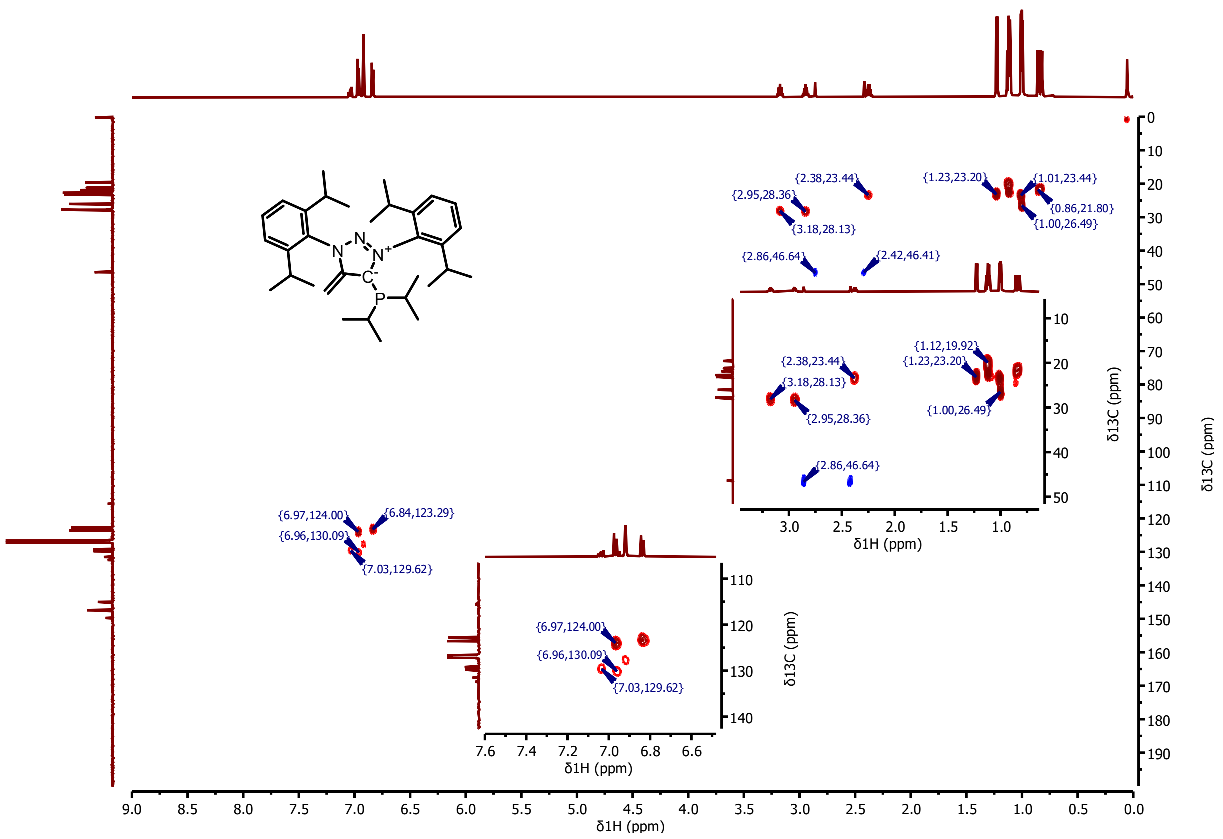

Fig S23: ^{1}H/^{13}C HSQC (600/150 MHz, C_6D_6, 298K) of **4.**

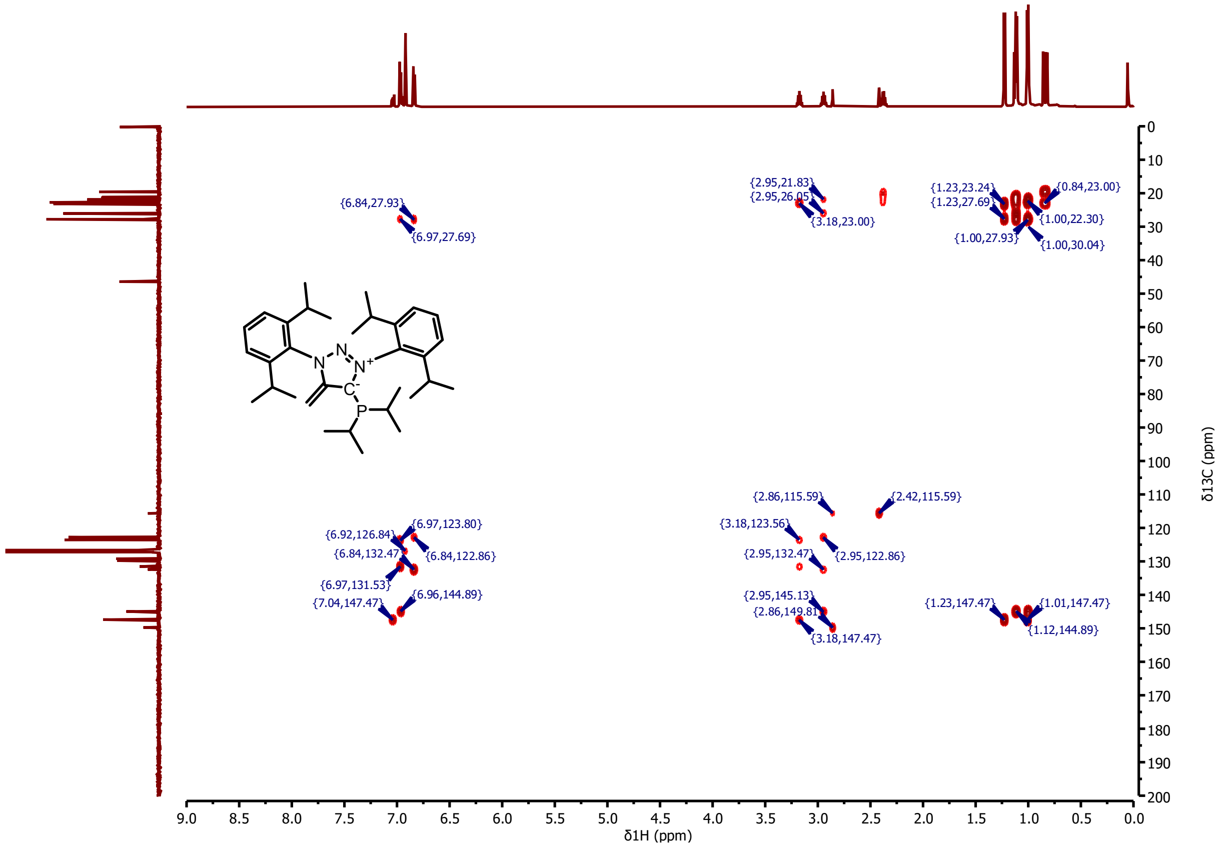

Fig S24: ^{1}H/^{13}C HMBC (600/150 MHz, C_6D_6, 298K) of **4.**

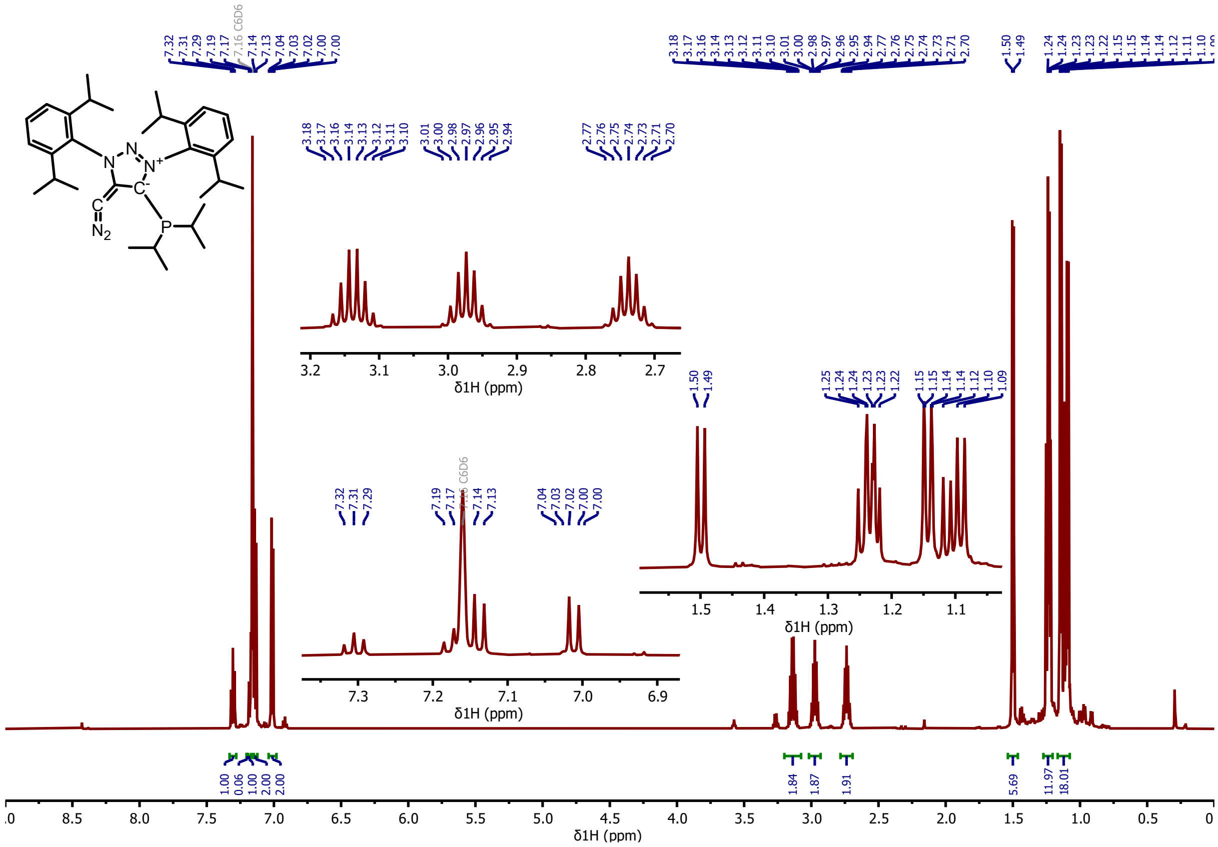

Fig S25: ^{1}**H NMR** (600 MHz, C_6D_6, 298K) of **5.**

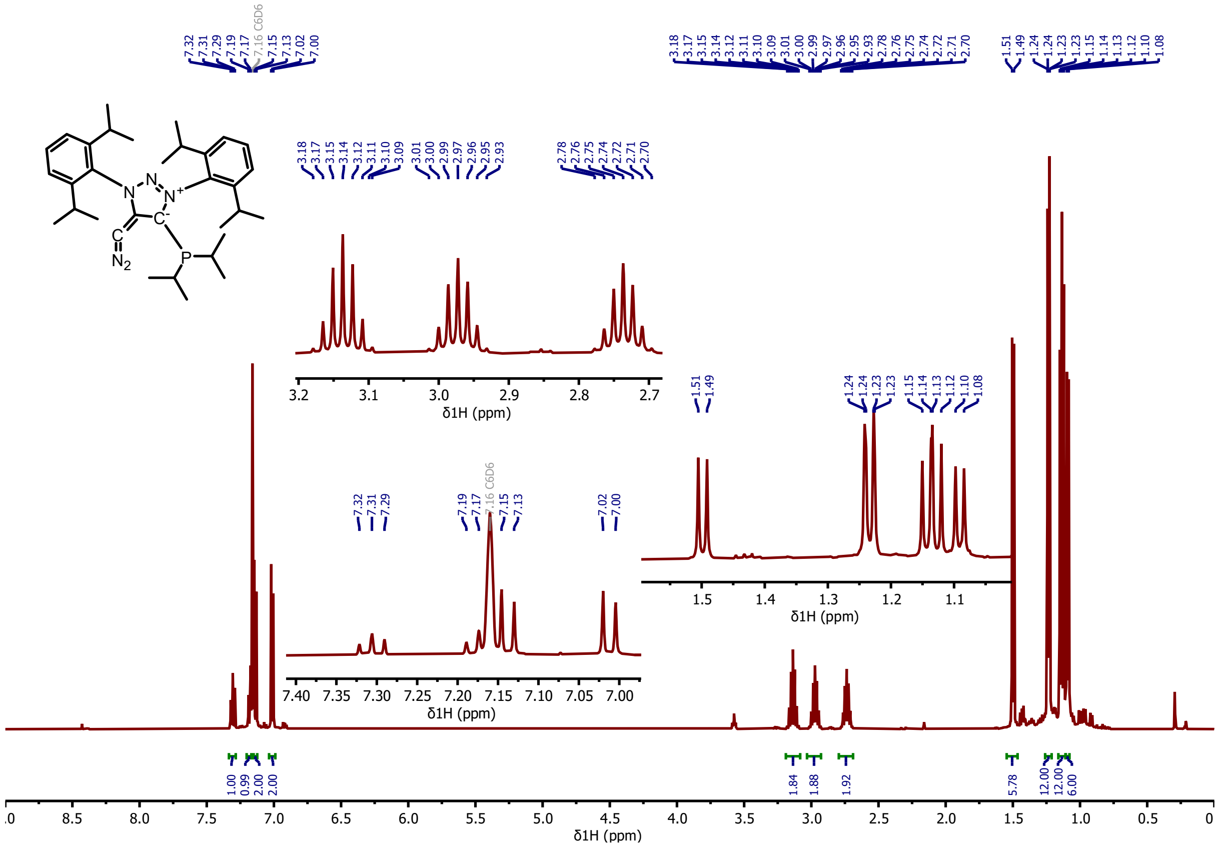

Fig S26: ^{1}**H NMR {**^{31}**P}** (500 MHz, C_6D_6, 298K) of **5.**

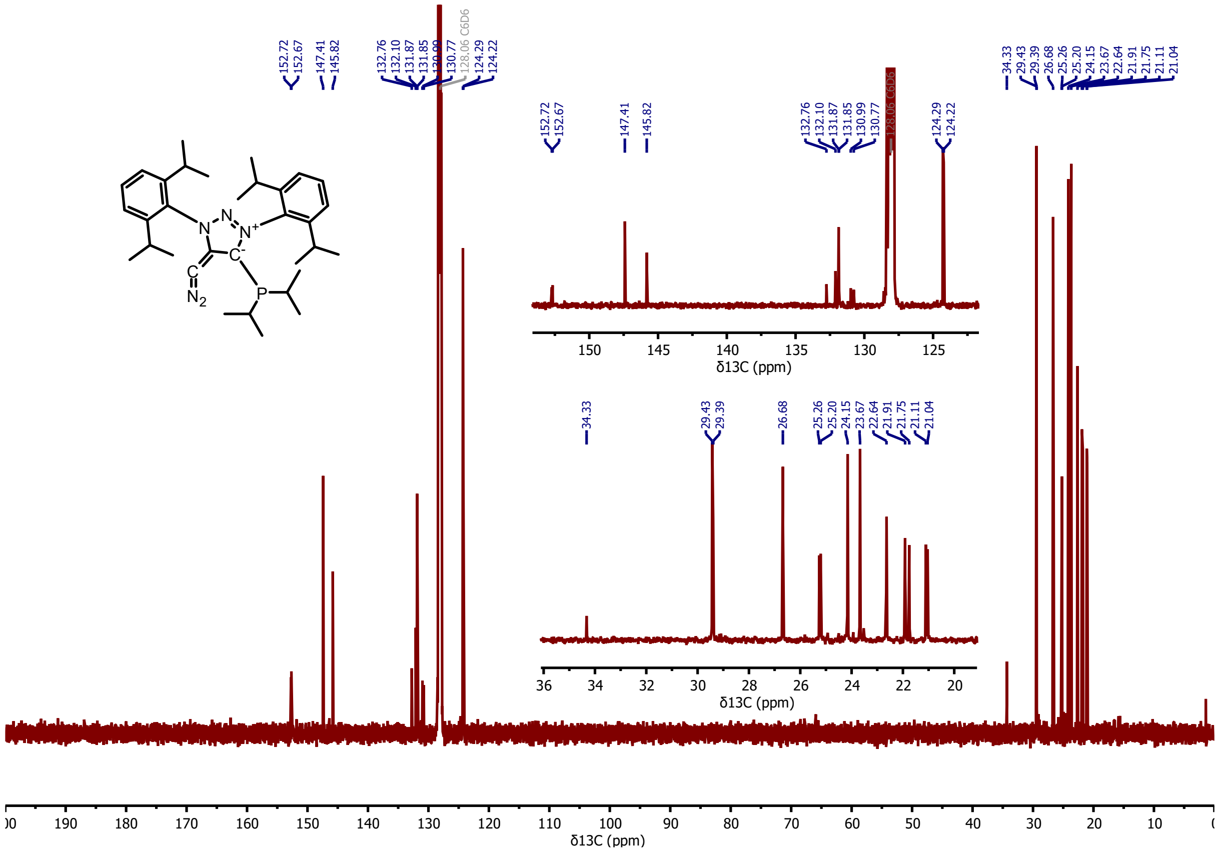

Fig S27: ^{13}**C NMR {**^{1}**H}** (150 MHz, C_6D_6, 298K) of **5.**

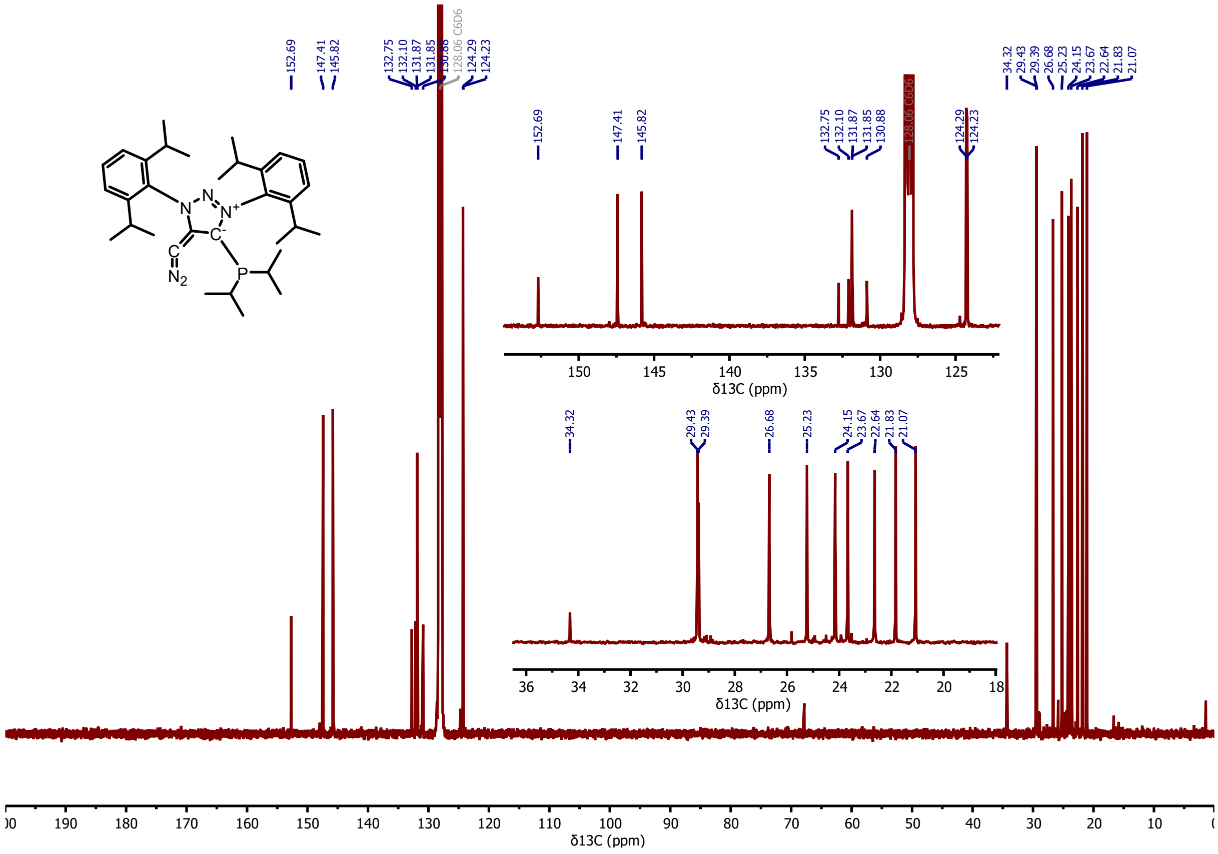

Fig S28: ^{13}**C NMR {**^{1}**H;**^{31}**P}** (125 MHz, C_6D_6, 298K) of **5.**

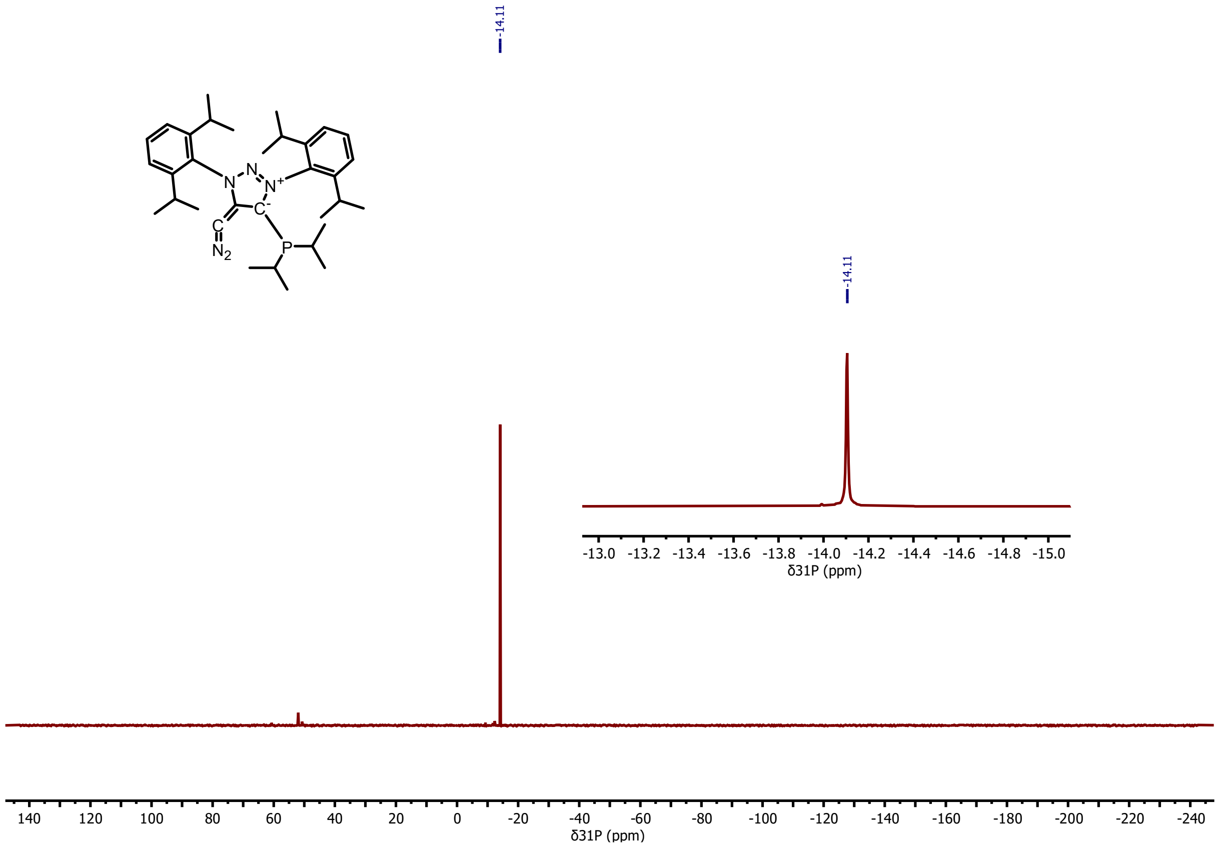

Fig S29: ^{31}**P NMR** (243 MHz, C_6D_6; 298 K) of **5.**

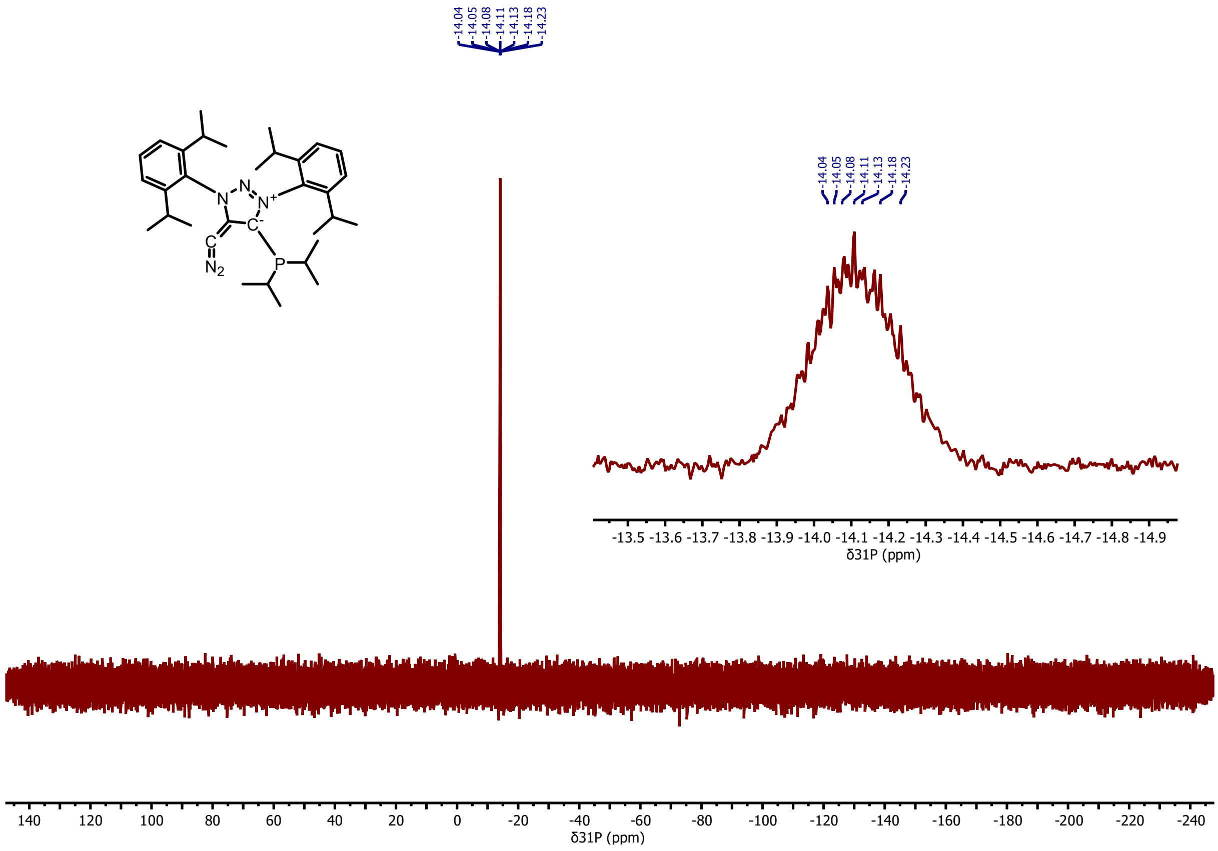

Fig S30: ^{31}**P NMR {^{1}H}** (243 MHz, C_6D_6; 298 K) of **5.**

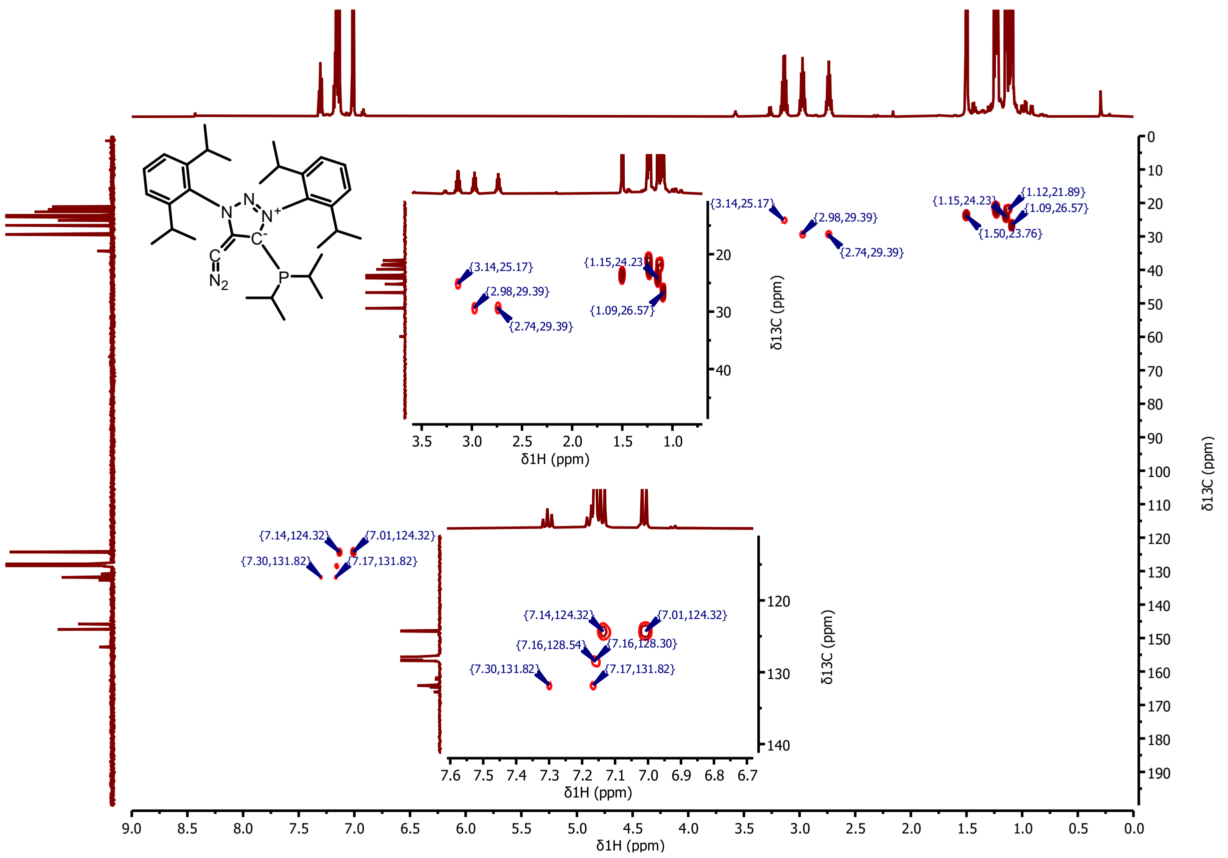

Fig S31: $^{1}H/^{13}C$ HSQC (600/150 MHz, C_6D_6, 298K) of **5.**

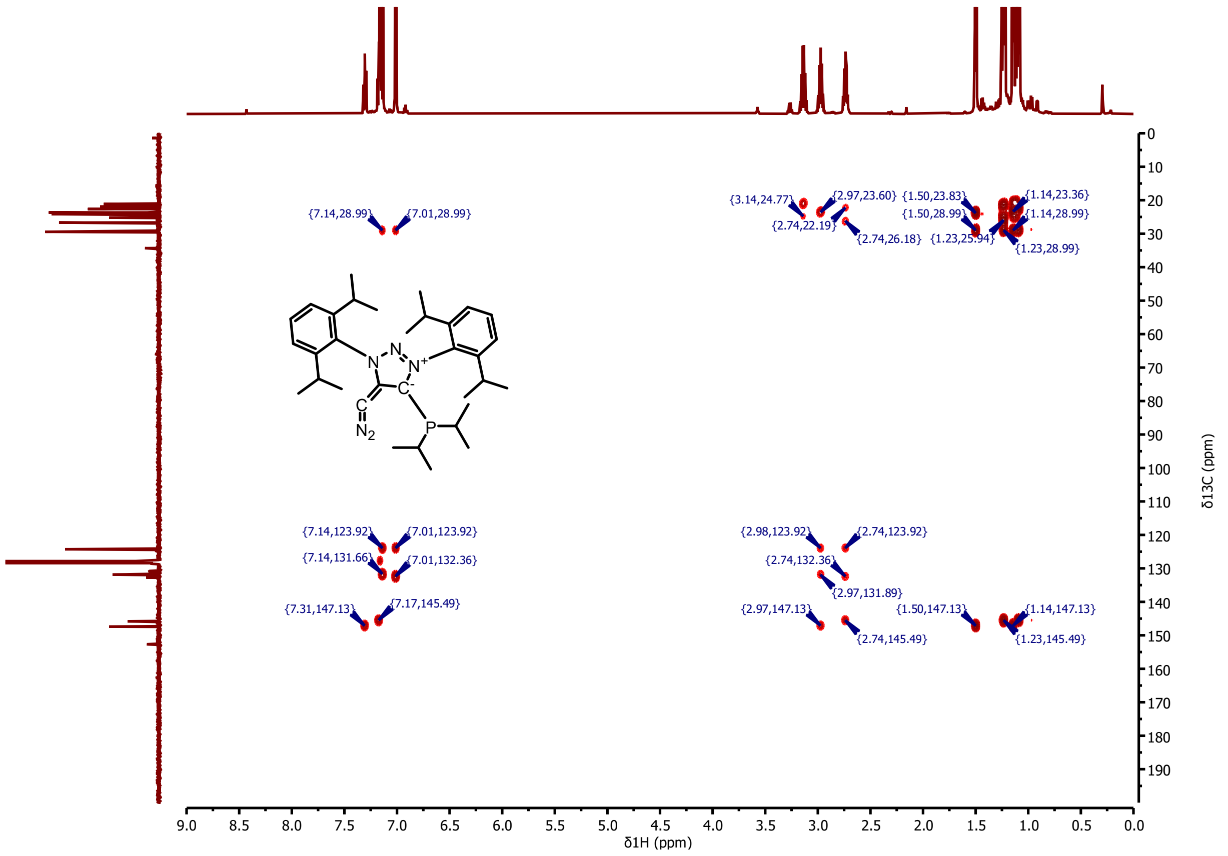

Fig S32: $^{1}H/^{13}C$ HMBC (600/150 MHz, C_6D_6, 298K) of **5.**

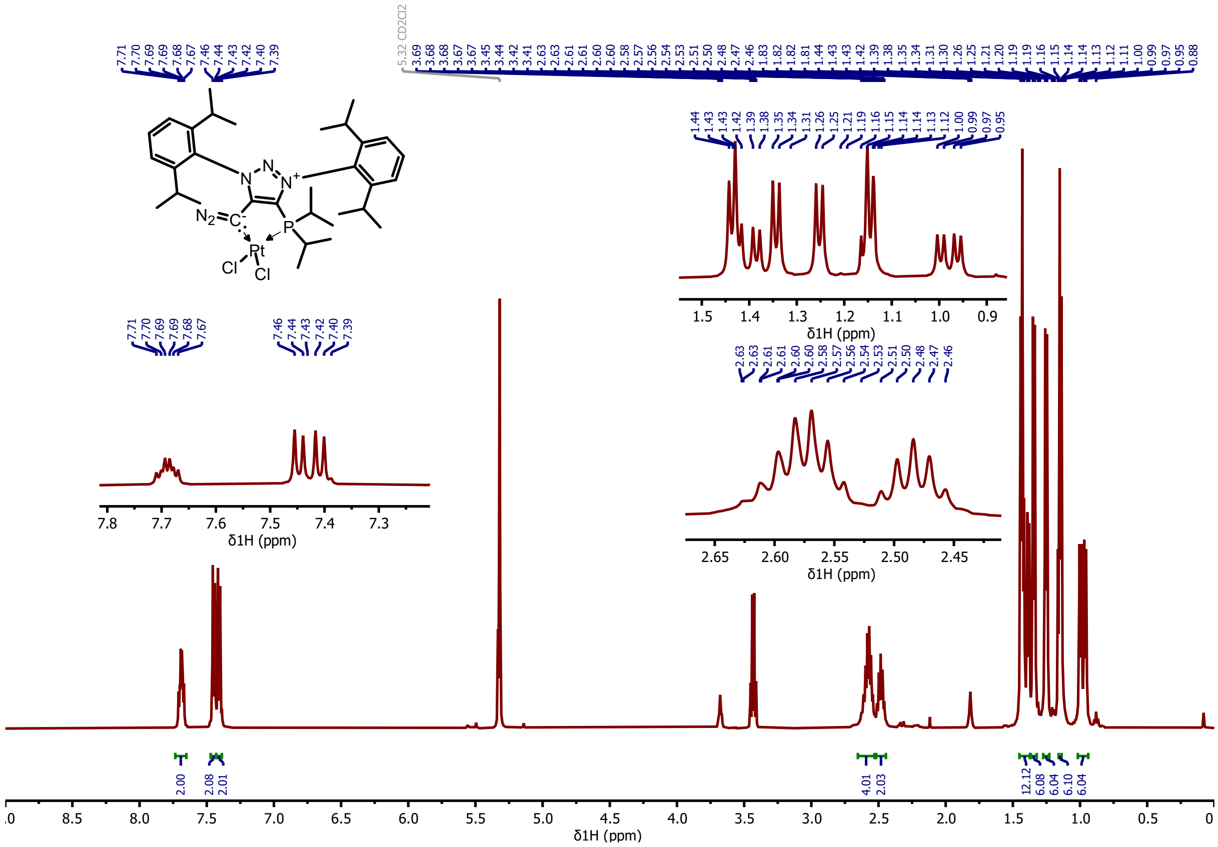

Fig S33: **^{1}H NMR** (500 MHz, CD_2Cl_2, 298K) of **6.**

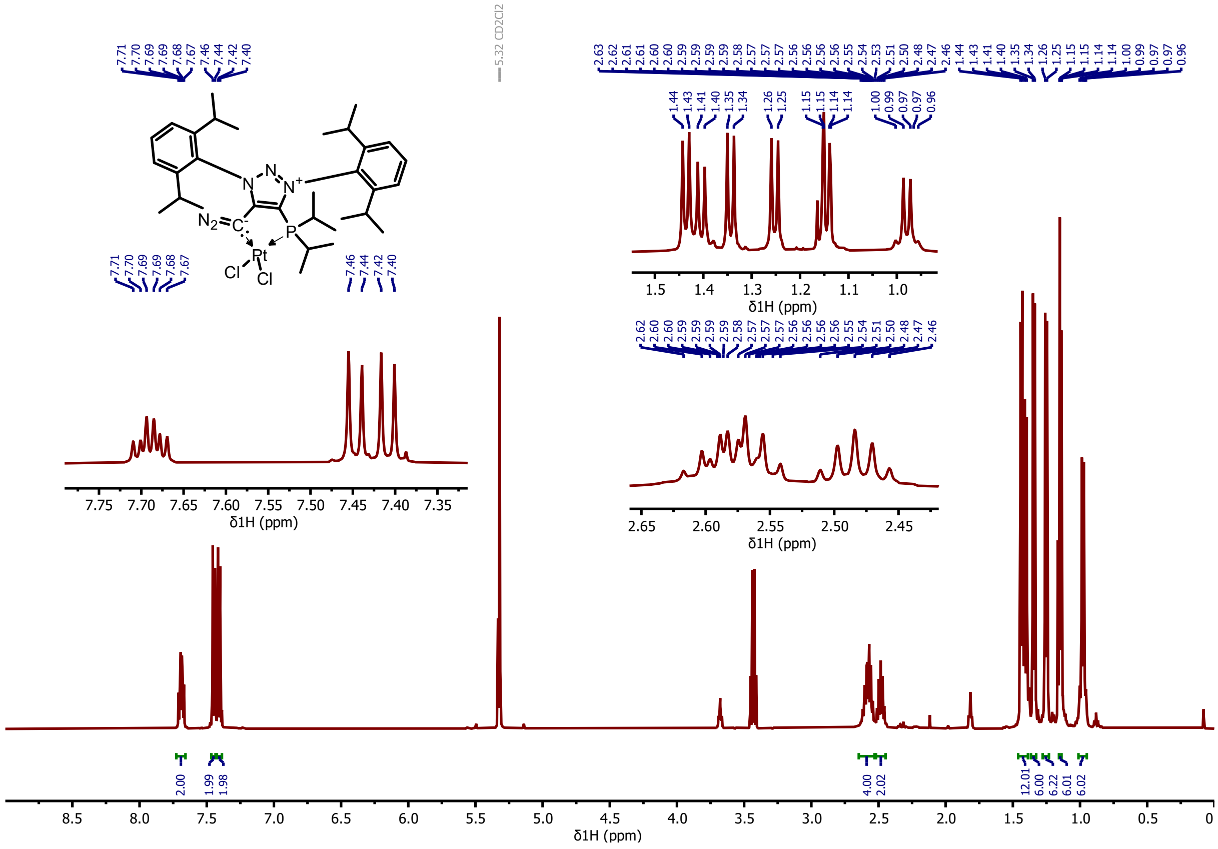

Fig S34: **^{1}H NMR {^{31}P}** (500 MHz, CD_2Cl_2, 298K) of **6.**

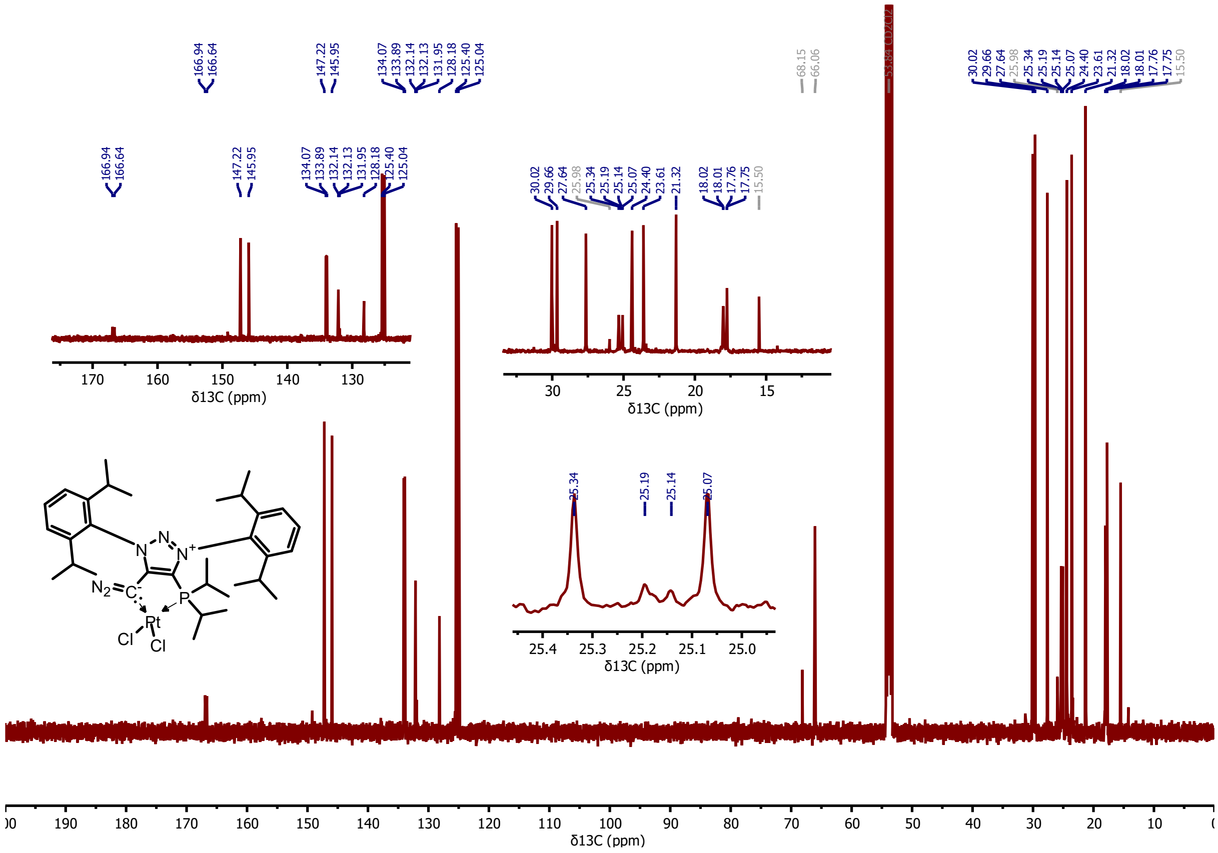

Fig S35: ^{13}C NMR {^{1}H} (125 MHz, CD_2Cl_2, 298K) of **6.**

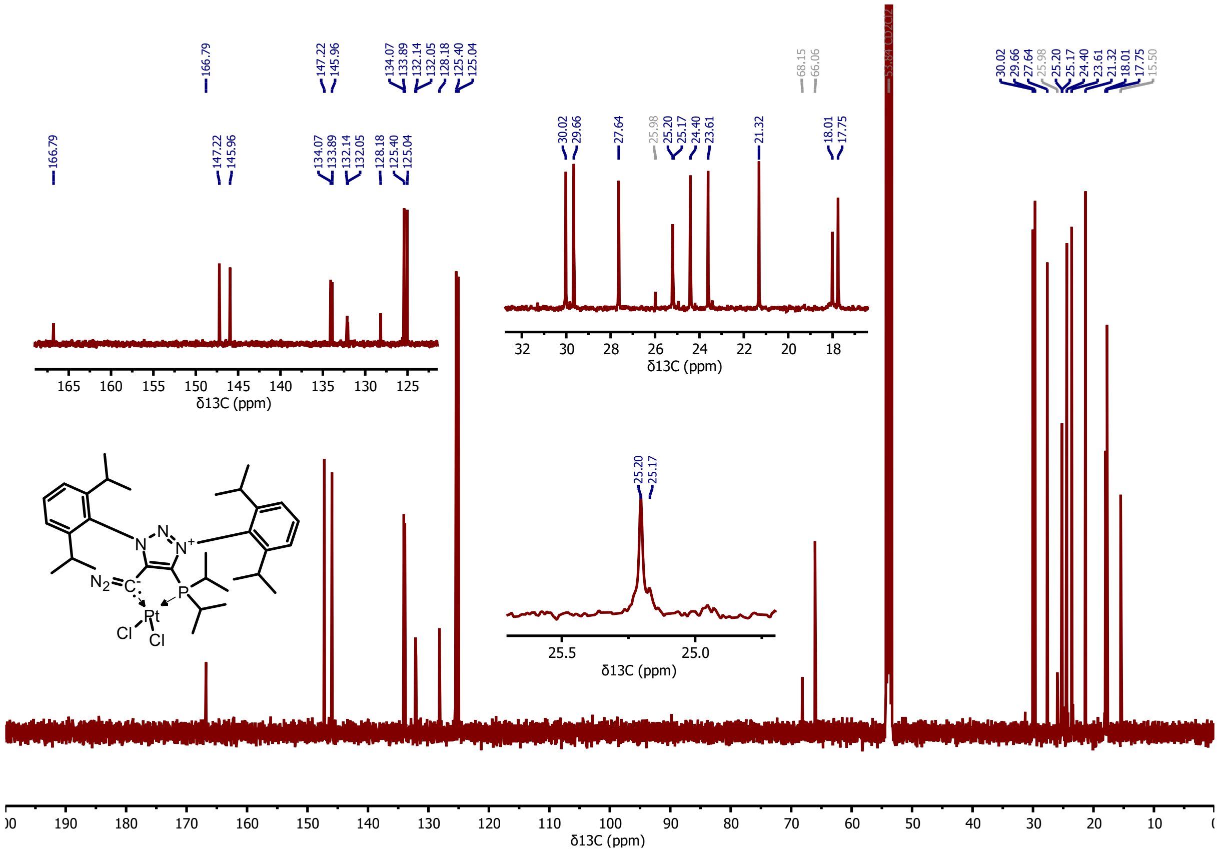

Fig S36: ^{13}C NMR {^{1}H; ^{31}P} (125 MHz, CD_2Cl_2, 298K) of **6.**

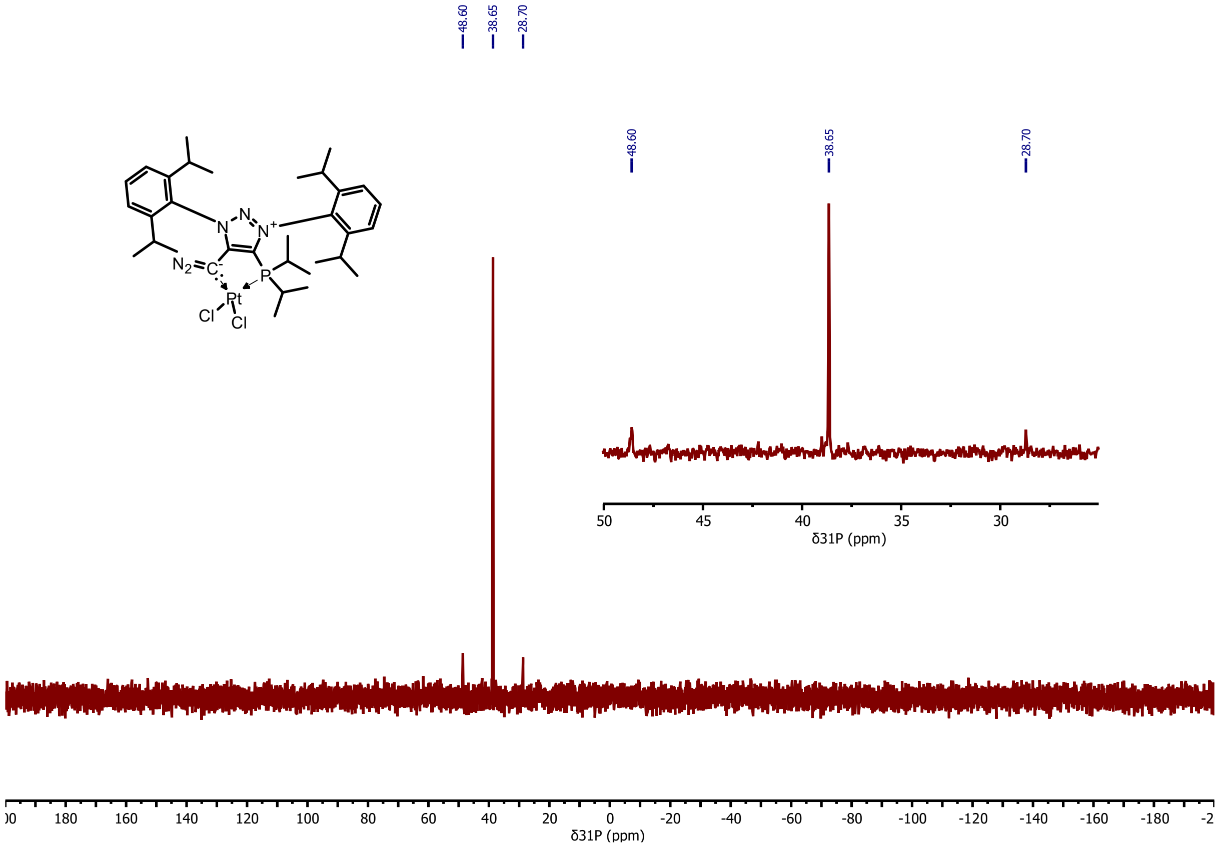

Fig S37: ^{31}P NMR {^{1}H} (203 MHz, CD_2Cl_2, 298K) of **6.**

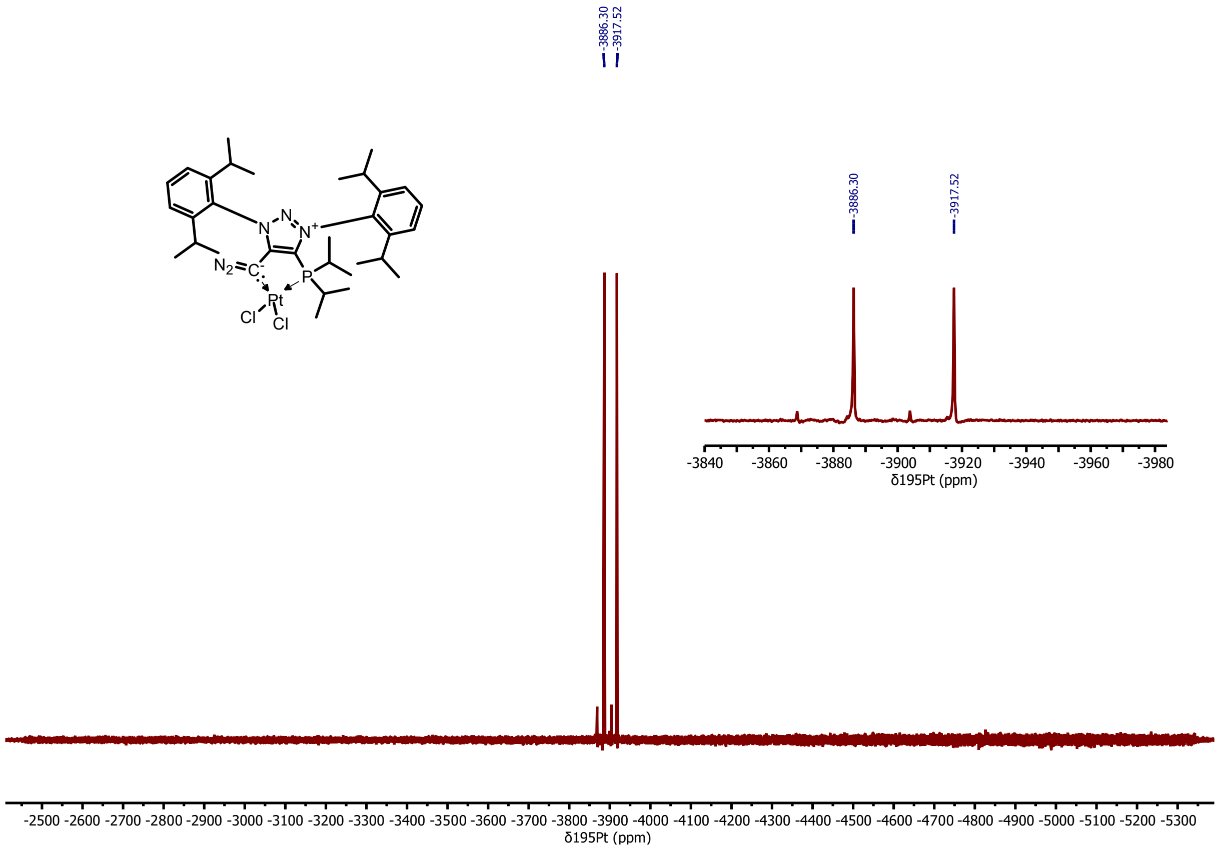

Fig S38: ^{195}Pt NMR (129 MHz, CD_2Cl_2, 298K) of **6.**

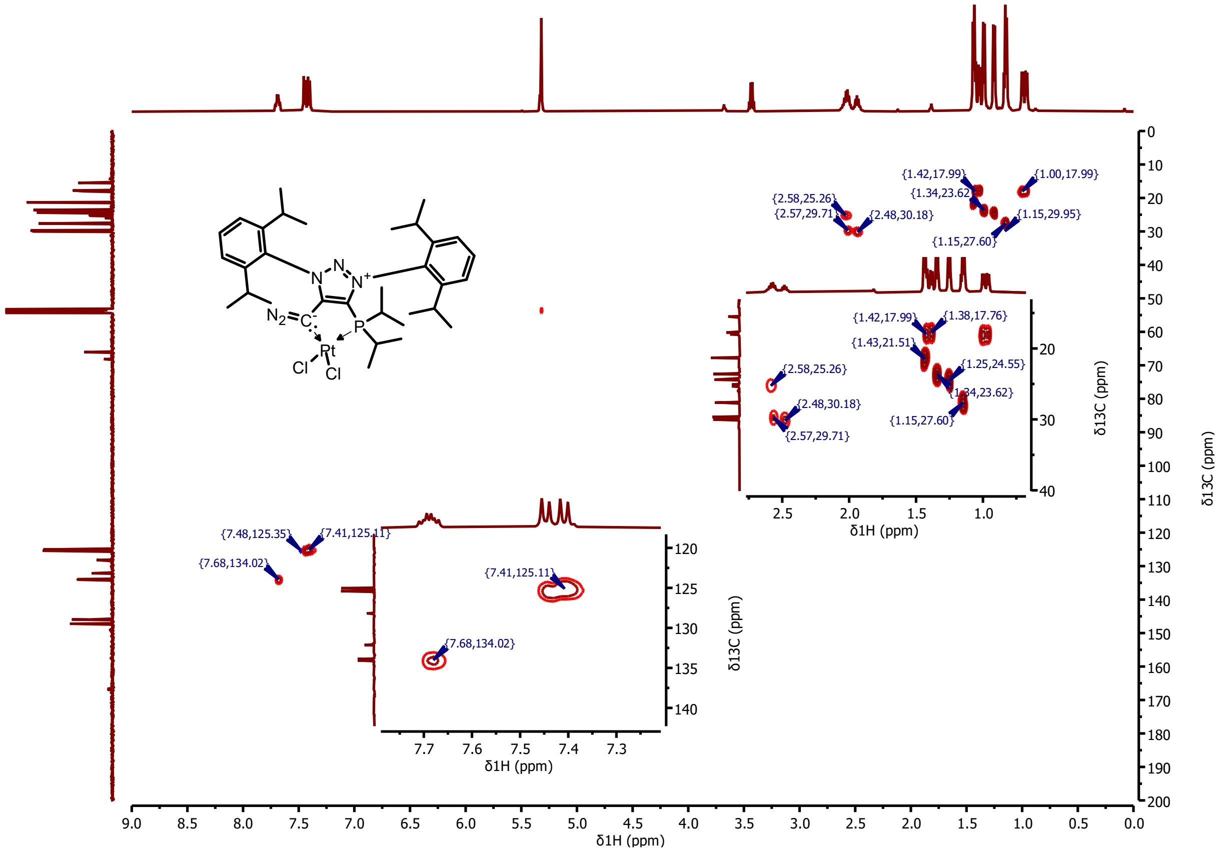

Fig S39: $^{1}H/^{13}C$ HSQC (500/125 MHz, CD_2Cl_2, 298K) of **6.**

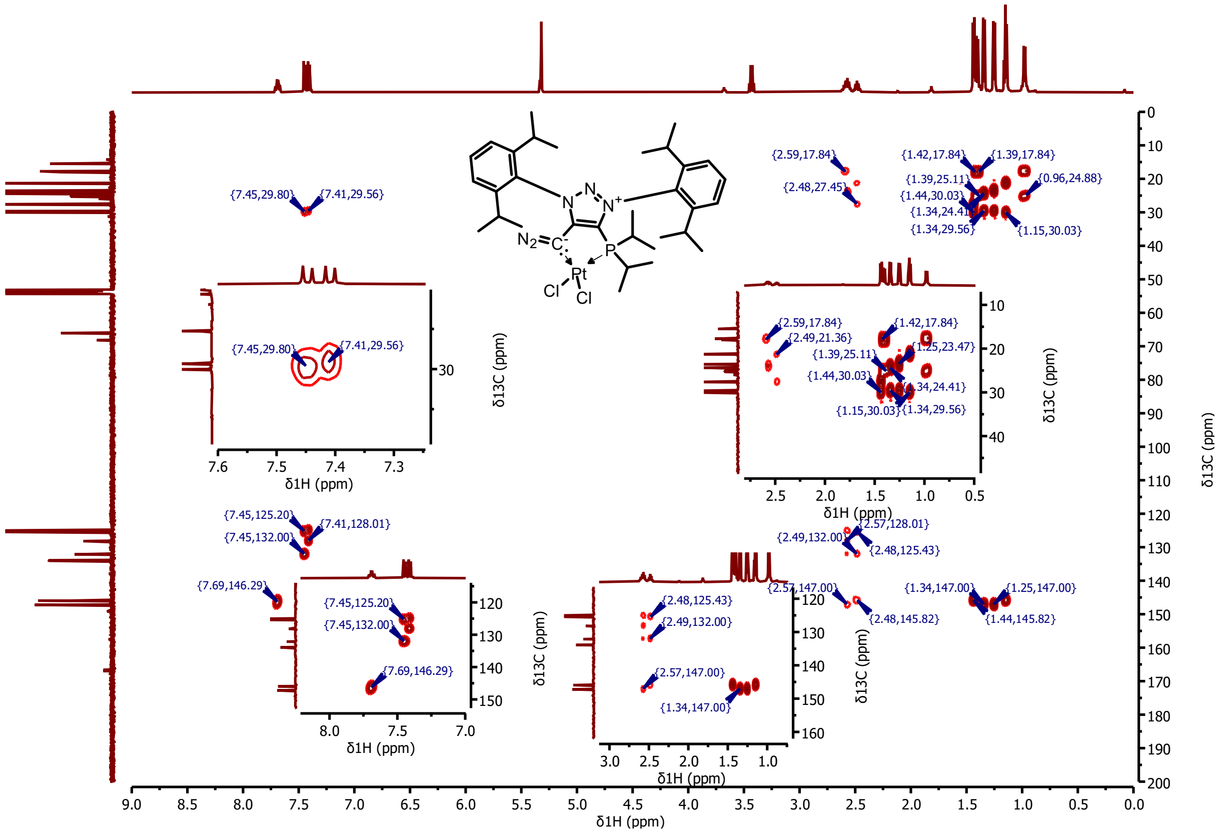

Fig S40: $^{1}H/^{13}C$ HMBC (500/125 MHz, CD_2Cl_2, 298K) of **6.**

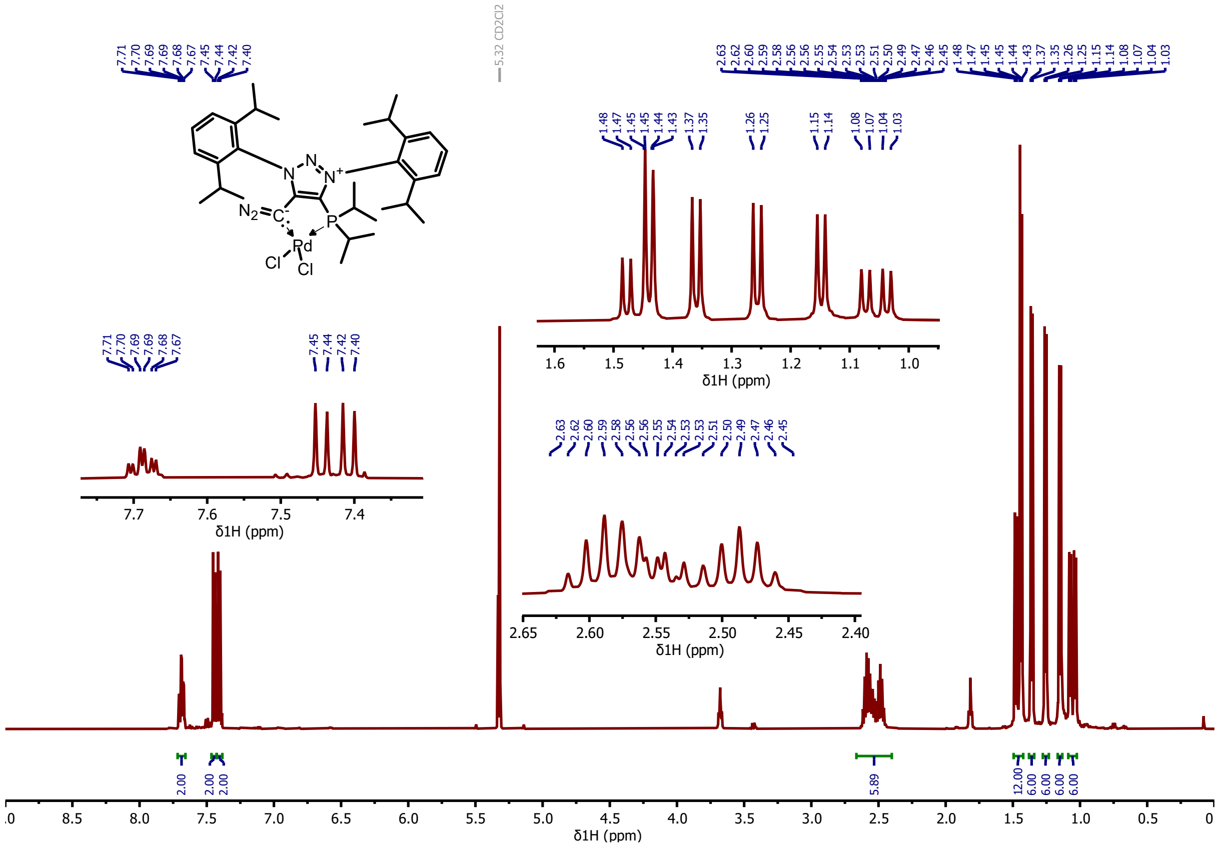

Fig S41: ^{1}H NMR (500 MHz, CD_2Cl_2, 298K) of **7.**

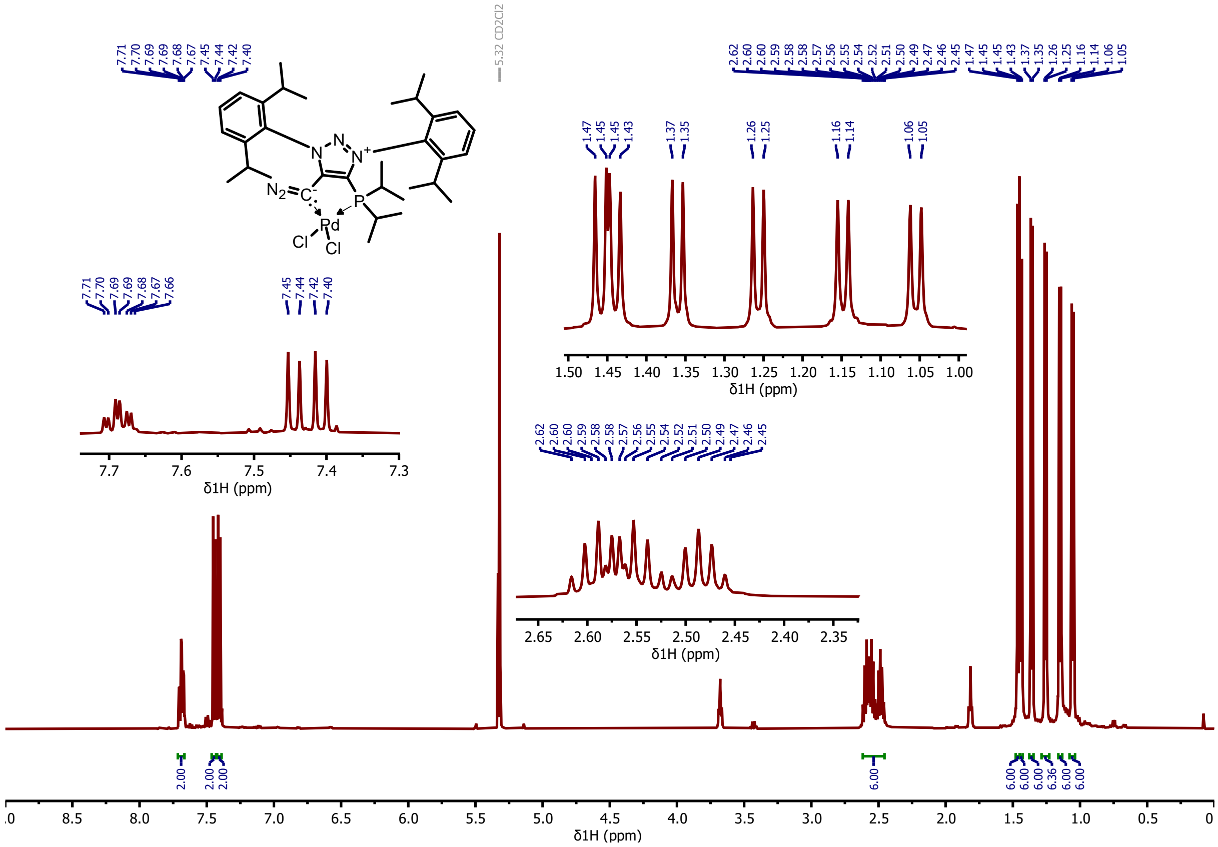

Fig S42: ^{1}H NMR {^{31}P} (500 MHz, CD_2Cl_2, 298K) of **7.**

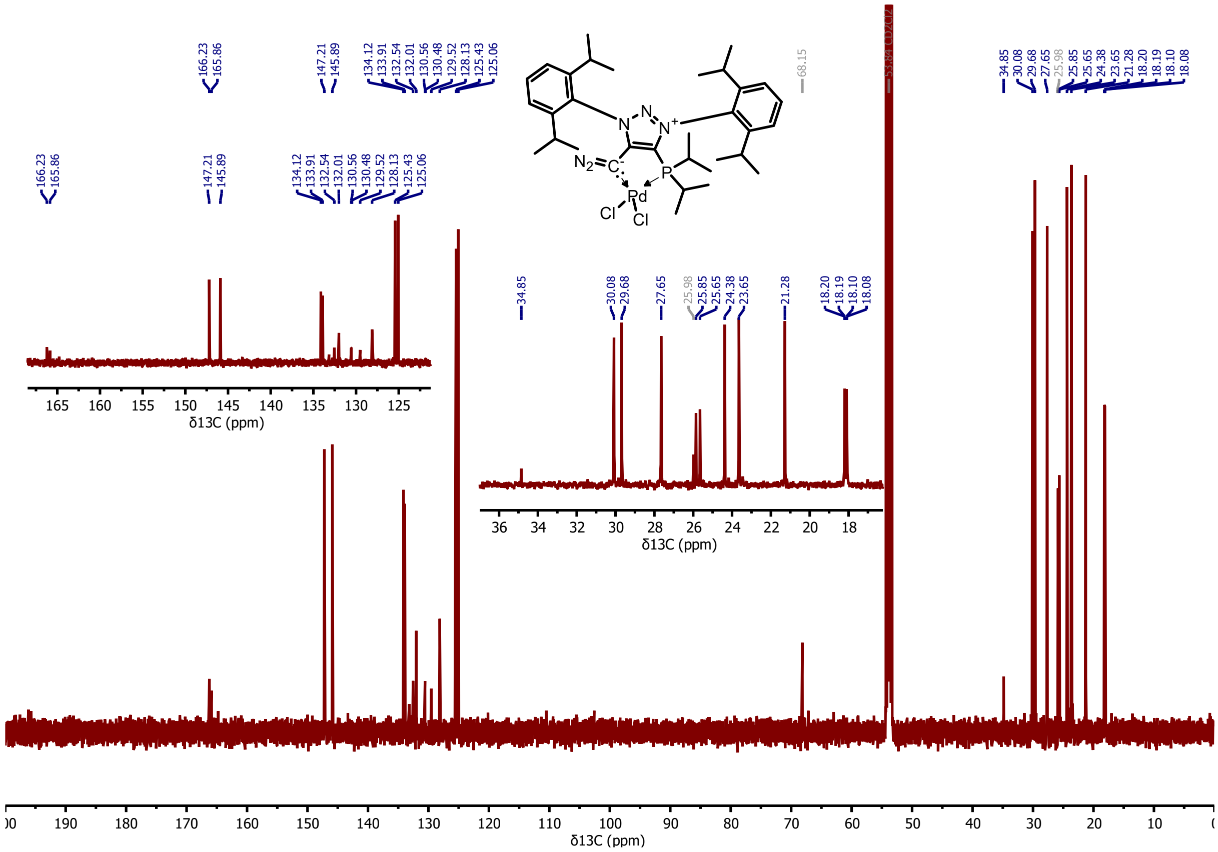

Fig S43: ^{13}**C NMR {**^{1}**H}** (125 MHz, CD_2Cl_2, 298K) of **7.**

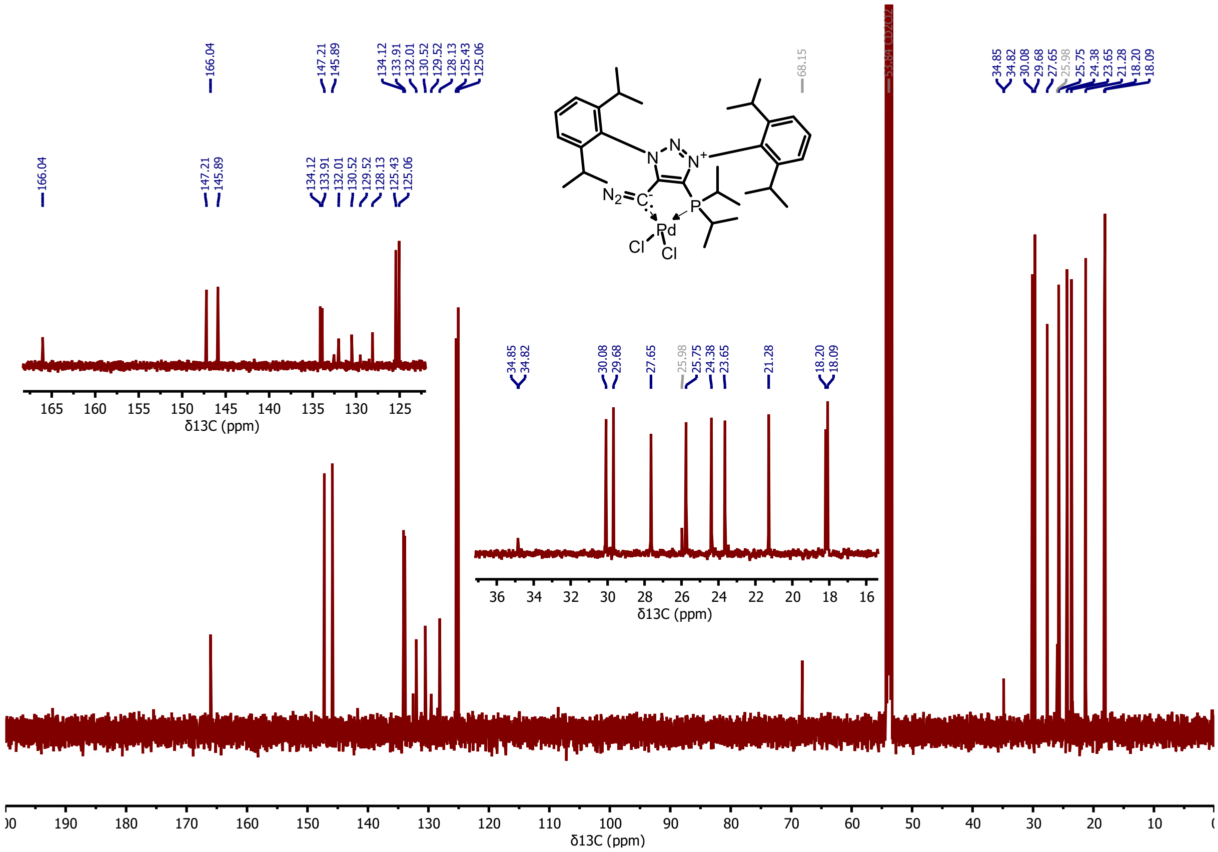

Fig S44: ^{13}**C NMR {**^{1}**H;** ^{31}**P}** (125 MHz, CD_2Cl_2, 298K) of **7.**

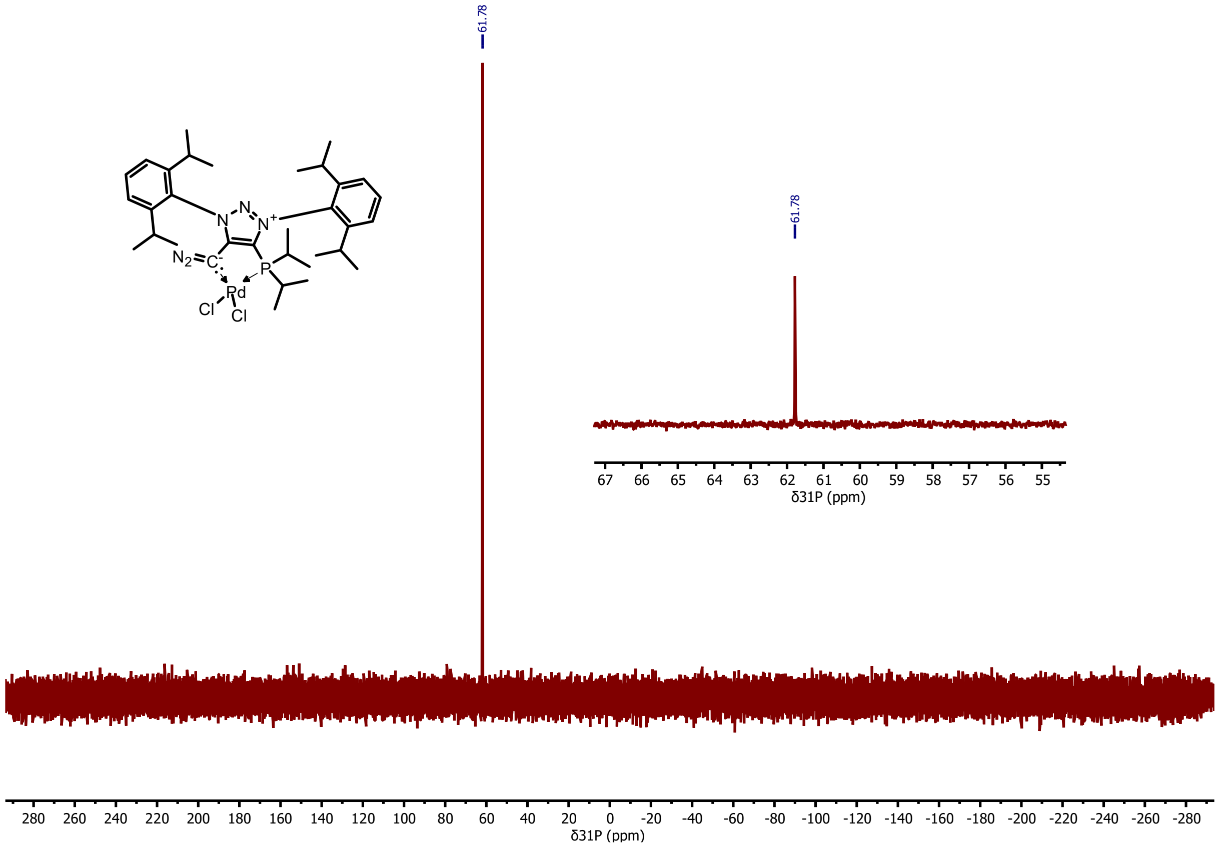

Fig S45: ^{31}P NMR {^{1}H} (203 MHz, CD_2Cl_2, 298K) of **7.**

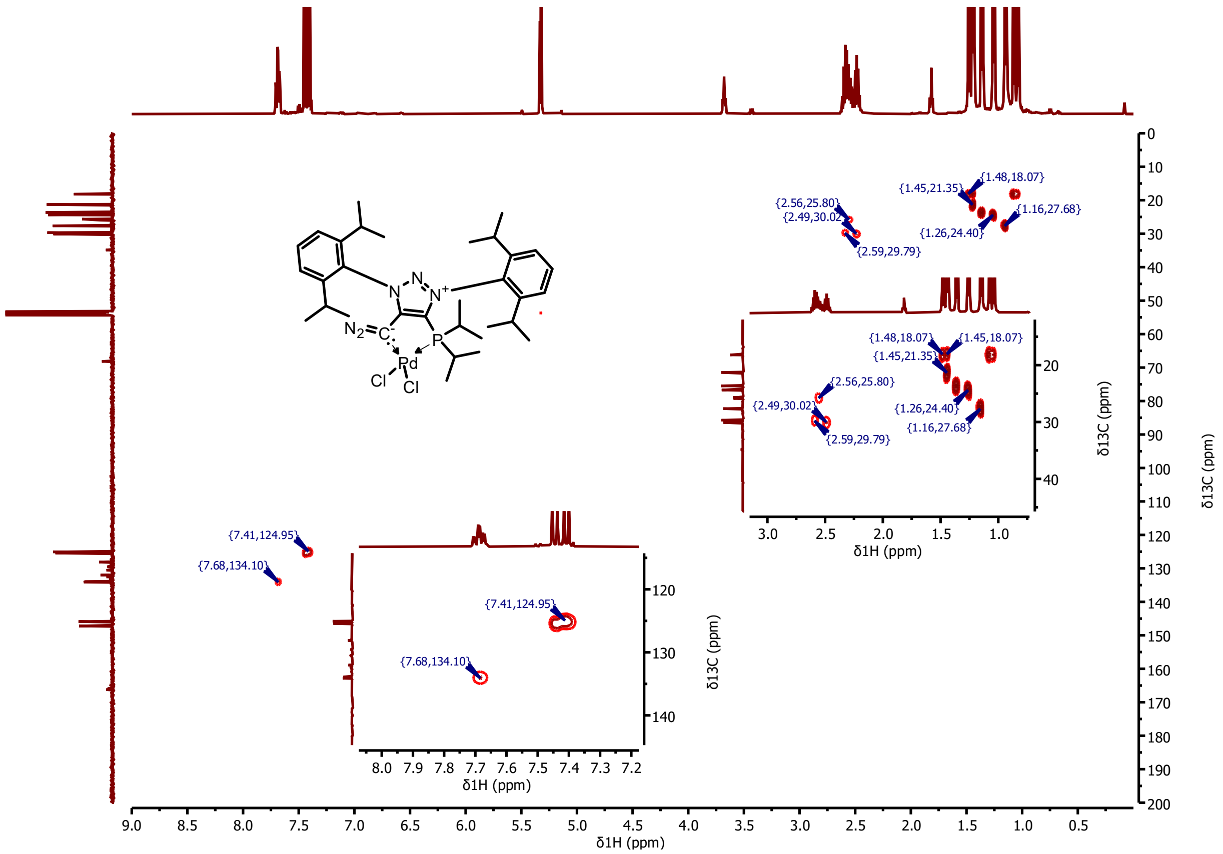

Fig S46: ^{1}H/^{13}C HSQC (500/125 MHz, CD_2Cl_2, 298K) of **7.**

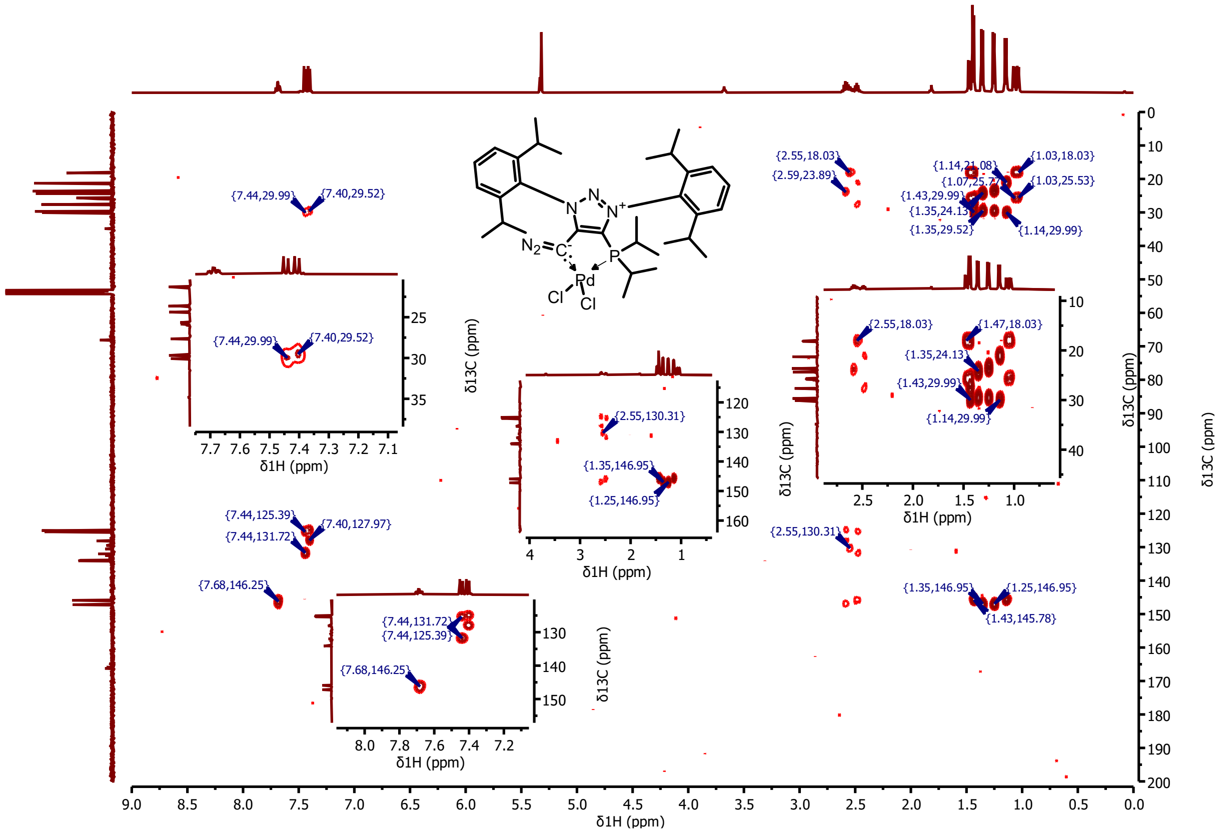

Fig S47: ^{1}**H/**^{13}**C HMBC** (500/125 MHz, CD_2Cl_2, 298K) of **7.**

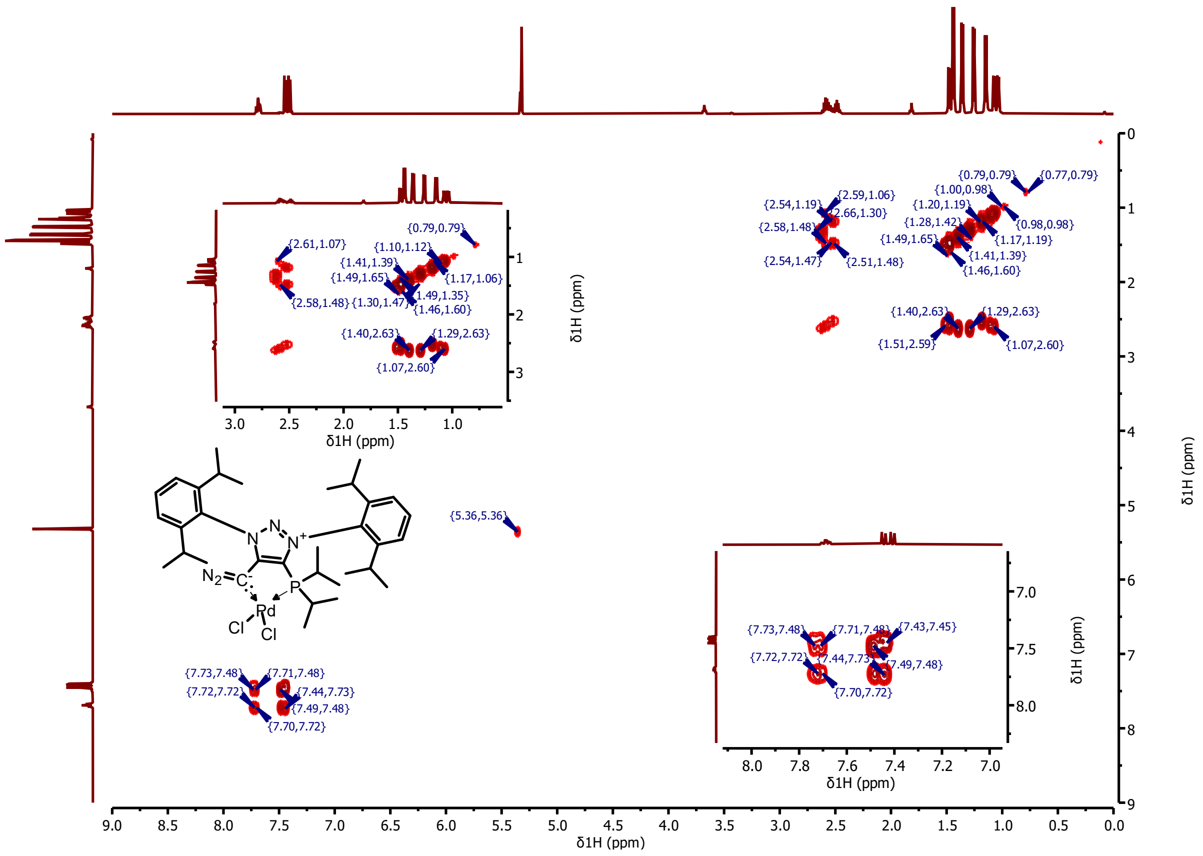

Fig S48: ^{1}**H/**^{1}**H COSY** (500/125 MHz, CD_2Cl_2, 298K) of **7.**

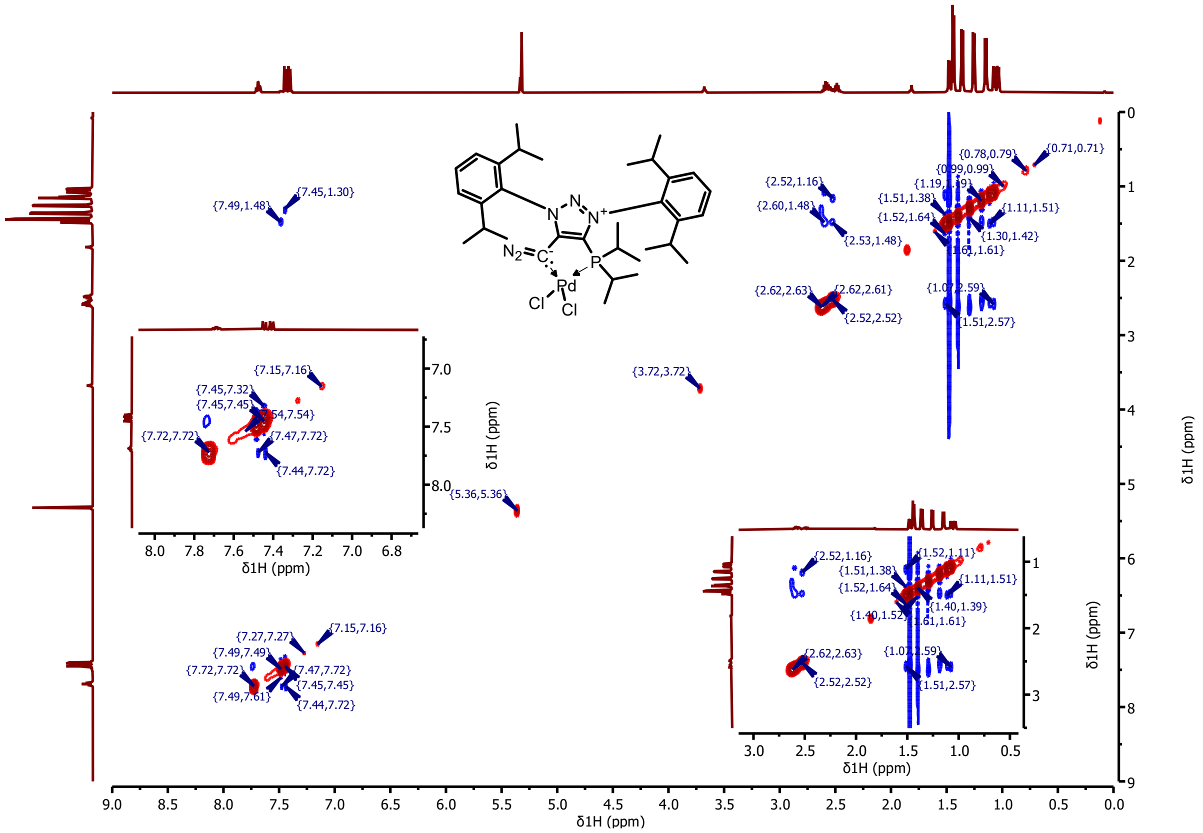

Fig S49: $^1H/^1H$ NOESY (500/125 MHz, CD_2Cl_2, 298K) of **7.**

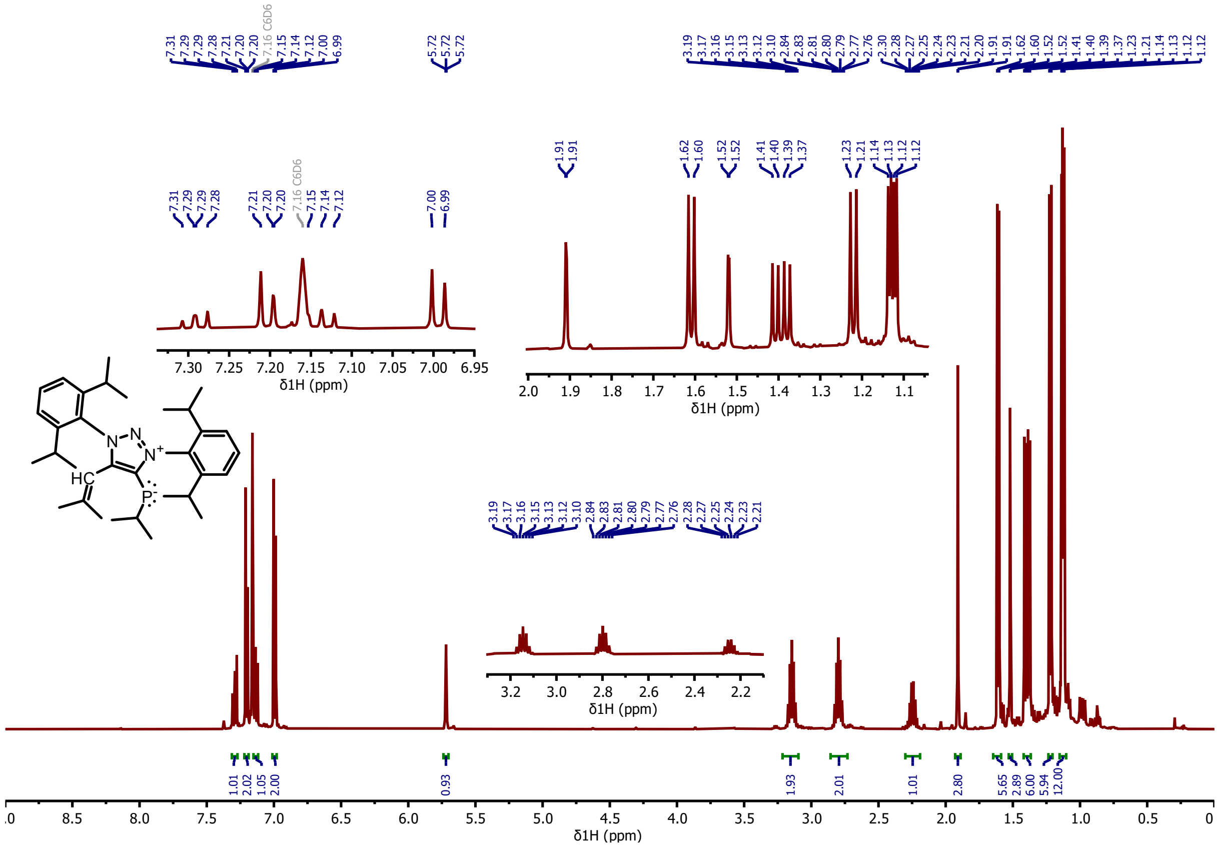

Fig S50: ^1H NMR (500 MHz, C_6D_6, 298K) of **11.**

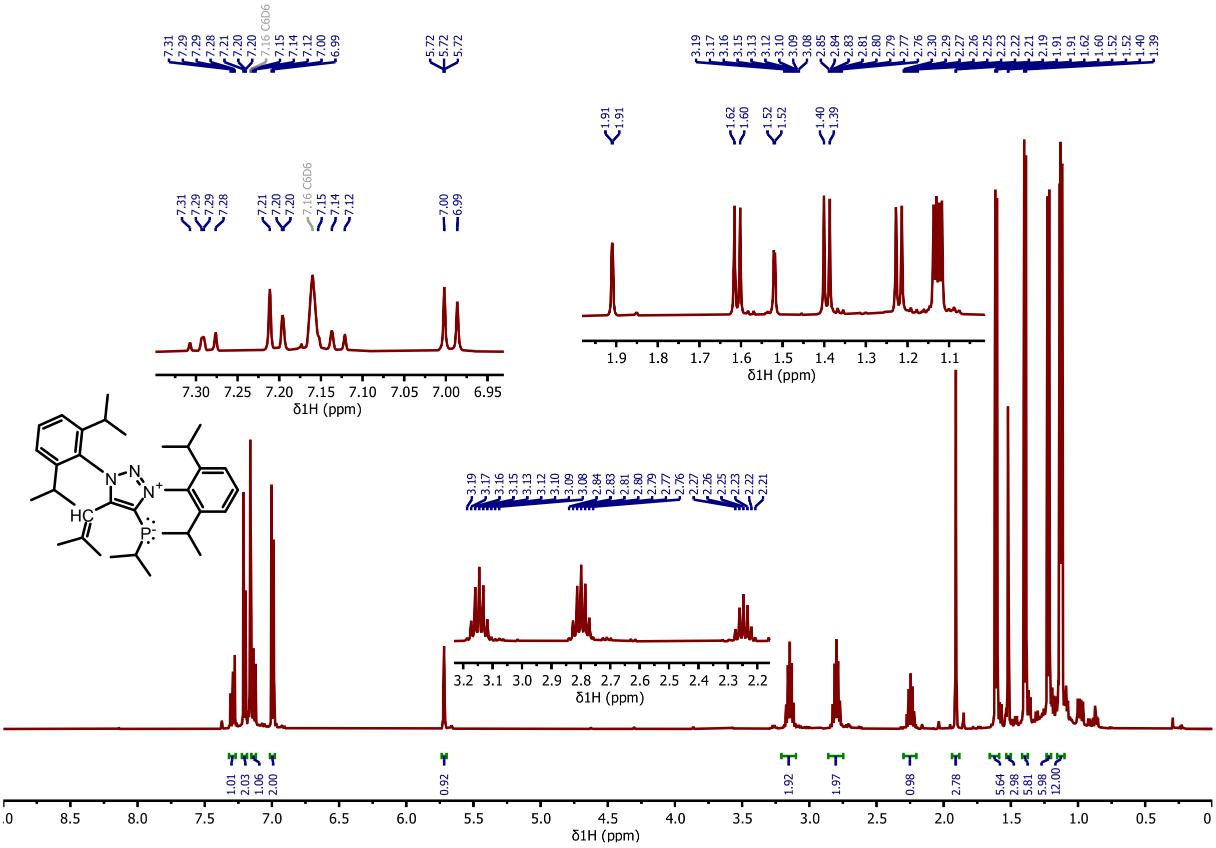

Fig S51: ^{1}**H NMR {**^{31}**P}** (600 MHz, C_6D_6, 298K) of **11.**

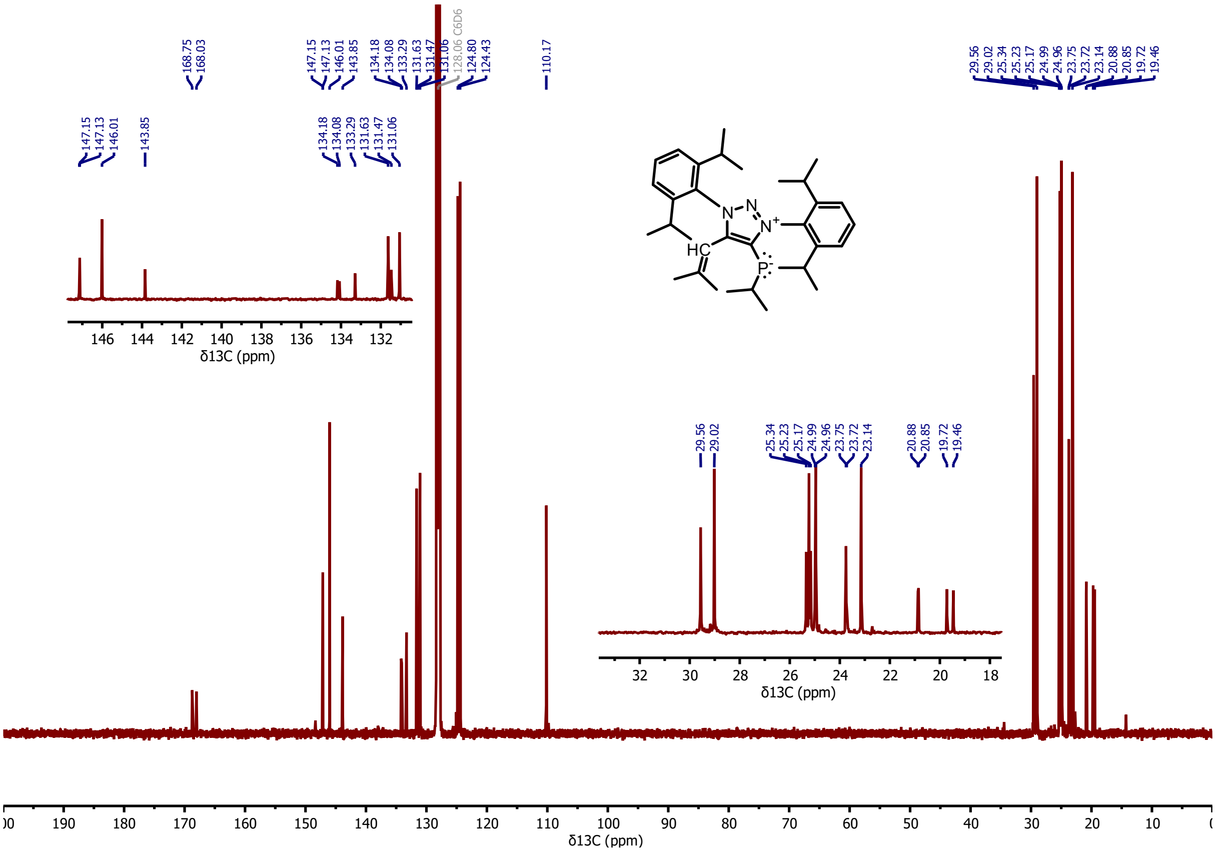

Fig S52: ^{13}**C NMR {**^{1}**H}** (126 MHz, C_6D_6, 298K) of **11.**

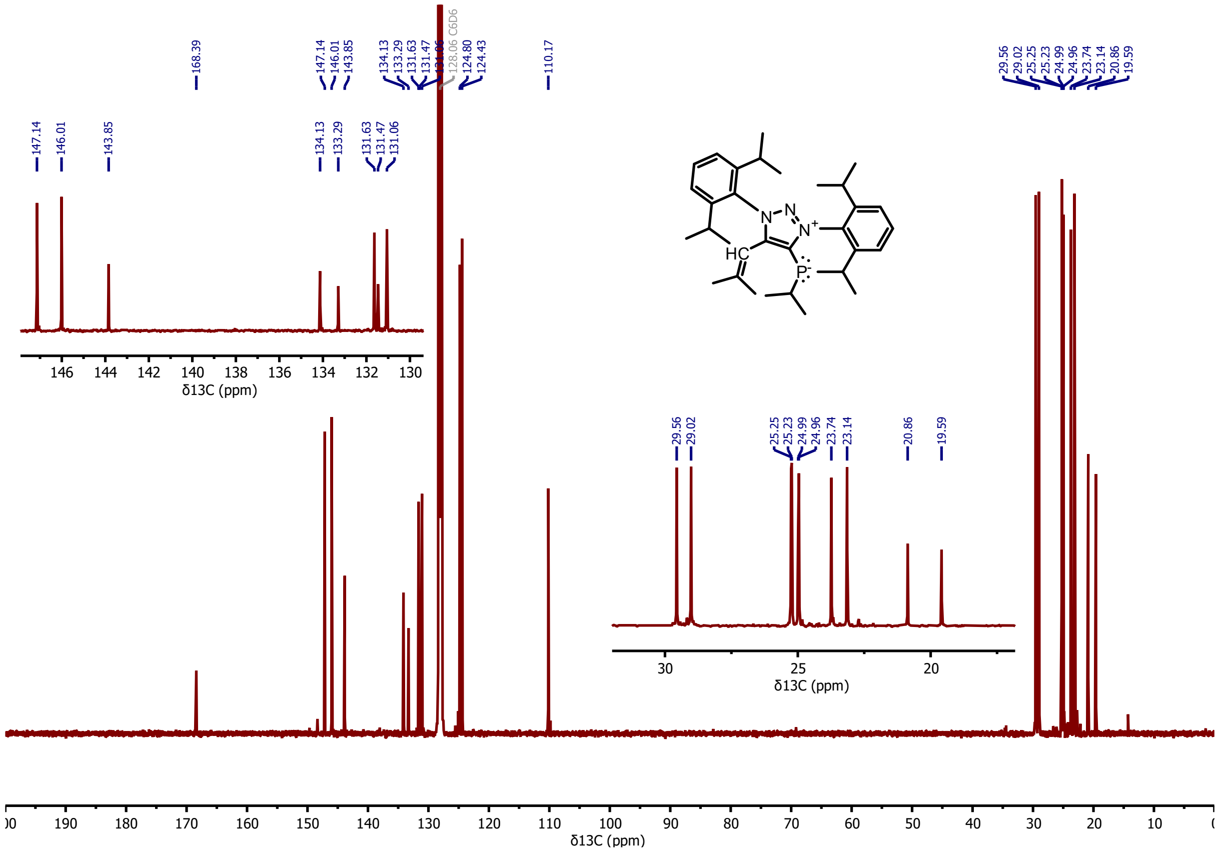

Fig S53: ^{13}C NMR {^{1}H;^{31}P} (126 MHz, C_6D_6, 298K) of **11.**

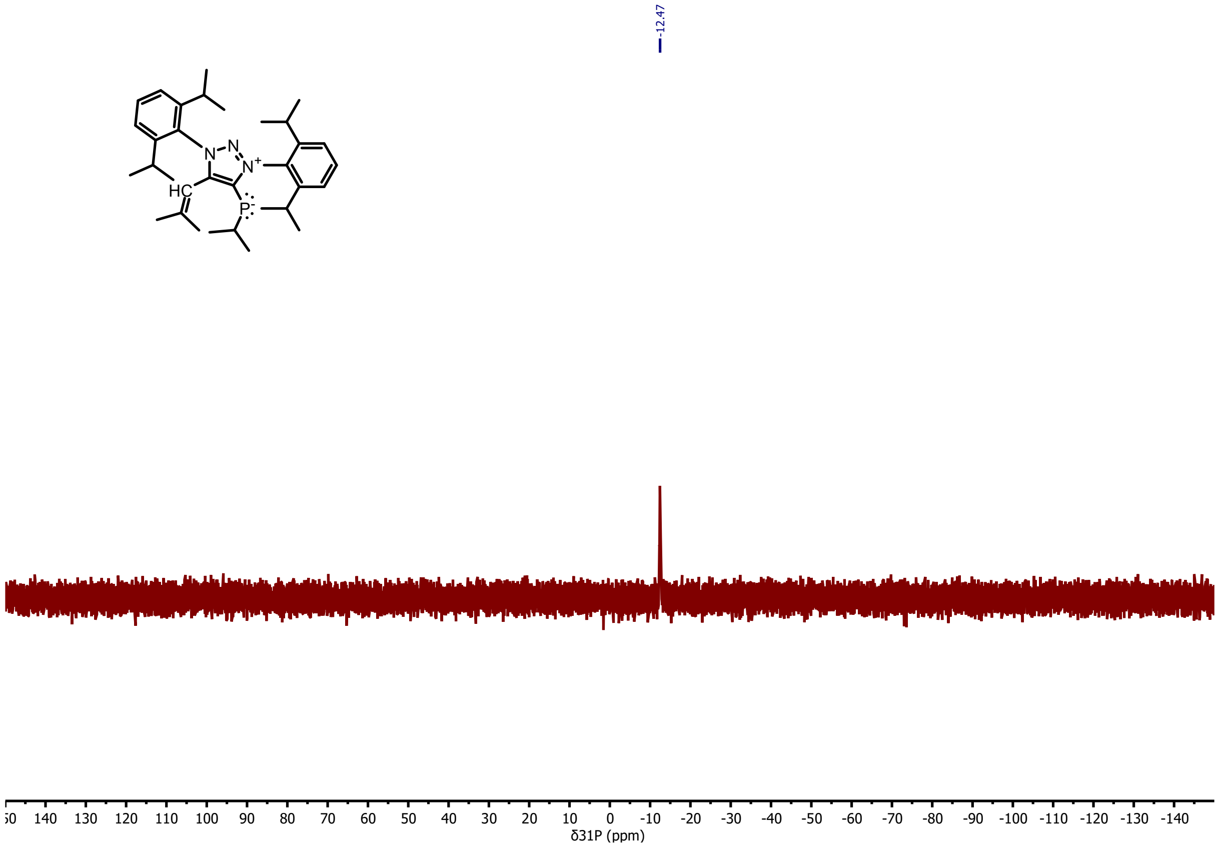

Fig S54: ^{31}P NMR {^{1}H} (203 MHz, C_6D_6, 298K) of **11.**

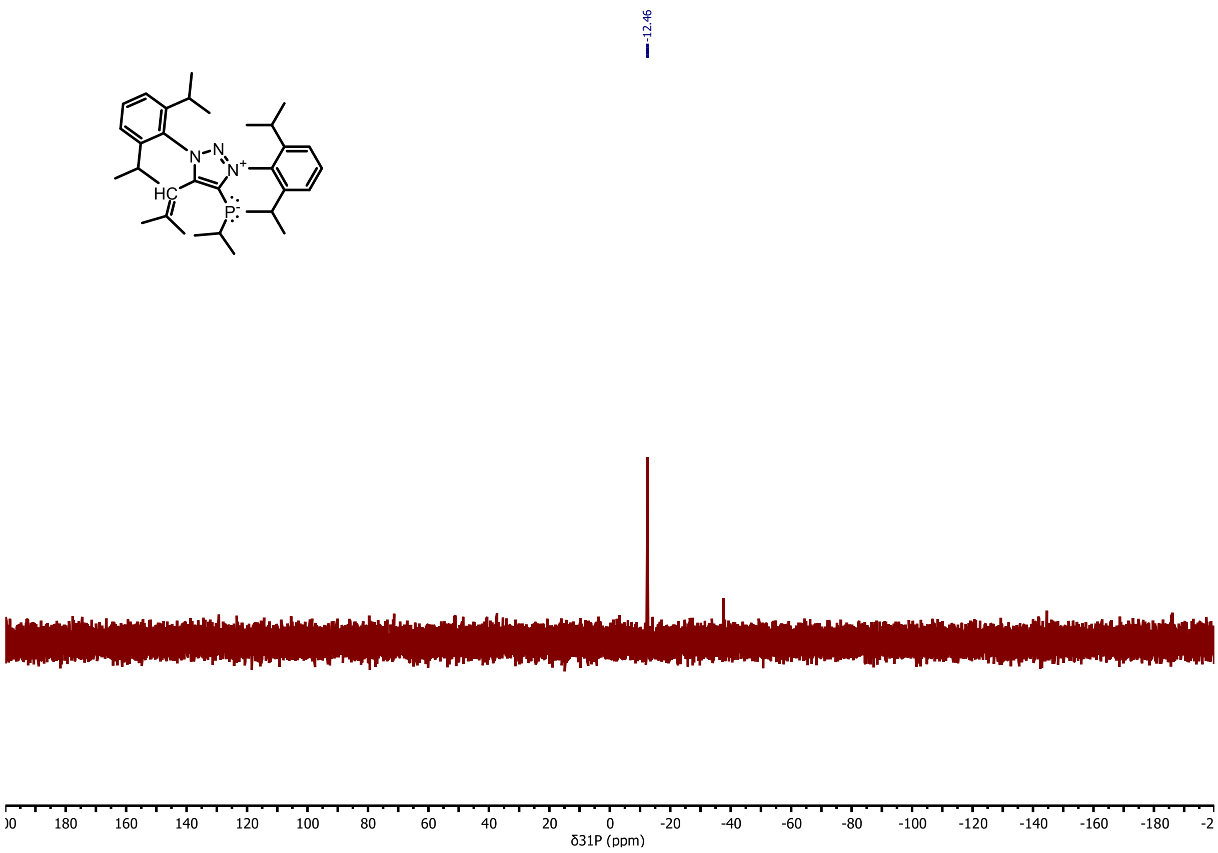

Fig S55: ^{31}P NMR (203 MHz, C_6D_6, 298K) of **11.**

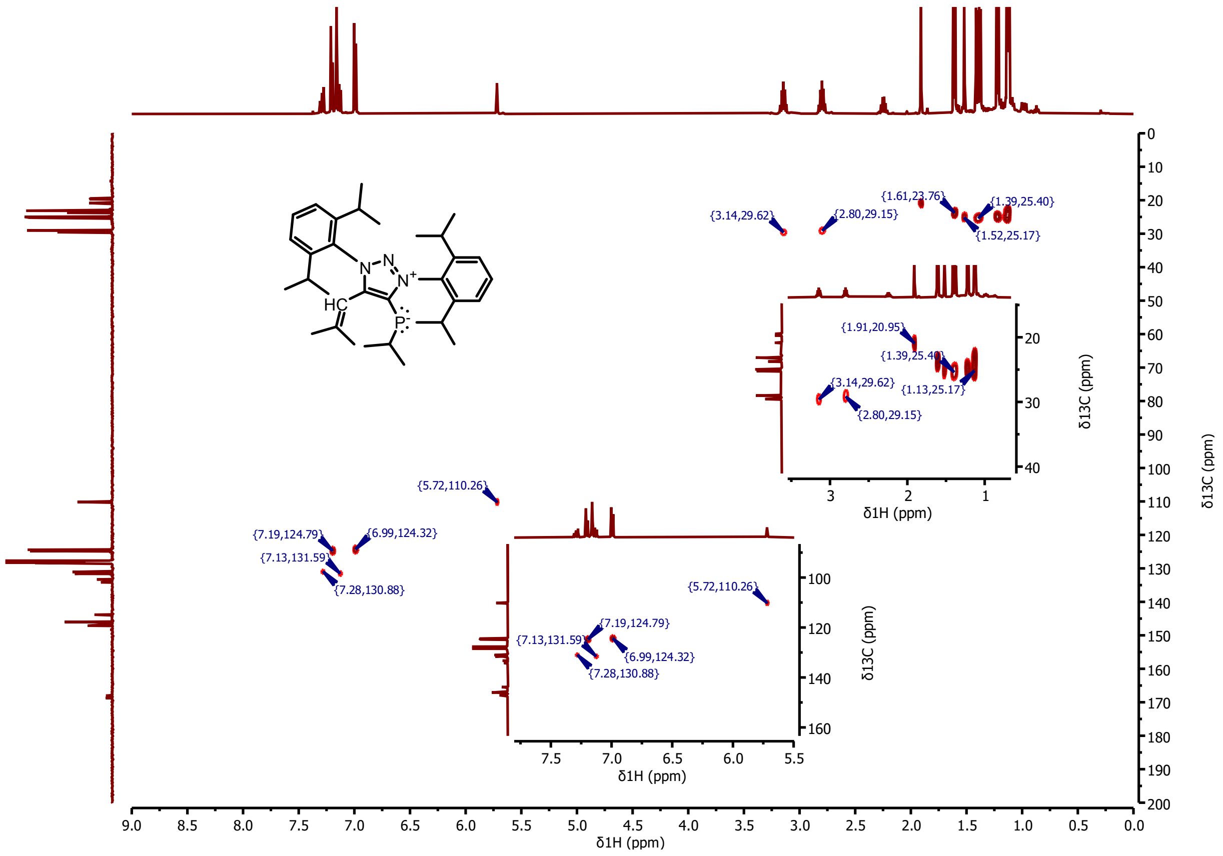

Fig S56: ^{1}H/^{13}C HSQC (500/125 MHz, C_6D_6, 298K) of **11.**

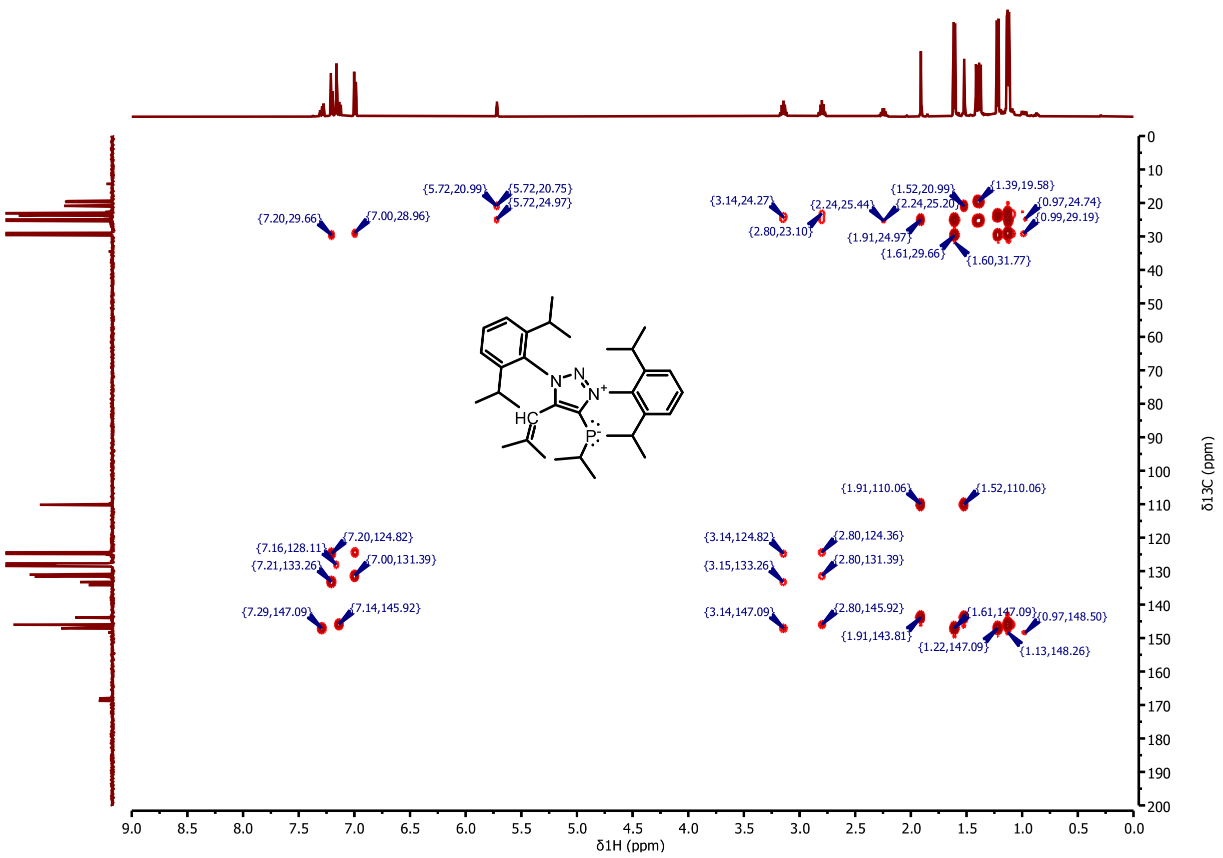

Fig S57: $^{1}H/^{13}C$ **HMBC** (500/125 MHz, C_6D_6, 298K) of **11.**

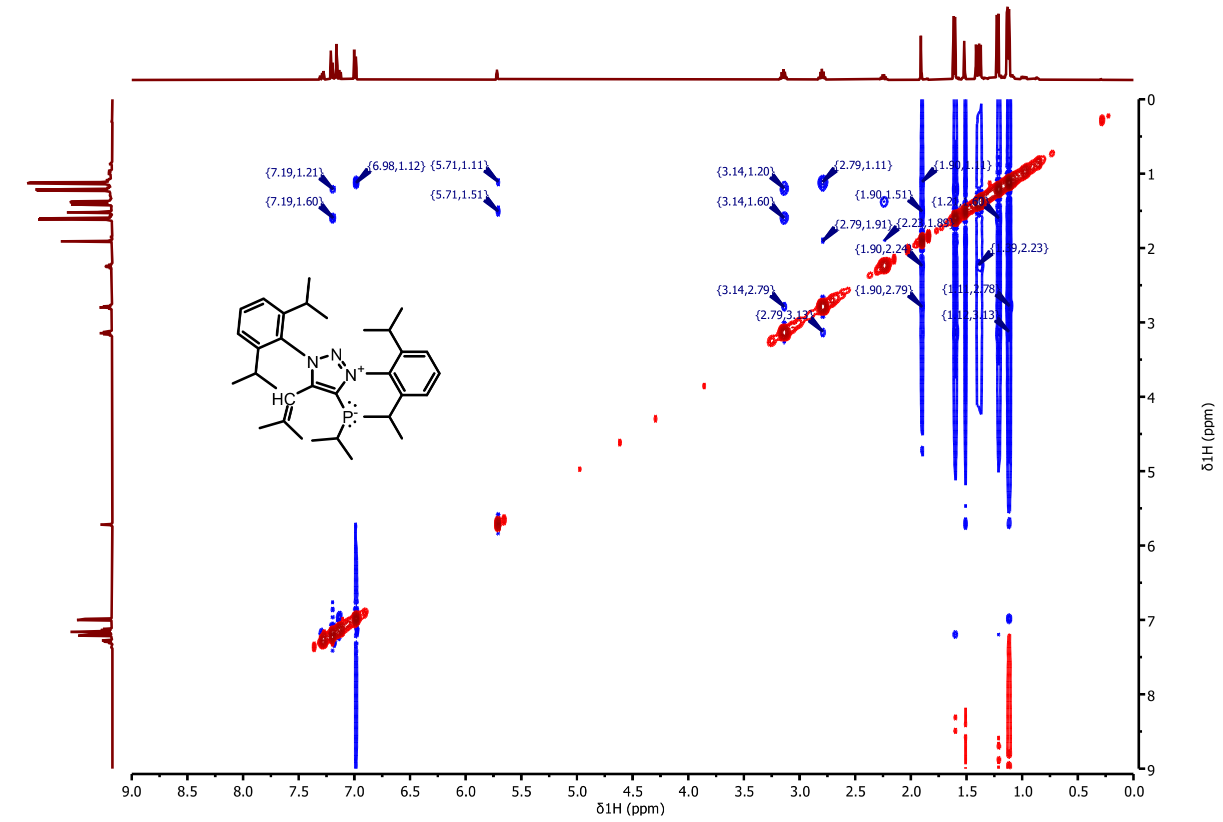

Fig S58: $^{1}H/^{1}H$ **NOESY** (500/500 MHz, C_6D_6, 298K) of **11.**

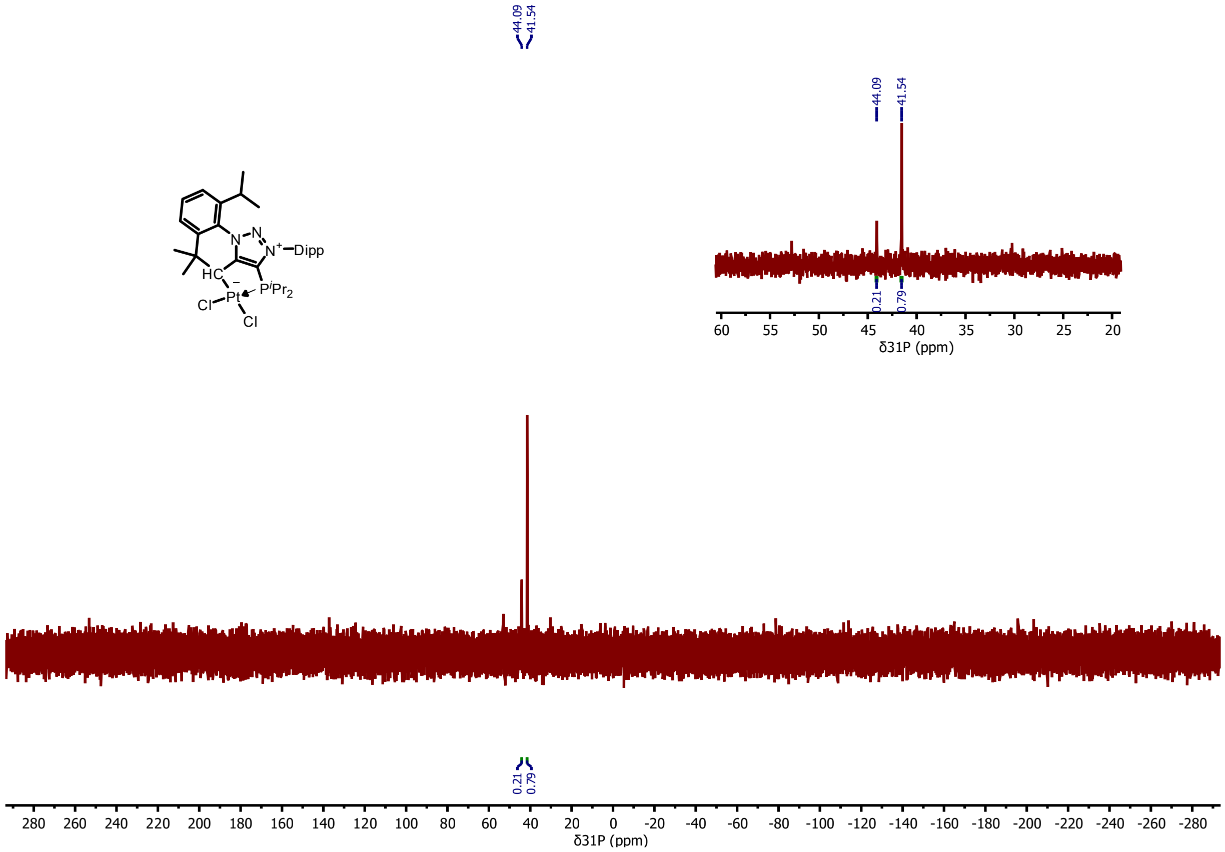

Fig S59: Crude ^{31}P NMR (203 MHz, CD_2Cl_2, 298K) of **12.**

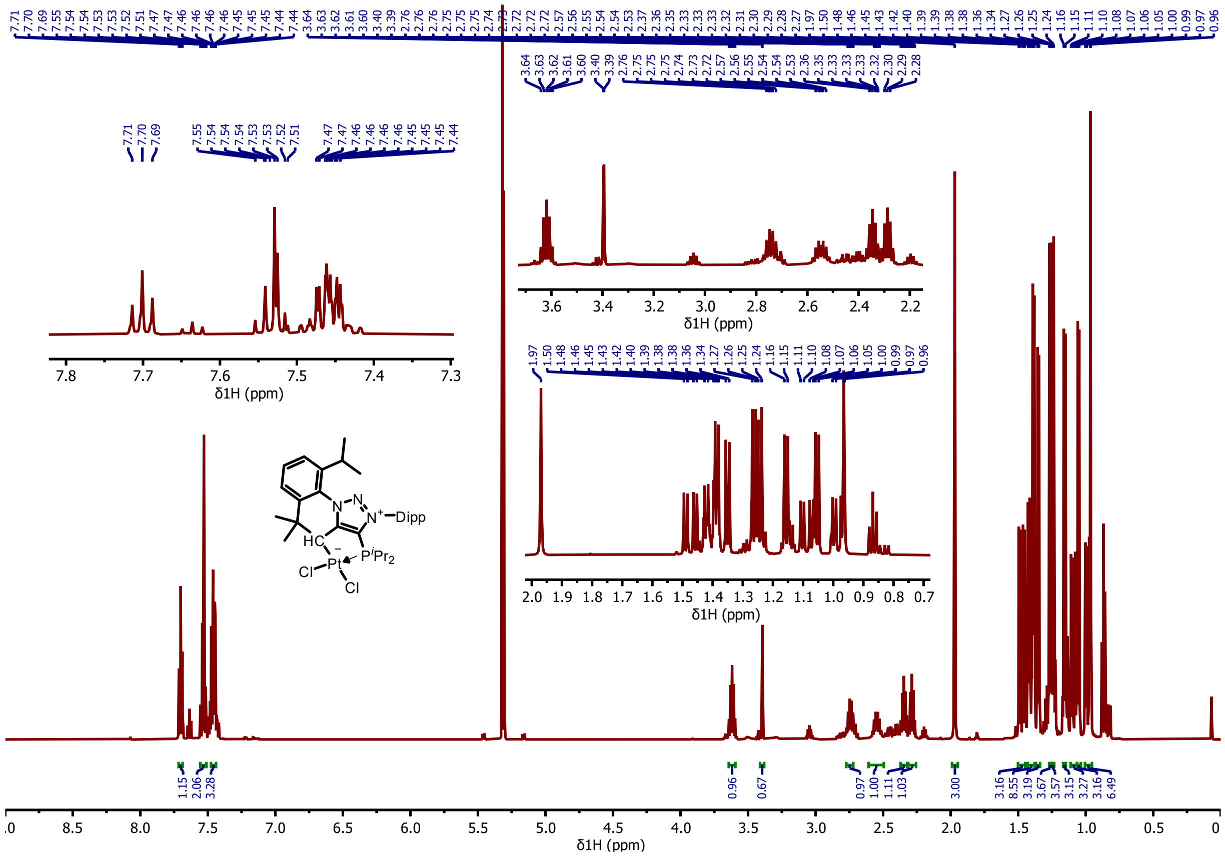

Fig S60: ^{1}H NMR (600 MHz, CD_2Cl_2, 298K) of **12.**

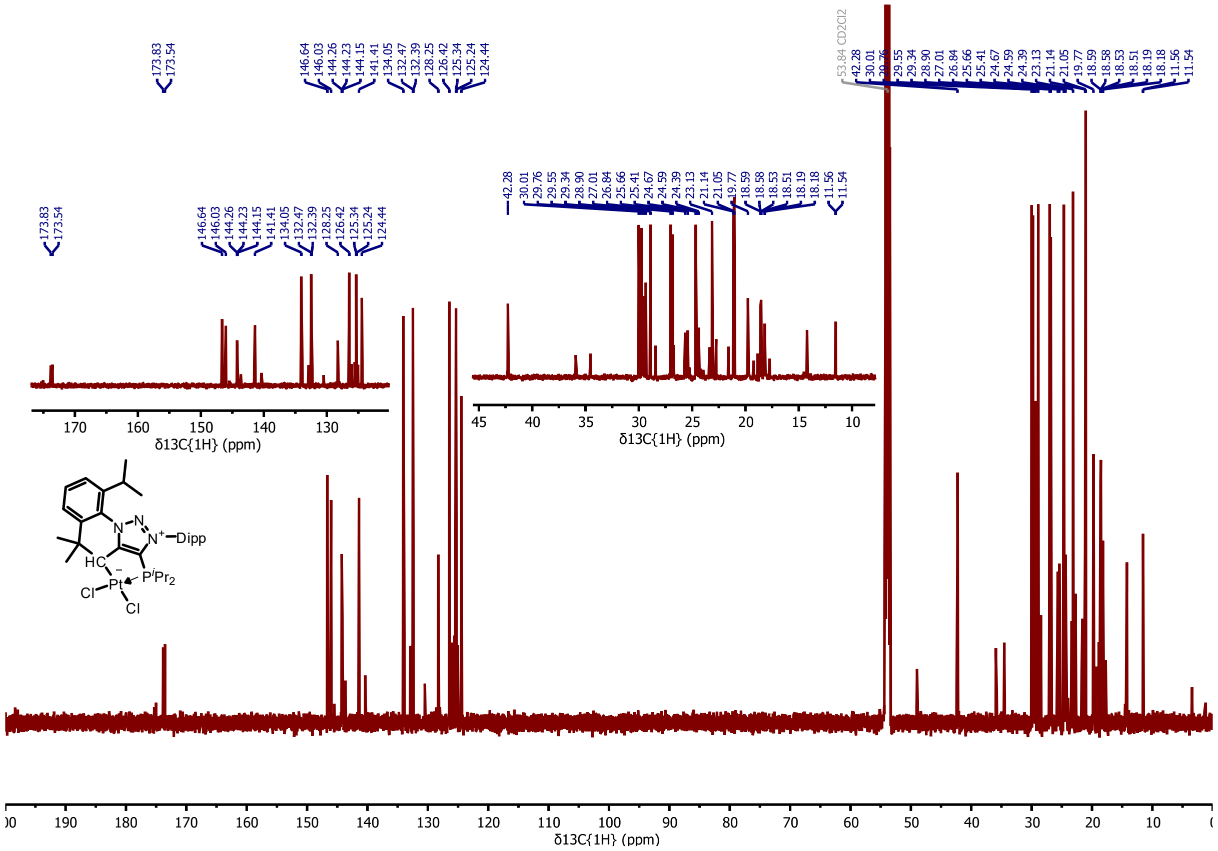

Fig S61: ^{13}C NMR {^{1}H} (150 MHz, CD_2Cl_2, 298K) of **12.**

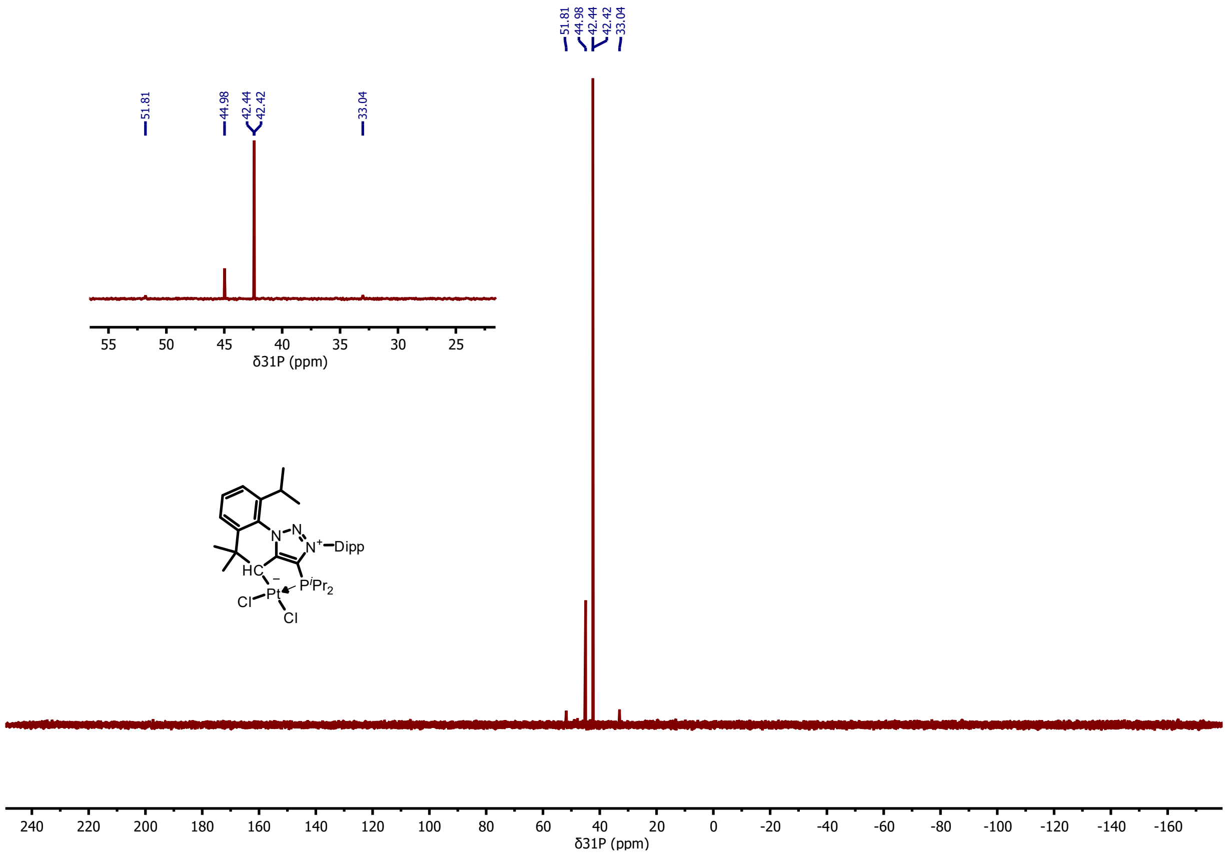

Fig S62: ^{31}P NMR (243 MHz, CD_2Cl_2, 298K) of **12.**

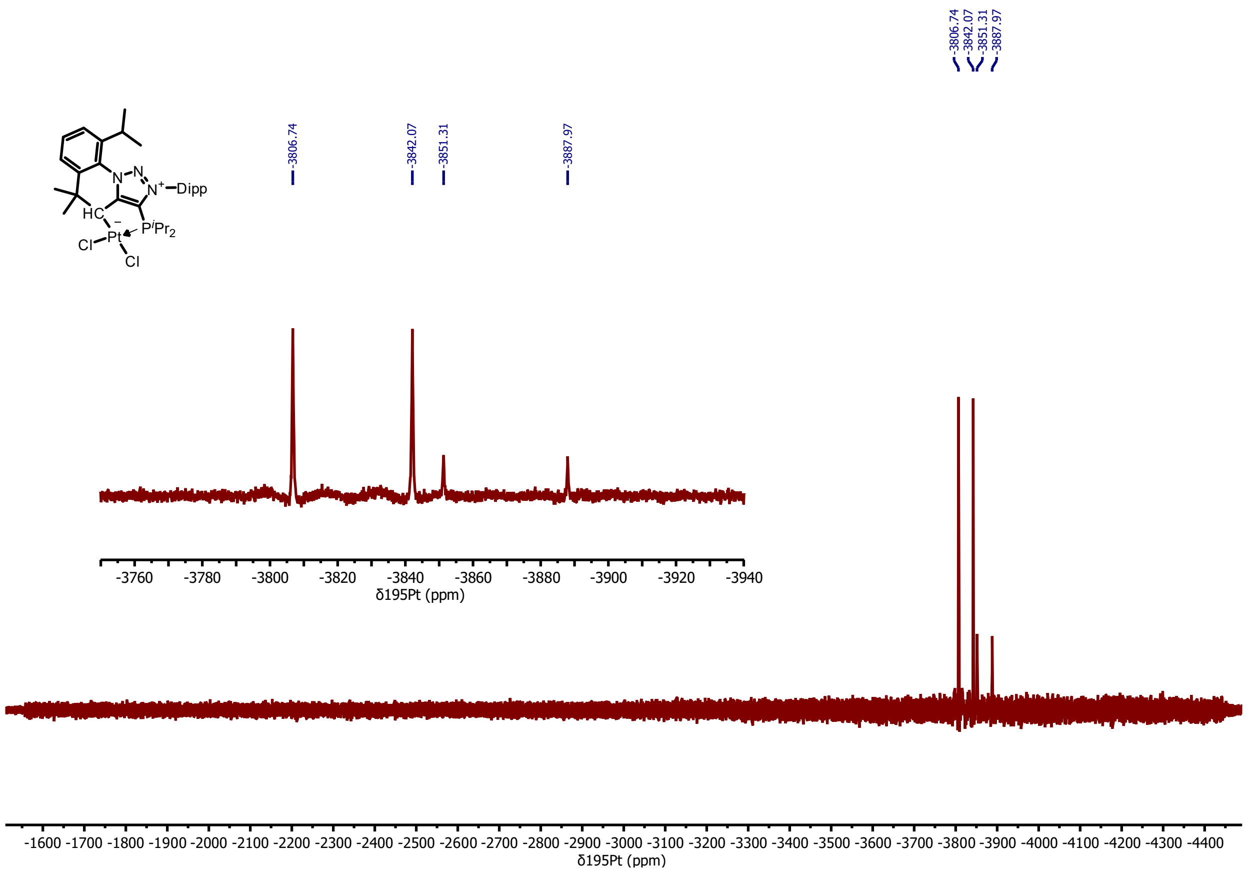

Fig S63: ^{195}**Pt NMR** (129 MHz, CD_2Cl_2, 298K) of **12.**

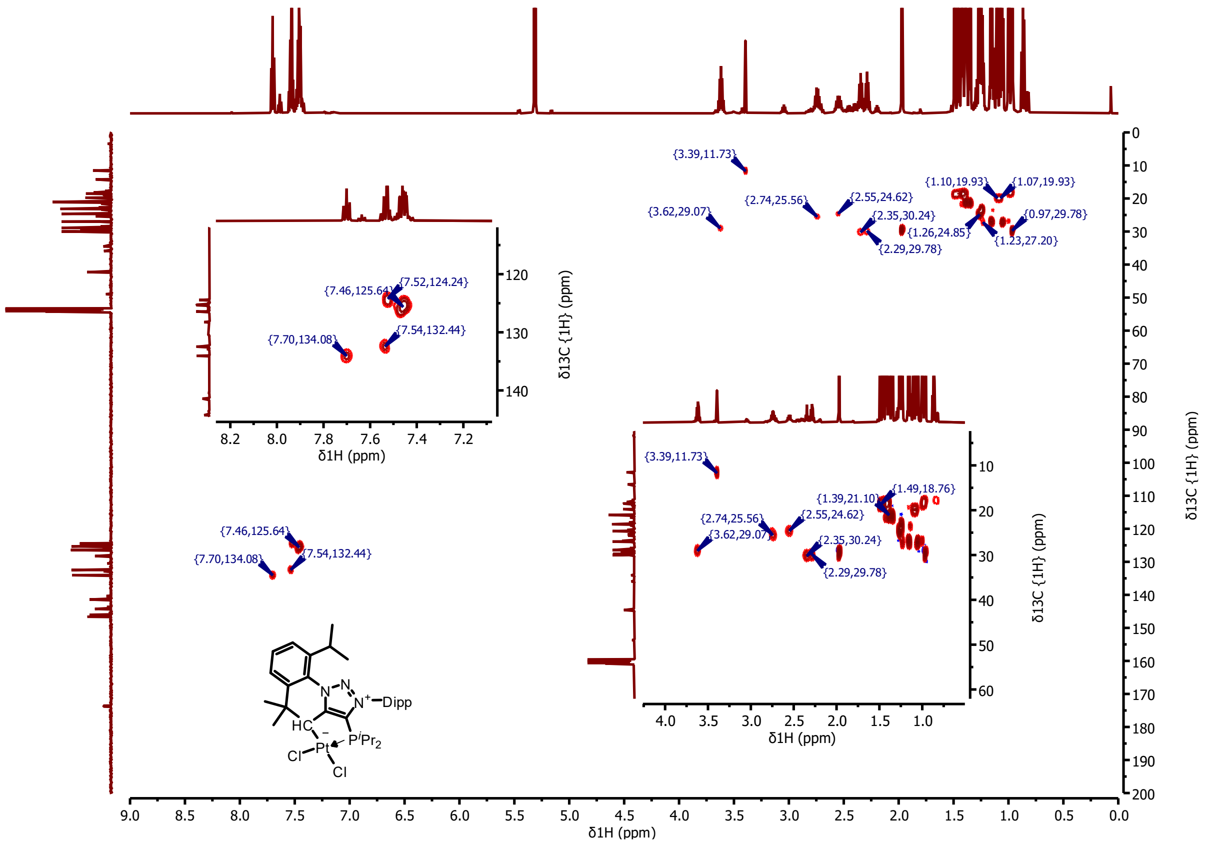

Fig S64: ^{1}**H/**^{13}**C HSQC** (600/150 MHz, CD_2Cl_2, 298K) of **12.**

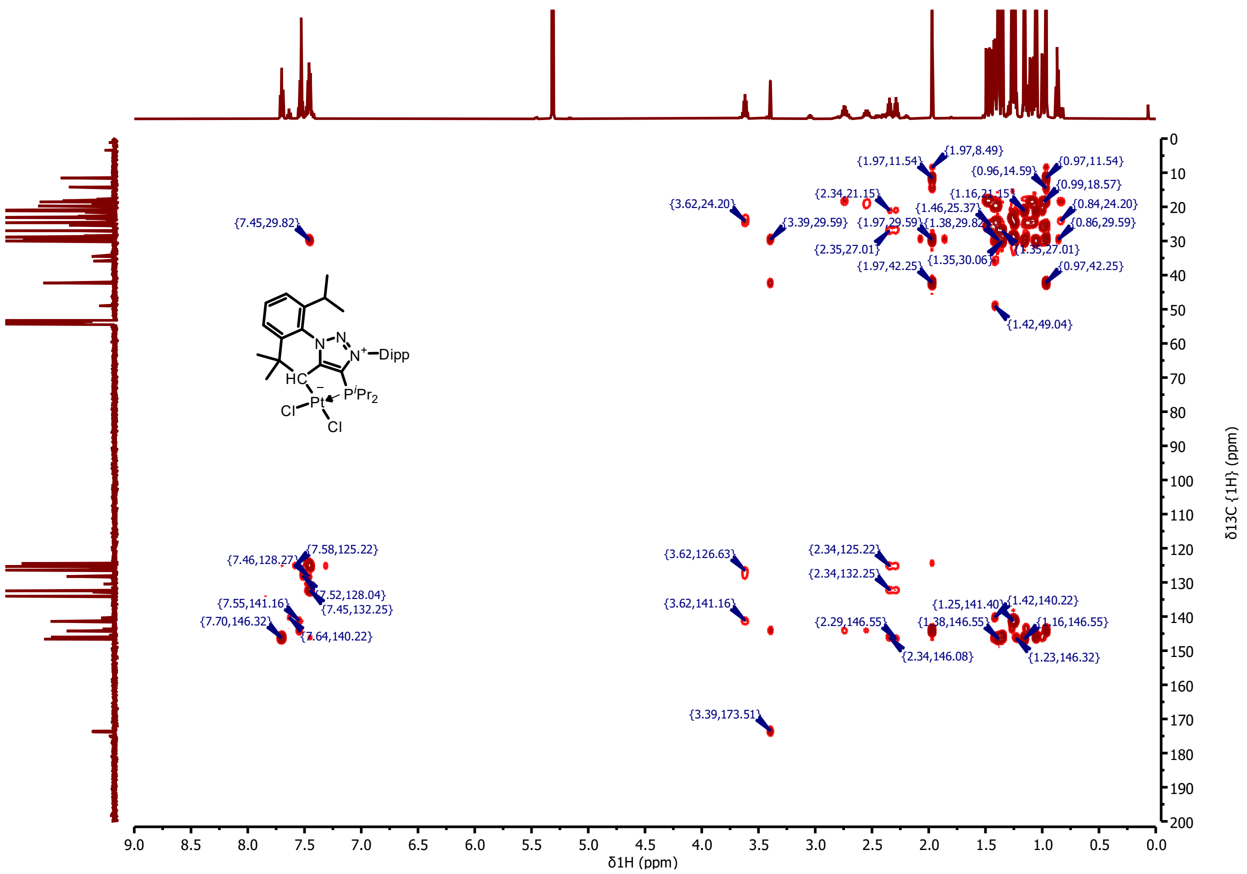

Fig S65: ^{1}H/^{13}C HMBC (600/150 MHz, CD_2Cl_2, 298K) of **12.**

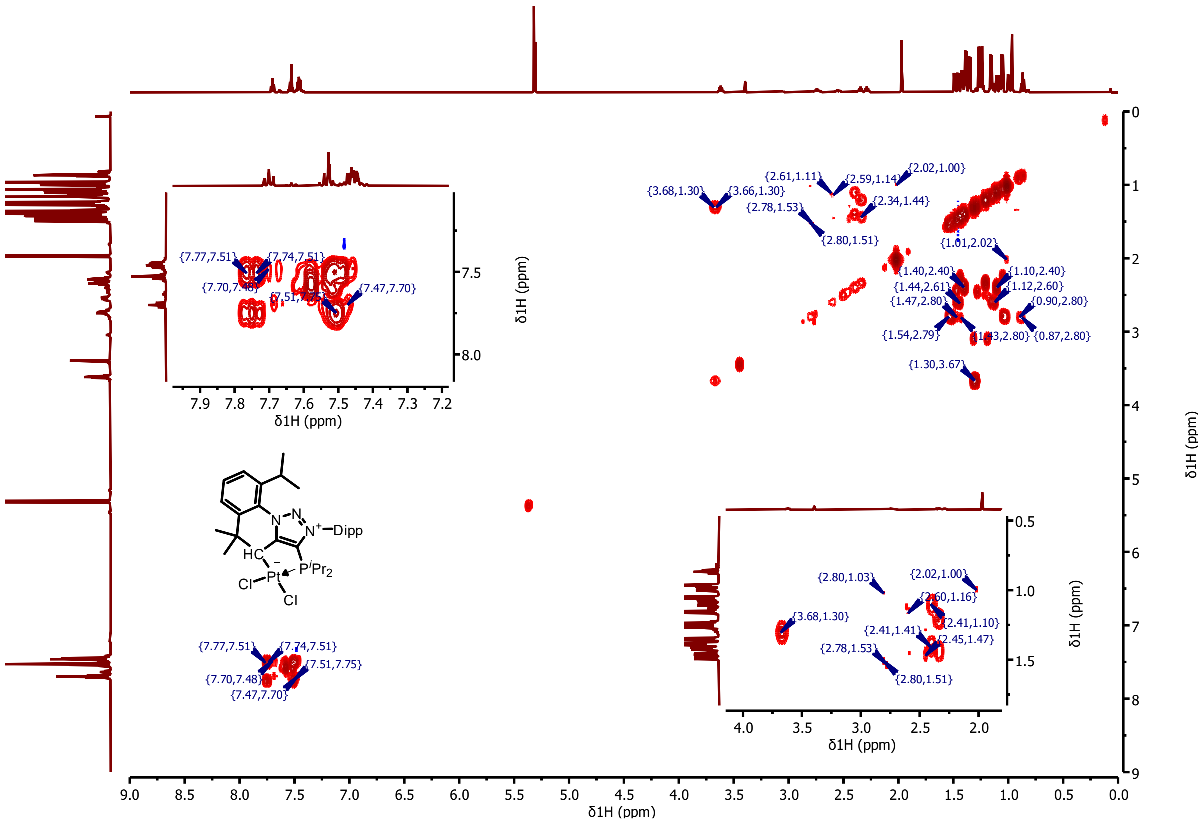

Fig S66: ^{1}H/^{1}H COSY (600/600 MHz, CD_2Cl_2, 298K) of **12.**

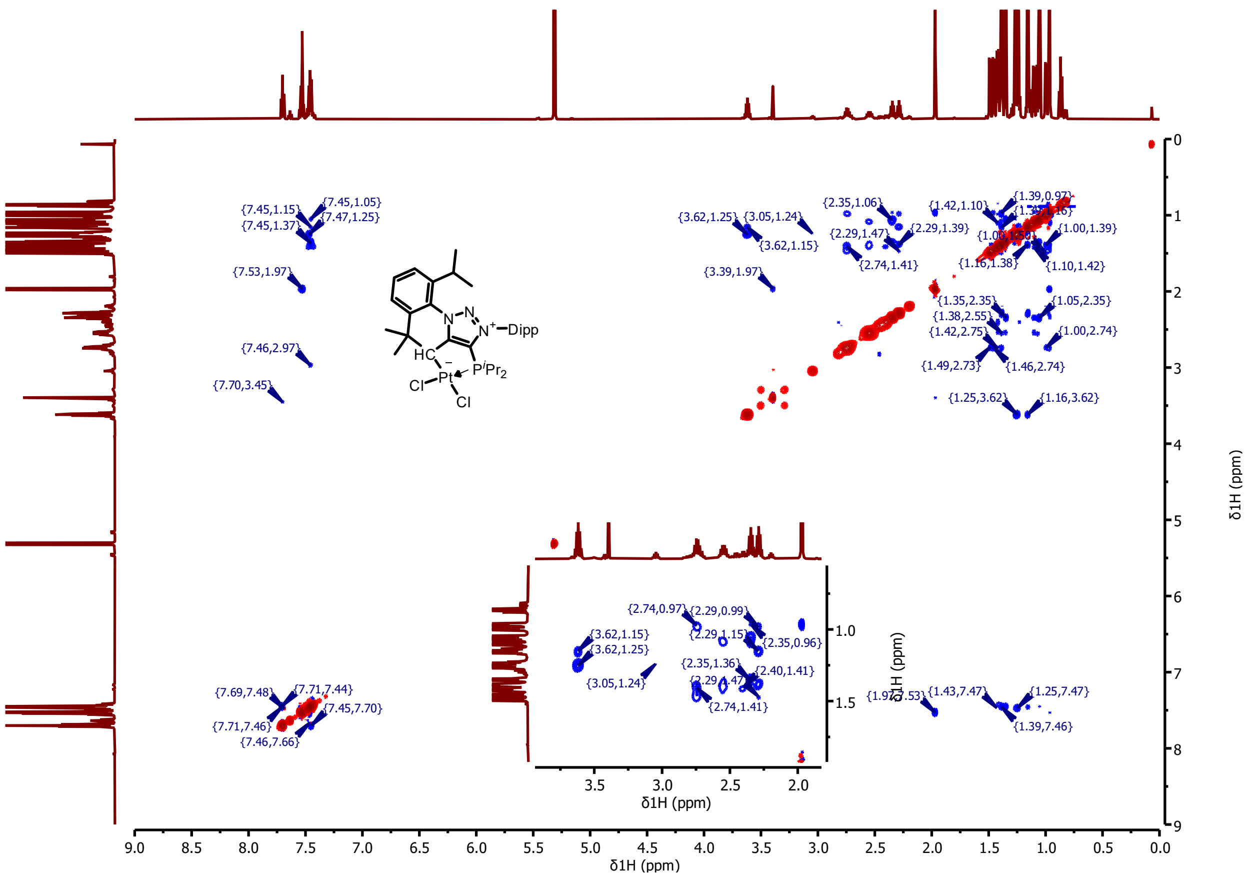

Fig S67: $^1H/^1H$ **NOESY** (600/600 MHz, CD_2Cl_2, 298K) of **12.**

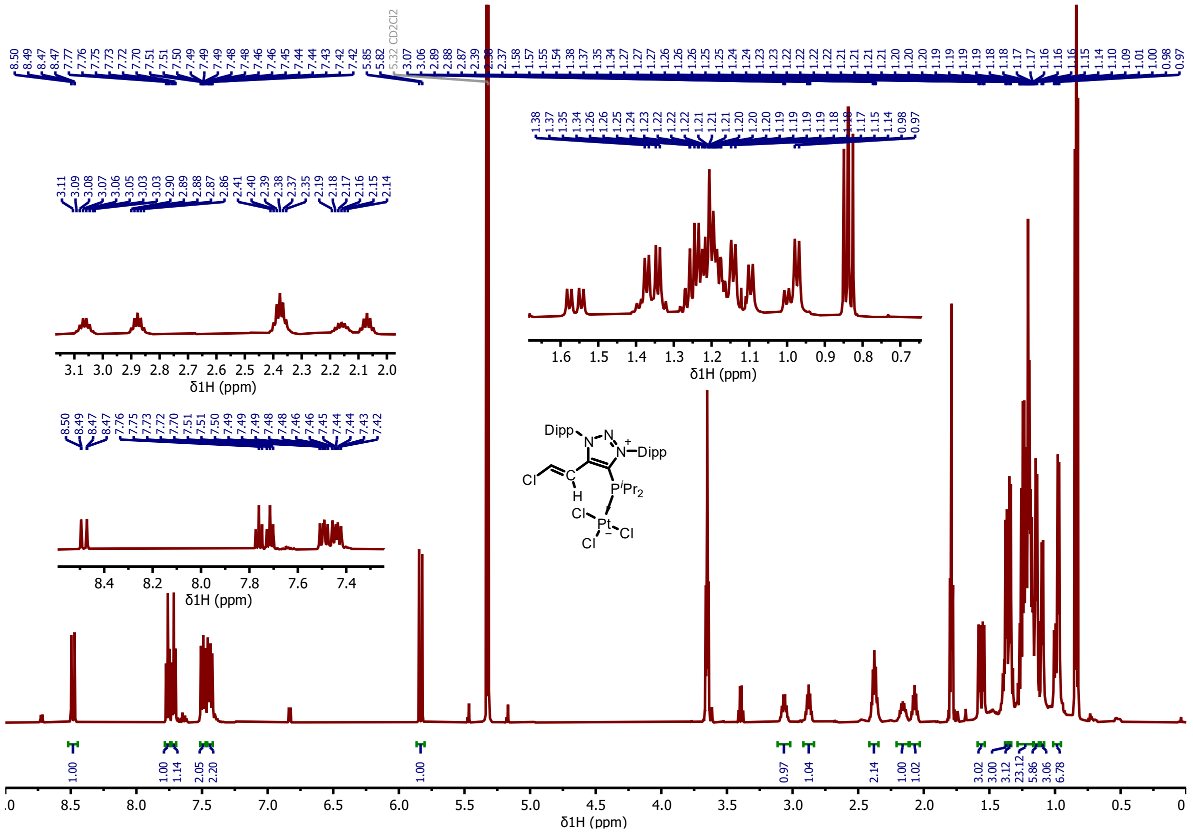

Fig S68: ^1H **NMR** (600 MHz, CD_2Cl_2, 243K) of **13.**

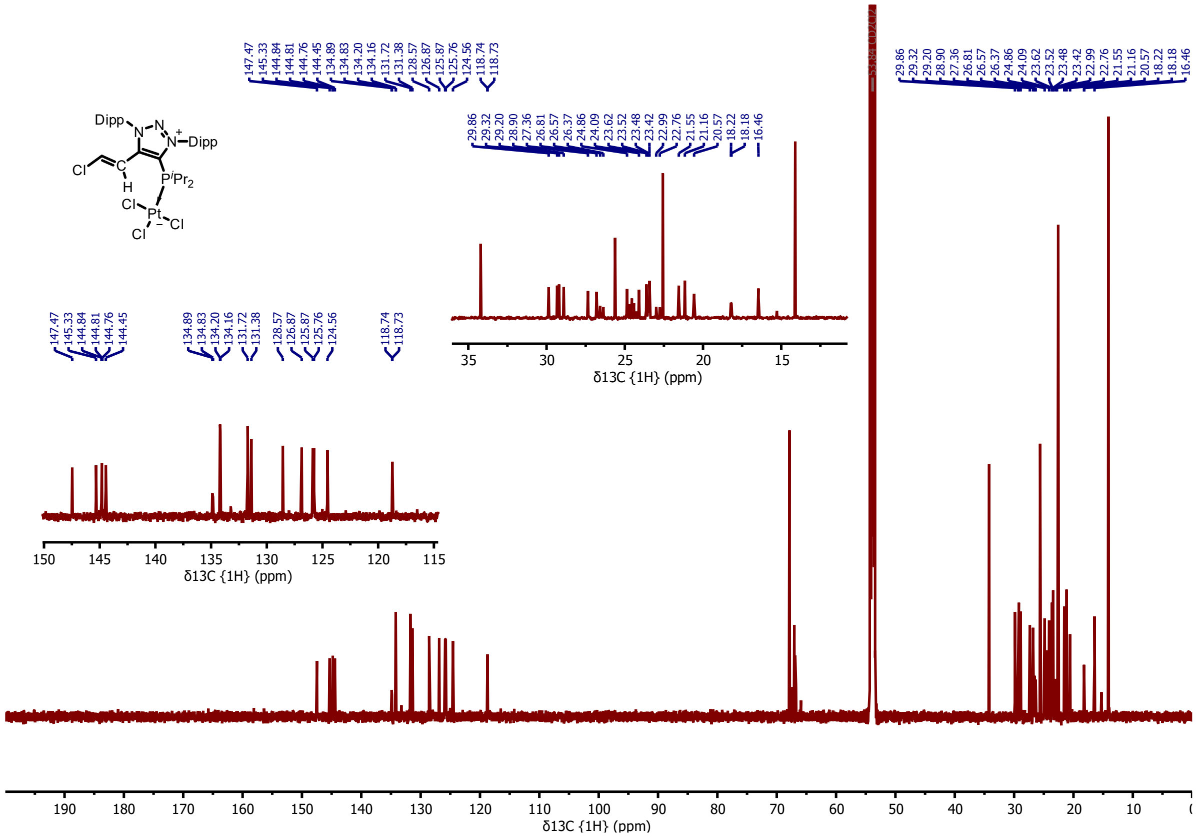

Fig S69: ^{13}**C NMR {**^{1}**H}** (150 MHz, CD_2Cl_2, 243K) of **13.**

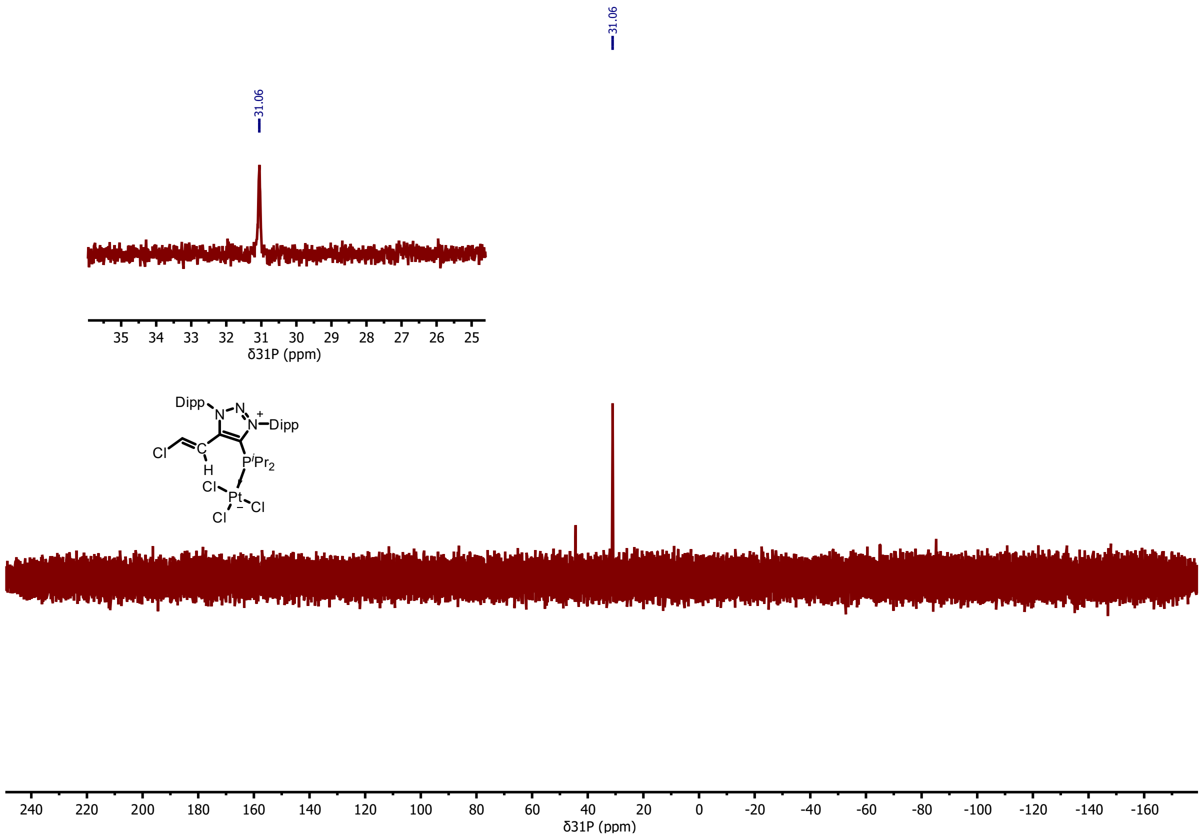

Fig S70: ^{31}**P NMR** (243 MHz, CD_2Cl_2, 243K) of **13.**

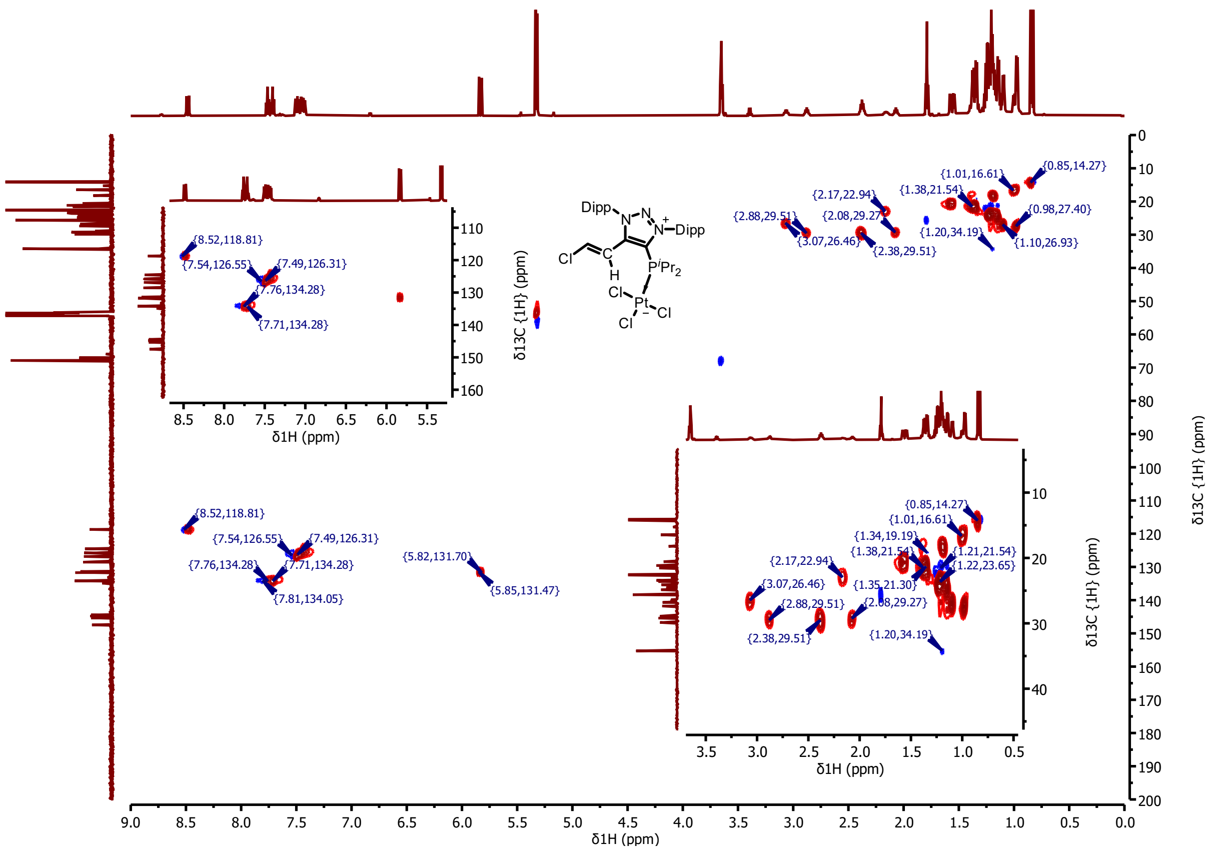

Fig S71: ^{1}H/^{13}C HSQC (600/150 MHz, CD_2Cl_2, 243K) of **13.**

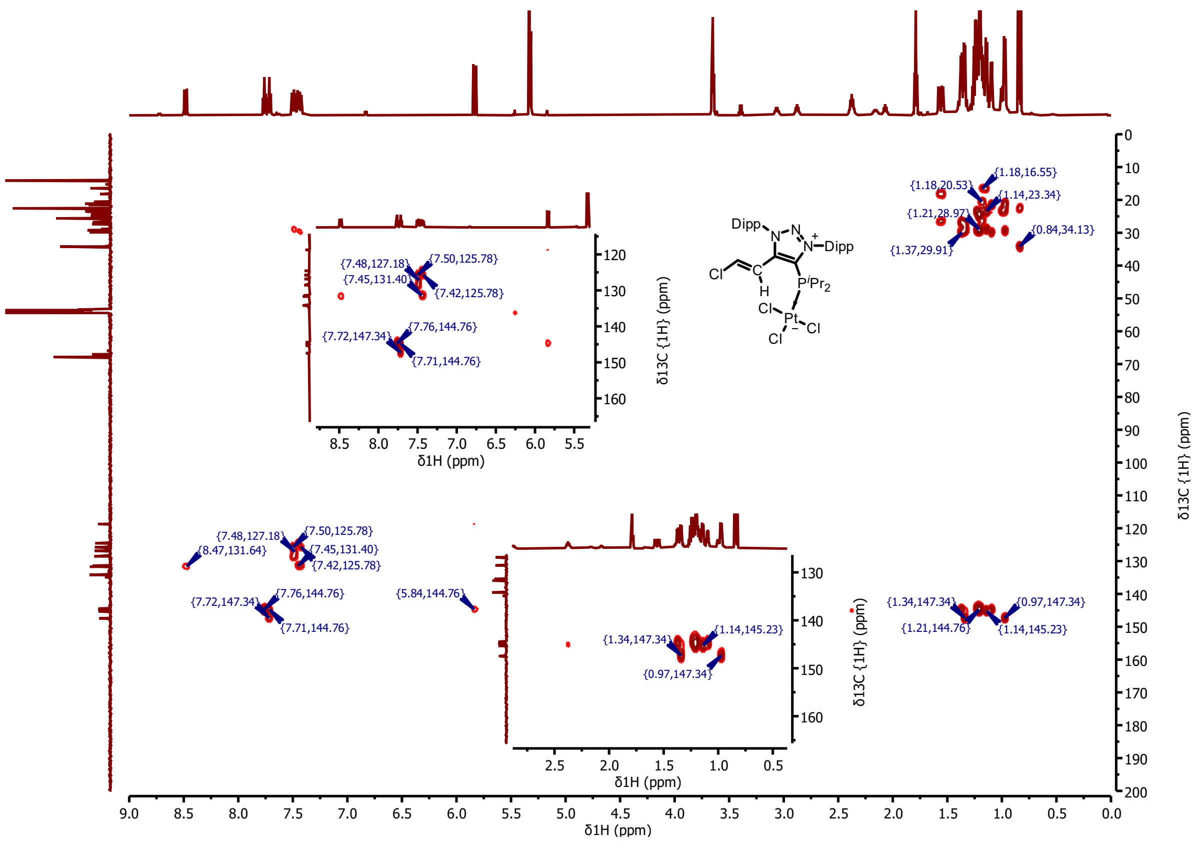

Fig S72: ^{1}H/^{13}C HMBC (600/150 MHz, CD_2Cl_2, 243K) of **13.**

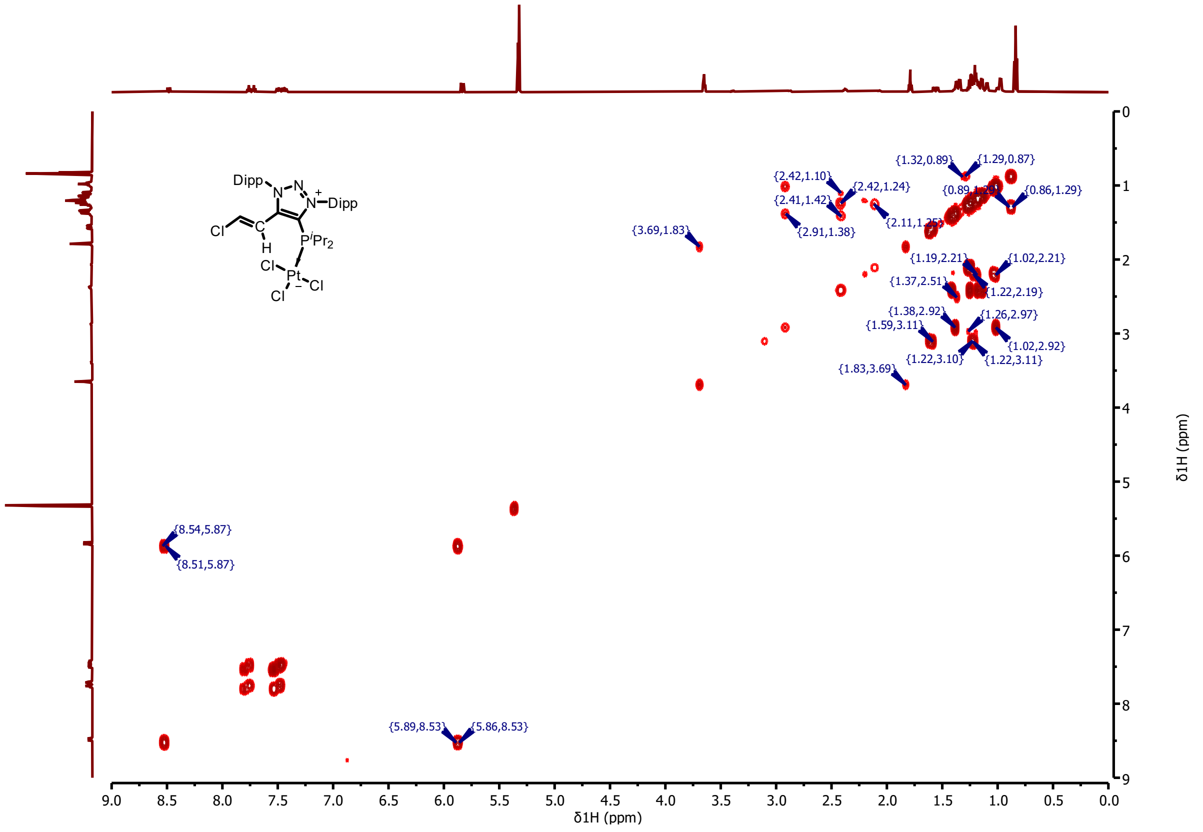

Fig S73: ^{1}H/^{1}H COSY (600/600 MHz, CD_2Cl_2, 243K) of **13.**

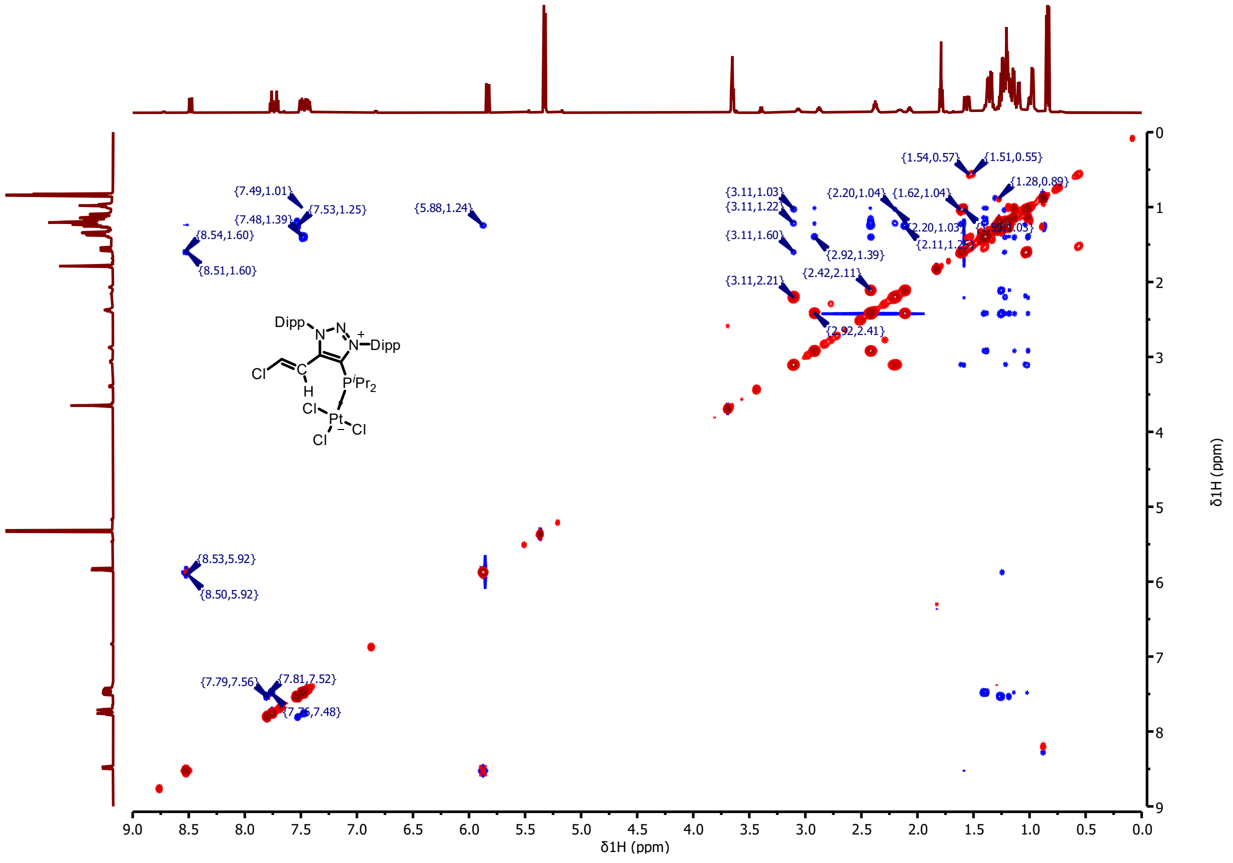

Fig S74: ^{1}H/^{1}H NOESY (600/600 MHz, CD_2Cl_2, 243K) of **13.**

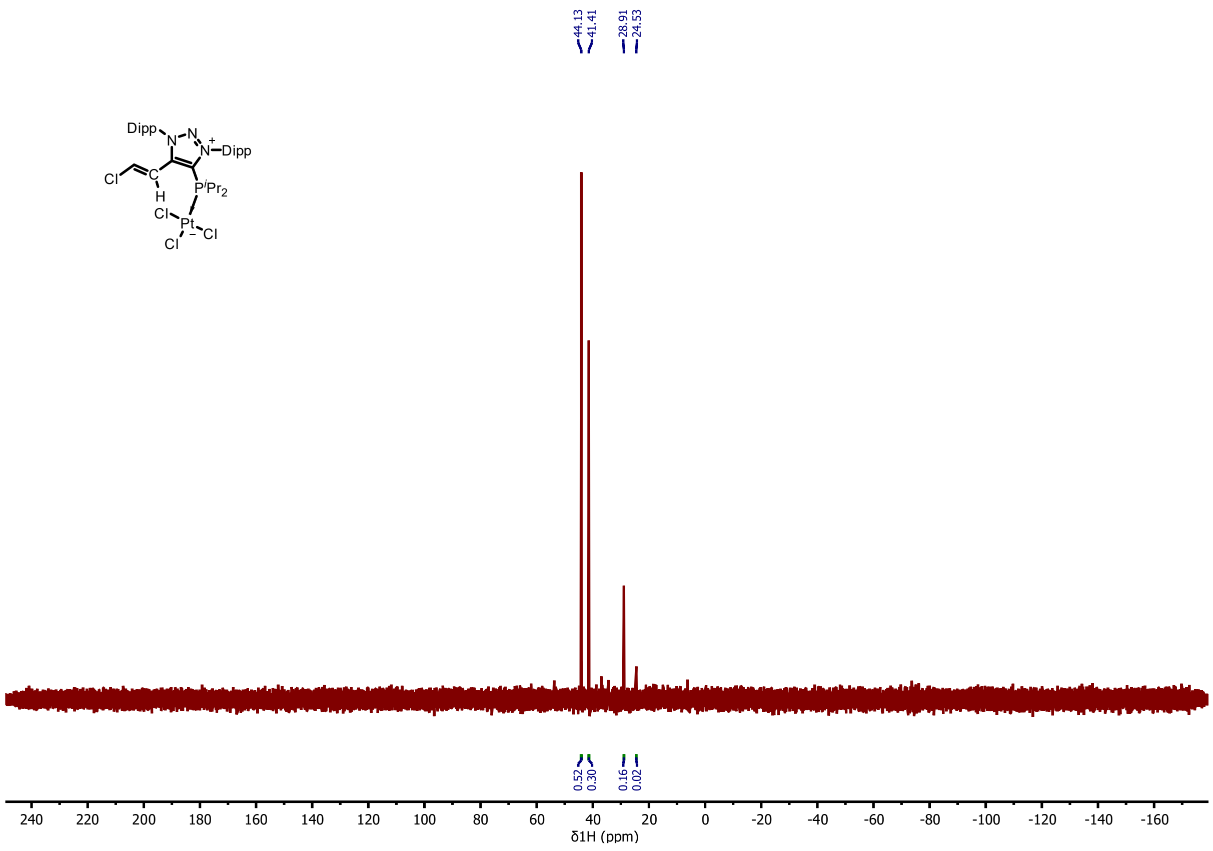

Fig S75: Crude ^{31}P NMR (203 MHz, CD_2Cl_2, 298K) of **13.**

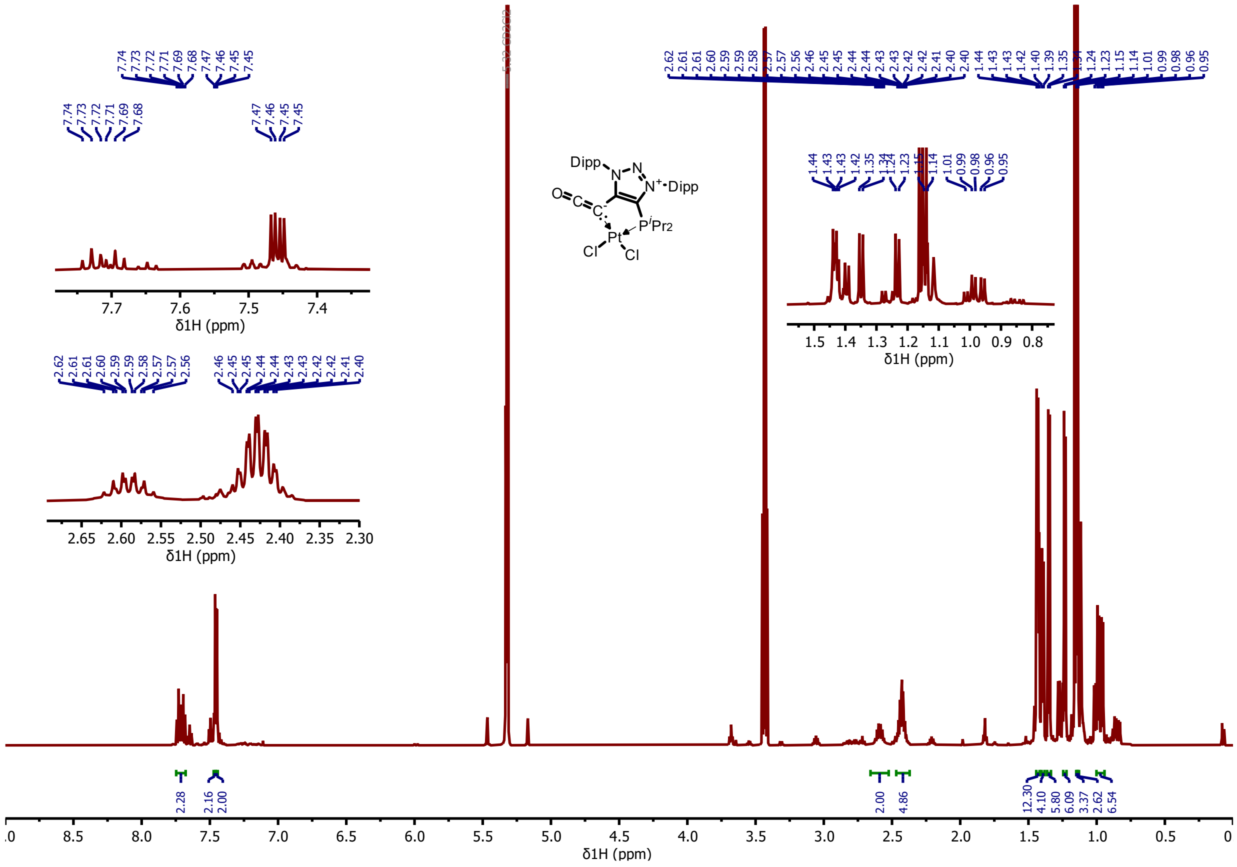

Fig S76: ^{1}H NMR (600 MHz, CD_2Cl_2, 298K) of **14.**

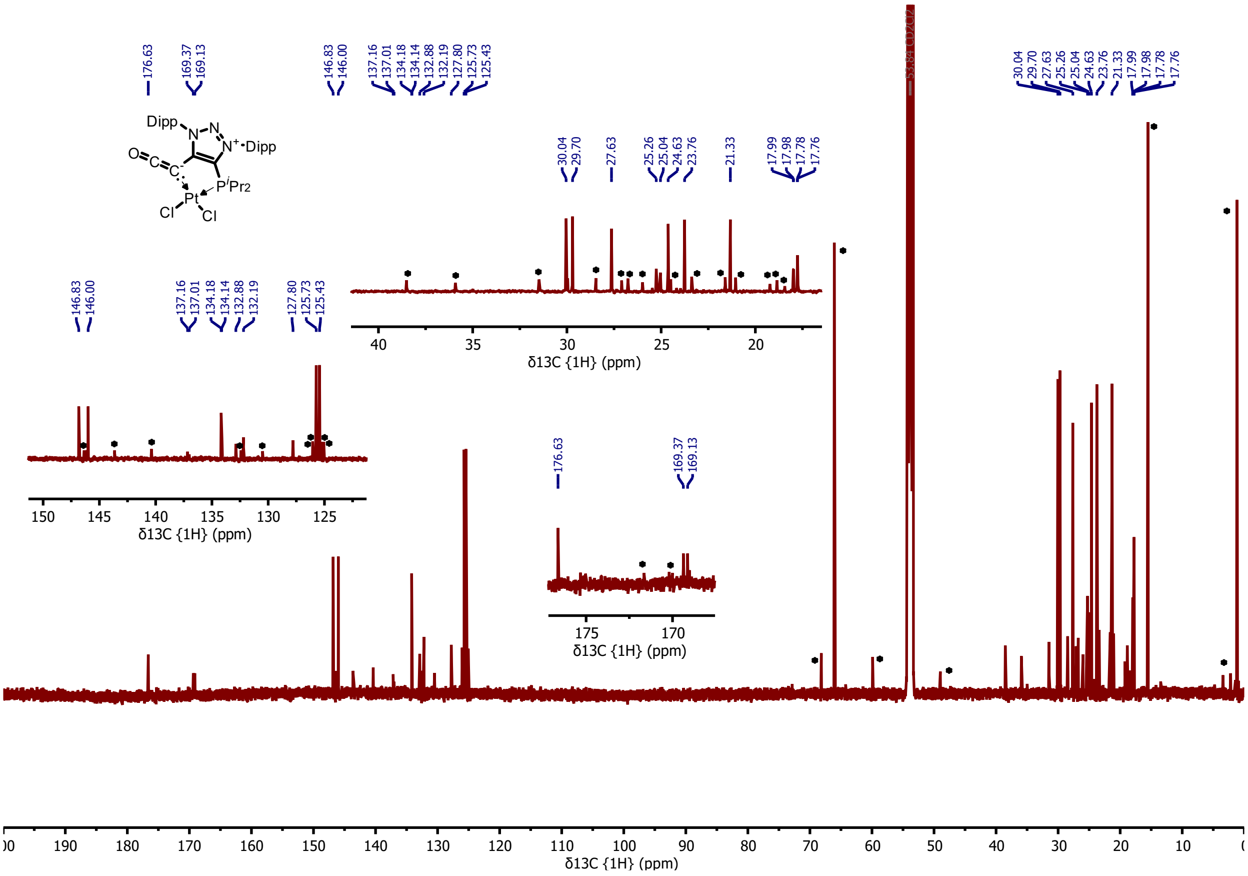

Fig S77: ^{13}C NMR {^{1}H} (150 MHz, CD_2Cl_2, 298K) of **14**. *Et_2O, grease and irradiation product.

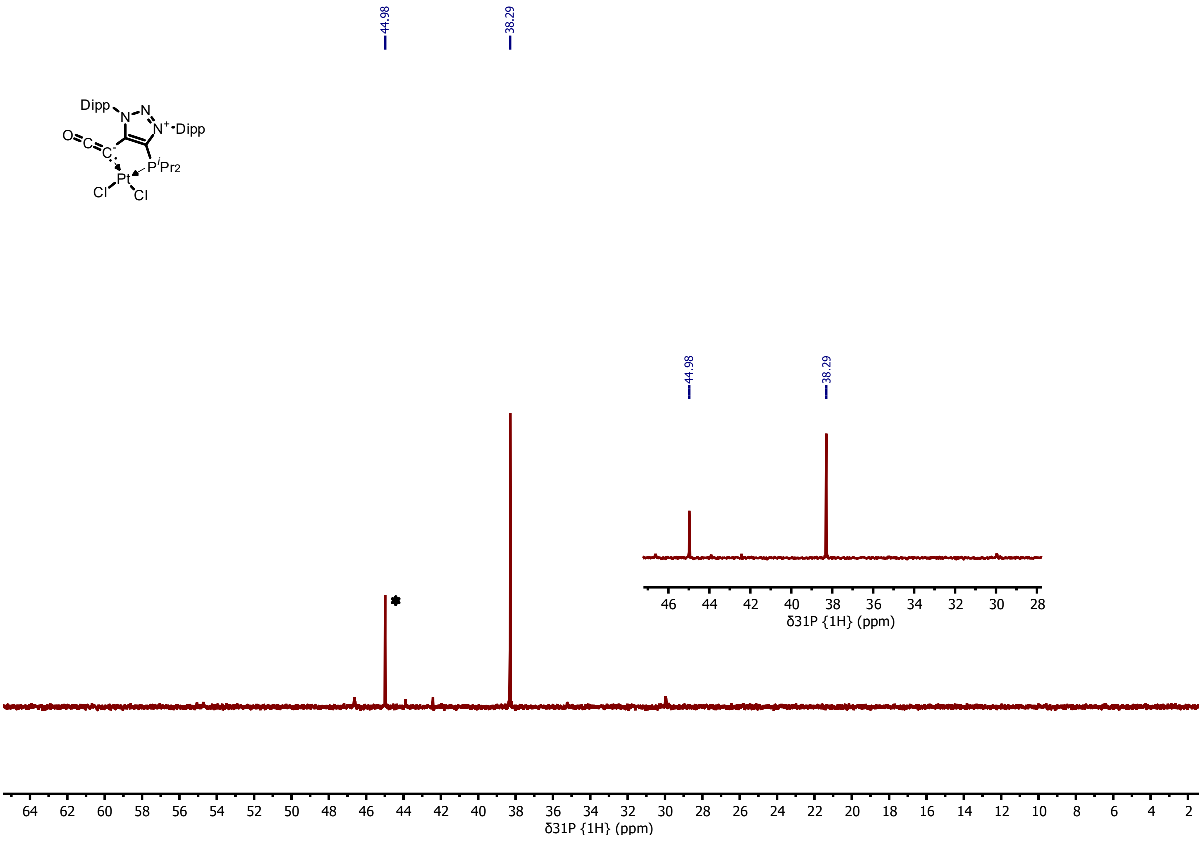

Fig S78: ^{31}P NMR (243 MHz, CD_2Cl_2, 298K) of **14**.* irradiation product.

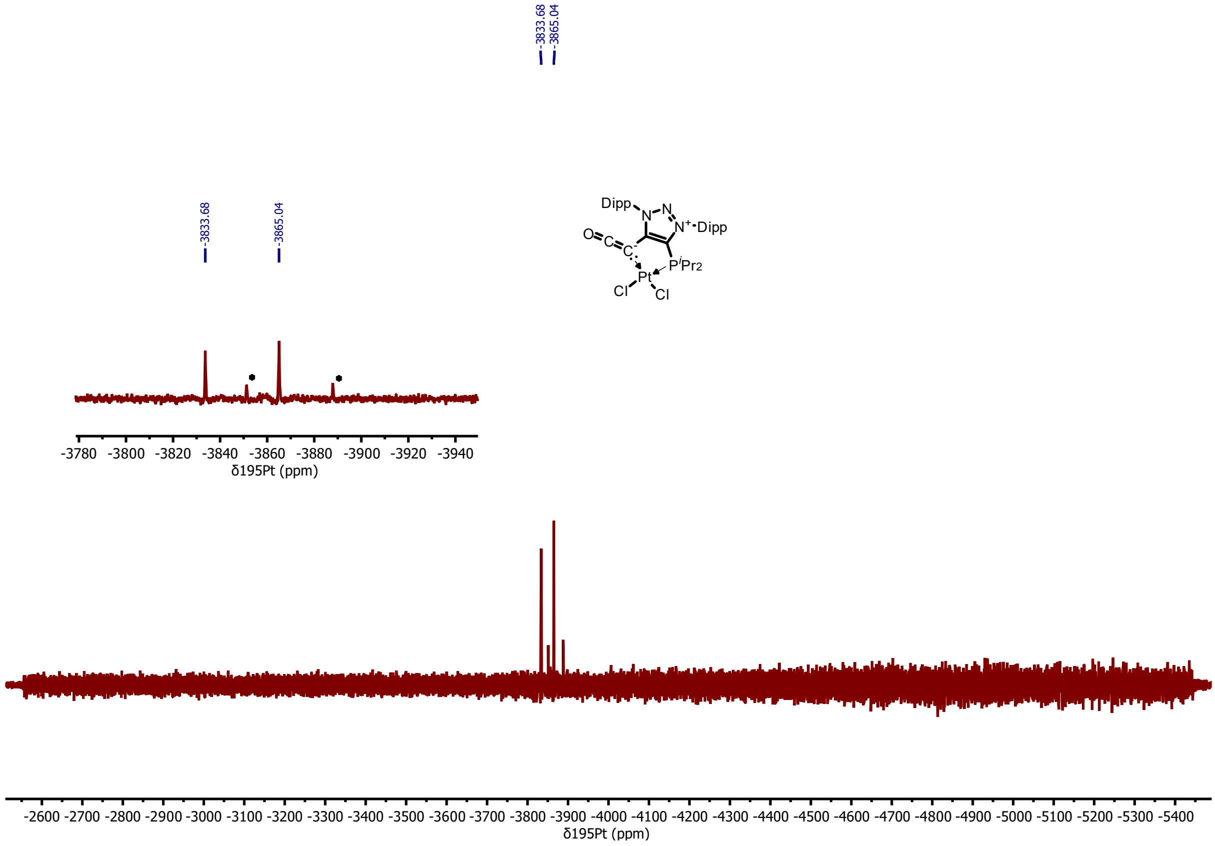

Fig S79: ^{195}Pt NMR (129 MHz, CD_2Cl_2, 298K) of **14**.*irradiation product.

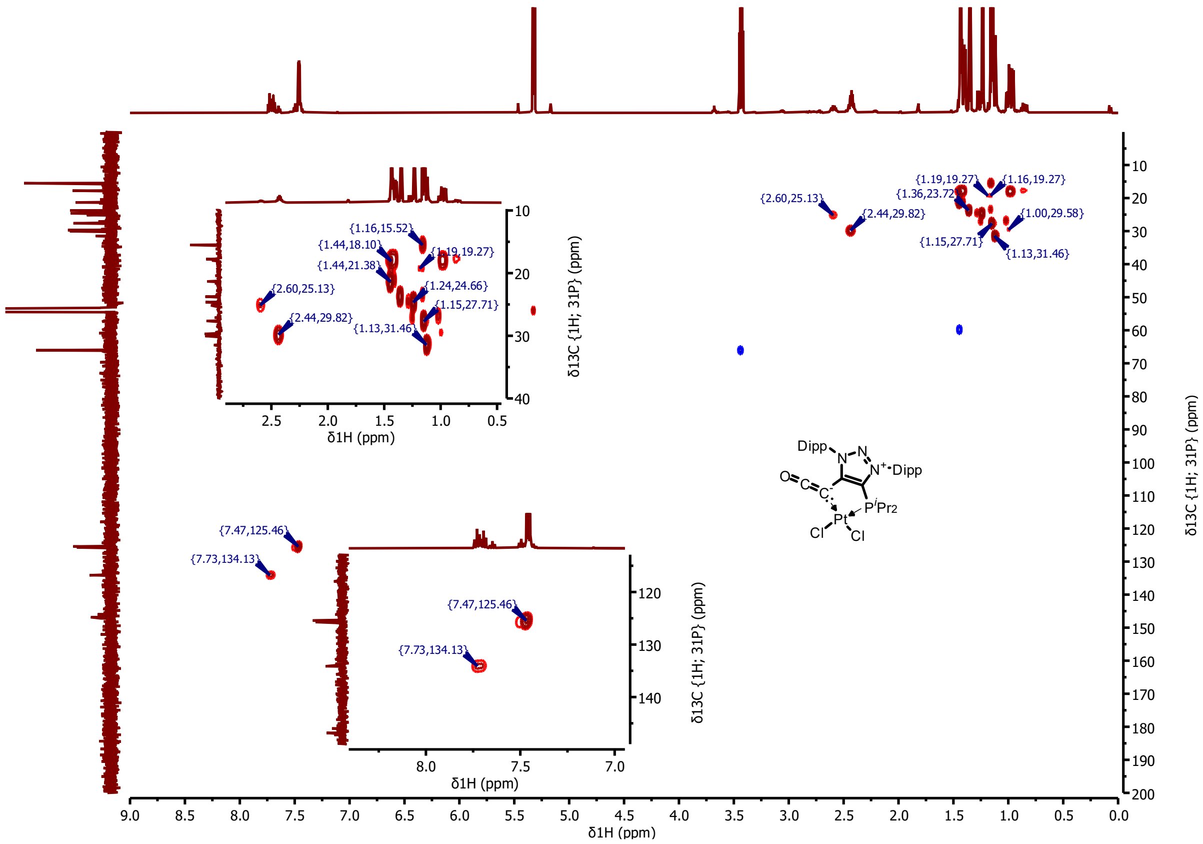

Fig S80: ^{1}H/^{13}C HSQC (600/150 MHz, CD_2Cl_2, 298K) of **14.**

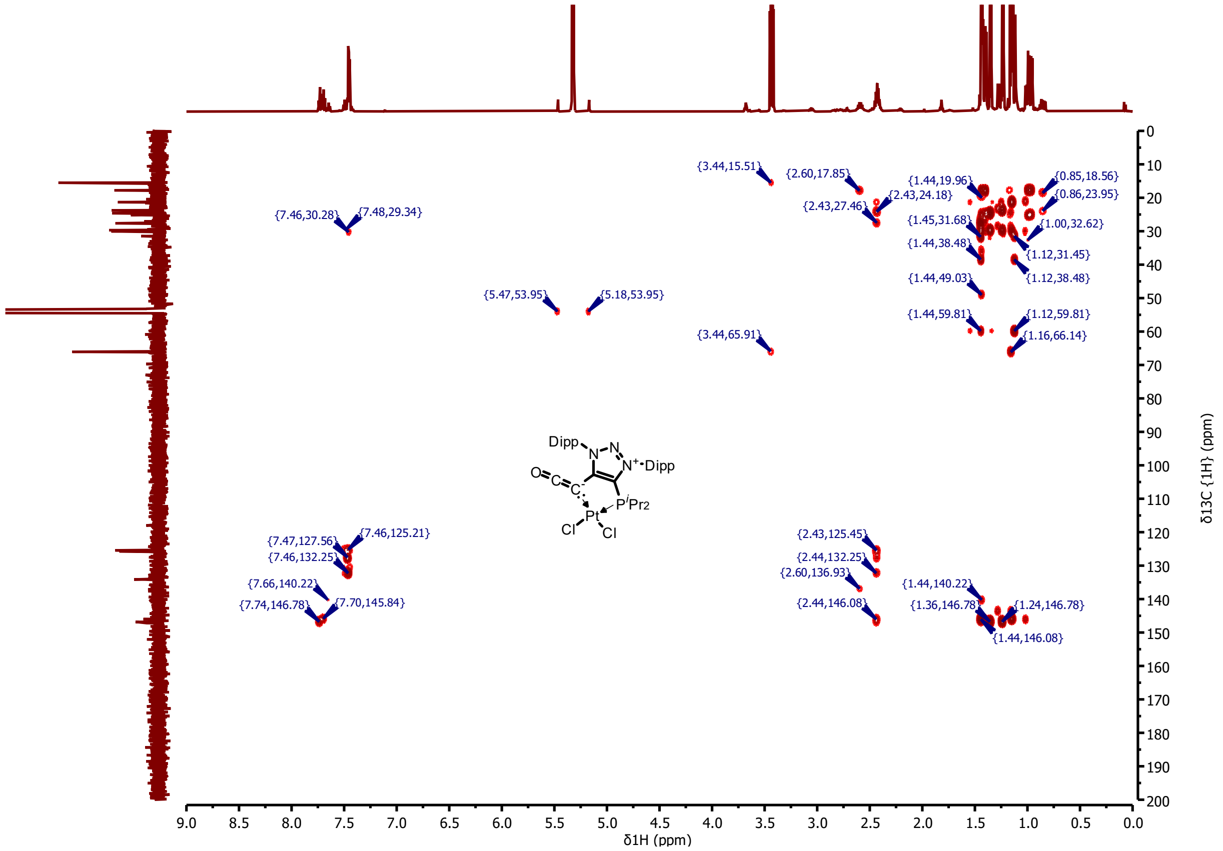

Fig S81: $^{1}H/^{13}C$ HMBC (600/150 MHz, CD_2Cl_2, 298K) of **14.**

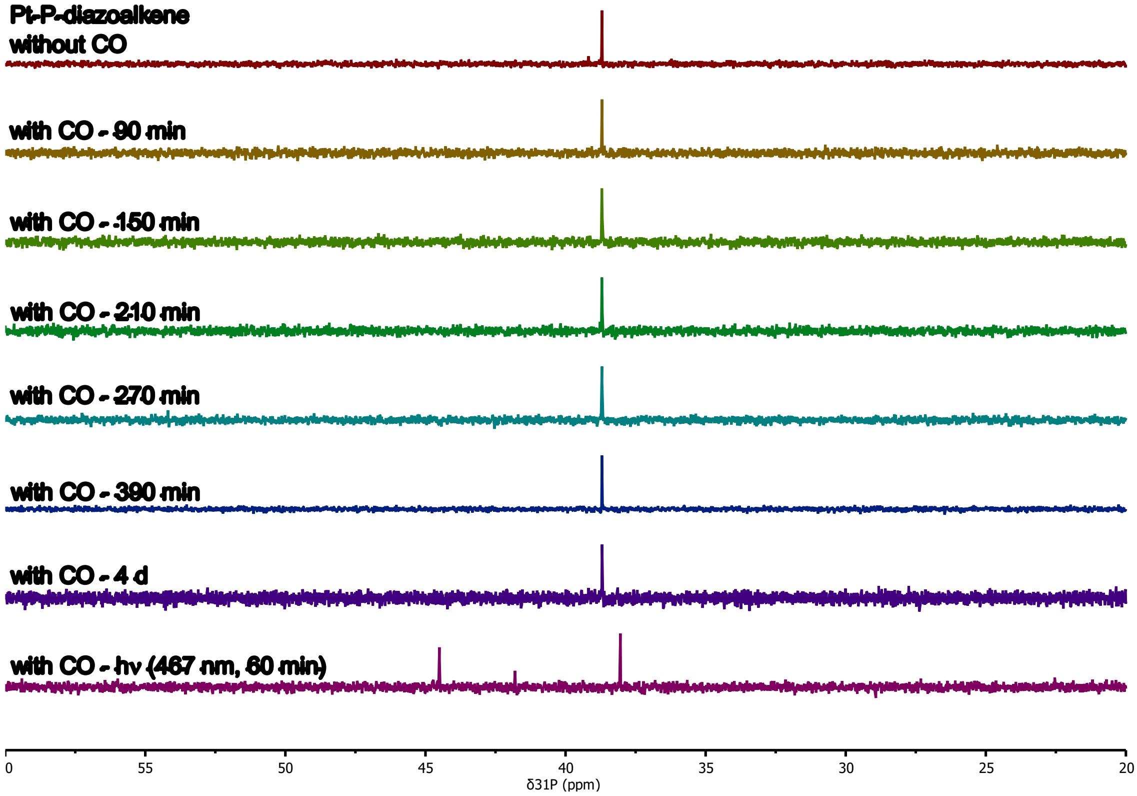

Fig S82: Crude ^{31}P NMR stack (203 MHz, CD_2Cl_2, 298K) of **14.**

4. IR Spectra

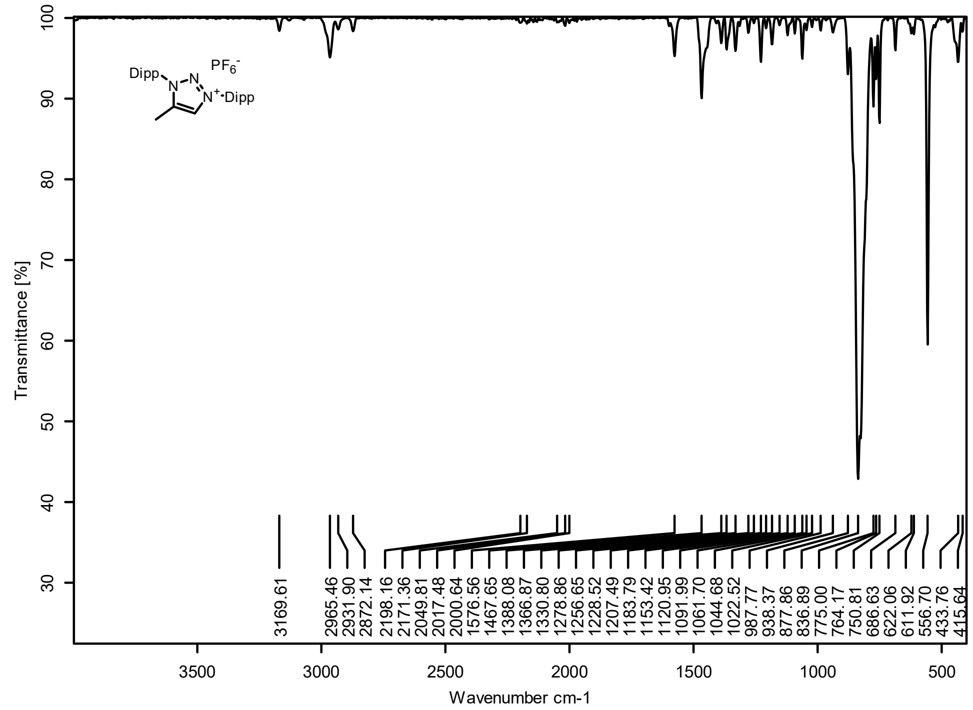

Fig. S83: ATR-IR spectrum (solid) of **1**.

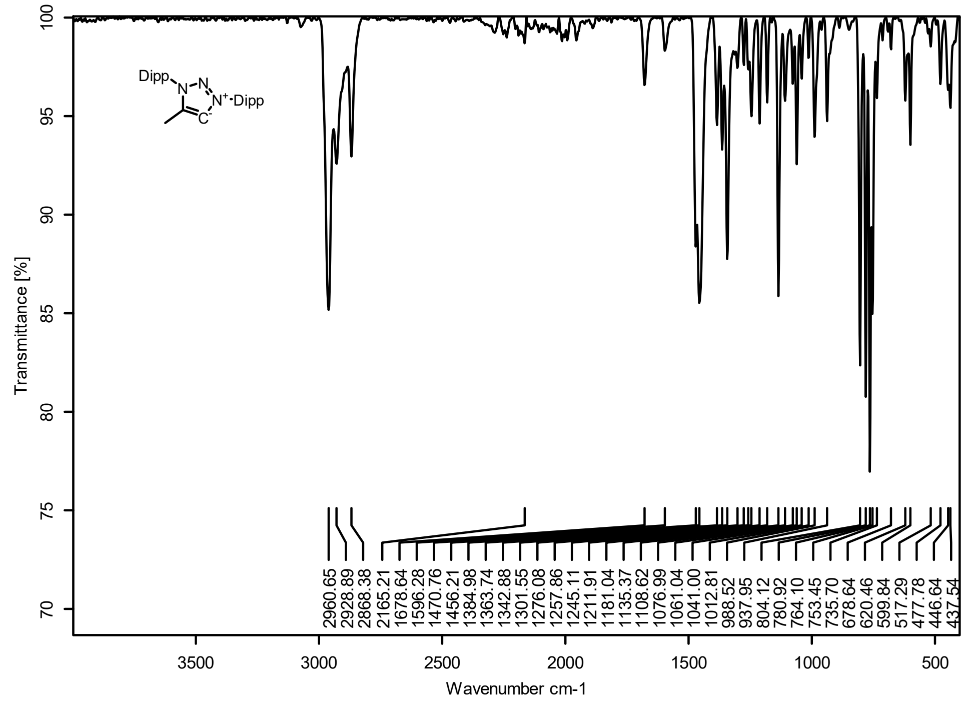

Fig. S84: ATR-IR spectrum (solid) of **2**.

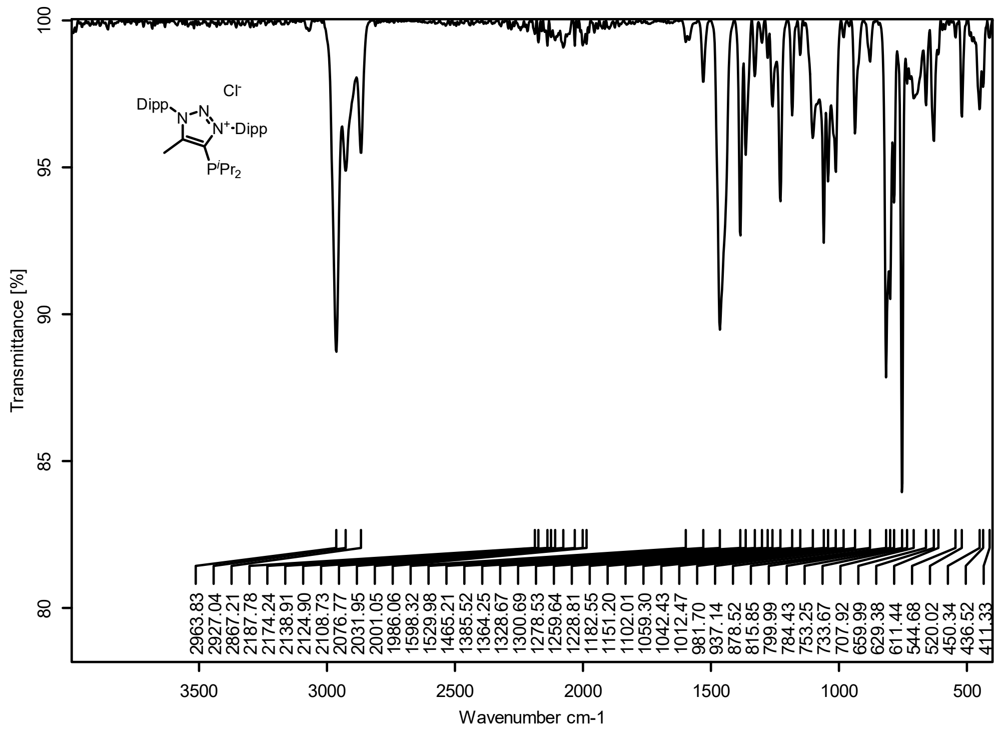

Fig. S85: ATR-IR spectrum (solid) of **3**.

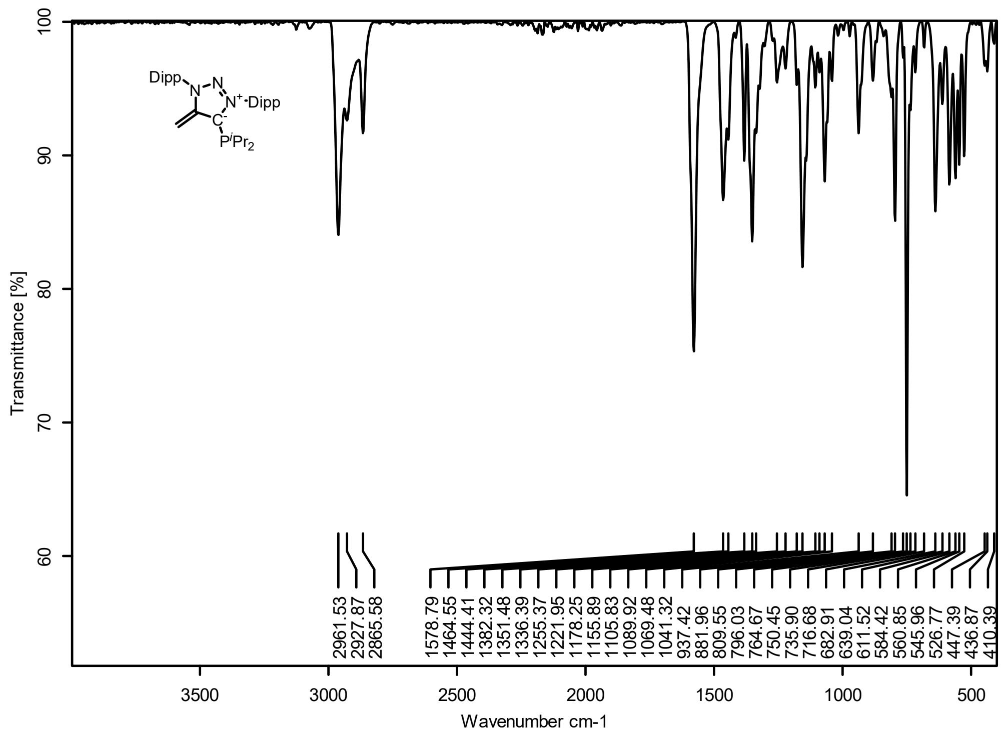

Fig. S86: ATR-IR spectrum (solid) of **4**.

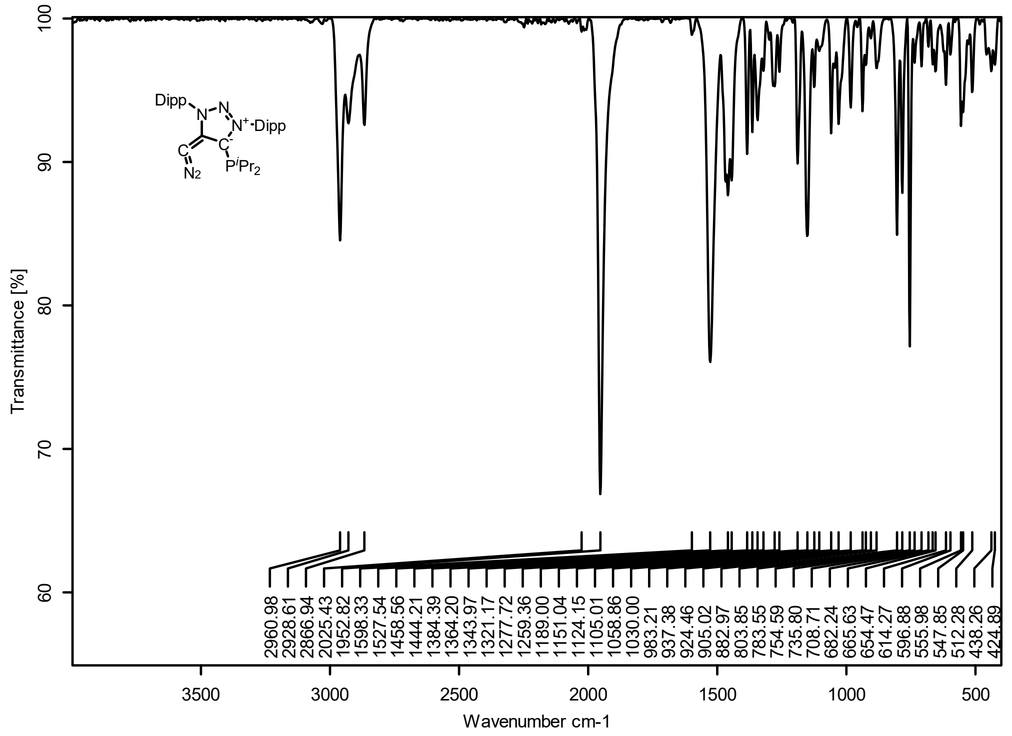

Fig. S87: ATR-IR spectrum (solid) of **5**.

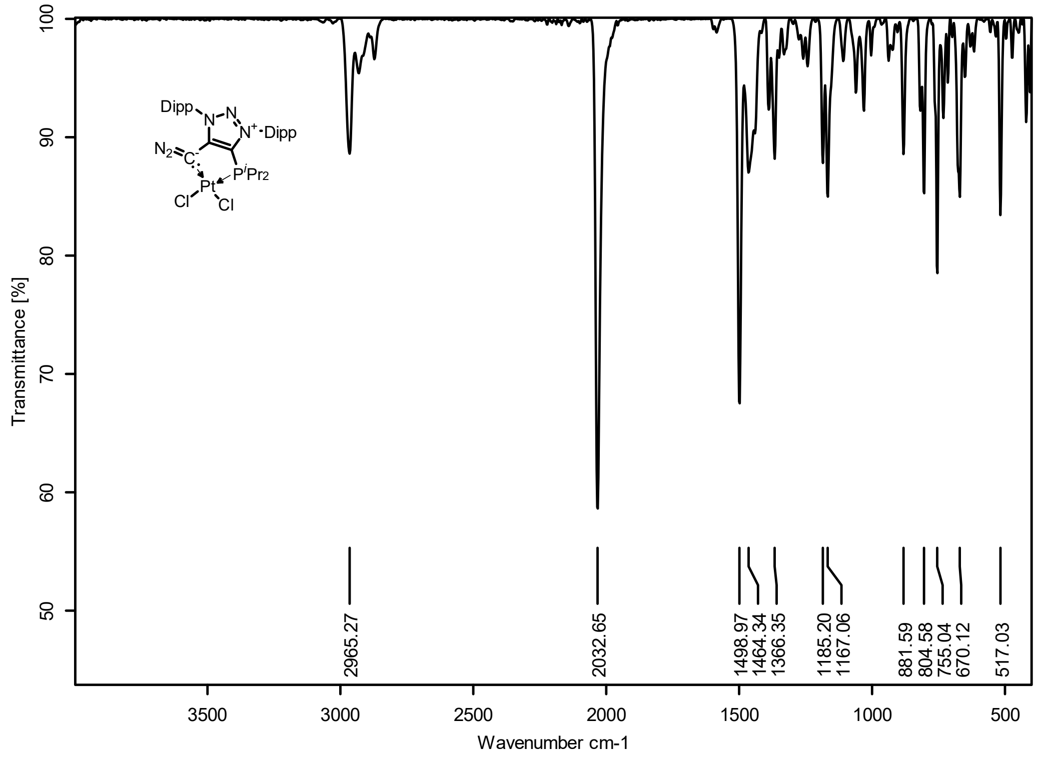

Fig. S88: ATR-IR spectrum (solid) of **6**.

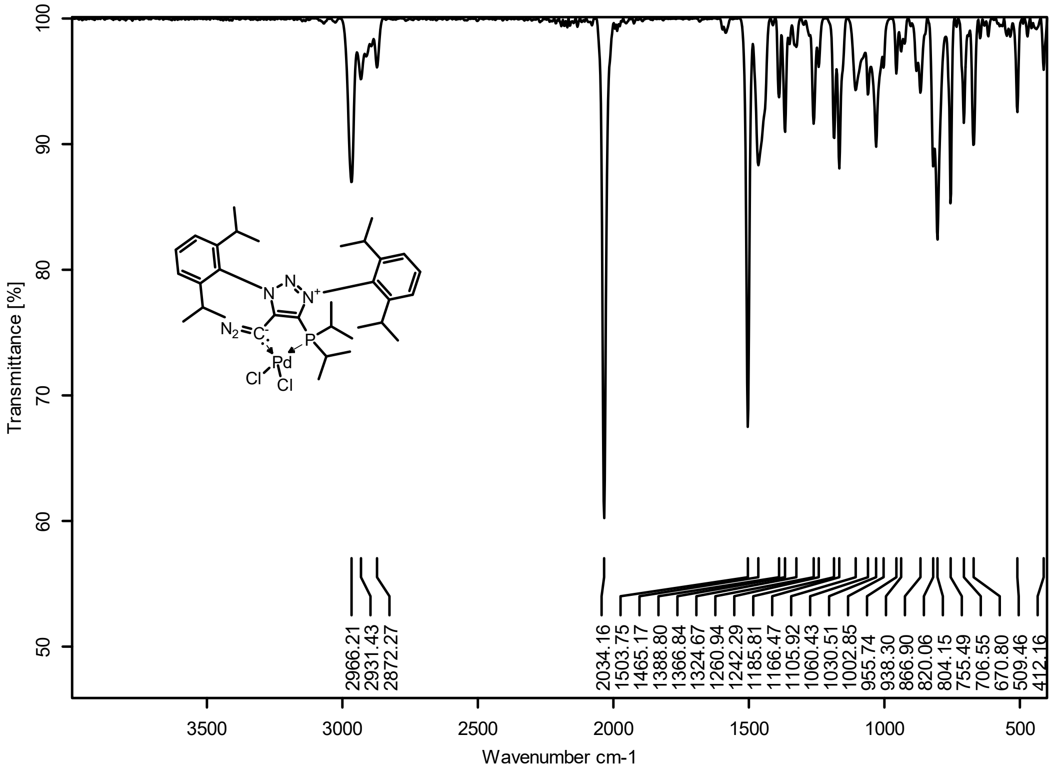

Fig. S89: ATR-IR spectrum (solid) of **7**.

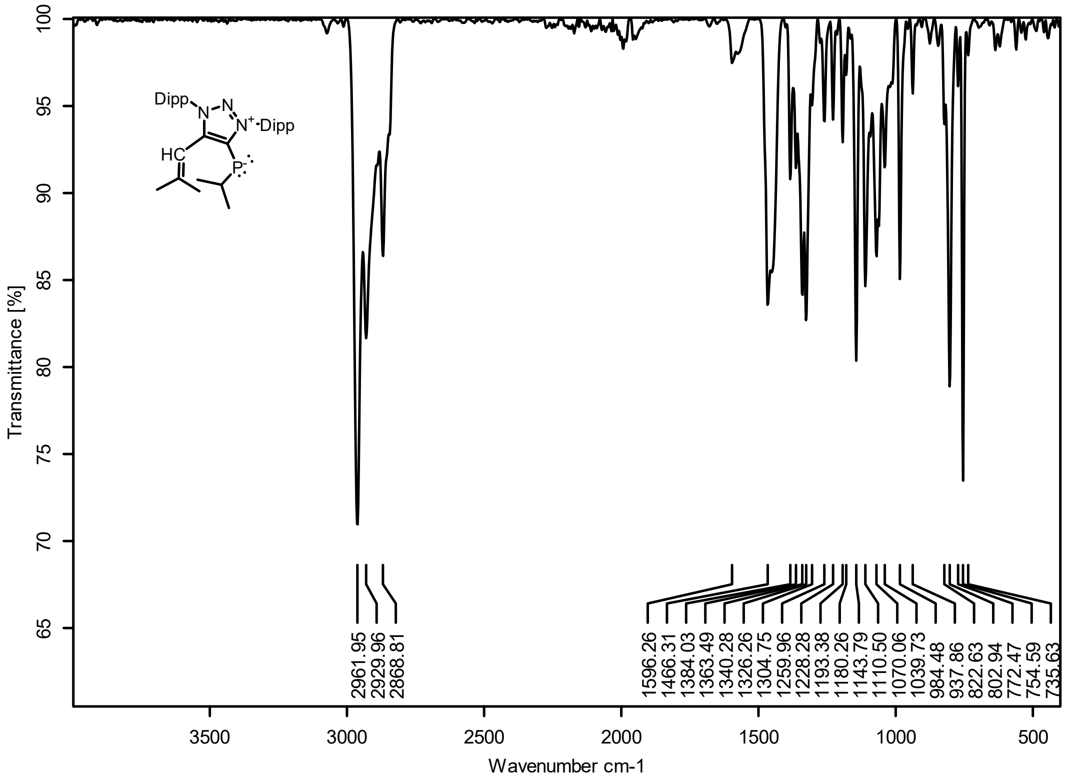

Fig. S90: ATR-IR spectrum (solid) of **11**.

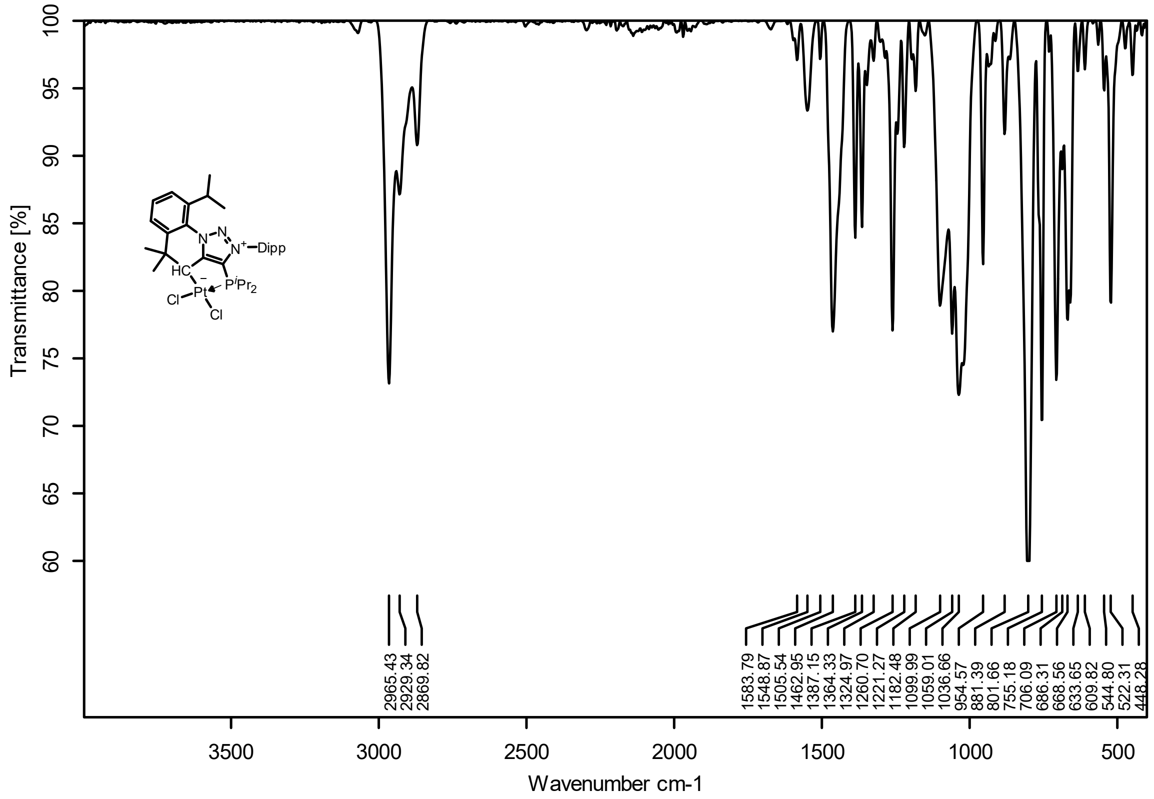

Fig. S91: ATR-IR spectrum (solid) of **12**.

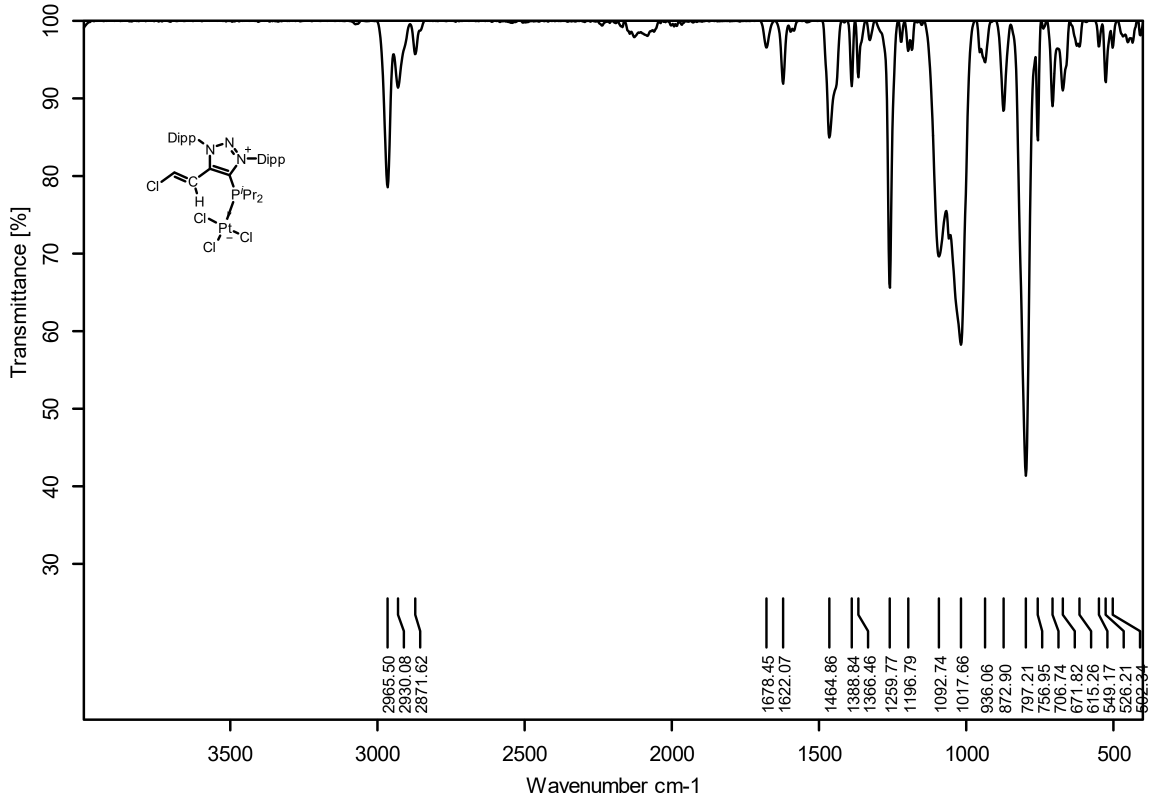

Fig. S92: ATR-IR spectrum (solid) of **13**.

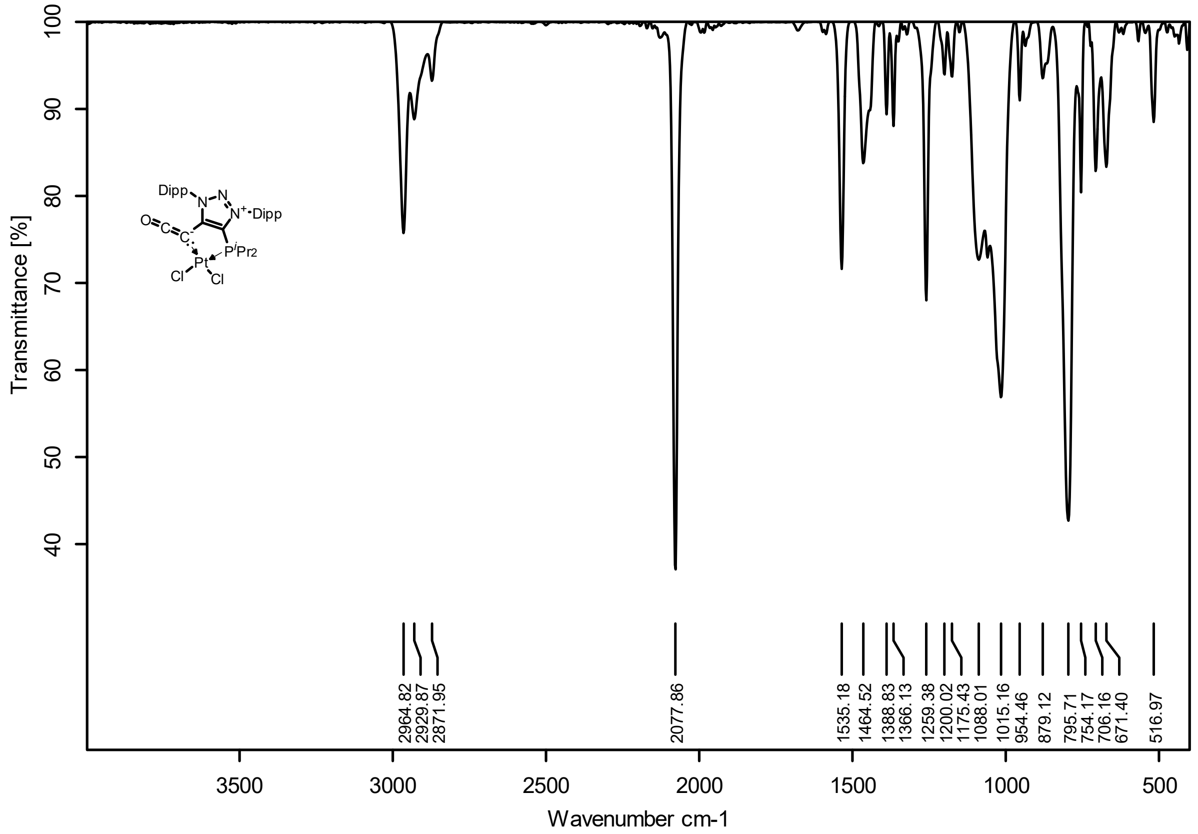

Fig. S93: ATR-IR spectrum (solid) of **14**.

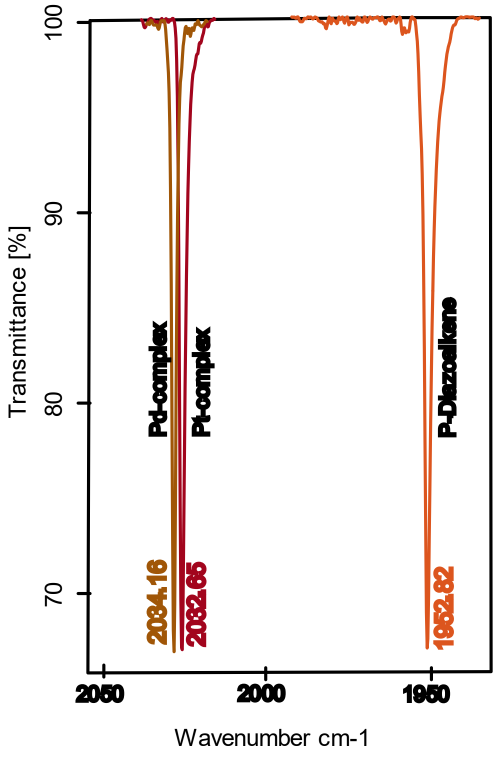

Fig. S94: ATR-IR spectrum (solid) Comparison of CN_2 stretch of **5** (orange)**, 6** (red) and **7** (brown).

5. UV-VIS

5.1 UV-VIS spectra

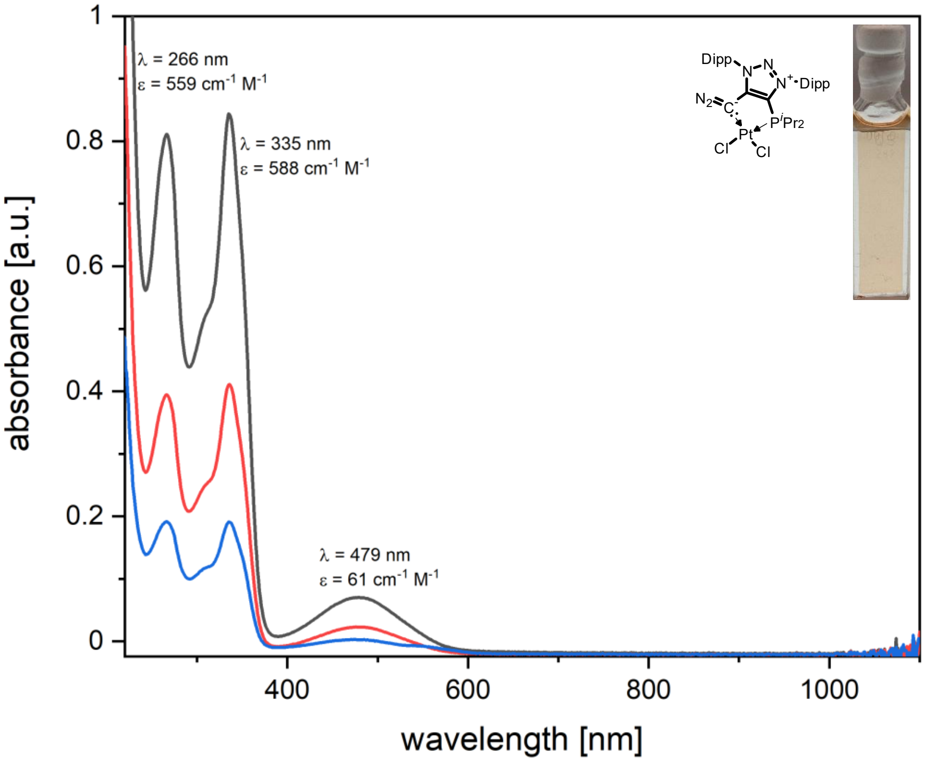

Fig. S95: UV-VIS-NIR spectra of **6** at 1.2 mg/mL (black), 0.6 mg/mL (red) and 0.3 mg/mL (blue) in CH_2Cl_2, 0.1 cm quartz cuvette.

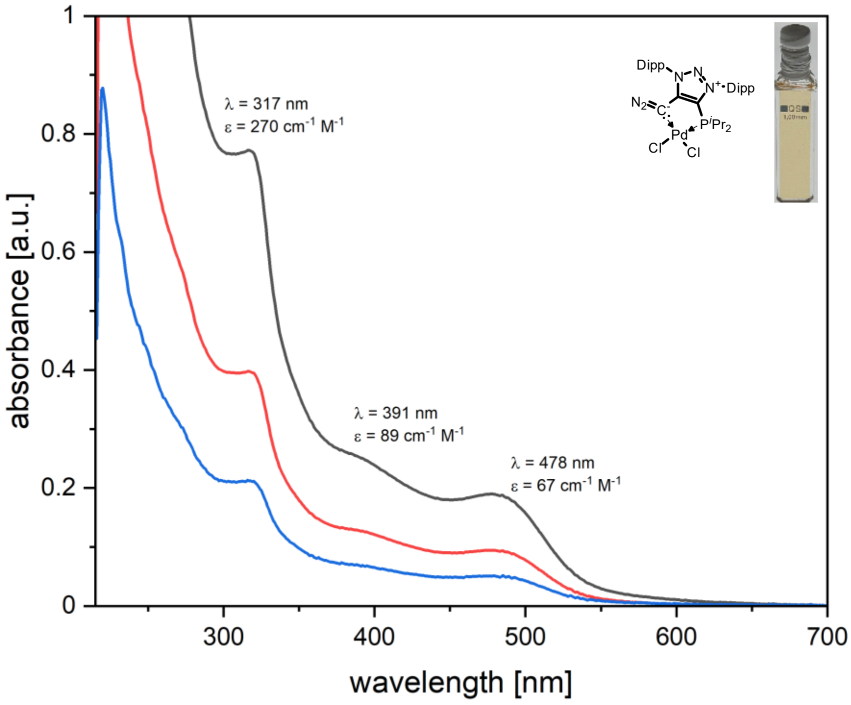

Fig. S96: UV-VIS-NIR spectra of **7** at 2.0 mg/mL (black), 1.0 mg/mL (red) and 0.5 mg/mL (blue) in CH_2Cl_2, 0.1 cm quartz cuvette.

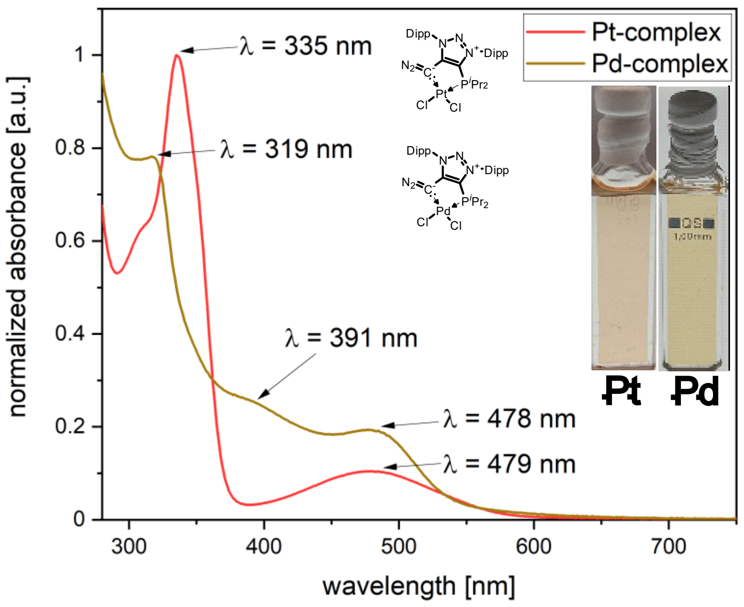

Fig. S97: Normalized UV-VIS-NIR spectra of **6** (red) at 0.6 mg/mL and of **7** (brown) at 1.0 mg/mL in CH_2Cl_2, 0.1 cm quartz cuvette.

5.2 VT UV-VIS

General: The UV-VIS spectrometer was cooled with the *UNISOKU Scientific Instruments - CoolSpeK UV* device. In a UV-VIS Cuvette (type *UNISOKU Scientific Instruemts – UnispeKs CoolSpeK* 1.0 x 1.0 cm) 1.2 mg of compound **6** was dissolved in 4 mL freshly distilled 1,3-difluorobenzene. Irradiation with a 455 nm glass fiber coupled LED were carried out at -45 °C under stirring in the UV-VIS spectrometer (see **Fig. S98**). During measurement the stirring and irradiation was stopped. After irradiation, the stability was checked every 2 minutes, followed by a warmup experiment (10 °C steps from -45 °C to 25 °C).

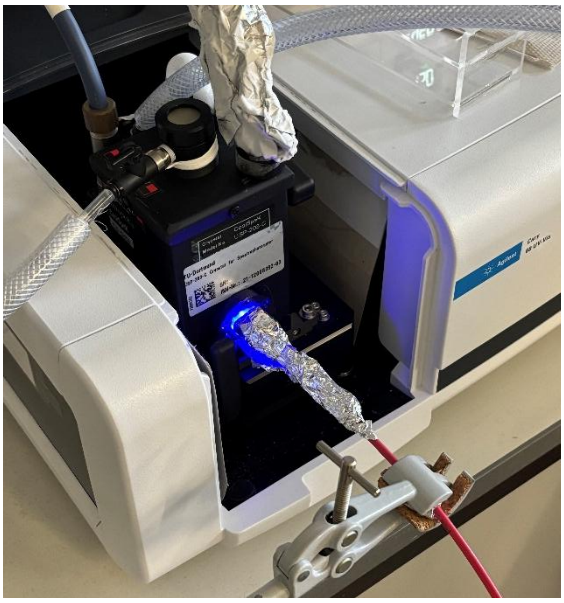

Fig S98: VT UV-VIS setup during irradiation.

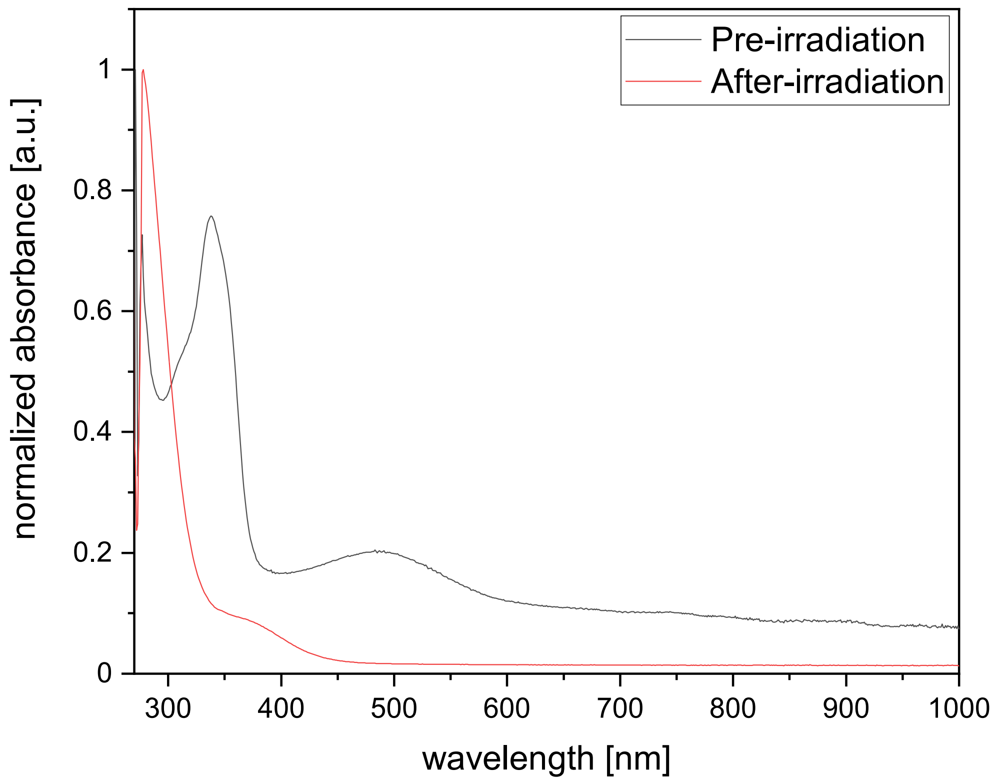

Fig. S99: Normalized UV-VIS spectra at r.t. of isolated **6** (black) and irradiation product (red) at 2.0 mg/mL in 1,3-difluorobenzene, 0.1 cm quartz cuvette.

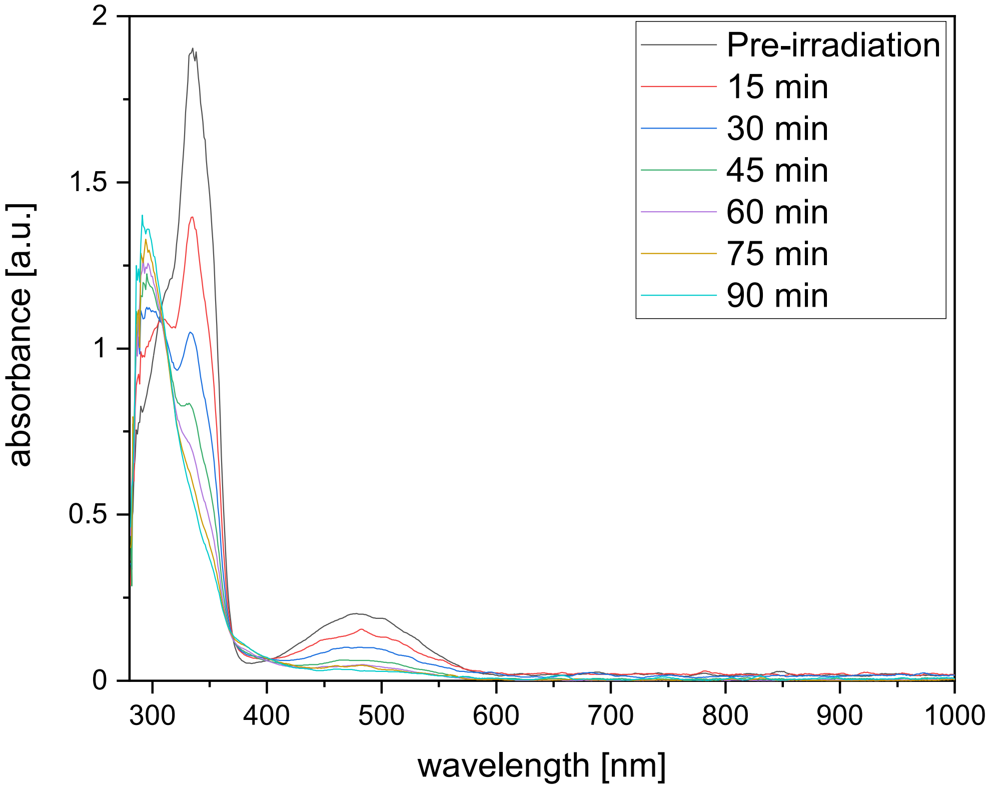

Fig. S100: VT UV-VIS irradiation experiment of **6** at -45 °C at 0.5 mg/mL in 1,3-difluorobenzene, 1.0 cm quartz cuvette.

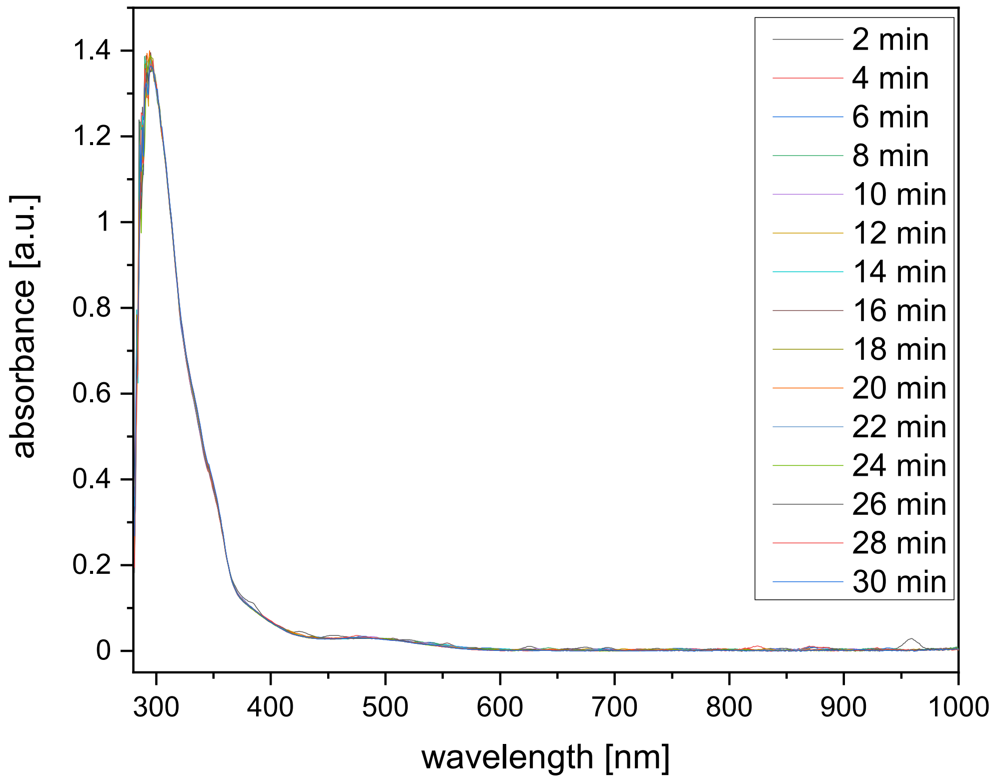

Fig. S101: VT UV-VIS after irradiation experiment of **6** for stability, measured every 2 min at -45 °C at 0.5 mg/mL in 1,3-difluorobenzene, 1.0 cm quartz cuvette.

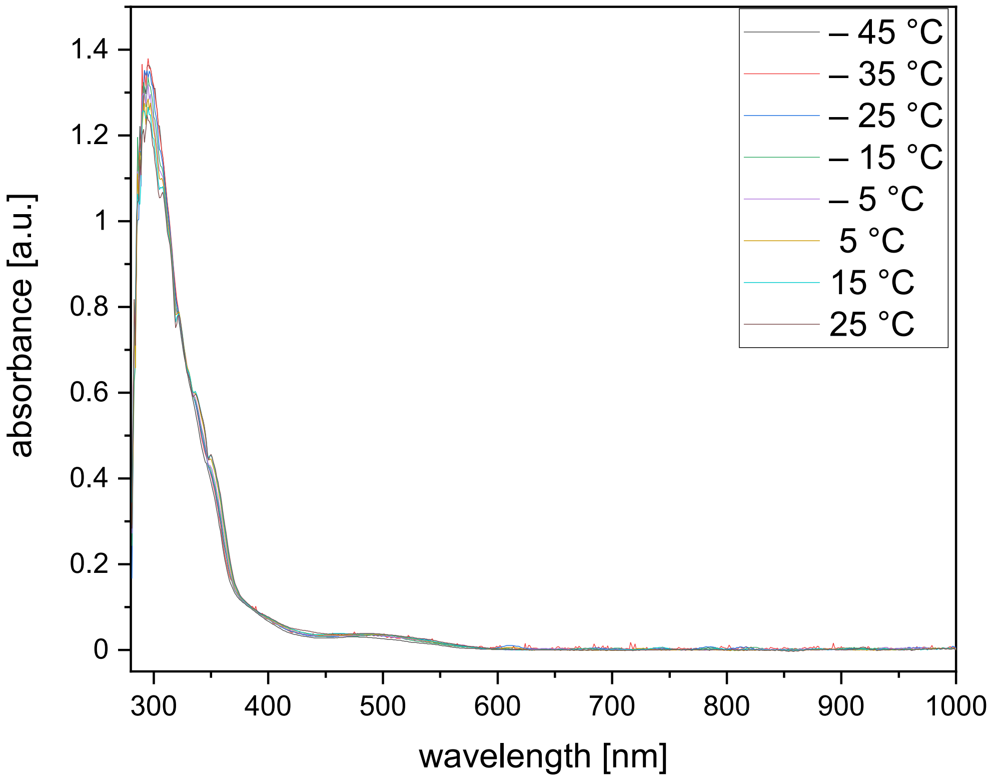

Fig. S102: VT UV-VIS warmup after irradiation experiment of **6** at -45 °C to 25 °C in 10 °C steps at 0.5 mg/mL in 1,3-difluorobenzene, 1.0 cm quartz cuvette.

6. SDT measurement

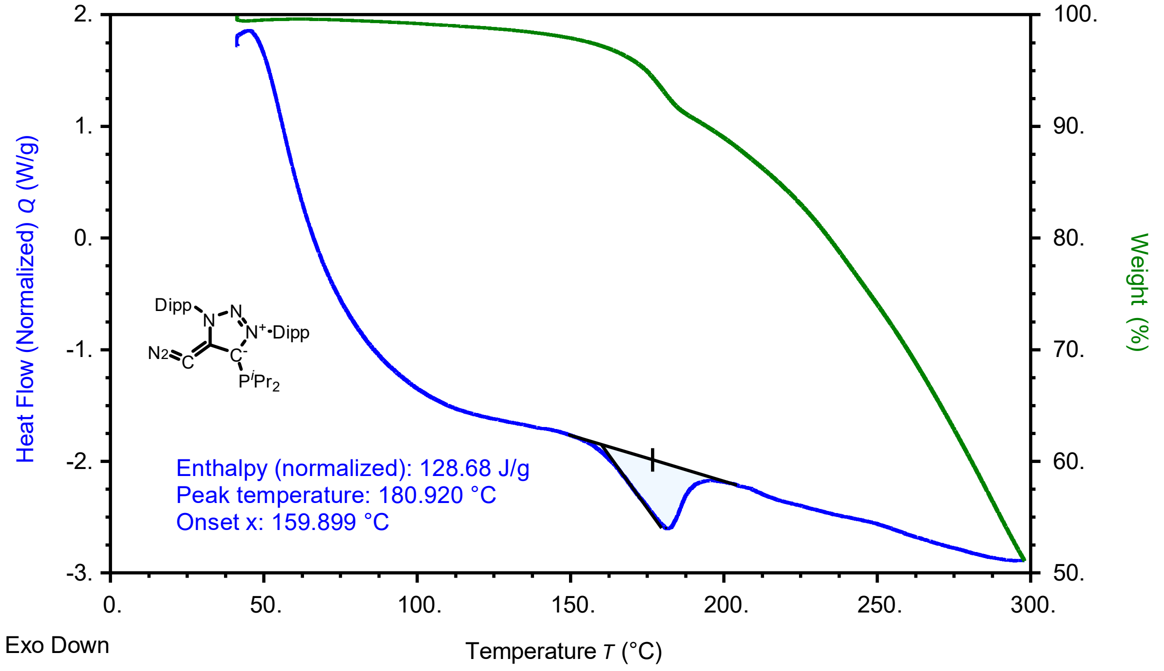

Fig. S103: SDT spectra of **5**.

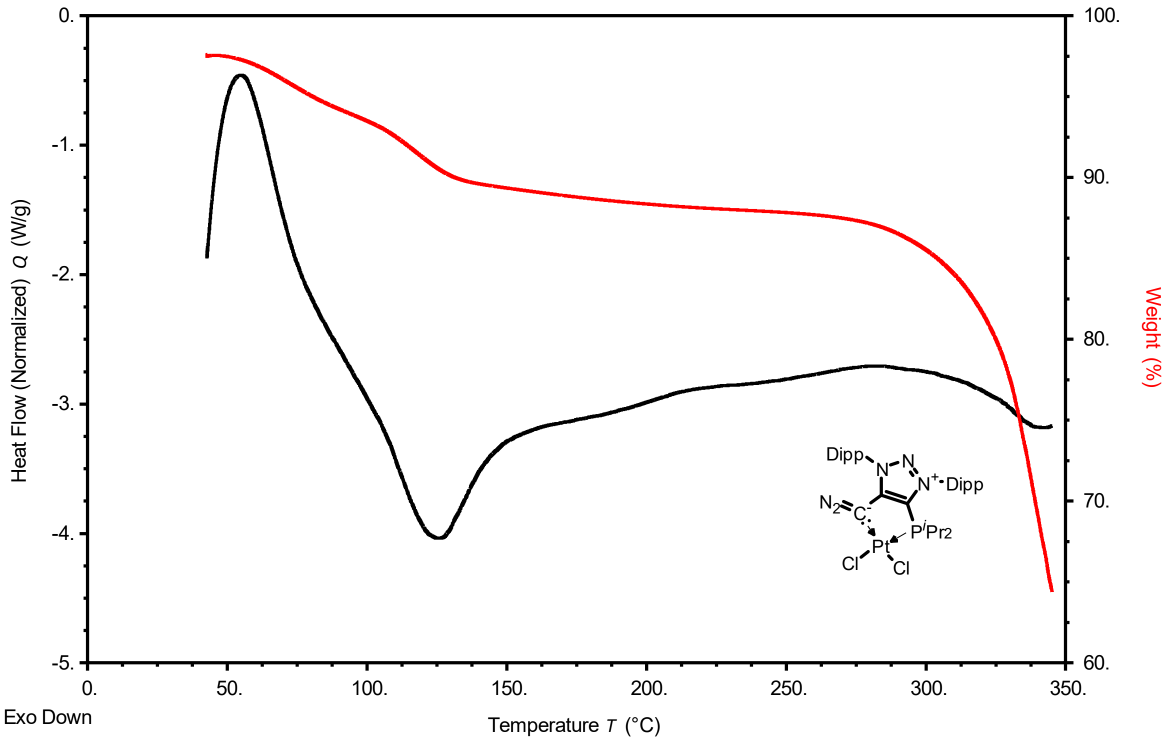

Fig. S104: SDT spectra of **6**.

7. TGA measurement

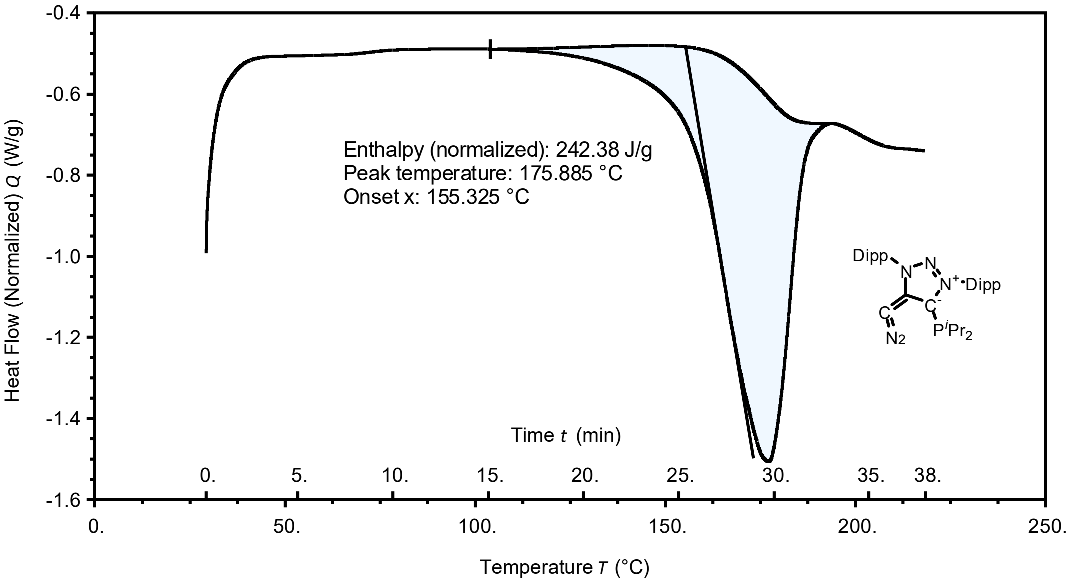

Fig. S105: TGA spectra of **5**.

8. X-ray characterization data

Crystallization conditions

Crystals of **5** were grown by slow cooling of saturated solution of compound in diethylether to -40°C in the glove box.

Crystals of **6** were grown by slow vapor diffusion of pentane into solution of compound in dichloromethane at -40°C in the glove box.

Crystals of **7** were grown by slow vapor diffusion of pentane into solution of compound in dichloromethane at -40°C in the glove box.

Crystals of **8** were grown by slow vapor diffusion of pentane into solution of compound in dichloromethane at -40°C in the glove box and subsequent in crystallo irradiation at -173°C on the diffractometer.

Crystals of **11** were grown by slow evaporation from pentane at -40°C in the glove box.

Crystals of **12** were grown by adding a solution of compound in pentane, o-dichlorobenzene and THF to a drop of NVH oil in the glove box a room temperature and thereby allowing the solvents to slowly diffuse into the NVH oil.

Crystals of **13** were grown by slow evaporation of saturated solution of compound in dichloromethane, pentane and a drop of benzene(d6) at room temperature in the glove box.

Crystals of **14** were grown by slow vapor diffusion of pentane into solution of compound in dichloromethane with two drops of o-dichlorobenzene at room temperature in the glove box.

Data collection

Diffraction data of single-crystals grown from compounds **5**, **6**, **8**, **11**, **12**, **13** and **14** were collected from flash-cooled single crystals at 100(2) K on a Bruker D8 VENTURE dual wavelength Mo/Cu or Ag/Cu four-circle diffractometer with a microfocus sealed X-ray tube using mirror optics as monochromator and a Bruker PHOTON II or PHOTON III detector. The diffractometer was equipped with a Oxford Cryostream 800 or 1000 low temperature device. All data were integrated with SAINT V8.40B and a multi-scan absorption correction using SADABS-2016/2 was applied.

Due to very thin needle-shaped crystals, the analysis of **7** was hampered by the limited scattering power of the samples not allowing to reach the desired atomic resolution using our modern microfocussed X-ray in-house CuK_α source. Gaining detailed structural insight thus required cryogenic crystal handling and highly brilliant synchrotron radiation. The crystal was quickly mounted onto a 50 µm Mitegen dual thickness MicroLoop LD™ and immediately flash-cooled in liquid

nitrogen. Crystals were stored at cryogenic temperature in dry shippers, in which they were safely transported to macromolecular beamline P11 at Petra III[3], DESY, Hamburg, Germany. A wavelength of λ = 0.688 Å was chosen using a liquid N_2 cooled double crystal monochromator. Single crystal X-ray diffraction data was collected at 100(2) K on a single axis goniometer, equipped with an Oxford Cryostream 1000 and an Eiger 2x 16M detector. 3600 diffraction images were collected in a 360° φ sweep at a detector distance of 154 mm, 100% filter transmission, 200 x 200 µm beam focus, 0.1° step width and 10 milliseconds exposure time per image. Data integration and reduction were undertaken using XDS.[4]

All structures were solved by intrinsic phasing/direct methods using SHELXT[5] and refined with SHELXL[6] using 22 CPU cores for full-matrix least-squares routines on F^2 and ShelXle[7] as a graphical user interface. All non-hydrogen atoms were refined with anisotropic displacement parameters. The hydrogen atoms were refined isotropically on calculated positions using a riding model with their U_{iso} values constrained to 1.5 times the U_{eq} of their pivot atoms for terminal sp^3 carbon atoms and 1.2 times for all other carbon atoms. Crystallographic data (including structure factors) for the structures reported in this paper have been deposited with the Cambridge Crystallographic Data Centre. CCDC 2454873-2454880 contain the supplementary crystallographic data for this paper. Copies of the data can be obtained free of charge from The Cambridge Crystallographic Data Centre via www.ccdc.cam.ac.uk/structures.

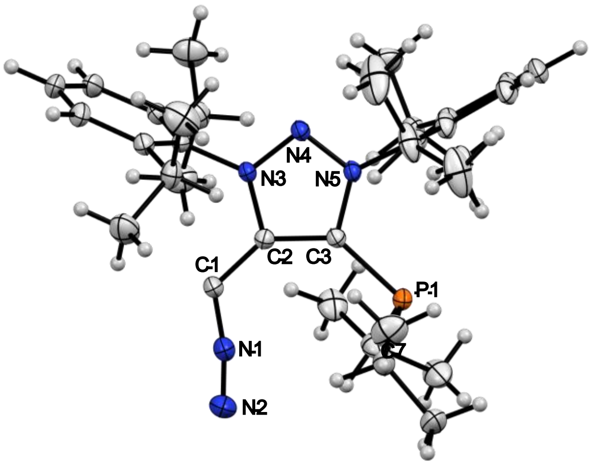

Fig. S106: X-ray solid-state structure of compound **5**. Thermal ellipsoids are shown with 50 % probability. Disordered solvent molecules (Et_2O) were omitted for clarity. Selected bond parameters in [Å] and [°]: P1–C3 1.8213(13), C1–C2 1.3951(16), C2–C3 1.4216(16), N1–C1 1.2660(18), N1–N2 1.1551(17), N2–N1–C1 169.08(13), N1–C1–C2 123.55(12).

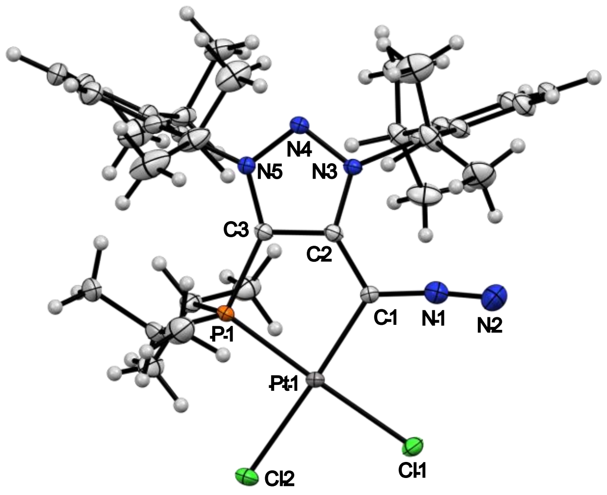

Fig. S107: X-ray solid-state structure of compound **6**. Thermal ellipsoids are shown with 50 % probability. A solvent molecule (CH_2Cl_2) was omitted for clarity. Selected bond parameters in [Å] and [°]: P1–C3 1.826(3), P1–Pt1 2.2135(7), C1–Pt1 2.007(3), C1–C2 1.422(4), C2–C3 1.389(4), N1–C1 1.324(4), N1–N2 1.154(4), Pt1–Cl1 2.3497(7), Pt1–Cl2 2.3571(7), N2–N1–C1 173.1(3), N1–C1–C2 118.8(3), C2–C1–Pt1 117.1(2), N1–C1–Pt1 124.1(2), C1–Pt1–P1 86.30(8), C3–P1–Pt1 100.51(10), C1–Pt1–Cl2 176.00(9), P1–Pt1–Cl1 176.92(3).

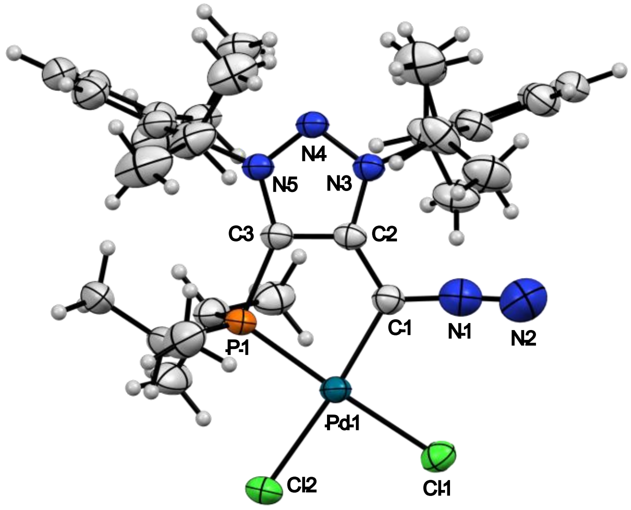

Fig. S108: X-ray solid-state structure of compound **7**. Thermal ellipsoids are shown with 50 % probability. A solvent molecule (CH_2Cl_2) was omitted for clarity. Selected bond parameters in [Å] and [°]: P1–C3 1.823(3), P1–Pd1 2.2408(14), C1–Pd1 1.994(4), C1–C2 1.413(5), C2–C3 1.389(5), N1–C1 1.374(6), N1–N2 1.199(7), Pd1–Cl1 2.3454(14), Pd1–Cl2 2.3575(14), N2–N1–C1 175.5(5), N1–C1–C2 119.3(3), C2–C1–Pd1 118.5(3), N1–C1–Pd1 122.2(3), C1–Pd1–P1 85.37(13), C3–P1–Pd1 100.45(13), C1–Pd1–Cl2 174.30(11), P1–Pd1–Cl1 176.55(3).

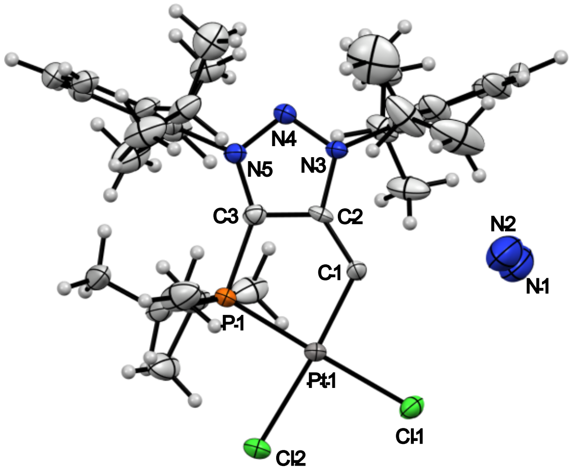

Fig. S109: X-ray solid-state structure of compound **8**. Thermal ellipsoids are shown with 50 % probability. Solvent molecules (CH_2Cl_2) and the second molecule of **8** in the unit cell were omitted for clarity. Selected bond parameters in [Å] and [°]: P1–C3 1.842(8), P1–Pt1 2.234(2), C1–Pt1 1.918(8), C1–C2 1.397(11), C2–C3 1.390(12), N1–N2 1.005(16), Pt1–Cl1 2.352(2), Pt1–Cl2 2.378(2), C2–C1–Pt1 120.8(6), C1–Pt1–P1 85.2(2), C3–P1–Pt1 100.7(3), C1–Pt1–Cl2 174.6 (2), P1–Pt1–Cl1 177.86(7).

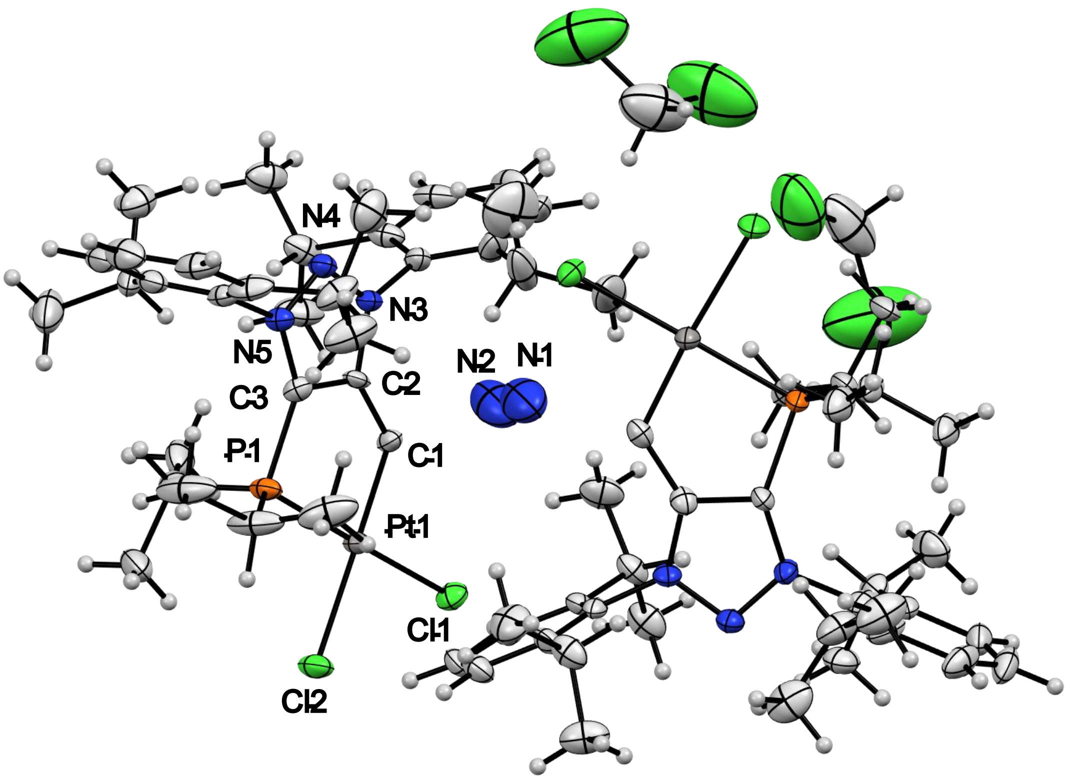

Fig. S110: X-ray solid-state structure of compound **8**. Thermal ellipsoids are shown with 50 % probability.

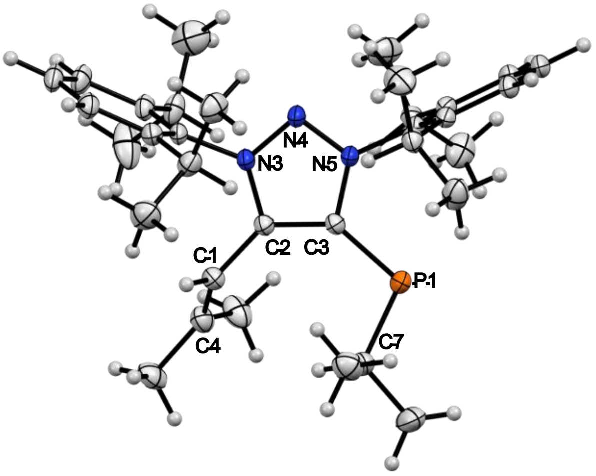

Fig. S111: X-ray solid-state structure of compound **11**. Thermal ellipsoids are shown with 50 % probability. Disordered solvent molecules (pentane) were omitted for clarity. Selected bond parameters in [Å] and [°]: P1–C3 1.7807(13), P1–C7 1.8717(14), C1–C2 1.4694(17), C1–C4 1.3337(19), C2–C3 1.4187(16).

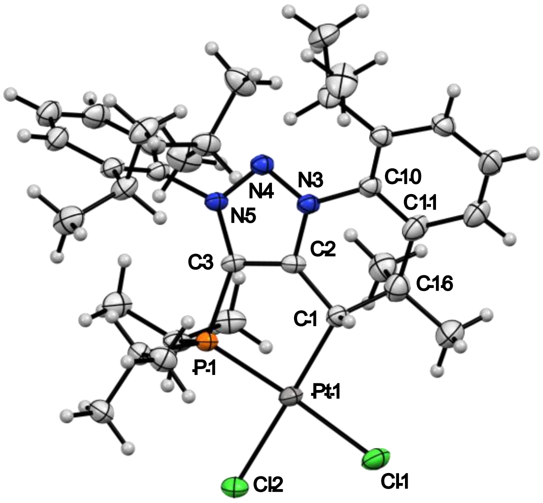

Fig. S112: X-ray solid-state structure of compound **12**. Thermal ellipsoids are shown with 50 % probability. A solvent molecule (pentane and *ortho*-dichlorobenzene) was omitted for clarity. Selected bond parameters in [Å] and [°]: C1–C2 1.475(4), C1–Pt1 2.077(3), P1–Pt1 2.1986(8), Pt1–Cl1 2.3654(8), Pt1–Cl2 2.3910(8), C2–C3 1.370(4), C3–P1 1.832(3), C1–C16 1.584(5), C11–C16 1.570(5), C10–C11 1.404(4), C2–C1–Pt1 107.9(2), C1–Pt1–P1 90.32(9), C1–Pt1–Cl2 178.88(9), P1–Pt1–Cl1 173.45(3), C2–C1–C16 101.9(3), C16–C1–Pt1 126.5(2), C10–C11–C16 121.1(3), C11–C16–C1 106.9(3).

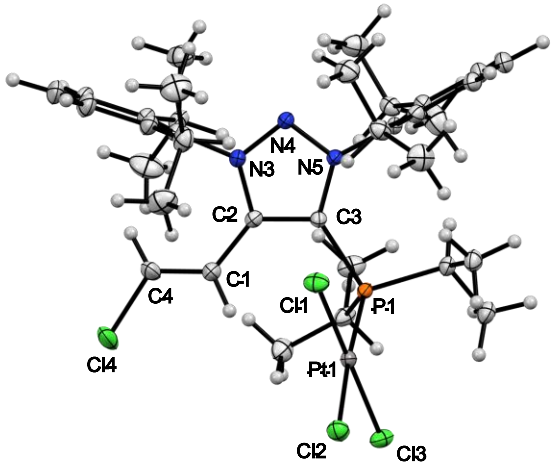

Fig. S113: X-ray solid-state structure of compound **13**. Thermal ellipsoids are shown with 50 % probability. A solvent molecule (CH_2Cl_2) was omitted for clarity. Selected bond parameters in [Å] and [°]: C1–C2 1.455(4), C1–C4 1.328(4), C4–Cl4 1.722(3), C2–C3 1.394(3), C3–P1 1.860(2), P1–Pt1 2.2137(7), Pt1–Cl1 2.3041(7), Pt1–Cl2 2.3443(7), Pt1–Cl3 2.3161(6).

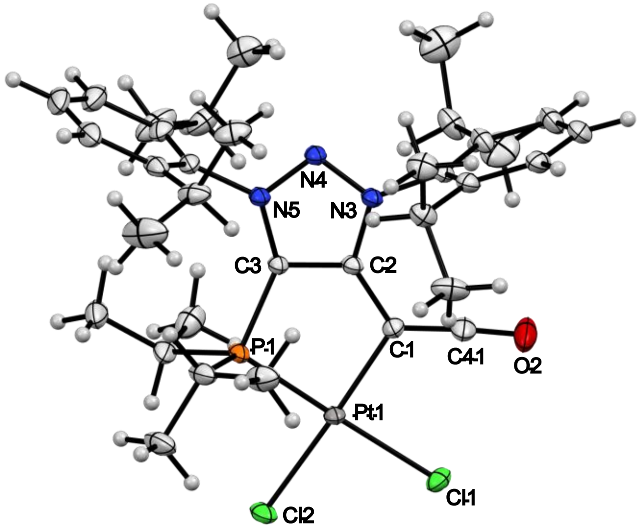

Fig. S114: X-ray solid-state structure of compound **14**. Thermal ellipsoids are shown with 50 % probability. Selected bond parameters in [Å] and [°]: C1–C2 1.433(5), C1–Pt1 2.044(2), C1–C41 1.313(5), C41–O2 1.167(5), P1–Pt1 2.207(1), Pt1–Cl1 2.352(1), Pt1–Cl2 2.3528(6), C2–C3 1.389(4), C3–P1 1.826(2), C2–C1–Pt1 114.7(2), C1–Pt1–P1 87.09(8), C1–Pt1–Cl2 177.27(9), P1–Pt1–Cl1 177.39(4), Pt1–C1–C41 122.1(2). C1–C41–O2 176.1(4).

Crystallographic Tables

Compound	**5**	**6**	**7**
CIF ID	**ma4_028e**	**ma3_189f**	**ma429**
CCDC number	2454873	2454874	2454875
Empirical formula	$C_{37}H_{58}N_5OP$	$C_{34}H_{50}Cl_4N_5PPt$	$C_{34}H_{50}Cl_4N_5PPd$
Formula weight	619.85	896.65	807.96
Temperature [K]	100(2)	100(2)	100(2)
Crystal system	monoclinic	monoclinic	monoclinic
Space group (number)	$P2_1/n$ (14)	$C2/c$ (15)	$C2/c$ (15)
a [Å]	12.4845(3)	34.0332(7)	34.30(2)
b [Å]	18.8823(5)	12.3586(2)	12.337(7)
c [Å]	15.6968(4)	24.6920(5)	24.668(19)
α [°]	90	90	90
β [°]	95.754(2)	129.8200(10)	130.100(7)
γ [°]	90	90	90
Volume [Å^3]	3681.66(16)	7976.7(3)	7984(9)
Z	4	8	8
ρ_{calc} [gcm^{-3}]	1.118	1.493	1.344
μ [mm^{-1}]	0.914	3.855	0.726
F(000)	1352	3600	3344
Crystal size [mm^3]	0.300×0.050×0.040	0.200×0.100×0.040	0.080×0.080×0.005
Crystal colour	orange	orange	yellow
Crystal shape	block	block	plate
Radiation	CuK_α (λ=1.54178 Å)	MoK_α (λ=0.71073 Å)	synchrotron (λ=0.68879 Å)
2Θ range [°]	7.35 to 158.66 (0.78 Å)	5.72 to 56.63 (0.75 Å)	3.01 to 55.46 (0.74 Å)
Index ranges	-15 ≤ h ≤ 15	-45 ≤ h ≤ 45	-44 ≤ h ≤ 45
	-22 ≤ k ≤ 24	-16 ≤ k ≤ 16	-15 ≤ k ≤ 15
	-19 ≤ l ≤ 19	-32 ≤ l ≤ 32	-32 ≤ l ≤ 32
Reflections collected	54783	67139	49450
Independent reflections	7795	9923	8313
	R_{int} = 0.0725	R_{int} = 0.0597	R_{int} = 0.0653
	R_{sigma} = 0.0345	R_{sigma} = 0.0358	R_{sigma} = 0.0421
Completeness	100.0 %	99.9 %	97.8 %
Data / Restraints / Parameters	7795/14/459	9923/0/418	8313/621/418
Goodness-of-fit on F^2	1.038	1.052	1.081
Final R indexes	R_1 = 0.0384	R_1 = 0.0274	R_1 = 0.0551
[I≥2σ(I)]	wR_2 = 0.0953	wR_2 = 0.0609	wR_2 = 0.1644
Final R indexes	R_1 = 0.0521	R_1 = 0.0348	R_1 = 0.0605
[all data]	wR_2 = 0.1023	wR_2 = 0.0649	wR_2 = 0.1696
Largest peak/hole [eÅ^{-3}]	0.29/-0.28	1.45/-1.30	1.36/-1.25
Flack X parameter			
Extinction coefficient			

Compound	**8**	**11**	**12**
CIF ID	**ma3_189f_hv_cc_sq**	**ma2_101p**	**ma4_059tp**
CCDC number	2454876	2454877	2454878
Empirical formula	$C_{68}H_{100}Cl_8N_8P_2Pt_2$	$C_{71}H_{108}N_6P_2$	$C_{44}H_{64}Cl_4N_3PPt$
Formula weight	1765.27	1107.57	1002.84
Temperature [K]	100(2)	100(2)	100(2)
Crystal system	monoclinic	triclinic	monoclinic
Space group (number)	Cc (9)	$P\bar{1}$ (2)	$C2/c$ (15)
a [Å]	34.5670(12)	10.7330(6)	33.0683(12)
b [Å]	12.4036(4)	11.8918(7)	12.2994(4)
c [Å]	24.8248(15)	15.2778(9)	27.0644(16)
α [°]	90	67.604(2)	90
β [°]	130.5580(10)	75.641(2)	123.5900(10)
γ [°]	90	89.734(2)	90
Volume [Å^3]	8086.6(6)	1737.77(18)	9169.6(7)
Z	4	1	8
ρ_{calc} [gcm^{-3}]	1.450	1.058	1.453
μ [mm^{-1}]	3.777	0.105	8.442
F(000)	3544	606	4080
Crystal size [mm^3]	0.200×0.100×0.040	0.608×0.389×0.176	0.300×0.050×0.050
Crystal colour	yellow	orange	colourless
Crystal shape	block	block	needle
Radiation	MoK_α (λ=0.71073 Å)	MoK_α (λ=0.71073 Å)	CuK_α (λ=1.54178 Å)
2Θ range [°]	4.72 to 59.27 (0.72 Å)	5.12 to 55.02 (0.77 Å)	6.42 to 158.58 (0.78 Å)
Index ranges	-48 ≤ h ≤ 48<br>-17 ≤ k ≤ 16<br>-34 ≤ l ≤ 34	-13 ≤ h ≤ 13<br>-15 ≤ k ≤ 15<br>-19 ≤ l ≤ 19	-40 ≤ h ≤ 42<br>-14 ≤ k ≤ 15<br>-34 ≤ l ≤ 34
Reflections collected	153516	51817	78770
Independent reflections	22752<br>R_{int} = 0.0687<br>R_{sigma} = 0.0458	7965<br>R_{int} = 0.0282<br>R_{sigma} = 0.0202	9742<br>R_{int} = 0.0744<br>R_{sigma} = 0.0328
Completeness	99.9 %	99.9 %	100.0 %
Data / Restraints / Parameters	22752/116/818	7965/113/441	9742/0/492
Goodness-of-fit on F^2	1.033	0.976	1.037
Final *R* indexes [*I*≥2σ(*I*)]	R_1 = 0.0355<br>wR_2 = 0.0787	R_1 = 0.0459<br>wR_2 = 0.1266	R_1 = 0.0295<br>wR_2 = 0.0747
Final *R* indexes [all data]	R_1 = 0.0441<br>wR_2 = 0.0833	R_1 = 0.0506<br>wR_2 = 0.1311	R_1 = 0.0366<br>wR_2 = 0.0785
Largest peak/hole [eÅ^{-3}]	1.66/-0.78	0.39/-0.38	1.47/-0.91
Flack X parameter	0.244(6)		
Extinction coefficient			

Compound	**13**	**14**
CIF ID	**ma4_058dbp**	**ma476dbp_sq**
CCDC number	2454879	2454880
Empirical formula	$C_{35}H_{52}Cl_6N_3PPt$	$C_{34}H_{48}Cl_2N_3OPPt$
Formula weight	953.55	811.71
Temperature [K]	100(2)	100(2)
Crystal system	monoclinic	monoclinic
Space group (number)	$P2_1/n$ (14)	$C2/c$ (15)
a [Å]	14.3657(5)	34.190(2)
b [Å]	15.2572(6)	12.3772(8)
c [Å]	18.3780(7)	24.7343(13)
α [°]	90	90
β [°]	99.0640(10)	130.096(2)
γ [°]	90	90
Volume [Å^3]	3977.8(3)	8007.0(8)
Z	4	8
ρ_{calc} [gcm^{-3}]	1.592	1.347
μ [mm^{-1}]	3.999	3.705
F(000)	1912	3264
Crystal size [mm^3]	0.200×0.100×0.030	0.150×0.050×0.030
Crystal colour	colourless	yellow
Crystal shape	block	block
Radiation	MoK_α (λ=0.71073 Å)	MoK_α (λ=0.71073 Å)
2Θ range [°]	5.34 to 66.37 (0.65 Å)	4.31 to 72.73 (0.60 Å)
Index ranges	-22 ≤ h ≤ 22<br>-23 ≤ k ≤ 23<br>-27 ≤ l ≤ 28	-55 ≤ h ≤ 56<br>-18 ≤ k ≤ 20<br>-40 ≤ l ≤ 39
Reflections collected	111743	133500
Independent reflections	14995<br>R_{int} = 0.0574<br>R_{sigma} = 0.0374	18250<br>R_{int} = 0.0608<br>R_{sigma} = 0.0461
Completeness	99.9 %	99.8 %
Data / Restraints / Parameters	14995/0/427	18250/0/391
Goodness-of-fit on F^2	1.027	1.041
Final *R* indexes [I≥2σ(I)]	R_1 = 0.0307<br>wR_2 = 0.0646	R_1 = 0.0365<br>wR_2 = 0.0673
Final *R* indexes [all data]	R_1 = 0.0541<br>wR_2 = 0.0752	R_1 = 0.0599<br>wR_2 = 0.0756
Largest peak/hole [eÅ^{-3}]	1.98/-1.07	2.07/-2.09
Flack X parameter		
Extinction coefficient		

In crystallo photoreaction of compound 6

After structure determination of compound **6**, the crystal was exposed to light irradiation (wavelength of 455 nm) with a fiber coupled 30 W LED, which was running at 600 mA (60% of the energy output), while the goniometer phi angle was regularly turned. The *in crystallo* reaction yielded a lower space group symmetry changing from monoclinic space group *C*2/c containing one molecule of the reaction educt **6** in the asymmetric unit to monoclinic space group *C*c with two molecules of the reaction product **8** as well as one (out of two) N_2 gas molecules and one extra void space. As indicated by the reaction product **8** the *in crystallo* reaction appears to be quantitative, the second N_2 gas molecule could not be modeled with discrete atomic positions in the extra void space of the asymmetric unit (see Fig. S110).

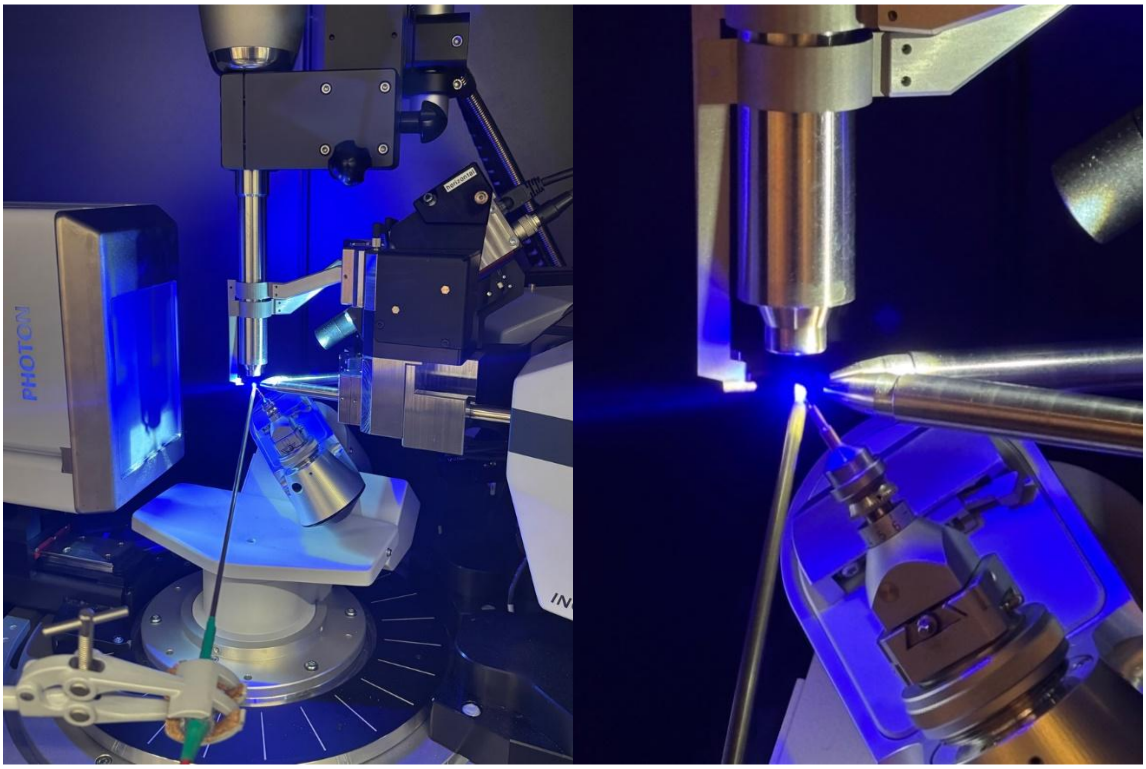

Fig. S115: Irradiation setup with fiber optic cable. After structure determination of **6** the in crystallo photoreaction to **8** is performed, while the crystal is cooled at 100 K and protected in N_2 cryostream on the goniometer. Structure determination of **8** was subsequently performed on the same crystal. Left: overview, right: close-up.

Specific refinement details for *in crystallo* photoreaction product **8**

The vinylidene structure could be successfully refined in monoclinic space group Cc, so that the symmetry in the crystal was reduced by the photoreaction, yielding two vinylidene molecules in the asymmetric unit. No significant residual could be found on the previous positions N1–N2 of

the reacted N_2 molecule, so that the compound decomposition photoreaction reaction appears to be quantitative. However, only one of the two expected N_2 molecules from the two product molecules **8** could be identified and successfully modelled in the asymmetric unit.

The refinement of ADP's for carbon, nitrogen and oxygen atoms was enabled by a combination of similarity restraints (SIMU) and rigid bond restraints (RIGU).[8] The second N_2 molecule was likely disordered, so that it could not be modeled with discrete atomic positions. It was instead handled using the SQUEEZE[9] routine in PLATON.[10] The solvent mask file (.fab) computed by PLATON was included in the SHELXL refinement via the ABIN instruction leaving the measured intensities untouched.

Due to the fact that no additional chiral information was added in during the photoreaction the structure was refined as a 2-component inversion twin.

Geometrically similarity (SADI) for the C-Cl bonds of both dichloromethane solvent molecules were employed as well as RESTRAINTS for anisotropic displacement parameters of isopropyl carbon atoms and the successfully modelled N_2 molecule produced by the *in crystallo* photoreaction.

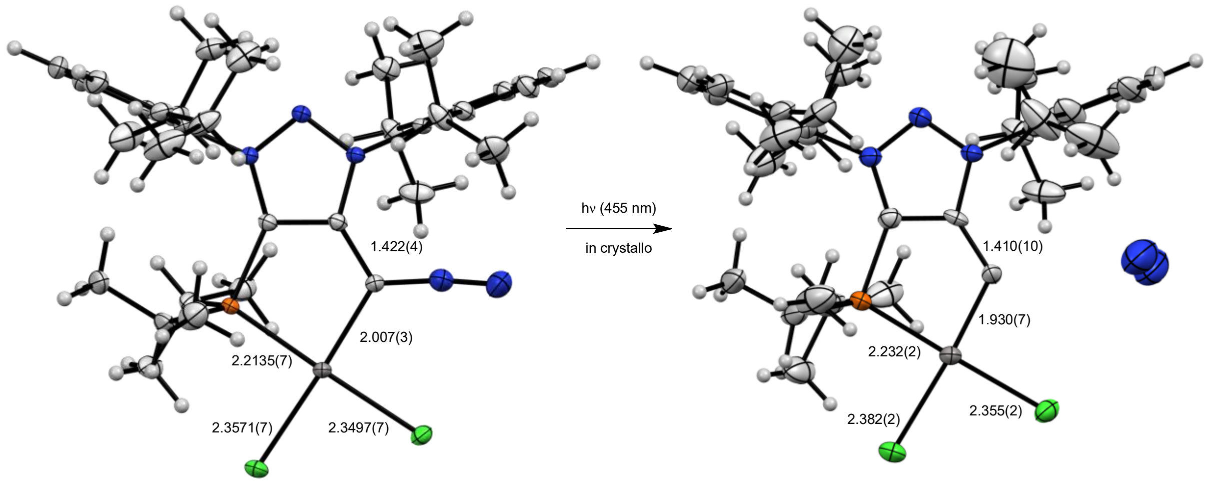

Fig. S116: X-ray solid-state structure of compound **6** with irradiation at 455 nm to convert photochemically to compound **8** in crystallo.

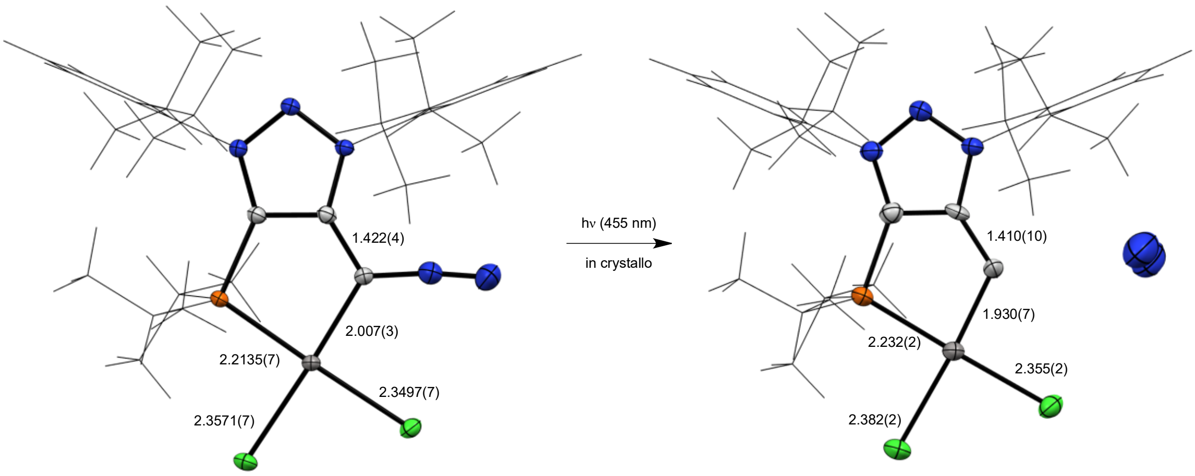

Fig. S117: X-ray solid-state structure of compound **6** with irradiation at 455 nm to convert photochemically to compound **8** in crystallo. ^{i}Pr- and Dipp-groups (2,6-diisopropylphenyl) are shown as wireframe for clarity.

Geometrical Parameters of educt and product of *in crystallo* radiation experiment

Full geometrical details of educt 6

Table S1. Bond lengths and angles for educt 6, ma3_189f.

Atom–Atom	Length [Å]
C1_1–N1_1	1.324(4)
C1_1–C2_1	1.422(4)
C1_1–Pt1_1	2.007(3)
Cl1_1–Pt1_1	2.3497(7)
Pt1_1–P1_1	2.2135(7)
Pt1_1–Cl2_1	2.3571(7)
P1_1–C3_1	1.826(3)
P1_1–C4_1	1.829(3)
P1_1–C7_1	1.840(3)
N1_1–N2_1	1.154(4)
C2_1–N3_1	1.371(4)
C2_1–C3_1	1.389(4)
C3_1–N5_1	1.370(4)
N3_1–N4_1	1.351(3)
N3_1–C10_1	1.455(4)
N4_1–N5_1	1.321(3)
C4_1–C6_1	1.524(6)
C4_1–C5_1	1.535(5)
C4_1–H4_1	1.0000
N5_1–C22_1	1.459(4)
C6_1–H6A_1	0.9800
C6_1–H6B_1	0.9800
C6_1–H6C_1	0.9800
C7_1–C8_1	1.525(5)
C7_1–C9_1	1.536(4)
C7_1–H7_1	1.0000
C8_1–H8A_1	0.9800
C8_1–H8B_1	0.9800
C8_1–H8C_1	0.9800
C9_1–H9A_1	0.9800
C9_1–H9B_1	0.9800
C9_1–H9C_1	0.9800
C10_1–C15_1	1.396(4)
C10_1–C11_1	1.399(4)
C11_1–C12_1	1.391(4)
C11_1–C16_1	1.517(4)
C12_1–C13_1	1.387(5)
C12_1–H12_1	0.9500
C13_1–C14_1	1.384(5)
C13_1–H13_1	0.9500
C14_1–C15_1	1.398(4)
C14_1–H14_1	0.9500
C15_1–C19_1	1.517(4)
C16_1–C17_1	1.522(5)
C16_1–C18_1	1.534(5)
C16_1–H16_1	1.0000
C17_1–H17A_1	0.9800
C17_1–H17B_1	0.9800
C17_1–H17C_1	0.9800
C18_1–H18A_1	0.9800
C18_1–H18B_1	0.9800
C18_1–H18C_1	0.9800
C19_1–C20_1	1.534(5)

C19_1–C21_1	1.537(4)
C19_1–H19_1	1.0000
C20_1–H20A_1	0.9800
C20_1–H20B_1	0.9800
C20_1–H20C_1	0.9800
C21_1–H21A_1	0.9800
C21_1–H21B_1	0.9800
C21_1–H21C_1	0.9800
C22_1–C23_1	1.389(5)
C22_1–C27_1	1.402(4)
C23_1–C24_1	1.405(5)
C23_1–C28_1	1.525(5)
C24_1–C25_1	1.380(6)
C24_1–H24_1	0.9500
C25_1–C26_1	1.372(6)
C25_1–H25_1	0.9500
C26_1–C27_1	1.404(4)
C26_1–H26_1	0.9500
C27_1–C31_1	1.514(5)
C28_1–C30_1	1.533(5)
C28_1–C29_1	1.535(6)
C28_1–H28_1	1.0000
C29_1–H29A_1	0.9800
C29_1–H29B_1	0.9800
C29_1–H29C_1	0.9800
C30_1–H30A_1	0.9800
C30_1–H30B_1	0.9800
C30_1–H30C_1	0.9800
C31_1–C33_1	1.527(5)
C31_1–C32_1	1.531(5)
C31_1–H31_1	1.0000
C32_1–H32A_1	0.9800
C32_1–H32B_1	0.9800
C32_1–H32C_1	0.9800
C33_1–H33A_1	0.9800
C33_1–H33B_1	0.9800
C33_1–H33C_1	0.9800
C5_1–H5A_1	0.9800
C5_1–H5B_1	0.9800
C5_1–H5C_1	0.9800
C1_2–Cl2_2	1.729(7)
C1_2–Cl1_2	1.741(7)
C1_2–H1A_2	0.9900
C1_2–H1AB_2	0.9900

**Atom–Atom–Atom**	**Angle [°]**
N1_1–C1_1–C2_1	118.8(3)
N1_1–C1_1–Pt1_1	124.1(2)
C2_1–C1_1–Pt1_1	117.1(2)
C1_1–Pt1_1–P1_1	86.30(8)
C1_1–Pt1_1–Cl1_1	91.90(8)
P1_1–Pt1_1–Cl1_1	176.92(3)
C1_1–Pt1_1–Cl2_1	176.00(9)
P1_1–Pt1_1–Cl2_1	91.42(3)
Cl1_1–Pt1_1–Cl2_1	90.52(3)
C3_1–P1_1–C4_1	111.54(15)
C3_1–P1_1–C7_1	104.27(14)
C4_1–P1_1–C7_1	107.93(17)
C3_1–P1_1–Pt1_1	100.51(10)
C4_1–P1_1–Pt1_1	117.56(11)
C7_1–P1_1–Pt1_1	114.07(11)
N2_1–N1_1–C1_1	173.1(3)
N3_1–C2_1–C3_1	104.9(2)
N3_1–C2_1–C1_1	133.8(3)
C3_1–C2_1–C1_1	121.3(3)
N5_1–C3_1–C2_1	105.6(2)
N5_1–C3_1–P1_1	140.5(2)
C2_1–C3_1–P1_1	113.2(2)
N4_1–N3_1–C2_1	112.3(2)
N4_1–N3_1–C10_1	119.7(2)
C2_1–N3_1–C10_1	127.6(2)
N5_1–N4_1–N3_1	104.3(2)
C6_1–C4_1–C5_1	112.6(3)
C6_1–C4_1–P1_1	109.7(2)
C5_1–C4_1–P1_1	115.3(3)
C6_1–C4_1–H4_1	106.2
C5_1–C4_1–H4_1	106.2
P1_1–C4_1–H4_1	106.2
N4_1–N5_1–C3_1	112.9(2)
N4_1–N5_1–C22_1	116.9(2)
C3_1–N5_1–C22_1	130.2(2)
C4_1–C6_1–H6A_1	109.5
C4_1–C6_1–H6B_1	109.5
H6A_1–C6_1–H6B_1	109.5
C4_1–C6_1–H6C_1	109.5
H6A_1–C6_1–H6C_1	109.5
H6B_1–C6_1–H6C_1	109.5
C8_1–C7_1–C9_1	111.0(3)
C8_1–C7_1–P1_1	111.0(2)
C9_1–C7_1–P1_1	109.7(2)
C8_1–C7_1–H7_1	108.4
C9_1–C7_1–H7_1	108.4
P1_1–C7_1–H7_1	108.4
C7_1–C8_1–H8A_1	109.5
C7_1–C8_1–H8B_1	109.5
H8A_1–C8_1–H8B_1	109.5
C7_1–C8_1–H8C_1	109.5
H8A_1–C8_1–H8C_1	109.5
H8B_1–C8_1–H8C_1	109.5
C7_1–C9_1–H9A_1	109.5
C7_1–C9_1–H9B_1	109.5
H9A_1–C9_1–H9B_1	109.5
C7_1–C9_1–H9C_1	109.5
H9A_1–C9_1–H9C_1	109.5
H9B_1–C9_1–H9C_1	109.5
C15_1–C10_1–C11_1	124.5(3)
C15_1–C10_1–N3_1	117.0(3)
C11_1–C10_1–N3_1	118.5(3)

C12_1–C11_1–C10_1	116.3(3)
C12_1–C11_1–C16_1	120.8(3)
C10_1–C11_1–C16_1	122.8(3)
C13_1–C12_1–C11_1	121.3(3)
C13_1–C12_1–H12_1	119.4
C11_1–C12_1–H12_1	119.4
C14_1–C13_1–C12_1	120.5(3)
C14_1–C13_1–H13_1	119.8
C12_1–C13_1–H13_1	119.8
C13_1–C14_1–C15_1	121.0(3)
C13_1–C14_1–H14_1	119.5
C15_1–C14_1–H14_1	119.5
C10_1–C15_1–C14_1	116.3(3)
C10_1–C15_1–C19_1	123.7(3)
C14_1–C15_1–C19_1	120.0(3)
C11_1–C16_1–C17_1	112.7(3)
C11_1–C16_1–C18_1	109.1(3)
C17_1–C16_1–C18_1	109.3(3)
C11_1–C16_1–H16_1	108.5
C17_1–C16_1–H16_1	108.5
C18_1–C16_1–H16_1	108.5
C16_1–C17_1–H17A_1	109.5
C16_1–C17_1–H17B_1	109.5
H17A_1–C17_1–H17B_1	109.5
C16_1–C17_1–H17C_1	109.5
H17A_1–C17_1–H17C_1	109.5
H17B_1–C17_1–H17C_1	109.5
C16_1–C18_1–H18A_1	109.5
C16_1–C18_1–H18B_1	109.5
H18A_1–C18_1–H18B_1	109.5
C16_1–C18_1–H18C_1	109.5
H18A_1–C18_1–H18C_1	109.5
H18B_1–C18_1–H18C_1	109.5
C15_1–C19_1–C20_1	110.3(3)
C15_1–C19_1–C21_1	111.2(3)
C20_1–C19_1–C21_1	110.3(3)
C15_1–C19_1–H19_1	108.3
C20_1–C19_1–H19_1	108.3
C21_1–C19_1–H19_1	108.3
C19_1–C20_1–H20A_1	109.5
C19_1–C20_1–H20B_1	109.5
H20A_1–C20_1–H20B_1	109.5
C19_1–C20_1–H20C_1	109.5
H20A_1–C20_1–H20C_1	109.5
H20B_1–C20_1–H20C_1	109.5
C19_1–C21_1–H21A_1	109.5
C19_1–C21_1–H21B_1	109.5
H21A_1–C21_1–H21B_1	109.5
C19_1–C21_1–H21C_1	109.5
H21A_1–C21_1–H21C_1	109.5
H21B_1–C21_1–H21C_1	109.5
C23_1–C22_1–C27_1	124.8(3)
C23_1–C22_1–N5_1	117.9(3)
C27_1–C22_1–N5_1	117.2(3)
C22_1–C23_1–C24_1	116.4(3)
C22_1–C23_1–C28_1	122.8(3)
C24_1–C23_1–C28_1	120.6(3)
C25_1–C24_1–C23_1	120.8(3)
C25_1–C24_1–H24_1	119.6
C23_1–C24_1–H24_1	119.6
C26_1–C25_1–C24_1	120.8(3)
C26_1–C25_1–H25_1	119.6
C24_1–C25_1–H25_1	119.6
C25_1–C26_1–C27_1	121.7(3)
C25_1–C26_1–H26_1	119.2
C27_1–C26_1–H26_1	119.2
C22_1–C27_1–C26_1	115.5(3)
C22_1–C27_1–C31_1	122.6(3)
C26_1–C27_1–C31_1	121.7(3)
C23_1–C28_1–C30_1	113.3(3)
C23_1–C28_1–C29_1	108.8(3)
C30_1–C28_1–C29_1	110.6(3)
C23_1–C28_1–H28_1	108.0
C30_1–C28_1–H28_1	108.0
C29_1–C28_1–H28_1	108.0
C28_1–C29_1–H29A_1	109.5
C28_1–C29_1–H29B_1	109.5
H29A_1–C29_1–H29B_1	109.5
C28_1–C29_1–H29C_1	109.5
H29A_1–C29_1–H29C_1	109.5
H29B_1–C29_1–H29C_1	109.5
C28_1–C30_1–H30A_1	109.5
C28_1–C30_1–H30B_1	109.5
H30A_1–C30_1–H30B_1	109.5
C28_1–C30_1–H30C_1	109.5
H30A_1–C30_1–H30C_1	109.5
H30B_1–C30_1–H30C_1	109.5
C27_1–C31_1–C33_1	114.2(3)
C27_1–C31_1–C32_1	108.7(3)
C33_1–C31_1–C32_1	110.3(3)

C27_1–C31_1–H31_1	107.8
C33_1–C31_1–H31_1	107.8
C32_1–C31_1–H31_1	107.8
C31_1–C32_1–H32A_1	109.5
C31_1–C32_1–H32B_1	109.5
H32A_1–C32_1–H32B_1	109.5
C31_1–C32_1–H32C_1	109.5
H32A_1–C32_1–H32C_1	109.5
H32B_1–C32_1–H32C_1	109.5
C31_1–C33_1–H33A_1	109.5
C31_1–C33_1–H33B_1	109.5
H33A_1–C33_1–H33B_1	109.5
C31_1–C33_1–H33C_1	109.5
H33A_1–C33_1–H33C_1	109.5
H33B_1–C33_1–H33C_1	109.5
C4_1–C5_1–H5A_1	109.5
C4_1–C5_1–H5B_1	109.5
H5A_1–C5_1–H5B_1	109.5
C4_1–C5_1–H5C_1	109.5
H5A_1–C5_1–H5C_1	109.5
H5B_1–C5_1–H5C_1	109.5
Cl2_2–C1_2–Cl1_2	114.9(4)
Cl2_2–C1_2–H1A_2	108.5
Cl1_2–C1_2–H1A_2	108.5
Cl2_2–C1_2–H1AB_2	108.5
Cl1_2–C1_2–H1AB_2	108.5
H1A_2–C1_2–H1AB_2	107.5

Table S2. Torsion angles for educt 6, ma3_189f

Atom–Atom–Atom–Atom	Torsion Angle [°]
N1_1–C1_1–C2_1–N3_1	-0.5(5)
Pt1_1–C1_1–C2_1–N3_1	178.4(3)
N1_1–C1_1–C2_1–C3_1	177.9(3)
Pt1_1–C1_1–C2_1–C3_1	-3.2(4)
N3_1–C2_1–C3_1–N5_1	-0.5(3)
C1_1–C2_1–C3_1–N5_1	-179.3(3)
N3_1–C2_1–C3_1–P1_1	171.97(19)
C1_1–C2_1–C3_1–P1_1	-6.8(4)
C4_1–P1_1–C3_1–N5_1	-54.3(4)
C7_1–P1_1–C3_1–N5_1	62.0(4)
Pt1_1–P1_1–C3_1–N5_1	-179.7(3)
C4_1–P1_1–C3_1–C2_1	137.2(2)
C7_1–P1_1–C3_1–C2_1	-106.6(2)
Pt1_1–P1_1–C3_1–C2_1	11.8(2)
C3_1–C2_1–N3_1–N4_1	0.5(3)
C1_1–C2_1–N3_1–N4_1	179.0(3)
C3_1–C2_1–N3_1–C10_1	-171.7(3)
C1_1–C2_1–N3_1–C10_1	6.9(5)
C2_1–N3_1–N4_1–N5_1	-0.2(3)
C10_1–N3_1–N4_1–N5_1	172.6(2)
C3_1–P1_1–C4_1–C6_1	-57.2(3)
C7_1–P1_1–C4_1–C6_1	-171.2(2)
Pt1_1–P1_1–C4_1–C6_1	58.1(3)
C3_1–P1_1–C4_1–C5_1	71.2(3)
C7_1–P1_1–C4_1–C5_1	-42.8(3)
Pt1_1–P1_1–C4_1–C5_1	-173.5(2)
N3_1–N4_1–N5_1–C3_1	-0.1(3)
N3_1–N4_1–N5_1–C22_1	179.4(2)
C2_1–C3_1–N5_1–N4_1	0.4(3)
P1_1–C3_1–N5_1–N4_1	-168.7(3)
C2_1–C3_1–N5_1–C22_1	-179.1(3)
P1_1–C3_1–N5_1–C22_1	11.9(5)
C3_1–P1_1–C7_1–C8_1	62.5(2)
C4_1–P1_1–C7_1–C8_1	-178.8(2)
Pt1_1–P1_1–C7_1–C8_1	-46.1(2)
C3_1–P1_1–C7_1–C9_1	-174.5(2)
C4_1–P1_1–C7_1–C9_1	-55.7(3)
Pt1_1–P1_1–C7_1–C9_1	76.9(3)
N4_1–N3_1–C10_1–C15_1	-88.7(3)
C2_1–N3_1–C10_1–C15_1	83.0(4)
N4_1–N3_1–C10_1–C11_1	91.9(3)
C2_1–N3_1–C10_1–C11_1	-96.4(4)
C15_1–C10_1–C11_1–C12_1	-2.3(5)
N3_1–C10_1–C11_1–C12_1	177.1(3)
C15_1–C10_1–C11_1–C16_1	174.0(3)
N3_1–C10_1–C11_1–C16_1	-6.7(4)
C10_1–C11_1–C12_1–C13_1	0.0(5)
C16_1–C11_1–C12_1–C13_1	-176.3(3)
C11_1–C12_1–C13_1–C14_1	2.0(5)
C12_1–C13_1–C14_1–C15_1	-1.9(5)
C11_1–C10_1–C15_1–C14_1	2.4(4)
N3_1–C10_1–C15_1–C14_1	-177.0(2)
C11_1–C10_1–C15_1–C19_1	-177.8(3)
N3_1–C10_1–C15_1–C19_1	2.8(4)
C13_1–C14_1–C15_1–C10_1	-0.2(4)

C13_1–C14_1–C15_1–C19_1	180.0(3)	C28_1–C23_1–C24_1–C25_1	174.8(3)
C12_1–C11_1–C16_1–C17_1	-47.5(4)	C23_1–C24_1–C25_1–C26_1	-1.2(5)
C10_1–C11_1–C16_1–C17_1	136.4(3)	C24_1–C25_1–C26_1–C27_1	0.1(5)
C12_1–C11_1–C16_1–C18_1	74.1(4)	C23_1–C22_1–C27_1–C26_1	-2.6(5)
C10_1–C11_1–C16_1–C18_1	-102.0(4)	N5_1–C22_1–C27_1–C26_1	179.6(3)
C10_1–C15_1–C19_1–C20_1	-109.5(3)	C23_1–C22_1–C27_1–C31_1	171.3(3)
C14_1–C15_1–C19_1–C20_1	70.2(4)	N5_1–C22_1–C27_1–C31_1	-6.4(4)
C10_1–C15_1–C19_1–C21_1	127.7(3)	C25_1–C26_1–C27_1–C22_1	1.7(5)
C14_1–C15_1–C19_1–C21_1	-52.5(4)	C25_1–C26_1–C27_1–C31_1	-172.4(3)
N4_1–N5_1–C22_1–C23_1	-87.1(3)	C22_1–C23_1–C28_1–C30_1	-146.5(3)
C3_1–N5_1–C22_1–C23_1	92.3(4)	C24_1–C23_1–C28_1–C30_1	39.4(5)
N4_1–N5_1–C22_1–C27_1	90.8(3)	C22_1–C23_1–C28_1–C29_1	90.0(4)
C3_1–N5_1–C22_1–C27_1	-89.8(4)		
C27_1–C22_1–C23_1–C24_1	1.7(5)	C24_1–C23_1–C28_1–C29_1	-84.2(4)
N5_1–C22_1–C23_1–C24_1	179.4(3)	C22_1–C27_1–C31_1–C33_1	146.6(3)
C27_1–C22_1–C23_1–C28_1	-172.7(3)	C26_1–C27_1–C31_1–C33_1	-39.8(4)
N5_1–C22_1–C23_1–C28_1	5.0(4)	C22_1–C27_1–C31_1–C32_1	-89.8(3)
C22_1–C23_1–C24_1–C25_1	0.3(5)	C26_1–C27_1–C31_1–C32_1	83.8(4)

Geometrical details of *in crystallo* radiation product 8

Table S3. Bond lengths and angles for *in crystallo* radiation product 8, ma3_189f_hv_cc_sq

**Atom–Atom**	**Length [Å]**
Pt1_1–C1_1	1.931(8)
Pt1_1–P1_1	2.232(2)
Pt1_1–Cl1_1	2.355(2)
Pt1_1–Cl2_1	2.382(2)
P1_1–C4_1	1.829(8)
P1_1–C3_1	1.831(8)
P1_1–C7_1	1.844(9)
C1_1–C2_1	1.411(13)
C2_1–N3_1	1.365(12)
C2_1–C3_1	1.400(12)
N3_1–N4_1	1.370(10)
N3_1–C10_1	1.467(10)
C3_1–N5_1	1.355(9)
N4_1–N5_1	1.334(10)
C4_1–C5_1	1.514(13)
C4_1–C6_1	1.553(11)
C4_1–H4_1	1.0000
N5_1–C22_1	1.453(11)
C5_1–H5A_1	0.9800
C5_1–H5B_1	0.9800
C5_1–H5C_1	0.9800
C6_1–H6A_1	0.9800
C6_1–H6B_1	0.9800

C6_1–H6C_1	0.9800	C29_1–H29B_1	0.9800
C7_1–C8_1	1.527(11)	C29_1–H29C_1	0.9800
C7_1–C9_1	1.528(12)	C30_1–H30A_1	0.9800
C7_1–H7_1	1.0000	C30_1–H30B_1	0.9800
C8_1–H8A_1	0.9800	C30_1–H30C_1	0.9800
C8_1–H8B_1	0.9800	C31_1–C33_1	1.522(13)
C8_1–H8C_1	0.9800	C31_1–C32_1	1.534(14)
C9_1–H9A_1	0.9800	C31_1–H31_1	1.0000
C9_1–H9B_1	0.9800	C32_1–H32A_1	0.9800
C9_1–H9C_1	0.9800	C32_1–H32B_1	0.9800
C10_1–C11_1	1.385(11)	C32_1–H32C_1	0.9800
C10_1–C15_1	1.393(11)	C33_1–H33A_1	0.9800
C11_1–C12_1	1.390(12)	C33_1–H33B_1	0.9800
C11_1–C16_1	1.511(12)	C33_1–H33C_1	0.9800
C12_1–C13_1	1.393(13)	Pt1_2–C1_2	1.918(8)
C12_1–H12_1	0.9500	Pt1_2–P1_2	2.234(2)
C13_1–C14_1	1.355(12)	Pt1_2–Cl1_2	2.352(2)
C13_1–H13_1	0.9500	Pt1_2–Cl2_2	2.378(2)
C14_1–C15_1	1.395(12)	P1_2–C7_2	1.826(10)
C14_1–H14_1	0.9500	P1_2–C3_2	1.842(8)
C15_1–C19_1	1.517(11)	P1_2–C4_2	1.847(9)
C16_1–C18_1	1.518(12)	C1_2–C2_2	1.397(11)
C16_1–C17_1	1.529(12)	C2_2–C3_2	1.390(12)
C16_1–H16_1	1.0000	C2_2–N3_2	1.400(11)
C17_1–H17A_1	0.9800	N3_2–N4_2	1.319(10)
C17_1–H17B_1	0.9800	N3_2–C10_2	1.441(10)
C17_1–H17C_1	0.9800	C3_2–N5_2	1.358(9)
C18_1–H18A_1	0.9800	N4_2–N5_2	1.334(9)
C18_1–H18B_1	0.9800	C4_2–C5_2	1.473(18)
C18_1–H18C_1	0.9800	C4_2–C6_2	1.527(14)
C19_1–C20_1	1.486(12)	C4_2–H4_2	1.0000
C19_1–C21_1	1.548(12)	N5_2–C22_2	1.461(10)
C19_1–H19_1	1.0000	C5_2–H5A_2	0.9800
C20_1–H20A_1	0.9800	C5_2–H5B_2	0.9800
C20_1–H20B_1	0.9800	C5_2–H5C_2	0.9800
C20_1–H20C_1	0.9800	C6_2–H6A_2	0.9800
C21_1–H21A_1	0.9800	C6_2–H6B_2	0.9800
C21_1–H21B_1	0.9800	C6_2–H6C_2	0.9800
C21_1–H21C_1	0.9800	C7_2–C8_2	1.524(15)
C22_1–C27_1	1.379(12)	C7_2–C9_2	1.545(13)
C22_1–C23_1	1.439(12)	C7_2–H7_2	1.0000
C23_1–C24_1	1.410(11)	C8_2–H8A_2	0.9800
C23_1–C28_1	1.482(11)	C8_2–H8B_2	0.9800
C24_1–C25_1	1.368(14)	C8_2–H8C_2	0.9800
C24_1–H24_1	0.9500	C9_2–H9A_2	0.9800
C25_1–C26_1	1.384(14)	C9_2–H9B_2	0.9800
C25_1–H25_1	0.9500	C9_2–H9C_2	0.9800
C26_1–C27_1	1.429(13)	C10_2–C11_2	1.392(13)
C26_1–H26_1	0.9500	C10_2–C15_2	1.396(11)
C27_1–C31_1	1.494(14)	C11_2–C12_2	1.400(14)
C28_1–C30_1	1.542(11)	C11_2–C16_2	1.530(14)
C28_1–C29_1	1.549(11)	C12_2–C13_2	1.342(14)
C28_1–H28_1	1.0000	C12_2–H12_2	0.9500
C29_1–H29A_1	0.9800	C13_2–C14_2	1.363(15)

C13_2–H13_2	0.9500
C14_2–C15_2	1.411(12)
C14_2–H14_2	0.9500
C15_2–C19_2	1.544(14)
C16_2–C17_2	1.419(18)
C16_2–C18_2	1.591(15)
C16_2–H16_2	1.0000
C17_2–H17A_2	0.9800
C17_2–H17B_2	0.9800
C17_2–H17C_2	0.9800
C18_2–H18A_2	0.9800
C18_2–H18B_2	0.9800
C18_2–H18C_2	0.9800
C19_2–C21_2	1.513(12)
C19_2–C20_2	1.521(12)
C19_2–H19_2	1.0000
C20_2–H20A_2	0.9800
C20_2–H20B_2	0.9800
C20_2–H20C_2	0.9800
C21_2–H21A_2	0.9800
C21_2–H21B_2	0.9800
C21_2–H21C_2	0.9800
C22_2–C27_2	1.339(12)
C22_2–C23_2	1.421(12)
C23_2–C24_2	1.390(13)
C23_2–C28_2	1.557(14)
C24_2–C25_2	1.344(15)
C24_2–H24_2	0.9500
C25_2–C26_2	1.364(14)
C25_2–H25_2	0.9500
C26_2–C27_2	1.409(12)
C26_2–H26_2	0.9500
C27_2–C31_2	1.503(13)
C28_2–C30_2	1.538(12)
C28_2–C29_2	1.549(15)
C28_2–H28_2	1.0000
C29_2–H29A_2	0.9800
C29_2–H29B_2	0.9800
C29_2–H29C_2	0.9800
C30_2–H30A_2	0.9800
C30_2–H30B_2	0.9800
C30_2–H30C_2	0.9800
C31_2–C32_2	1.528(12)
C31_2–C33_2	1.541(12)
C31_2–H31_2	1.0000
C32_2–H32A_2	0.9800
C32_2–H32B_2	0.9800
C32_2–H32C_2	0.9800
C33_2–H33A_2	0.9800
C33_2–H33B_2	0.9800
C33_2–H33C_2	0.9800
Cl1_3–C1_3	1.696(14)
C1_3–Cl2_3	1.698(14)
C1_3–H1A_3	0.9900
C1_3–H1B_3	0.9900
Cl1_4–C1_4	1.785(14)
C1_4–Cl2_4	1.702(14)
C1_4–H1A_4	0.9900
C1_4–H1B_4	0.9900
N1_6–N2_6	1.005(16)

**Atom–Atom–Atom**	**Angle [°]**
C1_1–Pt1_1–P1_1	85.2(3)
C1_1–Pt1_1–Cl1_1	93.8(3)
P1_1–Pt1_1–Cl1_1	177.50(8)
C1_1–Pt1_1–Cl2_1	175.7(2)
P1_1–Pt1_1–Cl2_1	91.10(8)
Cl1_1–Pt1_1–Cl2_1	90.00(8)
C4_1–P1_1–C3_1	112.3(4)
C4_1–P1_1–C7_1	106.3(4)
C3_1–P1_1–C7_1	104.4(4)
C4_1–P1_1–Pt1_1	117.7(3)
C3_1–P1_1–Pt1_1	100.6(2)
C7_1–P1_1–Pt1_1	114.7(3)
C2_1–C1_1–Pt1_1	120.3(6)
N3_1–C2_1–C3_1	105.1(8)
N3_1–C2_1–C1_1	134.2(8)
C3_1–C2_1–C1_1	120.7(8)
C2_1–N3_1–N4_1	112.8(7)
C2_1–N3_1–C10_1	128.5(7)
N4_1–N3_1–C10_1	118.2(6)
N5_1–C3_1–C2_1	105.2(7)
N5_1–C3_1–P1_1	142.8(6)
C2_1–C3_1–P1_1	111.0(6)
N5_1–N4_1–N3_1	102.5(6)
C5_1–C4_1–C6_1	112.9(7)
C5_1–C4_1–P1_1	110.8(6)
C6_1–C4_1–P1_1	115.0(6)
C5_1–C4_1–H4_1	105.7
C6_1–C4_1–H4_1	105.7
P1_1–C4_1–H4_1	105.7
N4_1–N5_1–C3_1	114.4(6)
N4_1–N5_1–C22_1	116.2(7)
C3_1–N5_1–C22_1	129.3(7)
C4_1–C5_1–H5A_1	109.5
C4_1–C5_1–H5B_1	109.5
H5A_1–C5_1–H5B_1	109.5
C4_1–C5_1–H5C_1	109.5
H5A_1–C5_1–H5C_1	109.5
H5B_1–C5_1–H5C_1	109.5
C4_1–C6_1–H6A_1	109.5
C4_1–C6_1–H6B_1	109.5
H6A_1–C6_1–H6B_1	109.5
C4_1–C6_1–H6C_1	109.5
H6A_1–C6_1–H6C_1	109.5
H6B_1–C6_1–H6C_1	109.5
C8_1–C7_1–C9_1	111.3(8)
C8_1–C7_1–P1_1	111.1(7)

C9_1–C7_1–P1_1	110.6(6)	H18A_1–C18_1–H18C_1	109.5
C8_1–C7_1–H7_1	107.9	H18B_1–C18_1–H18C_1	109.5
C9_1–C7_1–H7_1	107.9	C20_1–C19_1–C15_1	112.6(8)
P1_1–C7_1–H7_1	107.9	C20_1–C19_1–C21_1	109.7(8)
C7_1–C8_1–H8A_1	109.5	C15_1–C19_1–C21_1	110.0(7)
C7_1–C8_1–H8B_1	109.5	C20_1–C19_1–H19_1	108.1
H8A_1–C8_1–H8B_1	109.5	C15_1–C19_1–H19_1	108.1
C7_1–C8_1–H8C_1	109.5	C21_1–C19_1–H19_1	108.1
H8A_1–C8_1–H8C_1	109.5	C19_1–C20_1–H20A_1	109.5
H8B_1–C8_1–H8C_1	109.5	C19_1–C20_1–H20B_1	109.5
C7_1–C9_1–H9A_1	109.5	H20A_1–C20_1–H20B_1	109.5
C7_1–C9_1–H9B_1	109.5	C19_1–C20_1–H20C_1	109.5
H9A_1–C9_1–H9B_1	109.5	H20A_1–C20_1–H20C_1	109.5
C7_1–C9_1–H9C_1	109.5	H20B_1–C20_1–H20C_1	109.5
H9A_1–C9_1–H9C_1	109.5	C19_1–C21_1–H21A_1	109.5
H9B_1–C9_1–H9C_1	109.5	C19_1–C21_1–H21B_1	109.5
C11_1–C10_1–C15_1	124.7(7)	H21A_1–C21_1–H21B_1	109.5
C11_1–C10_1–N3_1	117.2(7)	C19_1–C21_1–H21C_1	109.5
C15_1–C10_1–N3_1	118.1(7)	H21A_1–C21_1–H21C_1	109.5
C10_1–C11_1–C12_1	116.9(8)	H21B_1–C21_1–H21C_1	109.5
C10_1–C11_1–C16_1	123.8(8)	C27_1–C22_1–C23_1	125.0(9)
C12_1–C11_1–C16_1	119.3(7)	C27_1–C22_1–N5_1	118.4(9)
C11_1–C12_1–C13_1	120.2(8)	C23_1–C22_1–N5_1	116.5(7)
C11_1–C12_1–H12_1	119.9	C24_1–C23_1–C22_1	115.1(7)
C13_1–C12_1–H12_1	119.9	C24_1–C23_1–C28_1	122.0(7)
C14_1–C13_1–C12_1	120.3(9)	C22_1–C23_1–C28_1	122.6(7)
C14_1–C13_1–H13_1	119.8	C25_1–C24_1–C23_1	122.1(8)
C12_1–C13_1–H13_1	119.8	C25_1–C24_1–H24_1	119.0
C13_1–C14_1–C15_1	122.5(8)	C23_1–C24_1–H24_1	119.0
C13_1–C14_1–H14_1	118.7	C24_1–C25_1–C26_1	120.7(8)
C15_1–C14_1–H14_1	118.7	C24_1–C25_1–H25_1	119.7
C10_1–C15_1–C14_1	115.2(7)	C26_1–C25_1–H25_1	119.7
C10_1–C15_1–C19_1	123.7(8)	C25_1–C26_1–C27_1	121.6(8)
C14_1–C15_1–C19_1	121.0(8)	C25_1–C26_1–H26_1	119.2
C11_1–C16_1–C18_1	112.4(7)	C27_1–C26_1–H26_1	119.2
C11_1–C16_1–C17_1	110.7(7)	C22_1–C27_1–C26_1	115.5(9)
C18_1–C16_1–C17_1	109.9(7)	C22_1–C27_1–C31_1	123.8(9)
C11_1–C16_1–H16_1	107.9	C26_1–C27_1–C31_1	120.7(8)
C18_1–C16_1–H16_1	107.9	C23_1–C28_1–C30_1	109.2(7)
C17_1–C16_1–H16_1	107.9	C23_1–C28_1–C29_1	114.1(8)
C16_1–C17_1–H17A_1	109.5	C30_1–C28_1–C29_1	110.2(7)
C16_1–C17_1–H17B_1	109.5	C23_1–C28_1–H28_1	107.7
H17A_1–C17_1–H17B_1	109.5	C30_1–C28_1–H28_1	107.7
C16_1–C17_1–H17C_1	109.5	C29_1–C28_1–H28_1	107.7
H17A_1–C17_1–H17C_1	109.5	C28_1–C29_1–H29A_1	109.5
H17B_1–C17_1–H17C_1	109.5	C28_1–C29_1–H29B_1	109.5
C16_1–C18_1–H18A_1	109.5		
C16_1–C18_1–H18B_1	109.5		
H18A_1–C18_1–H18B_1	109.5		
C16_1–C18_1–H18C_1	109.5		

H29A_1–C29_1–H29B_1	109.5
C28_1–C29_1–H29C_1	109.5
H29A_1–C29_1–H29C_1	109.5
H29B_1–C29_1–H29C_1	109.5
C28_1–C30_1–H30A_1	109.5
C28_1–C30_1–H30B_1	109.5
H30A_1–C30_1–H30B_1	109.5
C28_1–C30_1–H30C_1	109.5
H30A_1–C30_1–H30C_1	109.5
H30B_1–C30_1–H30C_1	109.5
C27_1–C31_1–C33_1	114.0(9)
C27_1–C31_1–C32_1	110.0(8)
C33_1–C31_1–C32_1	111.4(9)
C27_1–C31_1–H31_1	107.0
C33_1–C31_1–H31_1	107.0
C32_1–C31_1–H31_1	107.0
C31_1–C32_1–H32A_1	109.5
C31_1–C32_1–H32B_1	109.5
H32A_1–C32_1–H32B_1	109.5
C31_1–C32_1–H32C_1	109.5
H32A_1–C32_1–H32C_1	109.5
H32B_1–C32_1–H32C_1	109.5
C31_1–C33_1–H33A_1	109.5
C31_1–C33_1–H33B_1	109.5
H33A_1–C33_1–H33B_1	109.5
C31_1–C33_1–H33C_1	109.5
H33A_1–C33_1–H33C_1	109.5
H33B_1–C33_1–H33C_1	109.5
C1_2–Pt1_2–P1_2	85.2(2)
C1_2–Pt1_2–Cl1_2	93.3(2)
P1_2–Pt1_2–Cl1_2	177.86(7)
C1_2–Pt1_2–Cl2_2	174.6(2)
P1_2–Pt1_2–Cl2_2	91.11(7)
Cl1_2–Pt1_2–Cl2_2	90.47(8)
C7_2–P1_2–C3_2	104.2(4)
C7_2–P1_2–C4_2	109.8(5)
C3_2–P1_2–C4_2	109.5(4)
C7_2–P1_2–Pt1_2	114.4(3)
C3_2–P1_2–Pt1_2	100.7(3)
C4_2–P1_2–Pt1_2	116.9(4)
C2_2–C1_2–Pt1_2	120.8(6)
C3_2–C2_2–C1_2	122.0(8)
C3_2–C2_2–N3_2	104.2(7)
C1_2–C2_2–N3_2	133.9(8)
N4_2–N3_2–C2_2	111.0(7)
N4_2–N3_2–C10_2	121.9(7)
C2_2–N3_2–C10_2	126.6(7)
N5_2–C3_2–C2_2	107.1(7)
N5_2–C3_2–P1_2	142.3(6)
C2_2–C3_2–P1_2	110.1(6)
N3_2–N4_2–N5_2	107.0(7)
C5_2–C4_2–C6_2	113.9(10)
C5_2–C4_2–P1_2	109.8(8)
C6_2–C4_2–P1_2	113.3(8)
C5_2–C4_2–H4_2	106.4
C6_2–C4_2–H4_2	106.4
P1_2–C4_2–H4_2	106.4
N4_2–N5_2–C3_2	110.8(6)
N4_2–N5_2–C22_2	118.0(6)
C3_2–N5_2–C22_2	131.2(7)
C4_2–C5_2–H5A_2	109.5
C4_2–C5_2–H5B_2	109.5
H5A_2–C5_2–H5B_2	109.5
C4_2–C5_2–H5C_2	109.5
H5A_2–C5_2–H5C_2	109.5
H5B_2–C5_2–H5C_2	109.5
C4_2–C6_2–H6A_2	109.5
C4_2–C6_2–H6B_2	109.5
H6A_2–C6_2–H6B_2	109.5
C4_2–C6_2–H6C_2	109.5
H6A_2–C6_2–H6C_2	109.5
H6B_2–C6_2–H6C_2	109.5
C8_2–C7_2–C9_2	111.4(8)
C8_2–C7_2–P1_2	110.6(6)
C9_2–C7_2–P1_2	108.8(7)
C8_2–C7_2–H7_2	108.7
C9_2–C7_2–H7_2	108.7
P1_2–C7_2–H7_2	108.7
C7_2–C8_2–H8A_2	109.5
C7_2–C8_2–H8B_2	109.5
H8A_2–C8_2–H8B_2	109.5
C7_2–C8_2–H8C_2	109.5
H8A_2–C8_2–H8C_2	109.5
H8B_2–C8_2–H8C_2	109.5
C7_2–C9_2–H9A_2	109.5
C7_2–C9_2–H9B_2	109.5
H9A_2–C9_2–H9B_2	109.5
C7_2–C9_2–H9C_2	109.5
H9A_2–C9_2–H9C_2	109.5
H9B_2–C9_2–H9C_2	109.5
C11_2–C10_2–C15_2	123.4(8)
C11_2–C10_2–N3_2	118.9(7)
C15_2–C10_2–N3_2	117.7(8)
C10_2–C11_2–C12_2	116.4(9)
C10_2–C11_2–C16_2	121.0(9)
C12_2–C11_2–C16_2	122.5(10)

C13_2–C12_2–C11_2	121.7(10)
C13_2–C12_2–H12_2	119.2
C11_2–C12_2–H12_2	119.2
C12_2–C13_2–C14_2	121.4(9)
C12_2–C13_2–H13_2	119.3
C14_2–C13_2–H13_2	119.3
C13_2–C14_2–C15_2	120.7(9)
C13_2–C14_2–H14_2	119.6
C15_2–C14_2–H14_2	119.6
C10_2–C15_2–C14_2	116.3(9)
C10_2–C15_2–C19_2	122.5(8)
C14_2–C15_2–C19_2	121.2(8)
C17_2–C16_2–C11_2	111.6(10)
C17_2–C16_2–C18_2	111.6(11)
C11_2–C16_2–C18_2	111.4(11)
C17_2–C16_2–H16_2	107.3
C11_2–C16_2–H16_2	107.3
C18_2–C16_2–H16_2	107.3
C16_2–C17_2–H17A_2	109.5
C16_2–C17_2–H17B_2	109.5
H17A_2–C17_2–H17B_2	109.5
C16_2–C17_2–H17C_2	109.5
H17A_2–C17_2–H17C_2	109.5
H17B_2–C17_2–H17C_2	109.5
C16_2–C18_2–H18A_2	109.5
C16_2–C18_2–H18B_2	109.5
H18A_2–C18_2–H18B_2	109.5
C16_2–C18_2–H18C_2	109.5
H18A_2–C18_2–H18C_2	109.5
H18B_2–C18_2–H18C_2	109.5
C21_2–C19_2–C20_2	110.6(7)
C21_2–C19_2–C15_2	110.1(7)
C20_2–C19_2–C15_2	109.0(8)
C21_2–C19_2–H19_2	109.0
C20_2–C19_2–H19_2	109.0
C15_2–C19_2–H19_2	109.0
C19_2–C20_2–H20A_2	109.5
C19_2–C20_2–H20B_2	109.5
H20A_2–C20_2–H20B_2	109.5
C19_2–C20_2–H20C_2	109.5
H20A_2–C20_2–H20C_2	109.5
H20B_2–C20_2–H20C_2	109.5
C19_2–C21_2–H21A_2	109.5
C19_2–C21_2–H21B_2	109.5
H21A_2–C21_2–H21B_2	109.5
C19_2–C21_2–H21C_2	109.5
H21A_2–C21_2–H21C_2	109.5
H21B_2–C21_2–H21C_2	109.5
C27_2–C22_2–C23_2	125.3(8)
C27_2–C22_2–N5_2	118.7(7)
C23_2–C22_2–N5_2	116.0(8)
C24_2–C23_2–C22_2	116.2(10)
C24_2–C23_2–C28_2	120.0(8)
C22_2–C23_2–C28_2	123.4(8)
C25_2–C24_2–C23_2	119.4(10)
C25_2–C24_2–H24_2	120.3
C23_2–C24_2–H24_2	120.3
C24_2–C25_2–C26_2	123.0(9)
C24_2–C25_2–H25_2	118.5
C26_2–C25_2–H25_2	118.5
C25_2–C26_2–C27_2	120.6(10)
C25_2–C26_2–H26_2	119.7
C27_2–C26_2–H26_2	119.7
C22_2–C27_2–C26_2	115.5(8)
C22_2–C27_2–C31_2	122.3(7)
C26_2–C27_2–C31_2	122.0(8)
C30_2–C28_2–C29_2	110.5(8)
C30_2–C28_2–C23_2	112.9(9)
C29_2–C28_2–C23_2	108.1(7)
C30_2–C28_2–H28_2	108.4
C29_2–C28_2–H28_2	108.4
C23_2–C28_2–H28_2	108.4
C28_2–C29_2–H29A_2	109.5
C28_2–C29_2–H29B_2	109.5
H29A_2–C29_2–H29B_2	109.5
C28_2–C29_2–H29C_2	109.5
H29A_2–C29_2–H29C_2	109.5
H29B_2–C29_2–H29C_2	109.5
C28_2–C30_2–H30A_2	109.5
C28_2–C30_2–H30B_2	109.5
H30A_2–C30_2–H30B_2	109.5
C28_2–C30_2–H30C_2	109.5
H30A_2–C30_2–H30C_2	109.5
H30B_2–C30_2–H30C_2	109.5
C27_2–C31_2–C32_2	110.8(7)
C27_2–C31_2–C33_2	112.1(8)
C32_2–C31_2–C33_2	109.9(8)
C27_2–C31_2–H31_2	107.9
C32_2–C31_2–H31_2	107.9

C33_2–C31_2–H31_2	107.9
C31_2–C32_2–H32A_2	109.5
C31_2–C32_2–H32B_2	109.5
H32A_2–C32_2–H32B_2	109.5
C31_2–C32_2–H32C_2	109.5
H32A_2–C32_2–H32C_2	109.5
H32B_2–C32_2–H32C_2	109.5
C31_2–C33_2–H33A_2	109.5
C31_2–C33_2–H33B_2	109.5
H33A_2–C33_2–H33B_2	109.5
C31_2–C33_2–H33C_2	109.5
H33A_2–C33_2–H33C_2	109.5
H33B_2–C33_2–H33C_2	109.5
Cl1_3–C1_3–Cl2_3	112.2(9)
Cl1_3–C1_3–H1A_3	109.2
Cl2_3–C1_3–H1A_3	109.2
Cl1_3–C1_3–H1B_3	109.2
Cl2_3–C1_3–H1B_3	109.2
H1A_3–C1_3–H1B_3	107.9
Cl2_4–C1_4–Cl1_4	115.0(9)
Cl2_4–C1_4–H1A_4	108.5
Cl1_4–C1_4–H1A_4	108.5
Cl2_4–C1_4–H1B_4	108.5
Cl1_4–C1_4–H1B_4	108.5
H1A_4–C1_4–H1B_4	107.5

Table S4. Torsion angles for *in crystallo* radiation product 8, ma3_189f_hv_cc_sq.

**Atom–Atom–Atom–Atom**	**Torsion Angle [°]**
Pt1_1–C1_1–C2_1–N3_1	178.0(7)
Pt1_1–C1_1–C2_1–C3_1	-3.9(10)
C3_1–C2_1–N3_1–N4_1	0.8(8)
C1_1–C2_1–N3_1–N4_1	179.1(8)
C3_1–C2_1–N3_1–C10_1	-170.6(7)
C1_1–C2_1–N3_1–C10_1	7.7(14)
N3_1–C2_1–C3_1–N5_1	-0.5(8)
C1_1–C2_1–C3_1–N5_1	-179.1(7)
N3_1–C2_1–C3_1–P1_1	170.5(5)
C1_1–C2_1–C3_1–P1_1	-8.1(9)
C4_1–P1_1–C3_1–N5_1	-54.8(10)
C7_1–P1_1–C3_1–N5_1	60.1(10)
Pt1_1–P1_1–C3_1–N5_1	179.2(9)
C4_1–P1_1–C3_1–C2_1	139.7(6)
C7_1–P1_1–C3_1–C2_1	-105.4(6)
Pt1_1–P1_1–C3_1–C2_1	13.7(6)
C2_1–N3_1–N4_1–N5_1	-0.8(8)
C10_1–N3_1–N4_1–N5_1	171.6(6)
C3_1–P1_1–C4_1–C5_1	-62.9(7)
C7_1–P1_1–C4_1–C5_1	-176.5(6)
Pt1_1–P1_1–C4_1–C5_1	53.2(7)
C3_1–P1_1–C4_1–C6_1	66.7(7)
C7_1–P1_1–C4_1–C6_1	-46.9(7)
Pt1_1–P1_1–C4_1–C6_1	-177.2(5)
N3_1–N4_1–N5_1–C3_1	0.5(8)
N3_1–N4_1–N5_1–C22_1	179.2(6)
C2_1–C3_1–N5_1–N4_1	0.0(8)
P1_1–C3_1–N5_1–N4_1	-166.0(7)
C2_1–C3_1–N5_1–C22_1	-178.5(8)
P1_1–C3_1–N5_1–C22_1	15.5(14)
C4_1–P1_1–C7_1–C8_1	-54.0(7)
C3_1–P1_1–C7_1–C8_1	-172.9(6)
Pt1_1–P1_1–C7_1–C8_1	78.0(7)
C4_1–P1_1–C7_1–C9_1	-178.2(6)
C3_1–P1_1–C7_1–C9_1	62.9(6)
Pt1_1–P1_1–C7_1–C9_1	-46.2(6)
C2_1–N3_1–C10_1–C11_1	81.1(10)
N4_1–N3_1–C10_1–C11_1	-89.9(9)
C2_1–N3_1–C10_1–C15_1	-98.4(10)
N4_1–N3_1–C10_1–C15_1	90.7(9)
C15_1–C10_1–C11_1–C12_1	3.3(13)
N3_1–C10_1–C11_1–C12_1	-176.1(7)
C15_1–C10_1–C11_1–C16_1	-179.1(8)
N3_1–C10_1–C11_1–C16_1	1.5(12)
C10_1–C11_1–C12_1–C13_1	-1.9(13)
C16_1–C11_1–C12_1–C13_1	-179.6(8)
C11_1–C12_1–C13_1–C14_1	-0.9(14)
C12_1–C13_1–C14_1–C15_1	2.6(15)
C11_1–C10_1–C15_1–C14_1	-1.7(13)
N3_1–C10_1–C15_1–C14_1	177.6(7)
C11_1–C10_1–C15_1–C19_1	173.7(8)
N3_1–C10_1–C15_1–C19_1	-6.9(12)
C13_1–C14_1–C15_1–C10_1	-1.3(14)
C13_1–C14_1–C15_1–C19_1	-176.8(9)
C10_1–C11_1–C16_1–C18_1	128.8(9)

C12_1–C11_1–C16_1–C18_1	-53.7(11)
C10_1–C11_1–C16_1–C17_1	-107.9(10)
C12_1–C11_1–C16_1–C17_1	69.6(10)
C10_1–C15_1–C19_1–C20_1	130.2(9)
C14_1–C15_1–C19_1–C20_1	-54.7(12)
C10_1–C15_1–C19_1–C21_1	-107.1(10)
C14_1–C15_1–C19_1–C21_1	68.1(12)
N4_1–N5_1–C22_1–C27_1	-87.8(9)
C3_1–N5_1–C22_1–C27_1	90.6(11)
N4_1–N5_1–C22_1–C23_1	89.6(9)
C3_1–N5_1–C22_1–C23_1	-91.9(10)
C27_1–C22_1–C23_1–C24_1	-2.1(13)
N5_1–C22_1–C23_1–C24_1	-179.4(7)
C27_1–C22_1–C23_1–C28_1	171.3(9)
N5_1–C22_1–C23_1–C28_1	-6.0(12)
C22_1–C23_1–C24_1–C25_1	1.2(12)
C28_1–C23_1–C24_1–C25_1	-172.3(9)
C23_1–C24_1–C25_1–C26_1	0.2(14)
C24_1–C25_1–C26_1–C27_1	-0.8(15)
C23_1–C22_1–C27_1–C26_1	1.6(14)
N5_1–C22_1–C27_1–C26_1	178.8(8)
C23_1–C22_1–C27_1–C31_1	-176.9(9)
N5_1–C22_1–C27_1–C31_1	0.3(14)
C25_1–C26_1–C27_1–C22_1	-0.1(15)
C25_1–C26_1–C27_1–C31_1	178.5(9)
C24_1–C23_1–C28_1–C30_1	81.4(10)
C22_1–C23_1–C28_1–C30_1	-91.6(9)
C24_1–C23_1–C28_1–C29_1	-42.4(11)
C22_1–C23_1–C28_1–C29_1	144.7(8)
C22_1–C27_1–C31_1–C33_1	-145.1(9)
C26_1–C27_1–C31_1–C33_1	36.5(13)
C22_1–C27_1–C31_1–C32_1	89.0(11)
C26_1–C27_1–C31_1–C32_1	-89.5(11)
Pt1_2–C1_2–C2_2–C3_2	-1.7(10)
Pt1_2–C1_2–C2_2–N3_2	178.1(6)
C3_2–C2_2–N3_2–N4_2	0.1(8)
C1_2–C2_2–N3_2–N4_2	-179.7(8)
C3_2–C2_2–N3_2–C10_2	-171.8(7)
C1_2–C2_2–N3_2–C10_2	8.4(13)
C1_2–C2_2–C3_2–N5_2	179.5(7)
N3_2–C2_2–C3_2–N5_2	-0.4(8)
C1_2–C2_2–C3_2–P1_2	-7.0(9)
N3_2–C2_2–C3_2–P1_2	173.2(5)
C7_2–P1_2–C3_2–N5_2	61.5(9)
C4_2–P1_2–C3_2–N5_2	-55.9(10)
Pt1_2–P1_2–C3_2–N5_2	-179.7(8)
C7_2–P1_2–C3_2–C2_2	-108.3(6)
C4_2–P1_2–C3_2–C2_2	134.2(6)
Pt1_2–P1_2–C3_2–C2_2	10.5(5)
C2_2–N3_2–N4_2–N5_2	0.2(8)
C10_2–N3_2–N4_2–N5_2	172.5(7)
C7_2–P1_2–C4_2–C5_2	-169.7(7)
C3_2–P1_2–C4_2–C5_2	-55.9(8)
Pt1_2–P1_2–C4_2–C5_2	57.8(7)
C7_2–P1_2–C4_2–C6_2	-41.1(9)
C3_2–P1_2–C4_2–C6_2	72.7(9)
Pt1_2–P1_2–C4_2–C6_2	-173.6(7)
N3_2–N4_2–N5_2–C3_2	-0.4(8)
N3_2–N4_2–N5_2–C22_2	178.8(6)
C2_2–C3_2–N5_2–N4_2	0.5(8)
P1_2–C3_2–N5_2–N4_2	-169.5(7)
C2_2–C3_2–N5_2–C22_2	-178.6(7)
P1_2–C3_2–N5_2–C22_2	11.4(14)
C3_2–P1_2–C7_2–C8_2	63.0(7)
C4_2–P1_2–C7_2–C8_2	-179.8(6)
Pt1_2–P1_2–C7_2–C8_2	-46.0(7)
C3_2–P1_2–C7_2–C9_2	-174.4(7)
C4_2–P1_2–C7_2–C9_2	-57.2(8)
Pt1_2–P1_2–C7_2–C9_2	76.6(7)
N4_2–N3_2–C10_2–C11_2	97.1(10)
C2_2–N3_2–C10_2–C11_2	-91.8(11)
N4_2–N3_2–C10_2–C15_2	-83.7(10)
C2_2–N3_2–C10_2–C15_2	87.4(10)
C15_2–C10_2–C11_2–C12_2	-1.8(15)

N3_2–C10_2–C11_2–C12_2	177.4(9)
C15_2–C10_2–C11_2–C16_2	175.0(10)
N3_2–C10_2–C11_2–C16_2	-5.8(14)
C10_2–C11_2–C12_2–C13_2	2.9(16)
C16_2–C11_2–C12_2–C13_2	-173.8(11)
C11_2–C12_2–C13_2–C14_2	-2.9(17)
C12_2–C13_2–C14_2–C15_2	1.7(15)
C11_2–C10_2–C15_2–C14_2	0.6(14)
N3_2–C10_2–C15_2–C14_2	-178.5(7)
C11_2–C10_2–C15_2–C19_2	-179.6(9)
N3_2–C10_2–C15_2–C19_2	1.2(13)
C13_2–C14_2–C15_2–C10_2	-0.5(13)
C13_2–C14_2–C15_2–C19_2	179.7(9)
C10_2–C11_2–C16_2–C17_2	-87.4(14)
C12_2–C11_2–C16_2–C17_2	89.2(15)
C10_2–C11_2–C16_2–C18_2	147.1(10)
C12_2–C11_2–C16_2–C18_2	-36.3(16)
C10_2–C15_2–C19_2–C21_2	131.7(9)
C14_2–C15_2–C19_2–C21_2	-48.5(11)
C10_2–C15_2–C19_2–C20_2	-106.7(10)
C14_2–C15_2–C19_2–C20_2	73.0(11)
N4_2–N5_2–C22_2–C27_2	93.8(9)
C3_2–N5_2–C22_2–C27_2	-87.1(10)
N4_2–N5_2–C22_2–C23_2	-85.1(9)
C3_2–N5_2–C22_2–C23_2	94.0(10)
C27_2–C22_2–C23_2–C24_2	1.9(13)
N5_2–C22_2–C23_2–C24_2	-179.3(7)
C27_2–C22_2–C23_2–C28_2	-170.9(8)
N5_2–C22_2–C23_2–C28_2	7.9(12)
C22_2–C23_2–C24_2–C25_2	0.5(13)
C28_2–C23_2–C24_2–C25_2	173.6(9)
C23_2–C24_2–C25_2–C26_2	-2.8(15)
C24_2–C25_2–C26_2–C27_2	2.9(14)
C23_2–C22_2–C27_2–C26_2	-1.8(13)
N5_2–C22_2–C27_2–C26_2	179.4(7)
C23_2–C22_2–C27_2–C31_2	172.0(8)
N5_2–C22_2–C27_2–C31_2	-6.8(12)
C25_2–C26_2–C27_2–C22_2	-0.6(12)
C25_2–C26_2–C27_2–C31_2	-174.4(8)
C24_2–C23_2–C28_2–C30_2	45.5(12)
C22_2–C23_2–C28_2–C30_2	-142.0(8)
C24_2–C23_2–C28_2–C29_2	-77.0(11)
C22_2–C23_2–C28_2–C29_2	95.5(10)
C22_2–C27_2–C31_2–C32_2	-90.9(10)
C26_2–C27_2–C31_2–C32_2	82.5(10)
C22_2–C27_2–C31_2–C33_2	145.9(8)
C26_2–C27_2–C31_2–C33_2	-40.7(11)

9. Q-Band (34 GHz) and X-band (9.5 GHz) EPR

9.1 EPR sample preparation

All samples were prepared in a nitrogen-filled glovebox. For Q-band (34 GHz) measurements, 10–20 mM solutions of the precursors (P-diazoalkene **5** and diazoalkene Pt and Pd complexes **6** and **7**) in thoroughly degassed, dry solvents were transferred into 1.6 mm o.d. quartz tubes (~1 cm filling height), cooled to −40 °C for 15 min, sealed with BRAND sealing compound (~5 mm) and immediately flash-frozen in liquid nitrogen outside the glovebox. Toluene was used for **5** and **7**, and DCM for **6**. For X-band (9.5 GHz) measurements, a 20 mM toluene solution of **5** was transferred into a 4.8 mm o.d. quartz tube (~3 cm filling height), sealed with the sealing compound (~1 cm length) and immediately flash-frozen in liquid nitrogen outside the glovebox. All samples were covered in aluminum foil inside the glovebox and transported to the spectrometers under exclusion of light.

9.2 EPR experimental details

Q-Band pulse EPR measurements were carried out in the temperature range of 5–10 K using a Bruker Elexsys E580 spectrometer equipped with a 150 W TWT amplifier, Bruker EN 5107D2 resonator, Oxford Instruments CF935 continuous-flow helium cryostat and Oxford Instruments MercuryiTC temperature controller. Field-swept EPR spectra were detected via the electron spin echo (ESE) and/or free induction decay (FID) signal. For the ESE detection, the microwave (MW) $\pi/2$ pulse was 14 ns; the inter-pulse delay τ was 500 ns. For the FID detection, the $\pi/2$ pulse was 500 ns. In order to generate triplet species, the diazo precursors were illuminated in frozen solutions at 10 K using a Hg arc lamp (LOT LSB610U) inside the resonator for approx. 40–50 minutes.

X-Band CW EPR stability measurements in the temperature range of 95–125 K were carried out using a benchtop Magnettech ESR5000 spectrometer. The spectra were recorded under non-saturating conditions (1 mW); the modulation amplitude was 9.5 G. The triplet species was generated by illuminating the diazo precursor **5** (20 mM) in frozen toluene solution at 95 K inside the resonator until the triplet species intensity reached a plateau (~30 min). A 395 nm LED (Thorlabs M395FP1) was used for illumination.

Orientation-selective Davies ENDOR[11] spectra were collected with stochastic detection[12] at 6 K using an AR 600 W radiofrequency (RF) amplifier (AR 600A225A). The following microwave pulse sequence was used: π−T−$\pi/2$−τ−π−τ−echo. The RF pulse was applied during the time interval T and had a length of 30 µs; the MW inversion π pulse was 28−30 ns; the $\pi/2$ and π detection pulses were 14 and 28 ns, respectively; the inter-pulse delay τ was 340 ns.

EPR and ENDOR simulations were performed using the *EasySpin* package.[13] The intensity of the simulated half-field ($M_S = -1 \leftrightarrow 1$) EPR signal at ~560 mT was manually reduced by 88% to account for a difference in transition probabilities (Figure S118). To minimize the number of simulation parameters, an isotropic g-factor $g_{iso} = 2.0023$ was used.

9.3 EPR Data

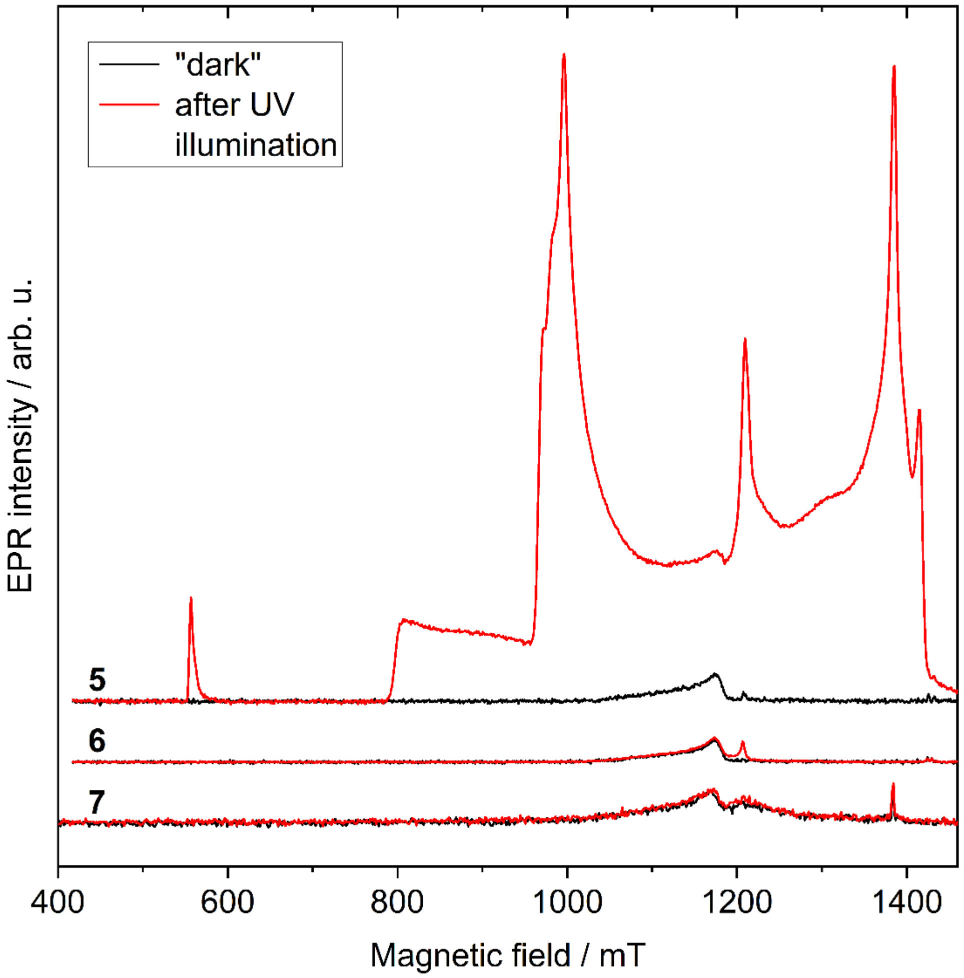

Fig. S118: Q-band EPR spectra of **5**, **6** and **7** before (black traces) and after (red traces) UV photolysis at 10 K. The triplet signal detected after illumination of **5** is assigned to the triplet vinylidene **10** (see main text). Large ZFS *D* parameters of the metallovinylidenes **8** and **9** make them inaccessible to Q-band EPR.

9.4 Stability measurements

The decay of **10** in frozen toluene solution (Fig. S119) starts at ~108 K, well below the glass transition temperature of toluene of T_g = 117 K, making it one of the least stable triplet vinylidenes based on a five-membered ring described to date.[14

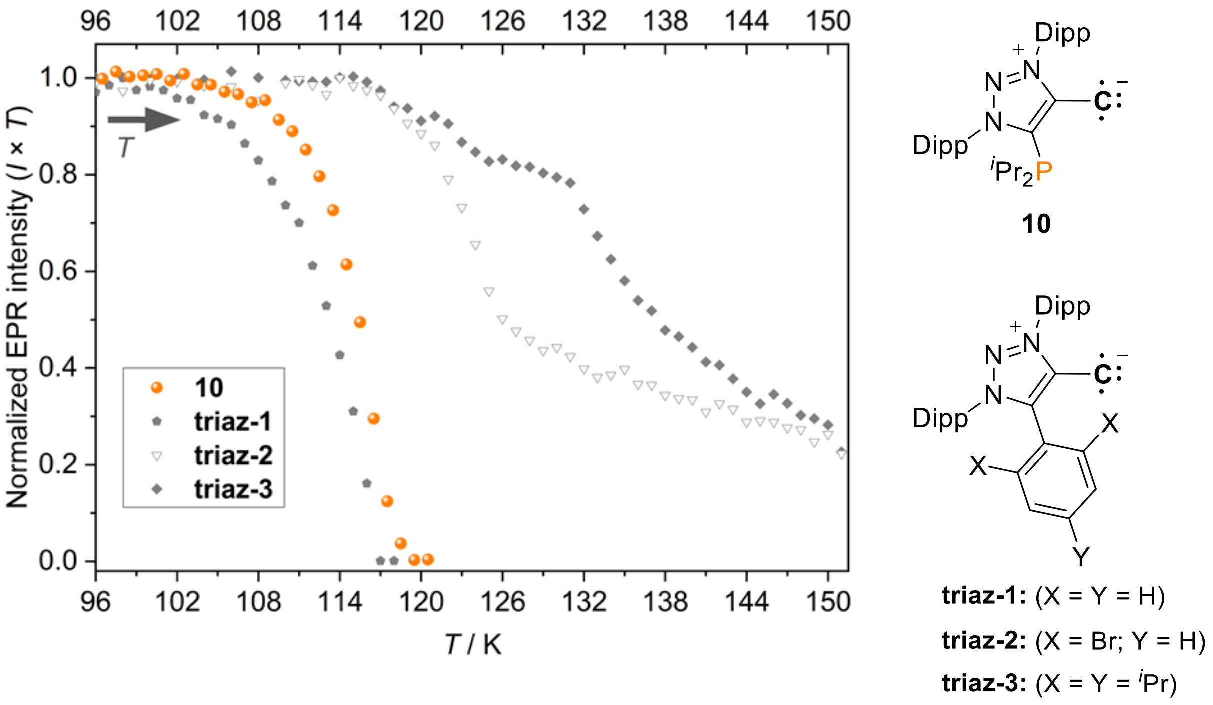

Fig S119: Normalized EPR intensity of triplet vinylidene **10** (orange spheres) measured at increasing temperatures following UV illumination of **5** at 95 K in frozen toluene solution (approx. 3 min per temperature point). For comparison, data is shown for three previously described vinylidenes[14] (gray symbols) based on the 1,2,3-triazole ring. The respective structures are shown on the right.

9.5 Analysis of the ^{14}N and ^{31}P ENDOR spectra

The high-intensity ENDOR features of **10** (Figure S120, left panel) could be simulated by introducing a single ^{14}N nucleus with a predominantly isotropic HF tensor and a quadrupole interaction (QI) tensor resolved along the X and Z directions of the ZFS. Both HF and QI tensors (see Table S5) are very close to those of the previously described 1,2,3-triazole-based vinylidenes[14] (the nitrogen atom was identified as the one closest to the terminal carbon). The set of simulation parameters was not unique and was obtained by assuming collinearity of the three tensors (ZFS, HF, QI) to reduce the number of fitting parameters.

Analysis of the ^{31}P HF tensor of **10** (Figure S120, right panel) was hindered by the low intensity of the ^{31}P ENDOR features. While ENDOR lines attributable to ^{31}P in a weak-coupling regime were clearly detected at the Y^- and Y^+ canonical field positions (~12 and 29 MHz, respectively), no clear features could be detected at X^-. Furthermore, the Z^- position falls outside of the

available field range of the magnet, creating an ambiguity, where the Z⁺ ENDOR features could be simulated using either $A_z(^{31}P)$ = −2.9 or +30.2 MHz. However, the latter produced an overall worse match to the experiment than the former (data not shown). Thus, only the $A_y(^{31}P)$ = −5.4 MHz component could be determined with confidence, while the $A_x(^{31}P)$ value is the least reliable one. We note that the computed ^{31}P HF tensor (see SI **12.4**) was strongly dependent on the conformation of the molecule, which was determined in our experiments by the initial conformation of the diazo-precursor (in contrast to the ^{14}N hf tensor).

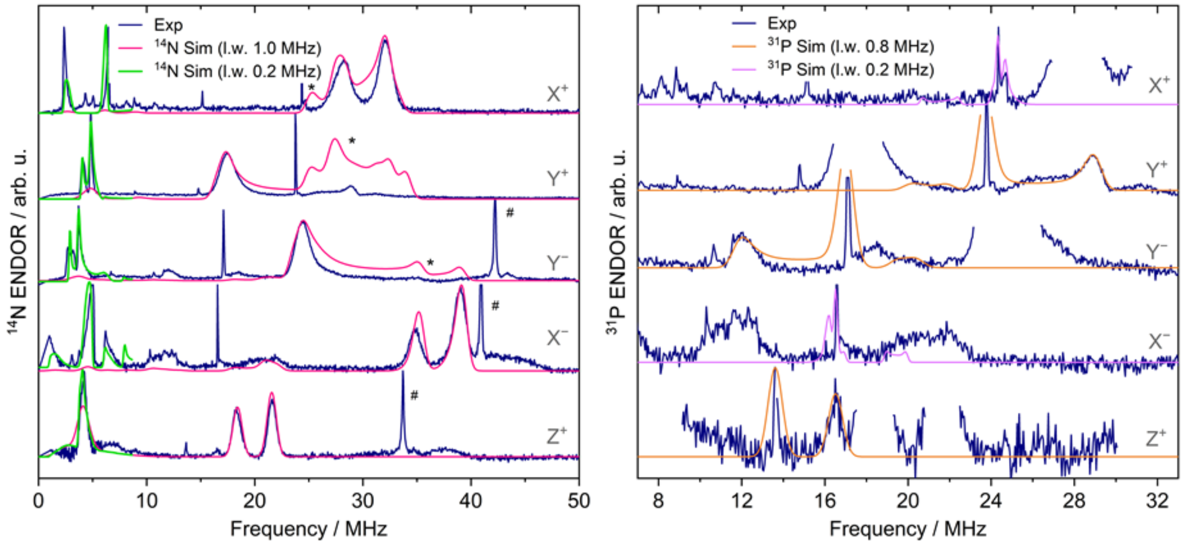

Fig S120: Davies ENDOR spectra of **10** recorded at the canonical field positions (dark blue traces) with simulations performed for ^{14}N (left) and ^{31}P (right) using two values of the FWHM line width. Asterisks (*) mark ^{14}N spectral features originating from non-canonical orientations, which are suppressed in the experiment; pound signs (#) mark ^{1}H spectral features. On the right-hand panel the spectra were expanded due to the low intensity of the ^{31}P spectral features; strong ^{14}N lines were omitted for clarity.

Table S5. Experimentally fitted ^{14}N HF and QI tensors and ^{31}P HF tensor (in MHz) for **10** in frozen toluene solution. [a] All fitted tensors were assumed collinear with the ZFS tensor; [b] the experimentally fitted ^{14}N QI tensor possesses a non-zero trace, indicating a deviation from the assumed collinearity. [c] $A_y(^{31}P)$ = −0.27 MHz was tentatively assigned based on the peak detected at the X⁺ field position.

	A_x	A_y	A_z	α [a]	β	γ	Q_x	Q_y	Q_z	α	β	γ
^{14}N	35.80	21.80	23.02	0	0	0	−1.6	0	0.8 [b]	0	0	0
^{31}P	(−0.27) [c]	−5.43	−2.90	0	0	0						

10. THz-EPR and IR spectroscopy

Sample preparation

The sample for infrared (IR) spectroscopy was prepared by mixing about 0.5 mg of Pt-viniylidene (**8**) and 120 mg KBr in an agate mortar and pressed to a pellet of 10 mm diameter at about 1000 psi under N_2 atmosphere. The sample was transferred in a bath of liquid nitrogen to the cryostat of the spectrometer.

The sample for far-infrared (FIR) and THz-EPR spectroscopy was prepared by mixing 3.8 mg of Pt-viniylidene (**8**) and 115 mg HD-PE in an agate mortar and pressed to a pellet of 10 mm diameter at about 1000 psi under N_2 atmosphere. The sample was transferred in a bath of liquid nitrogen to the variable temperature insert (VTI) of the superconducting magnet of the spectrometer.

IR spectroscopy under illumination

IR spectroscopy measurements were performed using a Bruker IFS 125 FTIR spectrometer with the OPUS 8.5 software. Figure S121 (A) shows an overview of the experimental setup. The spectrometer was equipped with a KBr beamsplitter, a helium gas flow cryostat (Bruker Optistat) and a liquid N_2 cooled HgCdTe detector (MCT-12.5-2, InfraRed associates) with an MCT-1000H preamplifier (InfraRed associates). The sample was irradiated with a fiber-coupled LED (Thorlabs) of 395 nm at a power of 3.4 mW measured in front of the window of the cryostat. The light from the LED was guided perpendicular to the MIR beam while the sample was rotated to match both, the LED and the MIR radiation. Spectra were recorded before and during the irradiation at a temperature of 10 K and a resolution of 2 cm^{-1}.

THz-EPR spectroscopy

Frequency-domain Fourier-transform THz electron paramagnetic resonance (FD-FT THz-EPR) spectroscopy was performed at the THz beamline of BESSY II. In Figure S121 (B) an overview of the experimental setup is depicted, and the details of the THz-EPR setup are described elsewhere.[15] Unpolarized THz radiation from the Hg lamp of the Bruker IFS 125 FTIR spectrometer, which was equipped with a 6 µm Mylar beamsplitter, was guided to the sample which was placed in the variable temperature insert (VTI) of a superconducting 12 T magnet (Cryogenic, Inc.). The measurement was performed at a temperature of 5 K in Voigt geometry in transmission mode and the signal was detected by a Si-bolometer (HDL-5, Infrared Labs) cooled to 4.2 K with liquid He. Prior magnetic measurements the sample was irradiated at 10 K by an LED (385 nm, Roschwege Star-UV385) at a power of 0.4 mW measured after the passing the empty VTI. The beam was guided colinear to the THz beam.

During LED irradiation, THz spectra were recorded continuously to follow the process of the photo reaction. FD-FT THz-EPR spectra were acquired by dividing two raw spectra recorded at different magnetic fields referred to as magnetic-field division spectra (MDS). Numerical simulations were performed using the EasySpin toolbox for MATLAB.[16,17]

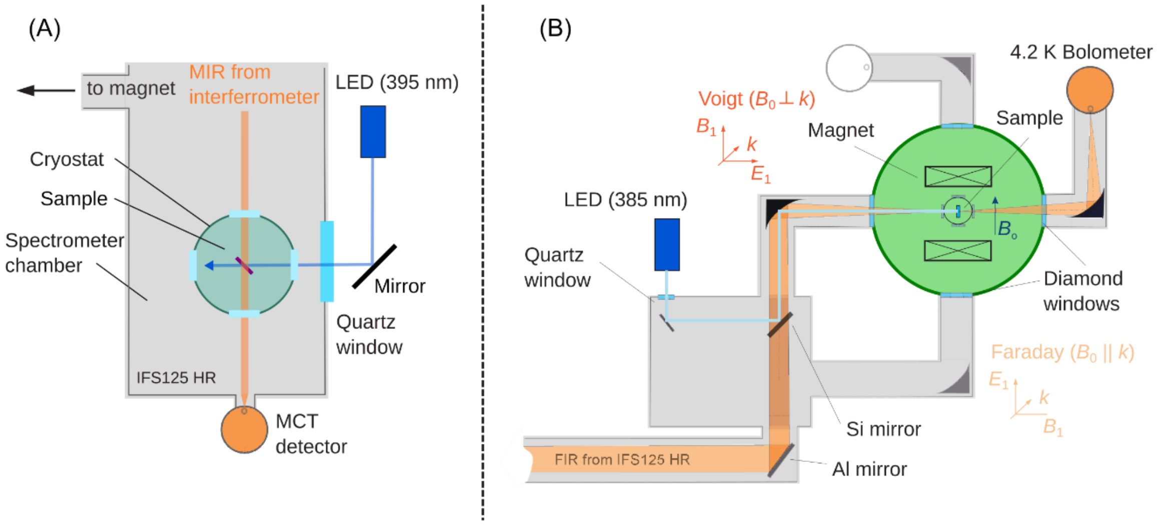

Fig. S121: Schematic of the experimental setup for IR (A) and THz-EPR (B) measurements. A detailed description on the experimental procedure is given in the text.

Results IR spectroscopy

In order to perform THz-EPR measurements on **8** we first investigated and optimized the irradiation of a sample pellet of the diazo precursor **6** by infrared spectroscopy at cryogenic temperatures (10 K). The IR transmission spectrum of **6** shows the characteristic stretch vibration band of the $PtCN_2$ bond at 2038 cm^{-1} (Figure S122(A)). Upon photoexcitation with a 395 nm LED, N_2 loss can be monitored by the decrease of the vibration band at 2038 cm^{-1}. Furthermore, a band at 1506 cm^{-1} decreases simultaneously. The decrease saturated after about 17 hours, however, turning the sample pellet to irradiate it from the backside, showed a rapid further decrease up to almost complete conversion.

Results THz-EPR spectroscopy

THz-EPR measurements were performed in a range from 50 to 700 cm^{-1} using a higher sample amount as compared to the IR experiments described above. Furthermore, HDPE was used as matrix material to prepare sample pellets. The prior illumination was guided by the results of the IR experiments, while THz spectra were continuously recorded to track the process of the conversion of the diazo precursor **6** to the Pt-vinylidene **8**. Figure S122(B) shows

the spectral changes during irradiation which were calculated by dividing a spectrum recorded at a particular illumination time by a spectrum taken in the dark before irradiation. The illumination was performed until a saturation of the changes occurred, while it was necessary to illuminate the sample pellet from both sides to optimize the conversion.

Subsequently, the illumination was stopped and THz-EPR measurements were directly performed at 5 K. The magnetic-field-division spectra, depicted in Figure S122(C), clearly show a magnetic-field-dependent signal around 125 cm^{-1}. The observed "up-down-up" pattern is indicative for at triplet spin state with an axial ZFS (*D*) and a small to vanishing rhombicity (*E*): At 0 T the transitions between the M_s = 0 and M_s = ± 1 manifolds are degenerated in case of *E* = 0 resulting in a narrow absorption line, while at higher fields the resonance gets broader as the M_s = ± 1 levels are more and more split by the electron Zeeman interaction (Figure S122(D)). Thus, dividing lower by higher magnetic field results in signals > 1 at the outer edges and < 1 in the center.

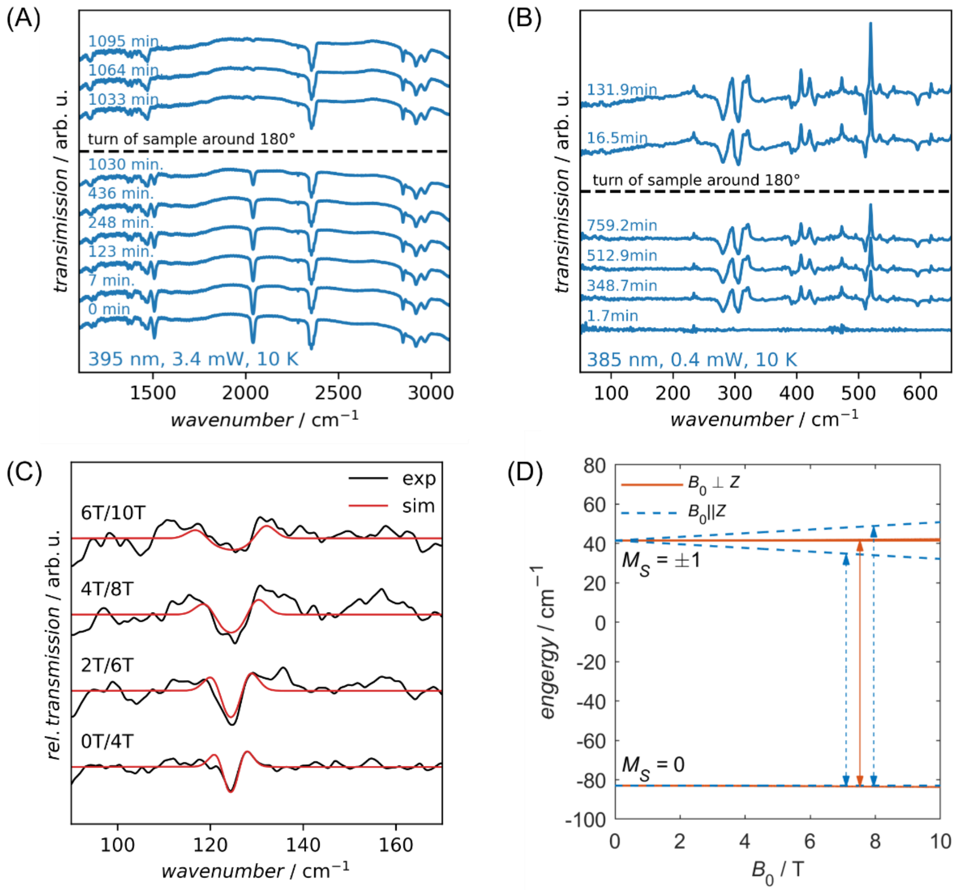

Fig S122: (A) Transmission IR spectra of the diazoalkane precursor **6** during light irradiation at 395 nm at a temperature of 10 K. (B) THz division spectra of the diazoalkene precursor **6** at several time points during illumination at 385 nm at a temperature of 10 K indicating the conversion to triplet Pt-vinylidene **8**. (C) Magnetic-field-division FD-FT THz-EPR spectra of triplet Pt-vinylidene **8** after completed conversion and stopped illumination at a temperature of 5 K (black) and numerical simulation of the spectra (red, see text for details). (D) Magnetic-field-dependent spin energy level diagram of a triplet spin system using identical parameters

as for the simulated spectra in (C) for a magnetic field orientation parallel and perpendicular to the unique axis (*Z*) of the axial ZFS tensor. The allowed transitions are indicated with double arrows.

The signals could be simulated with excellent agreement using the triplet spin Hamiltonian

$$\hat{H} = D\,(\widehat{S_z^2} - S^2 / 3) + \mu_B\, B_0\, g\, \hat{S},$$

where *D* and *E* are the axial and rhombic ZFS parameters, respectively, *g* is the *g*-value and μ_B the Bohr magneton. Optimization of the parameters yielded *g* = 2.0(2), *D* = 124.5(5) cm^{-1}, *E*/*D* = 0 and a gaussian linewidth of 4.0 cm^{-1}. Errors were estimated by manually changing the parameters and visually verifying the simulation results with the experimental spectra. *g* was considered isotropic as no direct signs of *g*-anisotropy are visible in the spectra. However, unresolved *g*-anisotropy or deviations from the free electron value can contribute to the linewidth. Therefore, its value was fixed to 2.0 while optimizing the other parameters. Afterwards the *g* value was varied to estimate its error. The rather broad linewidth can be explained by the relative high transition energy arising from the high ZFS and is most likely dominated by *D* strain. However, an inhomogeneous linewidth model was sufficient to fit the data. The rhombicity |*E*/*D*| was set to zero, but within the signal-to-noise ratio a value up to 0.01 would be possible to represent the experiment.

THz-EPR on Pd-vinylidene

The Pd-vinylidene was investigated in the same manner. Additionally, it was studied down to energies of 3 cm^{-1} using both, pressed pellets and frozen toluene solution samples with higher sample amounts (~20 mg) Therefore, the spectrometer configuration was changed accordingly (using coherent synchrotron irradiation, 1.6 K Bolometer, 50 um, or 125 um beamsplitters). However, none of these experiments yielded an EPR signal.

11. SQUID Magnetometry

a) The Setup

SQUID measurements were done on a Quantum Design MPMS3 SQUID. The irradiation products **8** and **9** are not stable at room temperature. Therefore, the irradiation must be done *in situ* using a fiber optical sample holder (FOSH) to get optical access to the sample in the magnetometer. To get maximal light intensity in the fiber a 390 nm (max 52 W) Kessil LED lamp was used. To couple the LED into a fiber a photography SLR lens with 28 mm focal length and *f* 2.8 was used. It was connected to a 3D printed mount that fixed a collimator at the center position of a camera sensor. The wide angle of the lens focuses the light on one spot and the internal construction simplifies the alignment of lens and light source. The internals of the 3D printed connector were covered with reflective tape to maximize efficiency. In addition to that, the path between LED and lens was covered by a tube with a reflective inner wall.

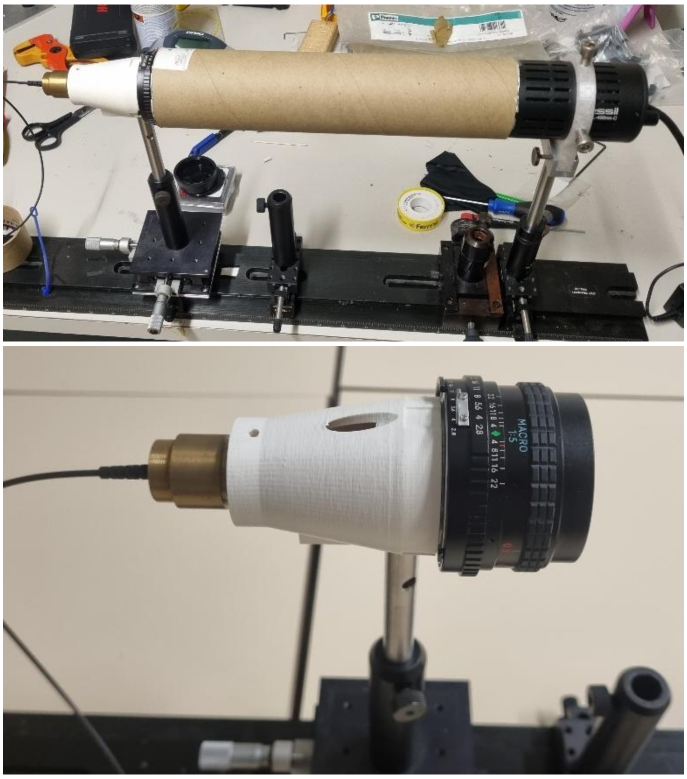

Fig. S123: Picture of the irradiation setup.

b) Pt-Vinylidene

The SQUID sample holder was filled with **6** in the glovebox under red light. In the SQUID magnetometer a full series of magnetization and susceptibility measurements were performed before irradiation. These measurements show that **6** is diamagnetic.

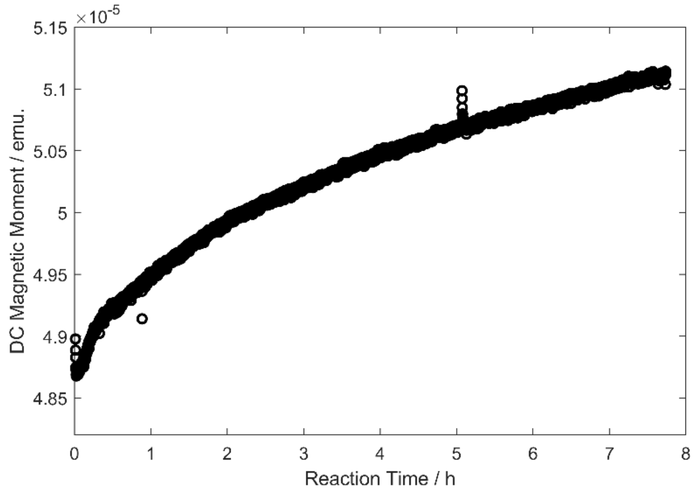

Fig. S124: Measurement of the magnetic moment at the sample position during the illumination at 10 K. The increase in magnetic moment is proportional to the formation of **8** in the SQUID.

For the illumination the temperature as set to 10 K and the magnetic moment of the sample was measured continuously for 8 h. The effect of the illumination is plotted in Figure S124.

After Illumination the magnetization measurements at low temperatures were performed first before measuring susceptibility from 1.8-300 K. To confirm the thermal decomposition of the sample, susceptibility was measured for a second time. (Figure S125)

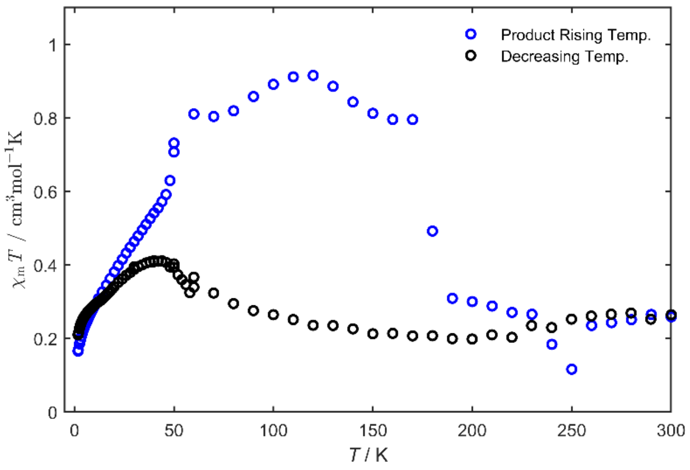

Fig. S125: $\chi_m^p T$ over T plot of **8** after illumination from 1.8-300 K (blue) and back down from 300-1.8 K (black) the difference below 180 K shows the irreversible decomposition of **8** in the first measurement.

Overall, the amount of paramagnetic species formed in the magnetometer is very little. Even though the paramagnetic susceptibility χ^p is much stronger than the diamagnetic susceptibility

χ^{dia}, it was necessary to use the raw data of **6** (before the illumination) for a diamagnetic correction of the product. The $\chi_m^p T$ over T (Figure S126A) and the χ_m^p over T plot (Figure S126B) show clearly the formation of a paramagnetic species.

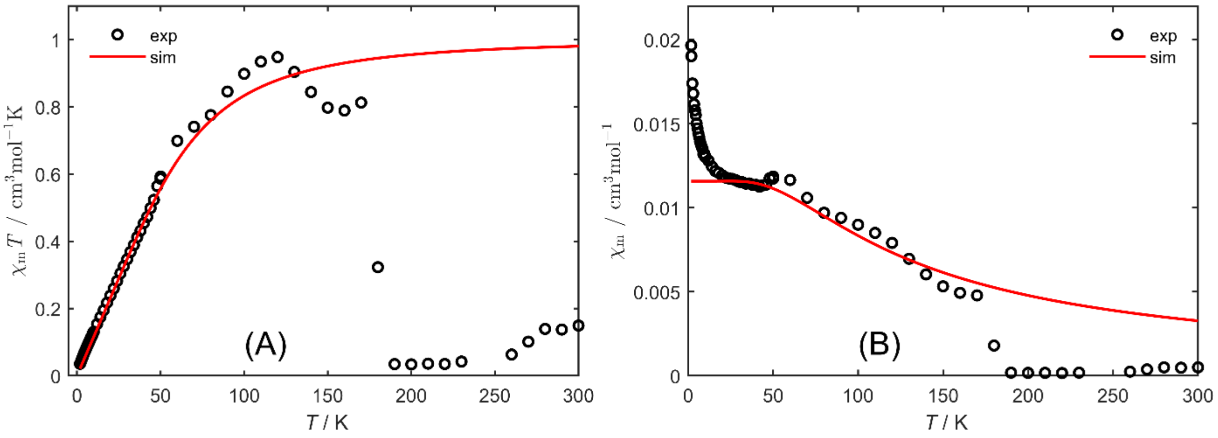

Fig. S126: (A) $\chi_m^p T$ over T plot from 1.8-300 K of **8** after correction with raw data from **6** before illumination and simulation of the data in red. (B) χ_m^p over T plot of the same dataset with the simulation based on the same parameters shown in Table S6.

The shape of the $\chi_m^p T$ over T plot indicates towards a large zero-field splitting (ZFS) which can be modelled well by the spin Hamiltonian. The parameters of the simulation are shown in Table S6.

Table S6: Parameters for the spin Hamiltonian simulations for **8** in Figure S126 and Figure S127.

**Parameter**	**Value**
Spin *S*	1
g value	2
ZFS *D* [cm^{-1}]	120(5)
ZFS *E/D*	0.0(1)

The comparison between the plots show that the fit is overall good but at low temperatures small deviations are present that are especially pronounced in the χ_m^p over T plot (Figure S126B). These deviations are also present when looking at the magnetization curves between 1.8-20 K. (Figure S127) The origin of the deviations could be a small paramagnetic impurity with a smaller ZFS that is most pronounced at very low temperatures.

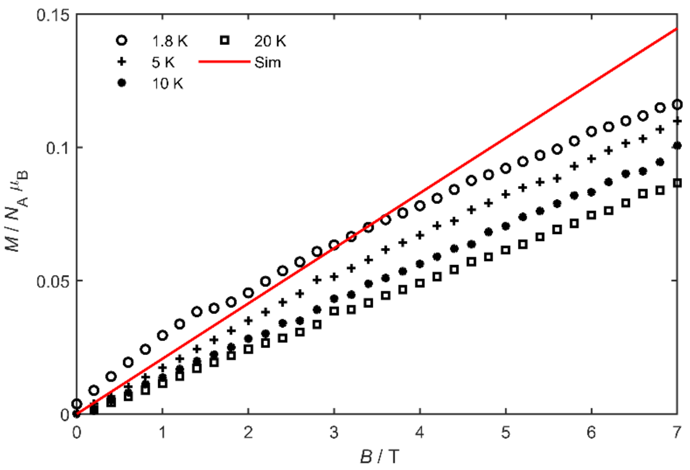

Fig. S127: Magnetization over Field plots between 1.8-20 K of **8** after correction with raw data from **6** before illumination and simulation of the data in red based on parameters in Table S6.

c) Pd-Vinylidene

The measurements and evaluations on the palladium complex **7** / **9** are done accordingly to the previously shown platinum complex **6** / **8**.

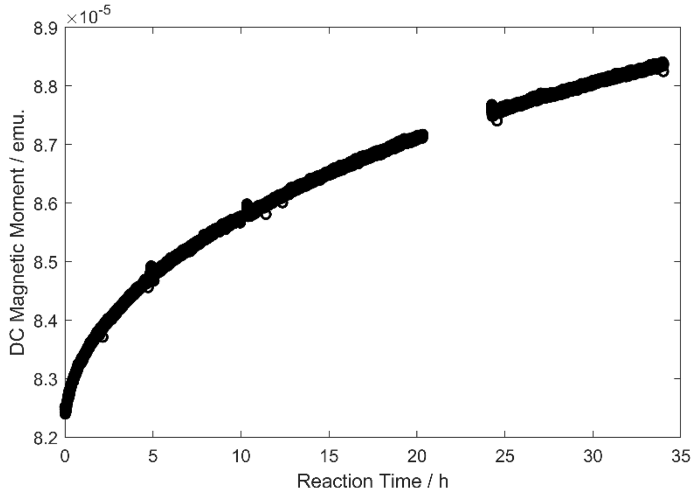

Fig. S128: Measurement of the magnetic moment at the sample position during the illumination at 10 K. The increase in magnetic moment is proportional to the formation of **9** in the SQUID.

The effect of illumination at 10 K of the complex in the SQUID shown in Figure S128. In this case the illumination lasted much longer to get as many paramagnetic species as possible.

After Illumination the magnetization measurements at low temperatures were performed first before measuring susceptibility from 1.8-300 K. To confirm the thermal decomposition of the sample, susceptibility was measured for a second time (Figure S129).

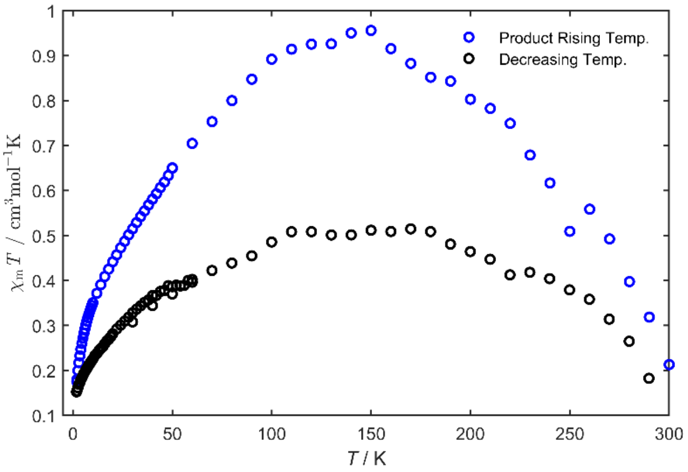

Fig. S129: $\chi_m^p T$ over T plot of **9** after illumination from 1.8-300 K (blue) and back down from 300-1.8 K (black) the difference below 180 K shows the irreversible decomposition of **9** in the first measurement.

Overall, the amount of paramagnetic species formed in the magnetometer is still very little. It was necessary to use the raw data of **7** (before the illumination) for a diamagnetic correction of the product. The $\chi_m^p T$ over *T* (Figure S130A) and the χ_m^p over *T* plot (Figure S130B) show clearly the formation of a paramagnetic species

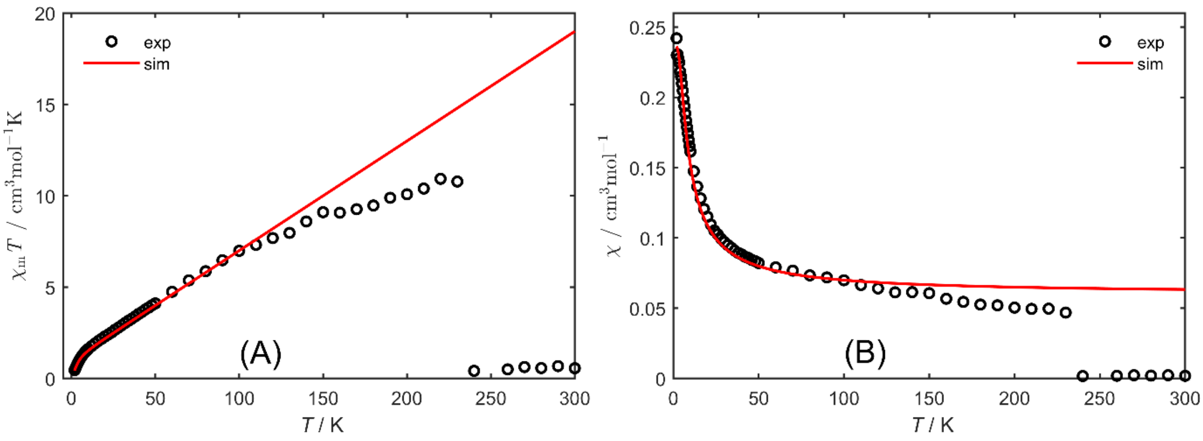

Fig. S130: (A) $\chi_m^p T$ over T plot from 1.8-300 K of **9** after correction with raw data from **7** before illumination and simulation of the data in red. (B) χ_m^p over T plot of the same dataset with the simulation based on the same parameters.

The shape of the $\chi_m^p T$ over *T* plot (Figure S130A) looks very different to the platinum complex. The bend at 10 K indicates a smaller ZFS and a temperature independent paramagnetism TIP. This becomes more evident when looking at χ_m^p over *T* plot (Figure S130B) and the corresponding simulations based on the parameters in Table S7.

Table S7: Parameters for the spin Hamiltonian simulations for **9** in Figure S130 and Figure S131.

**Parameter**	**Value**
Spin *S*	1
g value	2
ZFS *D* [cm^{-1}]	8.0(5)
ZFS *E/D*	0.1(1)
TIP [cm^3mol^{-1}]	0.060(1)

A look at the magnetization over field measurements in Figure S131 shows that the simulation with small ZFS fits this perspective of the dataset very well.

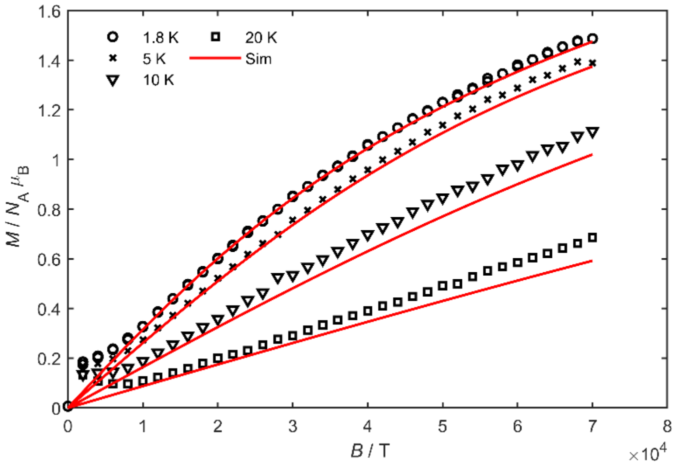

Fig. S131: Magnetization over Field plots between 1.8-20 K of **9** after correction with raw data from **7** before illumination and simulation of the data in red based on parameters in Table S7.

12. Computational Data

12.1 Computational Details

Geometries

All quantum chemical calculations were carried out with Orca 5.[18] Geometry optimizations were performed using the PBE0 functional,[19] which was found to best reproduce experimental metal-ligand bond distances of third-row transition-metal complexes.[20] Scalar relativistic effects were taken into account using the zero-order regular approximation (ZORA),[21,22,23,24] combined with ZORA-recontracted[25]versions of the def2 basis sets.[26] Triple-zeta basis sets were used for the ligands and the SARC-ZORA-TZVP basis set for Pt.[25] The resolution of identity approximation and the chain-of-spheres approximation to exact exchange[27](RIJCOSX in Orca convention) were employed to reduce computational time along with the SARC/J auxiliary basis set. Tight convergence criteria (TightSCF in Orca convention) were used in all calculations. Two structures were optimized for each metallovinylidene, one assuming a triplet and another a closed-shell singlet spin state. The triplet ground state structures were determined to be favored energetically by 8.6 and 9.6 kcal mol^{-1} for **8** and **9**, respectively, over the singlet ground state structures and were therefore used for property calculations in the rest of this work.

Coupled-cluster calculations

The vertical excitation energy from the triplet ground state to the closed-shell singlet excited state of simplified (Dipp groups replaced by -CH_3) models of **8** and **9** were calculated using a local correlation implementation of coupled-cluster theory, the domain-based local pair natural orbital coupled-cluster theory with singles, doubles, and perturbative triples excitations, DLPNO-CCSD(T).[28,29,30,31] All *ab initio* calculations were performed using the ZORA-def2-TZVP basis sets for C, N, P and Cl atoms, the ZORA-def2-SVP for H, and the SARC-ZORA-TZVPP basis set for Pt, along with large automatically generated auxiliary basis sets. Kohn−Sham determinants computed with the PBE0 functional were used as reference. Perturbative triple excitations were treated with the more accurate iterative (T1) approach,[31] as opposed to the semicanonical (T0),[29] which is adequate only for main group elements.[32] All electrons were included in the correlation treatment (NoFrozenCore in Orca convention). The NormalPNO (in Orca convention) default settings were used for all calculations. Two-point extrapolation of the correlation energy with respect to the pair natural orbitals (PNO) space[33] was performed using two different T_{CutPNO} cutoff values, $1{\cdot}10^{-6}$ and $3.33{\cdot}10^{-7}$, following a previously established protocol.[34,35]

Multireference wavefunction-based calculations

Complete active space self-consistent field (CASSCF) and 2nd-order N-electron valence state perturbation theory (NEVPT2)[36,37] calculations were performed at the DFT optimized geometries. Relativistic effects were considered with ZORA, but test calculations with the DKH2 and X2C Hamiltonians gave similar results for both energetics and magnetic properties. The active space of the metallovinylidenes includes 14 electrons in 11 orbitals, and that of the free vinylidene includes 10 electrons in 8 orbitals, which are shown in Figures S132a and b, respectively, along with their occupation numbers resulting from a state-averaged CASSCF calculation over 1 triplet and 2 singlet roots.

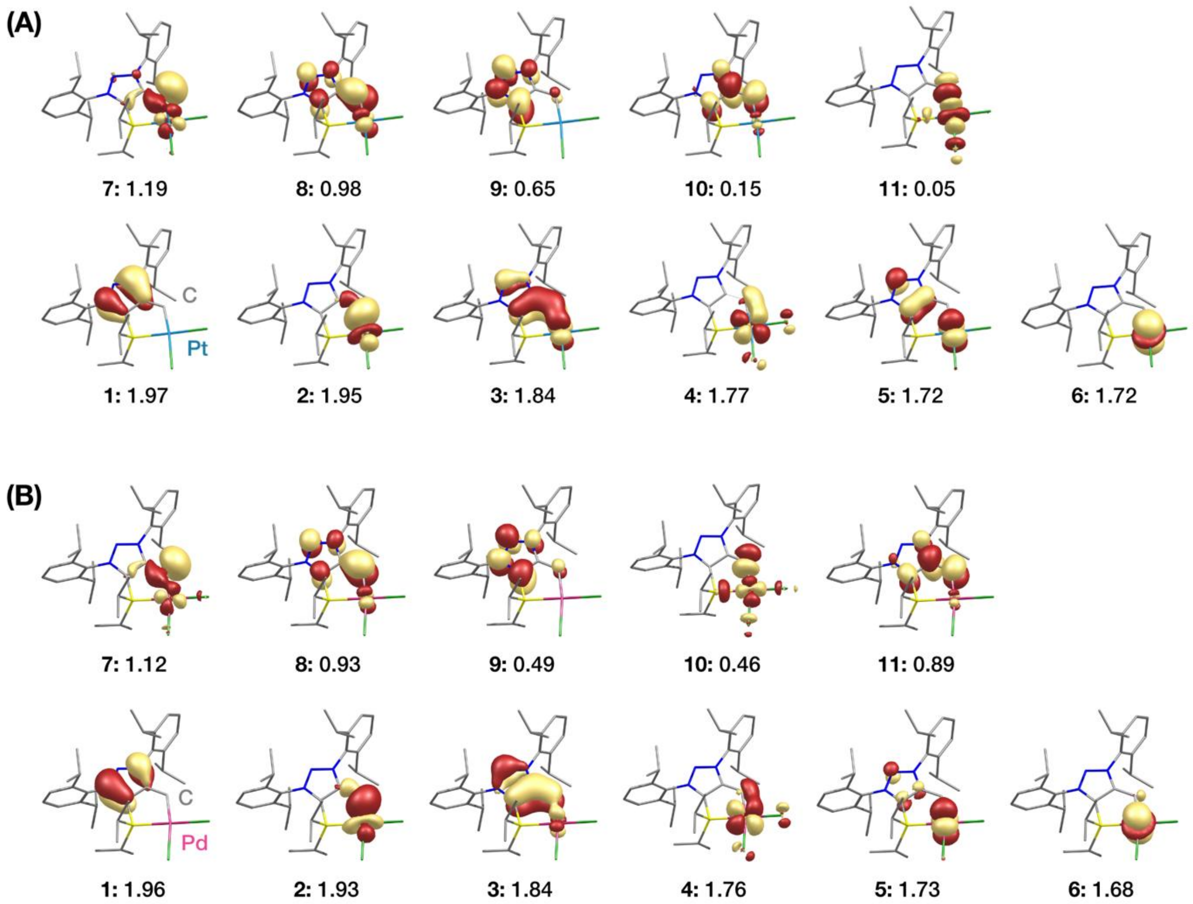

Fig S132: Active orbitals and natural occupation numbers of A) **8**, produced from a CASSCF(14,11) calculation averaged over 22 triplet and 22 singlet states, and B) **9**, produced from a CASSCF(14,11) calculation averaged over 35 triplet and 35 singlet states.

The zero-field splitting (ZFS) in compounds with S > 1/2 arises predominantly from the interaction of the ground state with electronically excited states as a result of spin-orbit coupling (SOC). Therefore, one needs to carefully choose the number of roots that will be included in the state-averaged CASSCF calculation, so that in addition to the ground state it provides a good description of the electronically excited states. Because including too many roots would

reduce the quality of the description of the ground state, one needs to include only the excited states that contribute considerably to the ZFS. This is obviously a system-dependent case-by-case optimization problem that requires testing different combinations of numbers of singlet, triplet and quintet roots to be included in the state-averaged CASSFF calculation until a converged description is acquired. Systematic investigations for the present systems showed that the combination of roots that provides a converged ZFS value while maintaining a good description of the ground state, is 22 triplet and 22 singlet states for **8**, and 35 triplet and 35 singlet states for **9**. Quintet states have zero contribution to the ZFS, therefore no quintet roots were included in the final state-averaged calculation. The effect of dynamic electron correlation was included by calculating the NEVPT2 energy correction for each one of these CASSCF states, which gives scalar relativistic excited states up to ~50,000 cm^{-1}. This energy range is sufficient to include all important SOC effects in the following QDPT calculation.[38] Subsequently, the spin-orbit coupling (SOC) contribution was computed by diagonalization of the SOC operator in the basis of the CASSCF states. For the SOC operator we use the spin-orbit mean field (SOMF) operator.[39,40] During this step, all magnetic sublevels $M_s = S, S–1, \ldots, –S$ that correspond to each scalar relativistic CASSCF state with $M_s = S$ were treated with quasi-degenerate perturbation theory (QDPT).

12.2 Vertical Singlet-Triplet Gaps

Table S8: Results of CASSCF/NEVPT2 calculations on compound **8**. The NEVPT2 energies of the singlet states relative to the triplet ground state are provided in eV. For each state, the ten leading configurations are shown along with their CI coefficients. The orbitals that describe the configurations are depicted in Figure S134.

T, S = 1 0.000 eV		S_1 (open-shell), S = 0 0.500 eV		S_2 (closed-shell), S = 0 0.913 eV	
CI coefficients	Configurations	CI coefficients	Configurations	CI coefficients	Configurations
0.8894	22222211000	0.8497	22222211000	0.7472	22222220000
0.0174	22222011200	0.0184	22222111100	0.1031	22222202000
0.0096	22222111100	0.0149	22222011200	0.0143	22222020200
0.0076	22220211200	0.0103	22222220000	0.0115	22222211000
0.0057	22112212010	0.0076	22220211200	0.0109	22222121000
0.0050	22222110200	0.0061	22112212010	0.0086	22112221010
0.0047	22022211020	0.0054	21212222000	0.0080	22212211010
0.0036	21122221010	0.0050	22222110200	0.0069	22202222000
0.0031	22222012100	0.0049	22222210001	0.0064	22220220200
0.0028	22220211101	0.0044	22222210100	0.0063	22222021100

Table S9: Results of CASSCF/NEVPT2 calculations on compound **9**. The NEVPT2 energies of the singlet states relative to the triplet ground state are provided in eV. For each state, the ten leading configurations are shown along with their CI coefficients. The orbitals that describe the configurations are depicted in Figure S134.

T, *S* = 1		S_1 (open-shell), *S* = 0		S_2 (closed-shell), *S* = 0	
0.000 eV		0.549 eV		0.948 eV	
CI coefficients	Configurations	CI coefficients	Configurations	CI coefficients	Configurations
0.8720	22222211000	0.8362	22222211000	0.7400	22222220000
0.0154	22222011200	0.0220	22222111100	0.0810	22222202000
0.0121	22222111100	0.0129	22222011200	0.0216	22122211010
0.0070	22202211020	0.0100	21222211010	0.0146	22222121000
0.0066	22112212010	0.0072	22112212010	0.0129	22222020200
0.0056	22220211200	0.0061	22202211020	0.0121	22112221010
0.0055	21222211010	0.0057	22220211200	0.0066	22022222000
0.0044	21212221010	0.0051	22222112000	0.0065	22222021100
0.0034	22221111101	0.0050	21212221010	0.0056	22202220020
0.0033	22222110200	0.0046	22222210100	0.0048	22222210010

Table S10: Results of CASSCF/NEVPT2 calculations on compound **10**. The NEVPT2 energies of the singlet states relative to the triplet ground state are provided in eV. For each state, the ten leading configurations are shown along with their CI coefficients. The orbitals that describe the configurations are depicted in Figure S134.

T, *S* = 1		S_1 (closed-shell), *S* = 0		S_2 (open-shell), *S* = 0	
0.000 eV		0.455 eV		0.623 eV	
CI coefficients	Configurations	CI coefficients	Configurations	CI coefficients	Configurations
0.9132	22221100	0.8752	22222000	0.9004	22221100
0.0152	22201120	0.0160	22220200	0.0136	22201120
0.0109	22021120	0.0159	22202020	0.0107	22021120
0.0067	22211101	0.0153	22211020	0.0089	22121110
0.0064	22121110	0.0137	2222200	0.0085	22220101
0.0063	22210120	0.0101	22220002	0.0068	22210120
0.0040	22111111	0.0088	22022020	0.0064	22211101
0.0030	22201102	0.0063	22221001	0.0034	22111111
0.0029	22212100	0.0052	22220020	0.0033	22220110
0.0028	21221101	0.0037	22121011	0.0030	21221101

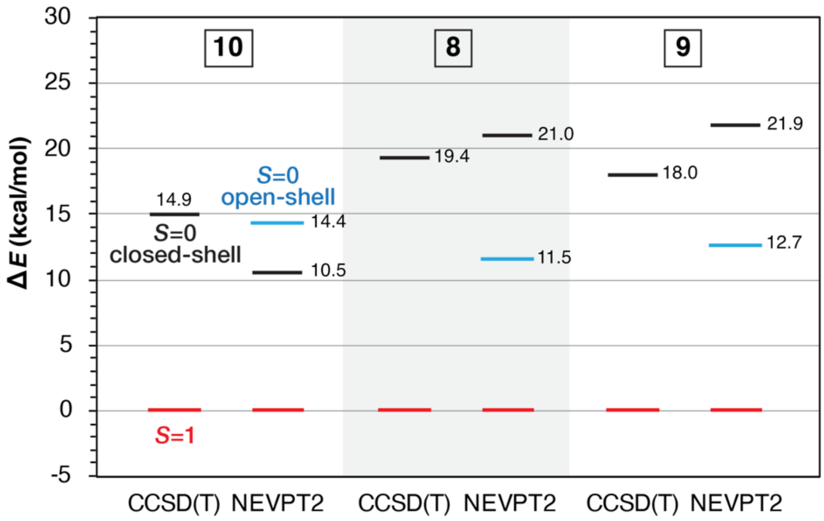

Fig. S133: Vertical energy differences between the ground triplet states (red) and the excited closed-shell (black) and open-shell (blue) singlet states of metallovinylidenes **8** and **9**, compared to those of the free vinylidene **10**, obtained from different levels of theory.

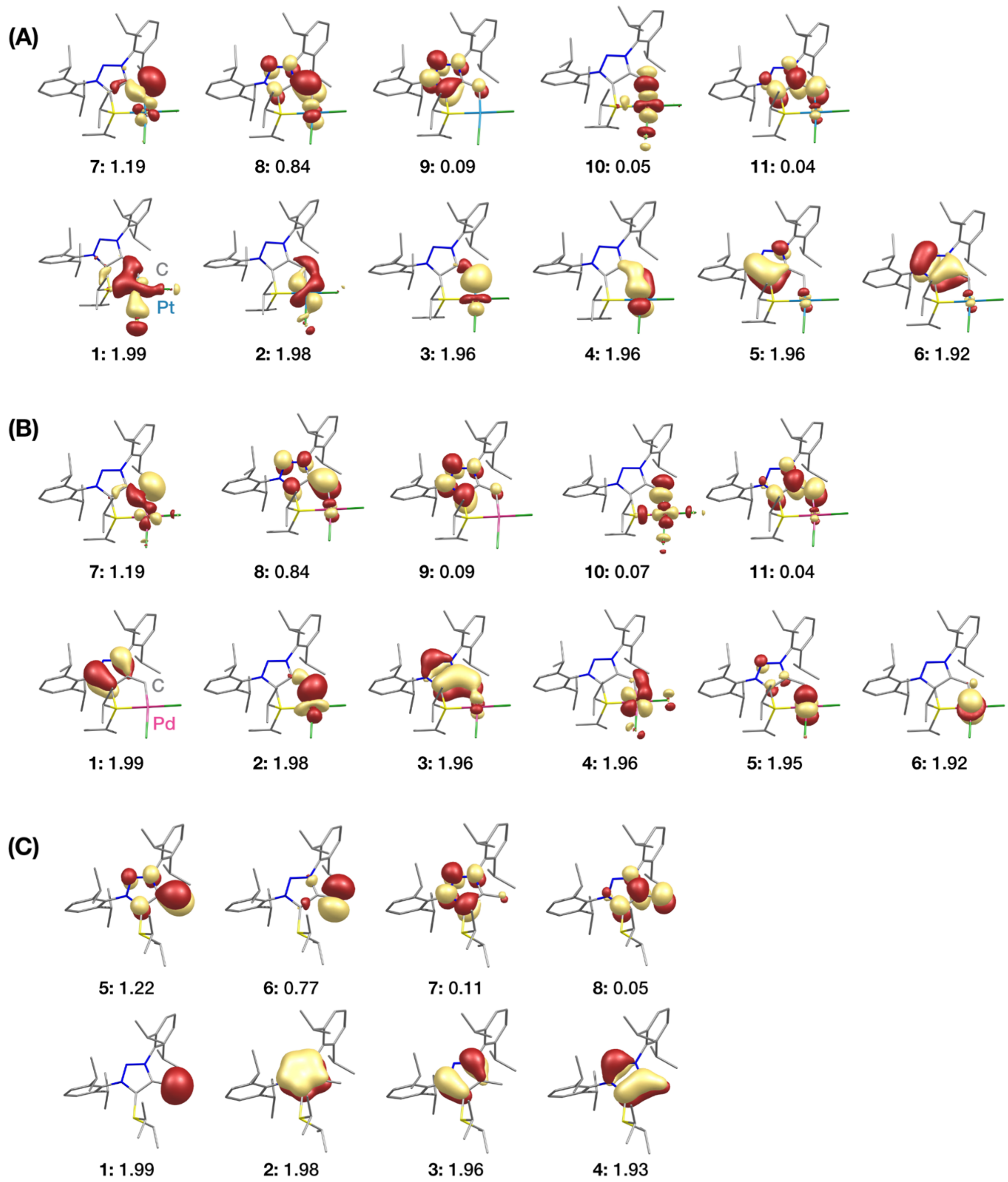

Fig S134: Active orbitals and natural occupation numbers of A) **8**, produced from a CASSCF(14,11) calculation averaged over 1 triplet and 2 singlet states, B) **9**, from a CASSCF(14,11) calculation averaged over 1 triplet and 2 singlet states, and C) **10**, from a CASSCF(10,8) calculation averaged over 1 triplet and 2 singlet states.

12.3 Natural Bond Orbital (NBO) Analysis

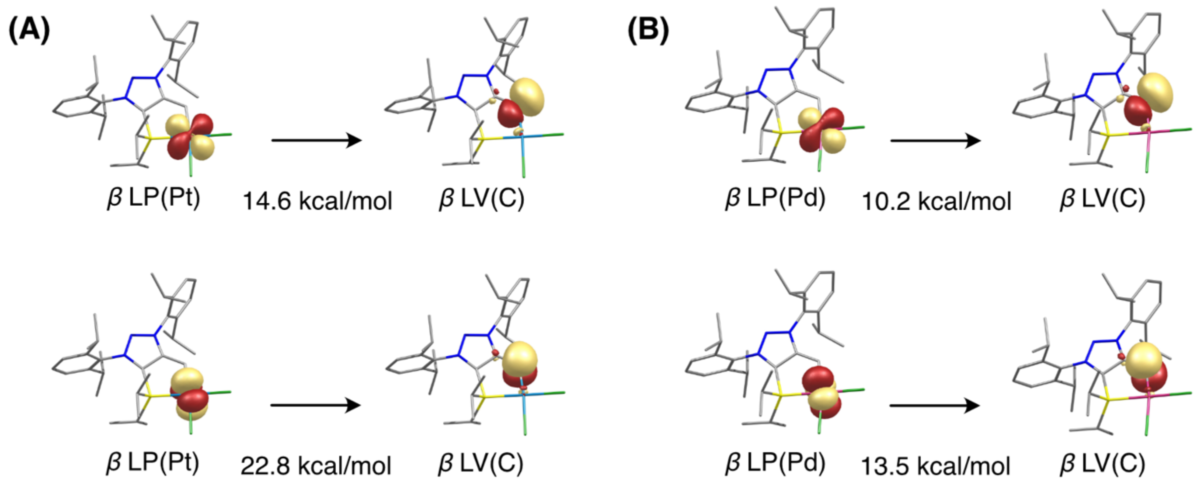

Fig S135: Relevant donor-acceptor interactions from NBO second-order perturbation analysis of (A) **8** and (B) **9**. The NBO analysis was performed on PBE0 results.

12.4 ^{31}P and ^{13}C Hyperfine Coupling Constants

Table S11: Calculated (TPSSh) hyperfine coupling tensors (in MHz) of ^{31}P for **10**, **8**, and **9** and of ^{13}C for **10**, **8**, **9**, and the metallocarbene Pt−C−SiMe$_3$ (see main text). The tensors are analyzed in terms of the isotropic (A_{iso}) and dipolar ($T_{x,y,z}$) contributions.

		A_x	A_y	A_z	A_{iso}	T_x	T_y	T_z
^{31}P	**10**	0.43	-1.57	-3.33	-1.49	-1.92	0.08	1.84
	**8**	0.19	-3.73	-4.65	-2.73	-2.91	1.00	1.92
	**9**	-7.35	-8.19	-10.62	-8.72	-1.37	-0.53	1.90
^{13}C	**10**	-12.24	55.60	93.86	45.74	57.98	-9.86	-48.12
	**8**	110.30	175.12	202.38	162.60	52.30	-12.52	-39.78
	**9**	104.11	171.00	194.39	156.50	52.39	-14.50	-37.88
	Pt−C−SiMe$_3$	-0.29	90.57	98.50	62.93	63.22	-27.64	-35.57

12.5 Zero Field Splitting

Table S12: Breakdown of contributions to the D value from excited states of compound **8**, along with their leading configurations obtained by CASSCF(14,11)/NEVPT2 calculations with 22 singlets and 22 triplets. The orbitals that describe the configurations are depicted in Figure S132. Many states of different nature contribute to the zero-field splitting, with the most important contributions noted in **bold**.

Excited State	Energy (cm^{-1})	Leading Configuration	Contribution to D (cm^{-1})
S = 1	5841	22222211000	0.0
S = 1	7724	22222220000	0.1
S = 1	9429	**22222202000**	**69.4**
S = 1	12988	**22222210100**	**3.3**
S = 1	24770	**22222112000**	**-1.4**
S = 1	27148	**22212221000**	**-17.8**
S = 1	27882	**22222121000**	**-14.1**
S = 1	30361	22222201100	-0.4
S = 1	33046	22212221000	0.3
S = 1	33442	22222111100	-0.5
S = 1	35278	**22221221000**	**-14.8**
S = 1	36656	22212212000	0.7
S = 1	37065	**22222120100**	**5.2**
S = 1	38513	22212220100	-0.1
S = 1	40131	22221220100	1.6
S = 1	40444	**22221211100**	**14.0**
S = 1	40854	22212211100	0.3
S = 1	41260	22212211100	0.8
S = 1	42113	22222210010	-1.0
S = 1	44162	22221211100	0.3
S = 1	53223	22222102100	-0.1
S = 1	53981	22221202100	0.0
S = 3	0	22222211000	0.0
S = 3	13345	**22222210100**	**55.9**
S = 3	22112	22222201100	-0.1
S = 3	23933	**22222112000**	**100.2**
S = 3	27438	**22212212000**	**-52.5**
S = 3	27666	22222121000	-0.5
S = 3	28121	22212212000	0.2
S = 3	30003	**22221221000**	**-8.9**
S = 3	31652	22222111100	-0.9
S = 3	33177	22212221000	-0.1
S = 3	34124	22221211100	-0.9

S = 3	35351	22222111100	-0.8
S = 3	35862	22212220100	-0.1
S = 3	37073	**22222120100**	**-13.3**
S = 3	37253	22221220100	-1.1
S = 3	37268	**22222210010**	**-6.0**
S = 3	37562	22212211100	-0.7
S = 3	40260	**22212220100**	**8.9**
S = 3	42076	22222111100	0.0
S = 3	43152	22222201010	-0.7
S = 3	43639	22212211100	-0.3
S = 3	46653	22122211100	0.0

Table S13: Breakdown of contributions to the *D* value from excited states of compound **9**, along with their leading configurations obtained by CASSCF(14,11)/NEVPT2 calculations with 35 singlets and 35 triplets. The orbitals that describe the configurations are depicted in Figure S132. Multiple states of different nature contribute to the zero-field splitting, with the most important contributions noted in **bold**.

Excited State	Energy (cm^{-1})	Leading Configuration	Contribution to *D* (cm^{-1})
S = 1	4947	22222211000	0.0
S = 1	7751	**22222202000**	**5.1**
S = 1	11737	22222202000	0.0
S = 1	15659	22222210100	0.0
S = 1	23786	**22222112000**	**2.0**
S = 1	24650	**22222121000**	**7.0**
S = 1	25787	**22212221000**	**8.1**
S = 1	28888	**22222210010**	**-2.4**
S = 1	29415	22221212000	0.0
S = 1	32516	22222201100	0.0
S = 1	34015	22222111100	0.0
S = 1	36318	22222120100	0.0
S = 1	36376	22212212000	0.0
S = 1	36848	22212221000	0.2
S = 1	38110	22212220100	0.3
S = 1	38847	22212220010	0.0
S = 1	40827	22221220100	0.2
S = 1	41334	22222111010	0.0
S = 1	41507	22212211100	0.2
S = 1	42083	22222210001	0.1
S = 1	42945	22222111100	0.6
S = 1	43211	**22221211100**	**2.0**

S = 1	43243	22221211100	0.0
S = 1	44449	22222111010	0.0
S = 1	45356	22221211010	0.0
S = 1	46248	22222102010	0.0
S = 1	46391	22122212000	0.0
S = 1	48277	22212210110	0.1
S = 1	49025	22212211010	0.0
S = 1	51542	22221210110	0.0
S = 1	51626	22221202010	0.1
S = 1	52576	22222120010	0.0
S = 1	53266	22222110110	0.0
S = 1	55209	22221210110	0.0
S = 1	56733	22212202010	-0.2
S = 3	0	22222211000	0.0
S = 3	15507	**22222210100**	**-1.2**
S = 3	21919	**22222112000**	**8.0**
S = 3	23474	22222201100	0.0
S = 3	24067	22222121000	-0.2
S = 3	26128	**22212221000**	**-2.0**
S = 3	26311	**22221212000**	**-8.4**
S = 3	26761	**22212212000**	**-2.2**
S = 3	27049	**22221221000**	**-4.6**
S = 3	28681	22221221000	0.0
S = 3	33069	22222111100	0.0
S = 3	33698	22212211010	0.0
S = 3	35210	22222111010	0.1
S = 3	35649	22221211100	-0.1
S = 3	35985	22222111010	0.0
S = 3	36417	22222120100	0.0
S = 3	38434	22212220100	-0.3
S = 3	39424	22222111010	0.0
S = 3	39580	22212211100	0.0
S = 3	39866	22221220100	-0.1
S = 3	40614	22221211100	-0.3
S = 3	41231	22222111100	0.0
S = 3	41247	22222210001	0.0
S = 3	42161	22212211010	0.0
S = 3	42550	22222210001	0.0
S = 3	43074	22221211010	-0.1
S = 3	43486	22222102010	0.0
S = 3	43711	22212220010	0.0
S = 3	44741	22222111010	-0.3
S = 3	45135	22221211100	0.0

S = 3	45172	22221211100	0.0
S = 3	45994	22122211100	0.0
S = 3	46716	22212211100	-0.5
S = 3	47786	22222120010	0.0
S = 3	48193	22222110110	0.0

13. Literature

[1] N. Nimitsiriwat, V. C. Gibson, E. L. Marshall, P. Takolpockdee, A. K. Tomov, A. J. P. White, D. J. Williams, M. R. J. Elsegood, S. H. Dale, *Inorg. Chem.* **2007**, *46*, 9988–9997; b) A. G. Barrett, M. R. Crimmin, M. S. Hill, P. B. Hitchcock, G. Kociok-Köhn, P. A. Procopiou, *Inorg. Chem.* **2008**, *47*, 7366–7376.

[2] J. Bouffard, B. K. Keitz, G. Guisado-Barrios, G. Frenking, R. H. Grubbs, G. Bertrand, *Organometallics* **2011**, 30, 2617-2627.

[3] Burkhardt, A., Pakendorf, T., Reime, B., Meyer, J., Fischer, P., Stübe, N., Panneerselvam, S., Lorbeer, O., Stachnik, K., Warmer, M., et al. (2016). Status of the crystallography beamlines at PETRA III. Eur. Phys. J. Plus. *131*, 56-64.

[4] Kabsch, W. (2010). XDS. Acta Crystallogr. D Biol. Crystallogr. *66*, 125–132.

[5] Sheldrick, G.M. (2015). SHELXT – Integrated space-group and crystal-structure determination. Acta Crystallogr. Sect. Found. Adv. *71*, 3–8.

[6] Sheldrick, G.M. (2015). Crystal structure refinement with SHELXL. Acta Crystallogr. C Struct. Chem. *71*, 3–8.

[7] Hübschle, C.B., Sheldrick, G.M., and Dittrich, B. (2011). ShelXle: a Qt graphical user interface for SHELXL. J. Appl. Crystallogr. *44*, 1281–1284.

[8] Thorn, A., Dittrich, B., and Sheldrick, G.M. (2012). Enhanced rigid-bond restraints. Acta Crystallogr. Sect. Found. Crystallogr. *68*, 448–451.

[9] Spek, A.L. (2015). PLATON SQUEEZE: a tool for the calculation of the disordered solvent contribution to the calculated structure factors. Acta Crystallogr. C Struct. Chem. *71*, 9–18.

[10] Spek, A.L. (2009). Structure validation in chemical crystallography. Acta Crystallogr. D Biol. Crystallogr. *65*, 148–155.

[11] E. R. Davies, *Phys. Lett. A* **1974**, *47*, 1–2

[12] W. Bruggemann, J. R. Niklas, *J. Magn. Reson., Ser. A* **1994**, *108*, 25–29

[13] S. Stoll, A. Schweiger, *J. Magn. Reson.* **2006**, *178*, 42–55.

[14] Y. Kutin, J. Reitz, M. Drosou, P. W. Antoni, Y. He, V. R. Selve, S. Boschmann, A. Savitsky, D. A. Pantazis, M. Kasanmascheff, M. M. Hansmann, JACS Au **2025**, *6*, 2884-2897.

[15] J. Nehrkorn, K. Holldack, R. Bittl, A. Schnegg, *J. Magn. Reson.* **2017**, *280*, 10–19.

[16] A. Schweiger, S. Stoll *J. Magn. Reson.* **2006**, 178, 42 – 55.

[17] J. Nehrkorn, J. Telser, K. Holldack, S. Stoll, A. Schnegg, *J. Phys. Chem. B*, **2015**, *119*, 13816 – 13824.

[18] F. Neese, F. Wennmohs, U. Becker, C. Riplinger, *J. Chem. Phys.* **2020**, *152*, 224108.

[19] C. Adamo, V. Barone, *J. Chem. Phys.* **1999**, *110*, 6158-6170.

[20] M. Bühl, C. Reimann, D. A. Pantazis, T. Bredow, F. Neese, *J. Chem. Theory Comput.* **2008**, *4*, 1449-1459.

[21] E. van Lenthe, E. J. Baerends, J. G. Snijders, *J. Chem. Phys.* **1993**, *99*, 4597-4610.

[22] E. van Lenthe, E. J. Baerends, J. G. Snijders, *J. Chem. Phys.* **1994**, *101*, 9783-9792.

[23] C. van Wüllen, *J. Chem. Phys.* **1998**, *109*, 392-399.

[24] E. van Lenthe, J. G. Snijders, E. J. Baerends, *J. Chem. Phys.* **1996**, *105*, 6505-6516.

[25] D. A. Pantazis, X.-Y. Chen, C. R. Landis, F. Neese, *J. Chem. Theory Comput.* **2008**, *4*, 908-919.

[26] F. Weigend, R. Ahlrichs, *Phys. Chem. Chem. Phys.* **2005**, *7*, 3297-3305.

[27] F. Neese, F. Wennmohs, A. Hansen, U. Becker, *J. Chem. Phys.* **2009**, *356*, 98-109.

[28] C. Riplinger, F. Neese, *J. Chem. Phys.* **2013**, *138*, 034106.

[29] C. Riplinger, B. Sandhoefer, A. Hansen, F. Neese, *J. Chem. Phys.* **2013**, *139*, 134101.

[30] Y. Guo, C. Riplinger, U. Becker, D. G. Liakos, Y. Minenkov, L. Cavallo, F. Neese, *J. Chem. Phys.* **2018**, *148*, 011101.

[31] Y. Guo, C. Riplinger, D. G. Liakos, U. Becker, M. Saitow, F. Neese, *J. Chem. Phys.* **2020**, *152*, 024116.

[32] D. G. Liakos, M. Sparta, M. K. Kesharwani, J. M. L. Martin, F. Neese, *J. Chem. Theory Comput.* **2015**, *11*, 1525-1539.

[33] A. Altun, F. Neese, G. Bistoni, *J. Chem. Theory Comput.* **2020**, *16*, 6142-6149.

[34] M. Drosou, C. A. Mitsopoulou, D. A. Pantazis, *Polyhedron* **2021**, *208*, 115399.

[35] M. Drosou, C. A. Mitsopoulou, D. A. Pantazis, *J. Chem. Theory Comput.* **2022**, *18*, 3538-3548.

[36] C. Angeli, R. Cimiraglia, S. Evangelisti, T. Leininger, J.-P. Malrieu, *J. Chem. Phys.* **2001**, *114*, 10252-10264.

[37] C. Angeli, R. Cimiraglia, J.-P. Malrieu, *Chem. Phys. Lett.* **2001**, *350*, 297-305.

[38] Y. Pang, N. Nöthling, M. Leutzsch, L. Kang, E. Bill, M. van Gastel, E. Reijerse, R. Goddard, L. Wagner, D. SantaLucia, S. DeBeer, F. Neese, J. Cornella, *Science* **2023**, *380*, 1043-1048.

[39] B. A. Heß, C. M. Marian, U. Wahlgren, O. Gropen, *Chem. Phys. Lett.* **1996**, *251*, 365-371.
[40] F. Neese, *J. Chem. Phys.* **2005**, *122*, 034107.
